# The long and the short of Huntington’s disease: how the sphingolipid profile is shifted in the caudate of advanced clinical cases

**DOI:** 10.1093/braincomms/fcab303

**Published:** 2021-12-23

**Authors:** Gabrielle R. Phillips, Jennifer T. Saville, Sarah E. Hancock, Simon H. J. Brown, Andrew M. Jenner, Catriona McLean, Maria Fuller, Kelly A. Newell, Todd W. Mitchell

**Affiliations:** Illawarra Health and Medical Research Institute, Wollongong, NSW 2522, Australia; School of Medicine, University of Wollongong, Wollongong, NSW 2522, Australia; Molecular Horizons, University of Wollongong, Wollongong, NSW 2522, Australia; Genetics and Molecular Pathology, SA Pathology at Women’s and Children’s Hospital, North Adelaide, SA 5006, Australia; School of Medical Sciences, University of New South Wales, Sydney, NSW 2052, Australia; Molecular Horizons, University of Wollongong, Wollongong, NSW 2522, Australia; School of Chemistry and Molecular Biosciences, University of Wollongong, Wollongong, NSW 2522, Australia; Bioanalytical Mass Spectrometry Facility, Mark Wainwright Analytical Centre, University of New South Wales, Sydney, NSW 2052, Australia; Department of Anatomical Pathology, Alfred Health and Florey Neuroscience, Parkville, VIC 3052, Australia; Genetics and Molecular Pathology, SA Pathology at Women’s and Children’s Hospital, North Adelaide, SA 5006, Australia; Adelaide Medical School, University of Adelaide, Adelaide, SA 5000, Australia; Illawarra Health and Medical Research Institute, Wollongong, NSW 2522, Australia; School of Medicine, University of Wollongong, Wollongong, NSW 2522, Australia; Molecular Horizons, University of Wollongong, Wollongong, NSW 2522, Australia; Illawarra Health and Medical Research Institute, Wollongong, NSW 2522, Australia; School of Medicine, University of Wollongong, Wollongong, NSW 2522, Australia; Molecular Horizons, University of Wollongong, Wollongong, NSW 2522, Australia

**Keywords:** Huntington's disease, glycosphingolipid, sphingolipid, mass spectrometry, striatum

## Abstract

Huntington’s disease is a devastating neurodegenerative disorder that onsets in late adulthood as progressive and terminal cognitive, psychiatric and motor deficits. The disease is genetic, triggered by a CAG repeat (polyQ) expansion mutation in the Huntingtin gene and resultant huntingtin protein. Although the mutant huntingtin protein is ubiquitously expressed, the striatum degenerates early and consistently in the disease. The polyQ mutation at the N-terminus of the huntingtin protein alters its natural interactions with neural phospholipids *in vitro*, suggesting that the specific lipid composition of brain regions could influence their vulnerability to interference by mutant huntingtin; however, this has not yet been demonstrated *in vivo*. Sphingolipids are critical cell signalling molecules, second messengers and membrane components. Despite evidence of sphingolipid disturbance in Huntington’s mouse and cell models, there is limited knowledge of *how* these lipids are affected in human brain tissue. Using post-mortem brain tissue from five brain regions implicated in Huntington’s disease (control *n* = 13, Huntington’s *n* = 13), this study aimed to identify *where* and *how* sphingolipid species are affected in the brain of clinically advanced Huntington’s cases. Sphingolipids were extracted from the tissue and analysed using targeted mass spectrometry analysis; proteins were analysed by western blot. The caudate, putamen and cerebellum had distinct sphingolipid changes in Huntington’s brain whilst the white and grey frontal cortex were spared. The caudate of Huntington’s patients had a shifted sphingolipid profile, favouring long (C13–C21) over very-long-chain (C22–C26) ceramides, sphingomyelins and lactosylceramides. Ceramide synthase 1, which synthesizes the long-chain sphingolipids, had a reduced expression in Huntington’s caudate, correlating positively with a younger age at death and a longer CAG repeat length of the Huntington’s patients. The expression of ceramide synthase 2, which synthesizes very-long-chain sphingolipids, was not different in Huntington’s brain. However, there was evidence of possible post-translational modifications in the Huntington’s patients only. Post-translational modifications to ceramide synthase 2 may be driving the distinctive sphingolipid profile shifts of the caudate in advanced Huntington’s disease. This shift in the sphingolipid profile is also found in the most severely affected brain regions of several other neurodegenerative conditions and may be an important feature of region-specific cell dysfunction in neurodegenerative disease.

## Teaser

The caudate, a region of targeted neurodegeneration in Huntington’s disease, has a shift in the relative abundance of sphingolipid species influenced by fatty acyl chain length. Long-chain species are favoured over very-long-chain species in multiple classes. Modifications to ceramide synthases may underlie these shifts.

## Graphical Abstract

**Graphical Abstract:**
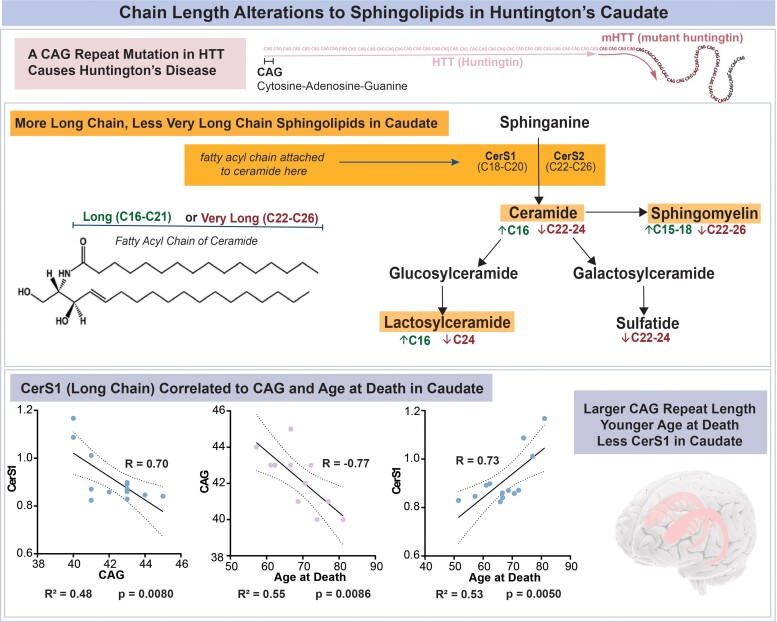

## Introduction

Huntington’s disease is an autosomal, dominant, neurodegenerative disease caused by a CAG repeat mutation in the Huntingtin gene (*HTT*). This mutation results in a polyglutamine expansion (polyQ) at the N-terminus of the huntingtin protein (HTT), referred to as mutant huntingtin (mHTT).^1^ The disease presents as progressive and terminal cognitive, psychiatric and motor disturbances lasting ∼15–20 years. Onset is typically in late adulthood; the age of onset is influenced by the CAG repeat length. Longer CAG repeat mutations are associated with an earlier age of onset, more severe clinical expression and an earlier age of death.^2,3^ HTT appears to play an important role in neurodevelopment,^4^ synaptic development, neuronal survival^5^ and transport,^6^ transcriptional regulation^6^ and autophagy^6,7^; however, it is still debated as to whether Huntington’s disease results from a ‘loss of function’ of HTT or a pathological ‘gain of function’ of mHTT.

Although mHTT is expressed ubiquitously in the brain, the atrophy of the striatum occurs early and severely in the disease.^8,9^ The cerebral cortex also atrophies, although not the same extent as the striatum.^9,10^ The striatum includes three smaller subregions: the caudate nucleus, putamen and nucleus accumbens. It is a component of the basal ganglia and has roles in voluntary movement, cognition, learning and memory.^11,12^ The caudate and putamen atrophy differently in Huntington’s disease,^8,13^ and correlate with different disease indices (caudate with the age of onset, putamen with disease severity).^9^ The underlying cause of the striatum’s vulnerability to mHTT is unknown.^14^ However, its dominant cell type, medium spiny neurons, are believed to be a significant contributing factor. HTT associates with phospholipids in neural cell cultures and its preferences and interactions are altered by the polyglutamine mutation on the N-terminus.^15,16^ Brain regions have distinct lipid compositions,^17^ tailored by their unique cell populations, neural connections and functional requirements.^18^ These compositions can create vulnerabilities to specific pathological triggers. For example, grey matter regions that are more caudal have a higher polyunsaturated fatty acid content and are therefore more susceptible to lipid oxidation and mitochondrial stress.^17^ Region-specific alterations to lipids and their metabolic genes, including sphingolipids, are a feature of the striatum in Huntington’s disease.^19–22^

**Figure 1:**
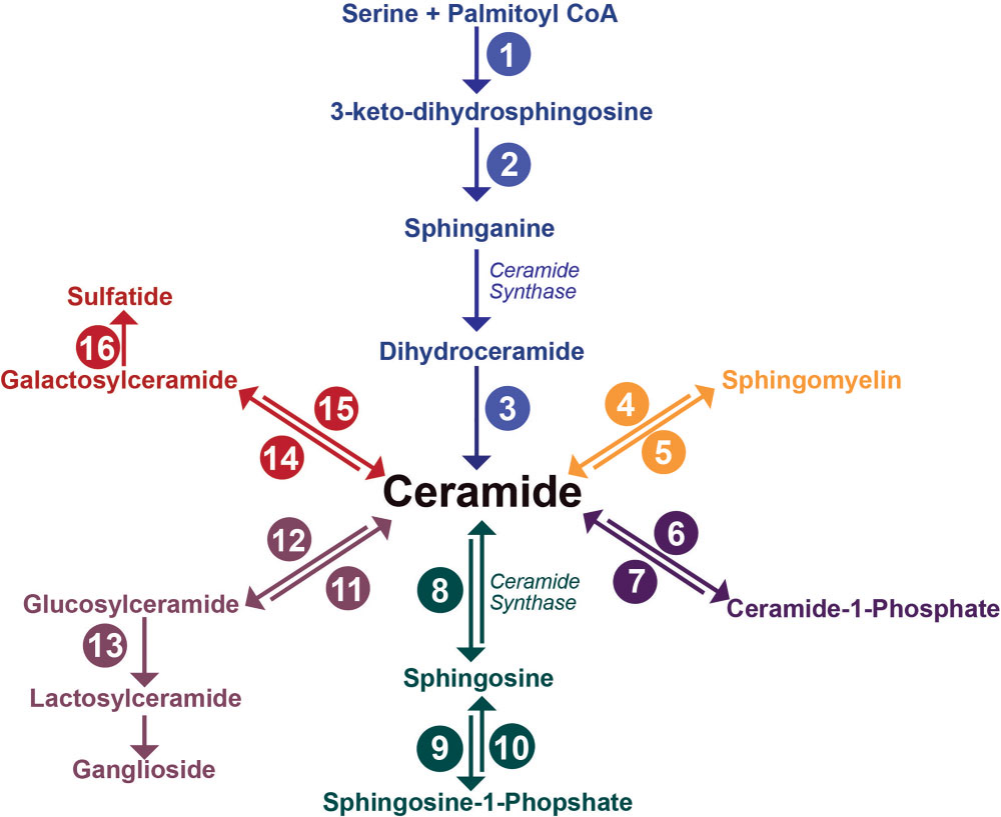
**Sphingolipid metabolic pathways.** Ceramide is the metabolic hub with synthesis available via the *de novo* pathway (blue), via the hydrolysis of sphingomyelin (orange) or via the salvage of more complex sphingolipids (red, lavender, forest green). Ceramides become glycosphingolipids via either conversion to glucosylceramide (lavender) or galactosylceramide (red). Ceramide synthases have been labelled to highlight their position in the pathways, with additional enzymes, numbered 1–16. 1, Serine palmitoyltransferase; 2, 3-ketosphinganine reductase; 3, dihydroceramide desaturase; 4, sphingomyelinase; 5, sphingomyelin synthase; 6, ceramide kinase; 7, ceramide phosphatase; 8, ceramidase; 9, sphingosine kinase; 10, sphingosine-1-phosphatase; 11, glucosylceramidase; 12, glucosylceramide synthase; 13, lactosylceramide synthase; 14, galactosylceramidase; 15, ceramide galactosyltransferase; 16, cerebroside sulphotransferase.

Sphingolipids are characterized by their sphingoid base and are involved in many key cellular and neural processes, including cell signalling, membrane formation and organization, inflammation, immune response, myelination and regulation of neurotransmitters.^23–27^ Ceramides (Cer) are the key building block for complex sphingolipids and lie at the centre of multiple synthesis and recycling pathways (Fig. 1). In the *de novo* synthesis pathway, Cer is formed via the acylation of sphinganine by one of six tissue-specific isoforms of ceramide synthase (CerS) (Fig. 1). Each of these isoforms has an affinity for different fatty acyl chain lengths: CerS1 (C18), CerS2 (C22–C24), CerS3 (C18 and C24), CerS4 (C18–C20), CerS5 and CerS6 (C14–C16).^27–29^ Fatty acids can be grouped according to their carbon chain length: short (≤C5), medium (C6–C12), long (C13–C21) and very long (≥22C). The length of the fatty acyl chain is an important factor contributing to the biological function and cellular location of lipids.^30–32^ CerS1 (RNA) is the most expressed isoform in the central nervous system and is primarily found in neurons.^33^ CerS2 is the second most abundant, with its high RNA expression observed in oligodendrocytes during periods of active myelination. CerS2 null mice show reduced abundances of very-long-chain sphingolipids.^34^ However, current knowledge of CerS relies heavily on murine studies.^28,35,36^ To date, few studies have examined the expression of CerS^37^ in human brain tissue or Huntington’s disease.

Neural Cer concentrations are low due to their constant metabolism. Sphingomyelin (SM) is synthesized from Cer via the addition of a phosphatidylcholine head group by SM synthase (Fig. 1) and has a much higher abundance in the brain. This makes SM useful as a reserve for Cer production.^26^ SM is primarily synthesized in oligodendrocytes and is an essential component of cell and myelin membranes. Due to increased oligodendroglial densities in Huntington’s disease, there may be consequential increases in SM.^38,39^

Cer can also be metabolized into more complex glycosphingolipids (Fig. 1). Glycosphingolipids are associated with crucial neuronal development and myelination periods and act as second messengers for cell signalling.^40^ Glucosylceramide and galactosylceramide are the simplest glycosphingolipids, arising from the addition of either a glucose or galactose ring to the terminal hydroxyl group of Cer.^23^ The addition of galactose to the existing glucose ring of glucosylceramide creates lactosylceramide (LacCer), an important precursor to gangliosides.^23^ On the other hand, galactosylceramide enters a different synthesis pathway to be converted to sulphatides by the addition of a sulphur group.^23^ Galactosylceramides and sulphatides contribute significantly to myelin and are proposed to assist myelin membrane stability and saltatory conduction.^40^

Multiple experimental models of Huntington’s disease have disturbances to sphingolipid metabolism, notably to sphingosine-1-phosphate, a precursor to Cer production.^19^ Transgenic R6/1 Huntington’s mice, despite unchanged total abundances of glycosphingolipids, have reductions in specific glycosphingolipid species (GM1 gangliosides) in their neurons.^22,41^ These mice also have alterations to several glycan-transferases, ganglioside degradation proteins^22,41^ and CerS.^19^ Post-mortem Huntington’s disease frontal cortex and caudate have extracellular deposits of cerebrosides, akin to those found in cerebroside storage disorders (Gaucher’s disease and Krabbe’s disease) and multiple sclerosis patients.^42^ The neighbouring subventricular zone, which borders the caudate, has increased abundancies of SM and deficiencies of sulphatides in Huntington’s disease patients.^43^

Despite the many disturbances to sphingolipid metabolism observed in Huntington’s disease models, an understanding of molecular-level and region-specific effects on the expression of sphingolipids in human Huntington’s disease tissue is lacking. Region-specific alterations to cholesterol metabolism have already been identified in Huntington’s striatum,^20,44^ and our research aimed to determine if these alterations extended to region-specific effects on sphingolipids in Huntington’s disease. For the first time, we show variations in the availability of sphingolipids governed by the length of their fatty acyl chain in Huntington’s caudate, a feature consistent with several other neurodegenerative diseases.^37,45,46^

## Materials and methods

### Human brain tissue

The Victorian Brain Bank provided post-mortem brain tissue from 13 advanced Huntington’s disease subjects and 13 age- and sex-matched controls (male *n* = 8, female *n* = 5/group). The tissue provided was from five key regions: caudate, putamen, cerebellum, and grey and white dorsomedial prefrontal cortex. Subject demographics are provided in Table 1. The clinical disease stage was measured using the Unified Huntington’s Disease Rating Scale. All Huntington’s disease tissue had a pathological Vonsattel grading of IV, the most severe.^47^ The CAG repeat of Huntington’s patients was assessed by the Victorian Clinical Genetics Service (2020). The post-mortem interval, brain pH and age were not significantly different between Huntington’s disease and control brains.^20^ The tissue was stored at −80°C until use. Ethics approval was obtained from the UOW Human Research Ethics Committee (HE10/327) and was carried out in accordance with the Declaration of Helsinki (2008).

**Table 1:** Subject demographics of post-mortem tissue

Subject	Sex	Age at death (years)	Post-mortem interval (hours)	pH	CAG	Cause of death
CON 1	M	78.3	46.0	6.54	–	Cardiomegaly, myocardial fibrosis, ischaemic coronary artery disease
CON 2	M	69.1	34.0	6.31	–	Ischaemic heart disease
CON 3	M	63.9	54.5	6.51	–	Ischaemic heart disease, coronary artery atherosclerosis
CON 4	M	81.0	36.5	6.56	–	Ischaemic heart disease, coronary artery disease, hypertension, hyperlipidaemia
CON 5	M	64.1	24.0	6.56	–	ischaemic heart disease, coronary artery atherosclerosis
CON 6	F	59.0	30.0	6.84	–	Asthma
CON 7	F	67.3	30.0	6.23	–	Pulmonary thromboembolism, deep vein thrombosis
CON 8	F	74.8	61.5	6.24	–	Coronary artery atherosclerosis
CON 9	F	68.3	71.5	6.34	–	Pulmonary thromboembolism, thrombosis of left calf
CON 10	F	60.4	49.0	6.23	–	Myocardial infarction
CON 11	M	63.9	32.0	6.47	–	Ischaemic heart disease
CON 12	M	69.4	24.0	6.27	–	Pulmonary thromboembolism, left leg thrombosis, cardiomegaly
CON 13	M	75.6	46.0	6.57	–	Ruptured abdominal aortic aneurysm
Mean (SEM)		68.8 (1.9)	41.5 (4.1)	6.44 (0.05)	–	
HD 1	M	77.0	8.5	6.57	41	Huntington’s disease, pneumonia
HD 2	M	68.7	72.0	6.32	41	Pneumonia, Huntington’s disease, ethanol abuse
HD 3	M	61.1	17.0	6.54	43	Huntington’s disease
HD 4	M	81.1	50.5	6.23	40	Huntington’s disease
HD 5	M	66.6	37.0	6.26	43	Huntington’s disease
HD 6	F	57.2	22.0	6.22	44	Bronchopneumonia aspiration, Huntington’s disease
HD 7	F	66.7	18.5	6.21	45	Pneumonia, Huntington’s disease
HD 8	F	72.2	22.0	6.43	44	Pneumonia, Huntington’s disease
HD 9	F	70.7	70.0	6.16	42	Huntington’s disease
HD 10	F	51.5	63.5	6.54	43	Sudden unexplained (Huntington’s disease for 21.5 years)
HD 11	M	62.3	21.5	6.62	43	Huntington’s disease, diabetes (2 years)
HD 12	M	65.9	58.0	6.33	41	Drowning (depression)
HD 13	M	73.9	26.5	6.31	40	Cardiac arrest, aortic stenosis and dementia, Huntington’s disease
Mean (SEM)		67.3 (2.2)	37.5 (6.2)	6.36 (0.04)	42.31 (0.44)	

Sex, age at death, post-mortem interval, pH and Vonsattel pathological grade were provided by the Victorian Brain Bank. CAG repeat lengths were determined by the Victorian Clinical Genetics Service. CAG repeat lengths are specific to the HD causing allele (>39 CAG) only and therefore controls are not included. CON, Control; HD, Huntington’s disease; SEM, standard error of mean.

### Lipid nomenclature

Lipid nomenclature and abbreviations are consistent with recommendations for sphingolipids.^48^ Examples are provided in Supplementary Table 1. The fatty acid notation describes the number of carbons and double bond equivalents in the fatty acyl chain, i.e. 16:1 has 16 carbons and 1 double bond equivalent. Fig. 1 highlights sphingolipid metabolic pathways. The molecular structure of analysed sphingolipid classes is provided in Supplementary Fig. 2.

### Lipid extractions

#### Ceramide and sphingomyelin

Lipids were extracted as described previously.^49,50^ Brain tissue (10 mg) was homogenized using a bead homogeniser (Fast-Prep 24, MP Bio, Sydney, Australia) at 6 m/s for 40 s, using 600 mg of 1.4 mm ceramic beads in 300 µl of methanol [liquid chromatography-mass spectrometry (LC-MS) grade; Bio-Strategy, Murarrie, Australia] containing 0.01% butylated hydroxyl-toluene (BHT; Sigma Aldrich, MO, USA) and internal standards (1 nmol Cer 17:0, 5 nmol dihydrosphingomyelin d18:0/12:0; Avanti Polar Lipids, AL, USA). The homogenate was transferred into 2 ml glass vials, 920 µl methyl tert-butyl ether [high-performance liquid chromatography (HPLC) grade; Bio-Strategy, Murarrie, Australia] was added and the samples were rotated for 1 h at room temperature. Ammonium acetate (HPLC grade; Sigma Aldrich, Castle Hill, Australia) was added (230 µl of 150 mM) and the samples vortexed for 20 s before being centrifuged at 2000 × *g* for 5 min. The top organic phase was removed from each sample without disturbing the bottom aqueous phase and transferred into a new 2 ml glass vial before storage at −20°C.

To enhance sphingolipid detection, lipid extracts (300 µl) were subjected to base hydrolysis to remove glycerophospholipids.^51,52^ To the extract, 90 µl of methanol (0.01% BHT) and 25 µl of 10 M sodium hydroxide (Bio-Strategy, Murarrie, Australia) were added and the samples rotated overnight at 4°C. Following this, 90 µl of 150 mM ammonium acetate was added and the samples were vortexed before centrifugation at 2000 × *g* for 5 min. The top phase was removed (∼100 µl) and stored in new glass vials at −20°C. Extracts were diluted 100-fold in methanol:chloroform (LC-MS grade; Bio-Strategy, Murarrie, Australia) (2:1 v/v with 5 mM ammonium acetate) for mass spectrometric analysis of Cer and SM.

#### Glycosphingolipids

Glycosphingolipids were extracted from brain regions as previously described.^53,54^ Brain tissue was homogenized by sonication probe (Misonix; Farmingdale, NY, USA) in 0.02 M Tris (pH 7) containing 0.5 M NaCl and 0.1% Nonidet P-40. Total protein was determined by the method of Lowry *et al.*^55^ and lipids were extracted from 0.1 mg protein in 10 µl by addition of 0.2 ml chloroform:methanol (2:1) containing 10 pmol of LacCer 18:1;O2/16:0 (*d*_3_), galactosylceramide 18:1;O2/15:0 and trihexosylceramide 18:1;O2/17:0 as internal standards. All standards were purchased from Matreya LLC (State College, PA, USA). Samples were vortexed before being shaken on a platform shaker (10 min, 150 opm), sonicated in a water bath for 30 min and allowed to stand at room temperature for 20 min. Samples were then centrifuged (10 min, 13 000 × *g*) to sediment protein and the supernatant was transferred to a 96-well plate and dried under a gentle stream of nitrogen at 40°C. Dried samples were stored at −20°C until analysis by liquid chromatography coupled with electrospray ionization tandem mass spectrometry (LC-ESI-MS/MS).

### Mass spectrometry

#### Ceramide and sphingomyelin

Nanoelectrospray ionization mass spectrometry of lipid extracts was performed using a hybrid triple quadrupole linear ion trap mass spectrometer (QTRAP 5500, Sciex, Concord, Canada), equipped with an automated chip-based nanoelectrospray source (TriVersa Nanomate, Advion Biosciences, Ithaca, NY, USA) as described previously.^56^ Samples were loaded onto a 96-well plate (Eppendorf Twin-Tec 96) and sealed before direct infusion. Spray parameters were set at a gas pressure of 0.4 psi and a voltage of 1.2 kV.^50,56^ Declustering potential was set to 100 V, collision cell exit potential 8 V, entrance potential 10 V and scan rate at 200 Da/s. Lipid data were acquired by targeted precursor ion scans, as shown in Supplementary Table 2. Target lists for molecular lipid species within each class were generated after the manual review of spectra in Analyst (v1.6, Sciex, Ontario, Canada). Mass spectrometry data were analysed and quantified using LipidView software (v1.2, Sciex). Processing settings were set at a mass tolerance of 0.5 Da and minimum signal/noise of 10. Smoothing and deisotoping were enabled. Lipids were quantified by comparison of peak areas to class-specific internal standards after isotope correction.^57^

#### Glycosphingolipids

Glycosphingolipid analysis was performed by LC-ESI-MS/MS as previously described^54^ using a Shimadzu Nexera *x*2 LC system (Shimadzu Corp., Kyoto, Japan) coupled with a SCIEX QTRAP 6500 triple quadrupole mass spectrometer (SCIEX, Framingham, MA, USA). Samples were reconstituted in 100 µl of 10 mM ammonium formate in methanol and partial separation of the lipids was achieved by injection of 1 µl onto a Zorbax Eclipse C18 column (2.1 × 50 mm; 1.8 µm; Agilent Technologies) maintained at 40°C with an Agilent 1290 inline filter containing a 0.3 µm frit placed in front of the column. Mobile Phase A contained water:acetonitrile (60:40) and 10 mM ammonium formate, whilst mobile Phase B contained isopropanol:acetonitrile (90:10) with 10 mM ammonium formate. The flow rate was 0.4 ml/min and the column was equilibrated at 10% mobile Phase B before a linear ramp to 50% by 2 min and 100% B at 8 min. This was held for 0.5 min before a return to 10% B at 9 min and re-equilibration for 1 min before the next injection. The first 1 min was diverted to waste before being directed into the electrospray source (spray voltage 5.5 kV) in positive ion mode. The ion source temperature was 250°C, curtain gas was 25 units, collision gas set at medium; nebulizer gas 1 at 20 units and auxiliary gas 2 at 40 units.

In this method, the stereoisomers glucose and galactose cannot be separated and are reported using the generic term hexose (hex). Individual species of hexosylceramide (HexCer), dihexosylceramide (Hex2Cer) and hydroxylated sulphatide (SHexCer) were quantified using scheduled multiple reaction monitoring with concentrations determined in Multiquant 3.0.1 software (SCIEX, Framingham, MA, USA) by relating the peak area of the analyte to the peak area of the internal standard (noting that a trihexosylceramide internal standard was used to quantify SHexCer).

### Western blotting

Human brain samples were homogenized in buffer [0.1 M Tris–HCl, 2 mM ethylenediaminetetraacetic acid, glycerol 10% v/v, 0.5 mM phenylmethylsulphonyl fluoride, Protease Inhibitor Cocktail (P8340, Sigma, Australia) and Phosphatase Inhibitor Cocktail 2 (Sigma)] using a bead homogenizer (Fast-Prep 24, MP Bio) for 40 s at 6 m/s. Samples (12.5 µg total protein for CerS2, 10 µg protein for CerS1) were loaded on a 4–12% Criterion Stain-Free (Bio-Rad, CA, USA) gel in triplicate. A ‘pool’ of samples was created and loaded on each membrane to standardize measurements between the membranes. Electrophoresis was performed at 180 V for 50 min in sodium dodecyl sulphate–polyacrylamide gel electrophoresis buffer. The gels were washed in western transfer buffer (20% methanol) and transferred onto 0.2 µM polyvinylidene difluoride membranes at 100 V for 1 h. Membranes were washed and blocked in 5% skim milk in tris-buffered saline with Tween^®^ 20 (TBST) for 1 h at room temperature before overnight incubation at 4°C in the primary antibody {[anti-CerS 1 1:5000, (Recombinant) (ab131169); Abcam, Cambridge, UK] or [anti-CerS 2 1:1000 (ab176709), Abcam]} in 2.5% milk in TBST. Membranes were then washed in TBST and incubated in secondary antibody [goat × anti-rabbit (AP307P) 1:5000, Merck Millipore, MA, USA] in 2.5% milk in TBST for 1 h at room temperature. Membranes were washed in TBST before being visualized by chemiluminescence. Membranes were stripped with stripping buffer (ThermoFisher, MA, USA), washed in TBST, re-blocked and incubated with either glyceraldehyde 3-phosphate dehydrogenase (GAPDH) or β-actin before re-imaging. Expression of CerS1 and CerS2 was normalized to their relevant ‘pooled’ samples and housekeepers. Detailed antibody information is available in Supplementary Table 3.

### Statistical analysis

*A priori* sample size calculation was conducted in JMP (v15, JMP, Lane Cove, Australia), using the mean and standard deviation of previous sphingolipid analysis in control post-mortem brain (*unpublished*). Using an alpha of 0.05, the priori determined 13 per group sufficient to detect at least a 30% difference between groups with 80% power. Previous post-mortem studies of human Huntington’s brain have used between 3^43,58^ and 14^59^ subjects per group for lipid analysis. Outliers were identified using a 2.2 interquartile range rule from the mean of each respective lipid/enzyme total concentration. Data were assessed for normality using a D’Agostino Pearson Omnibus test. Testing for relevant data is indicated in figure captions. The significance level was set at *P* < 0.01. For testing between Huntington’s disease and control, data were assessed using an unpaired, two-tailed *t*-test with Welch’s correction or a Mann–Whitney U-test where appropriate. Brown Forsythe and Welch’s ANOVA tests were used to compare control regions and were adjusted for multiple comparisons using a two-stage linear step-up Benjamini, Krieger and Yekutieli method. *T*-test and Mann–Whitney U-tests were tested for multiple comparisons using a Benjamini, Krieger and Yekutieli false discovery rate of 1%; however, 30 of the original 34 significant tests did not survive these corrections. As the purpose of this study was exploratory in nature, the authors have reported the data using the unadjusted *P*-values in the manuscript but have included the adjusted *P*-values (*q*) in the supplementary material for the readers information. All means, standard error of the mean, test statistics and exact *P*-values are available in relevant Supplementary Tables 4–28 and are indicated in the Results section. Processed lipid and protein values for individual subjects are available in Supplementary Excel Data file. Correlations were conducted using a Pearson’s correlation where all data were normally distributed and a Spearman’s correlation where not all data were normally distributed. Correlations are reported with their respective *r*, *r*^2^ and *P*-values and the 95% confidence intervals. All statistical tests were conducted using GraphPad Prism (v8, MA, USA) and SPSS (v25, USA).

### Data availability

Processed lipid and protein values and large correlation tables are available in Supplementary Excel Data file. Full western blots have been provided in Supplementary material PDF.

## Results

### Sphingolipid class totals

To determine if total lipid abundances differed between control regions, a Brown Forsythe and Welch’s ANOVA were performed for each subclass of lipid [Cer, SM, HexCer, Hex2Cer, SHexCer, hydroxylated sulphatide (*OH*-SHexCer)] (Supplementary Fig. 3 and Tables 4–9). For all lipid subclasses, the white cortex had significantly higher sphingolipid abundances than all grey matter regions, including the grey cortex (*P* ≤ 0.0001; *q* < 0.0001). Typically, concentrations of sphingolipid classes were comparable across grey matter regions, except for the putamen, which had higher concentrations of SM (4558 ± 296 versus 3069 ± 265 pmol/mg tissue, *P* = 0.0001, *q* ≤ 0.0001) than the cerebellum. Additionally, the grey cortex had higher abundances of SM compared to the caudate (7685 ± 945 versus 3642 ± 162 pmol/mg tissue, *P* = 0.0001, *q* ≤ 0.0001) (Supplementary Fig. 3B). The caudate and putamen had the same concentrations of all lipid subclasses, whilst the cerebellum was the region with the lowest lipid abundances.

Total abundances of each lipid class were then compared with Huntington’s disease subjects. The only difference detected between the two groups was a higher abundance of Hex2Cer in the cerebellum of Huntington’s disease subjects (+65%, *P* = 0.0090) (Supplementary Fig. 3).

### Sphingolipid species

In total, between the five regions, we identified 65 Cer, SM, HexCer, Hex2Cer and SHexCer species. The cortex had a more limited variety of Cer and SM species, containing 11 fewer species than the striatum and cerebellum. To determine the influence of acylation on each of the sphingolipid classes, we assessed region-specific changes in sphingolipid species between Huntington’s disease and control patients. Of the species identified in Huntington’s patients, 18 were significantly altered in the caudate (28%), 9 in the putamen (14%), 4 in the cerebellum (1%) and none were altered in either the white or grey cortex.

#### Fatty acyl chain length alterations in Huntington’s caudate

**Figure 2:**
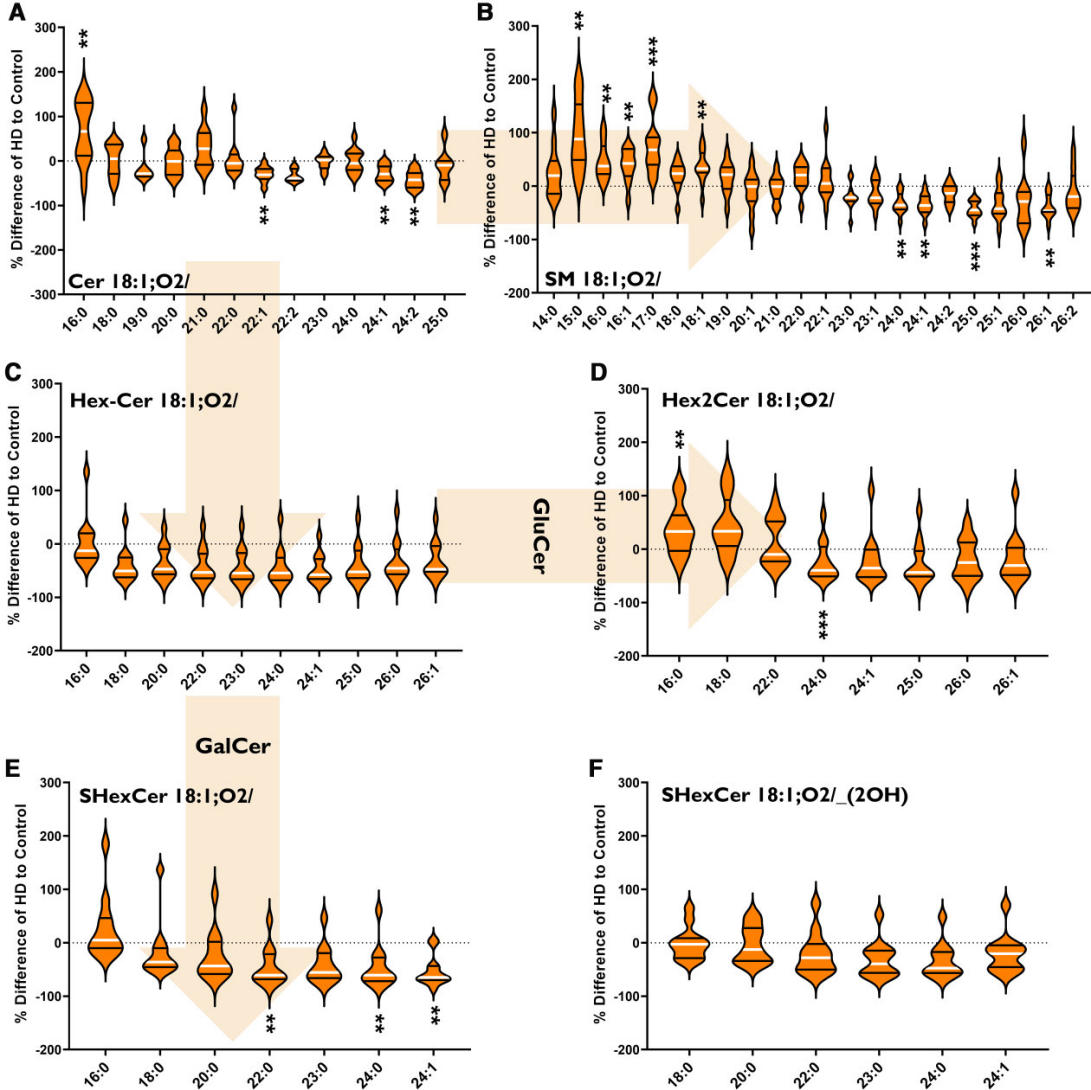
**Percentage differences of (A) Cer, (B) SM, (C) HexCer (GluCer, GalCer), (D) Hex2Cer (lactosylceramide), (E) SHexCer and (F) *OH*-SHexCer species in the caudate of HD subjects (*n* = 12) compared to controls (*n* = 12).** The labels below each violin refer to the fatty acyl chains attached to each lipid class (in bold). Arrows indicate metabolic pathways. Percentage differences were calculated as {[(HD–Control)/(Control)] × 100}. Data were assessed for normality using a D’Agostino Pearson Omnibus test and analysed using an unpaired *t*-test with Welch’s correction or Mann–Whitney U-test where appropriate. The violin plots show the distribution of values, medians (white line) and quartiles (first and third). Accepted significance was set at *P* < 0.01. **P < 0.01, ***P < 0.001. Statistical test details, including exact *P*-values, can be found in Supplementary Tables 10–14. Cer, ceramide; HD, Huntington’s disease; GalCer, galactosylceramide; GluCer, glucosylceramide; SM, sphingomyelin.

In the caudate, Huntington’s disease subjects had an increased abundance of long-chain sphingolipid species (C13–C21) alongside a decreased abundance of very-long-chain species (C22–C26) (Fig. 2). This shift occurred predominately in SM (Fig. 2B) and Hex2Cer (Fig. 2D). Although also occurring in Cer, we did not detect it in as many species (Fig. 2A). Long-chain species of SHexCer, which are derived from galactosylceramide, were decreased in Huntington’s caudate by 40–50%: SHexCer 18:1;O2/22:0 (−46%, *P* = 0.0095), SHexCer 18:1;O2/24:0 (−47%, *P* = 0.0068) and SHexCer 18:1;O2/24:1 (−53%, *P* = 0.0057) (Fig. 2E). However, Hex2Cer, derived from glucosylceramide had the same alteration in sphingolipid chain length as SM and Cer (Fig. 2D). Overall, in Huntington’s caudate, sphingolipids with C16:0 chains were increased in Cer (+66%, *P* = 0.0095), SM (+46%, *P* = 0.0010) and Hex2Cer (+36%, *P* = 0.0058), whilst those with C24:1 were decreased in Cer (−29%, *P* = 0.0059), SM (−35%, *P* = 0.0030), Hex2Cer (−21%, *P* = 0.0001) and SHexCer (−53%, *P* = 0.0057). Due to the changes in sphingolipid fatty acyl chain length, CerS specific for these chain lengths was investigated in the striatum. Lipid data from the caudate can be found in Supplementary Tables 10–14.

#### Increased SM species in Huntington’s putamen

**Figure 3:**
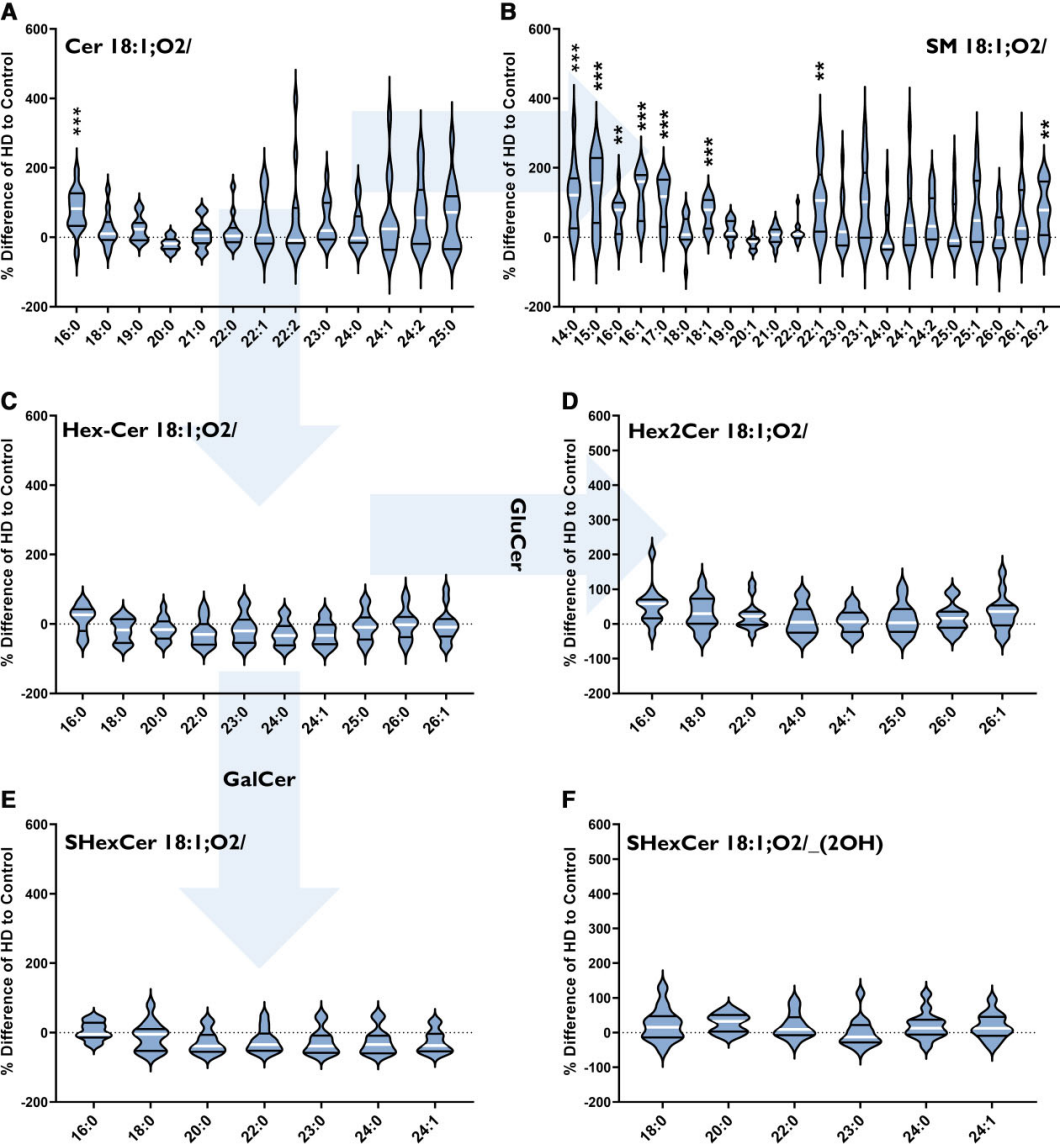
**Percentage differences of (A) Cer, (B) SM, (C) HexCer (GluCer, GalCer), (D) Hex2Cer (lactosylceramide), (E) SHexCer and (F) *OH*-SHexCer species in the putamen of HD subjects (*n* = 13) compared to controls (*n* = 12)**. The labels below each violin refer to the fatty acyl chains attached to each lipid class (in bold). Arrows indicate metabolic pathways. Percentage differences were calculated as {[(HD–Control)/(Control)]** **× 100}. Data were assessed for normality using a D’Agostino Pearson Omnibus test and analysed using an unpaired *t*-test with Welch’s correction or Mann–Whitney U-test where appropriate. The violin plots show the distribution of values, medians (white line) and quartiles (first and third). Accepted significance was set at *P* < 0.01. **P < 0.01, ***P < 0.001. Statistical test details, including exact *P*-values, can be found in Supplementary Tables 15–19. Cer, ceramide; HD, Huntington’s disease; GalCer, galactosylceramide; GluCer, glucosylceramide; SM, sphingomyelin.

One species of Cer was found to be different in Huntington’s putamen: Cer 18:1;O2/16:0 (+28%, *P* = 0.0006) (Fig. 3A). Of the 22 SM species detected in the putamen, eight had an increased concentration in Huntington’s disease subjects. These species were increased by between 50 and 100% compared to controls and included both long and very-long-chain species: SM 18:1;O2/14:0 (+62%, *P* = 0.0008), SM 18:1;O2/15:0 (+135%, *P* = 0.0005), SM 18:1;O2/16:0 (+52%, *P* = 0.0019), SM 18:1;O2/16:1 (+122%, *P* = 0.0000), SM 18:1;O2/17:0 (+91%, *P* = 0.0002), SM 18:1;O2/18:1 (+128%, *P* = 0.0000), SM 18:1;O2/22:1 (+119%, *P* = 0.0044) and SM 18:1;O2/26:2 (+79%, *P* = 0.0077) (Fig. 3B). No differences in HexCer, Hex2Cer or SHexCer species were detected (Fig. 3C–F). Lipid data from the putamen can be found in Supplementary Tables 15–19.

#### Increased Hex2Cer species in Huntington’s cerebellum

**Figure 4:**
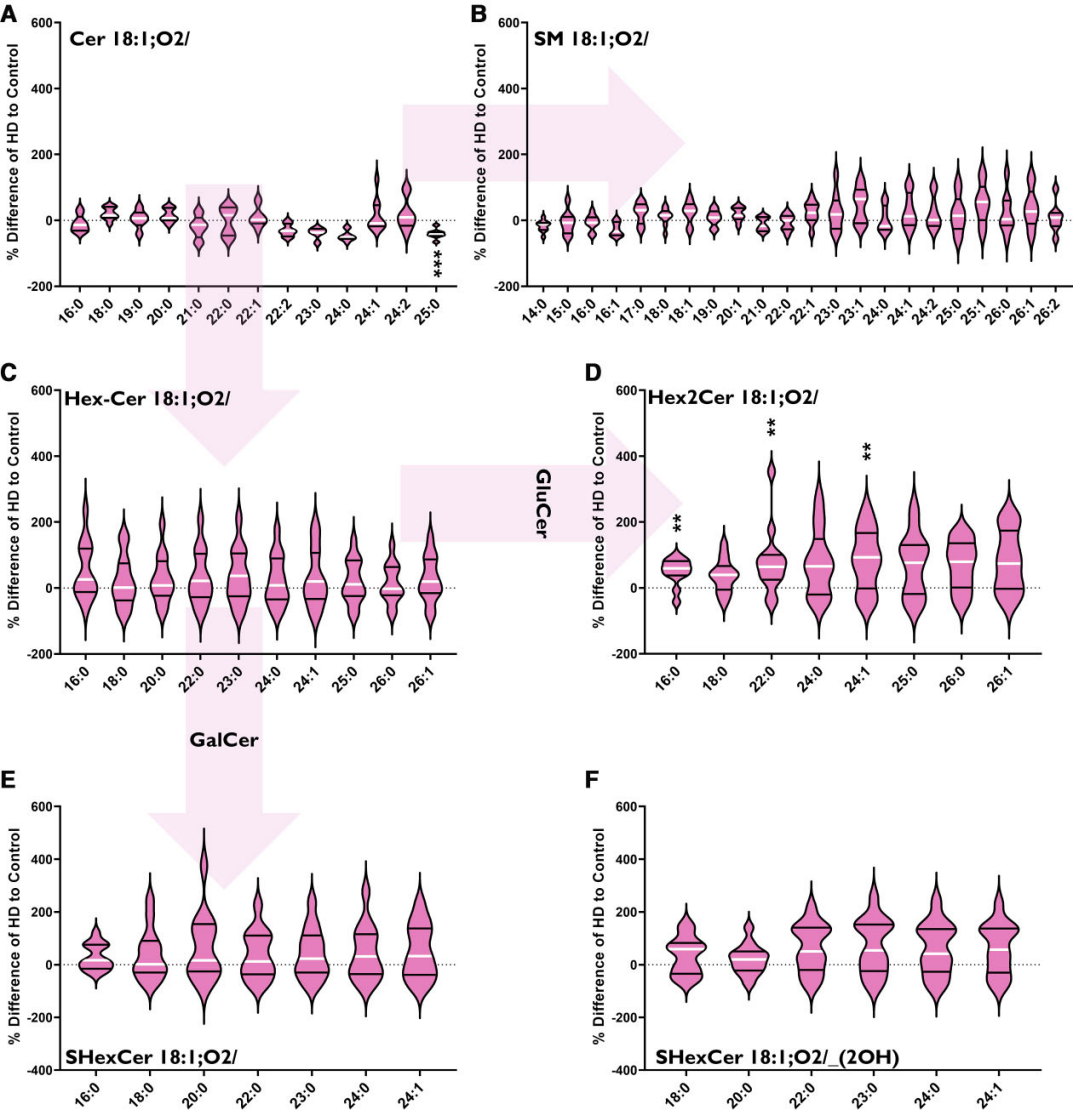
**Percentage differences of (A) Cer, (B) SM, (C) HexCer (GluCer, GalCer), (D) Hex2Cer (lactosylceramide), (E) SHexCer and (F) *OH*-SHexCer species in the cerebellum of HD subjects (*n* = 13) compred to controls (*n* = 13)**. The labels below each violin refer to the fatty acyl chains attached to each lipid class (in bold). Arrows indicate metabolic pathways. Percentage differences were calculated as {[(HD–Control)/(Control)] × 100}. Data were assessed for normality using a D’Agostino Pearson Omnibus test and analysed using an unpaired *t*-test with Welch’s correction or Mann–Whitney U-test where appropriate. The violin plots show the distribution of values, medians (white line) and quartiles (first and third). Accepted significance was set at *P* < 0.01. **P < 0.01, ***P < 0.001. Statistical test details, including exact *P*-values, can be found in Supplementary Tables 20–24. Cer, ceramide; HD, Huntington’s disease; GalCer, galactosylceramide; GluCer, glucosylceramide; SM, sphingomyelin.

Huntington’s disease patients had an increased abundance of Hex2Cer (Fig. 4D) in the cerebellum driven by increases in three Hex2Cer species. The largest increase was in Hex2Cer 18:1;O2/16:0 (+145%, *P* = 0.0020), whilst the remaining species had increases of 78% (Hex2Cer 18:1;O2/22:0, *P* = 0.0057) and 85% (Hex2Cer 18:1;O2/24:1, *P* = 0.0058). Cer, SM, HexCer, SHexCer and OH-SHexCer species showed no remarkable differences in Huntington’s disease subjects (Fig. 4A–C, E, F). Lipid data from the cerebellum can be found in Supplementary Tables 20–24.

#### The preservation of sphingolipid homeostasis in the dorsomedial prefrontal cortex of Huntington’s patients

No differences in any sphingolipid species between Huntington’s disease patients and controls were detected in either the white (Supplementary Fig. 4 and Tables 25–29) or grey (Supplementary Fig. 5 and Tables 30–34) matter of the dorsomedial prefrontal cortex.

#### CAG repeat length and sphingolipid concentrations

*In vitro* models suggest an influence of polyQ (CAG repeat) length on interference with lipid bilayers.^60,61^ Pearson’s correlations were used to investigate relationships between the concentration of sphingolipid species and the CAG repeat length of Huntington’s disease subjects in each brain region. No relationships were identified.

### Ceramide synthases

To determine if the chain length alterations in sphingolipid species were a result of changes to CerS, we performed western blot analyses of two isoforms. CerS1 and CerS2 were chosen due to their specificity for the sphingolipid acyl chain lengths affected (CerS1 C18; CerS2 C22–C26), the high abundance of these sphingolipid species in our analysis and the high mRNA expression of these isoforms in mouse brain.^33,62^ Before adjusting for CerS expression, the expression of housekeeping proteins was assessed to see if there were any significant differences between Huntington’s subjects and controls. In the putamen, we found a significant difference in the expression of β-actin (−14 to −44% in Huntington’s disease), so this housekeeper was not used for our analysis of the putamen (Supplementary Table 35). Full western blot images are available in Supplementary Figs 5–8.

#### Ceramide synthase 1

**Figure 5:**
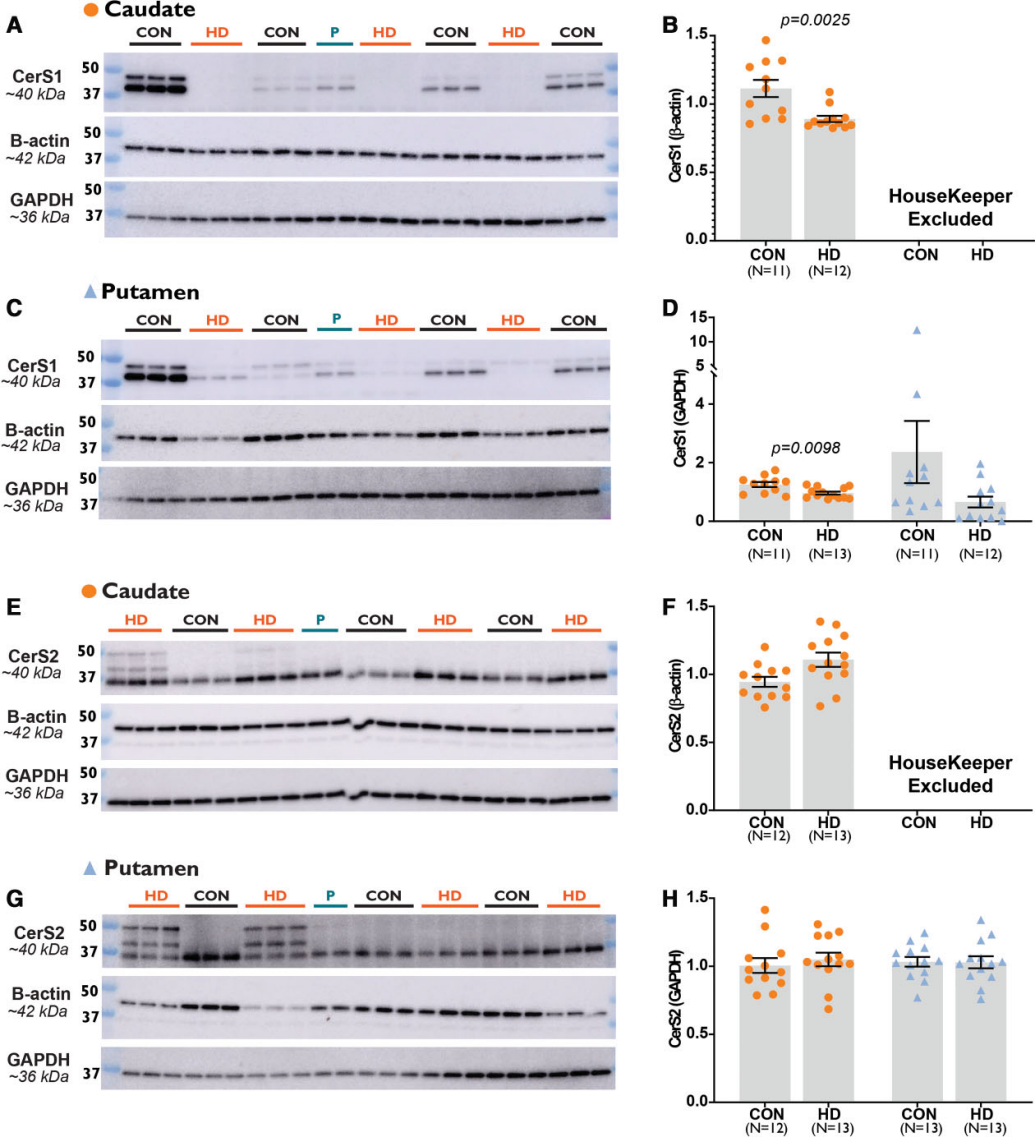
**The expression of CerS1 and CerS2 in Control and HD striatum.** The mean expression of CerS1 (∼40 kDa) between control and HD subjects adjusted by (**B**) β-actin and (**D**) GAPDH expression in striatal subregions (orange circles for caudate; blue triangles for putamen). The mean expression of CerS2 (∼40 kDa) between control and HD subjects adjusted by (**F**) β-actin and (**H**) GAPDH expression in the same regions. Graphs display the mean, standard error of the mean and individual subject values. Representative western blots of CerS1 and CerS2 in the (**A** and **E**) caudate and (**C** and **G**) putamen with β-actin and GAPDH housekeepers. β-Actin was excluded as a housekeeper for putamen samples as the expression was significantly different in HD subjects compared with controls. Samples were combined to create the ‘pool’ and standardize measurements between membranes. Data were assessed for normality using a D’Agostino Pearson Omnibus test and then analysed with an unpaired *t*-test with Welch’s correction or Mann–Whitney U-test. Accepted significance was set at *P* < 0.01. Full western blots are provided in Supplementary Figs 7–10. Statistical test information is available in Supplementary Tables 36 and 37. CerS1, ceramide synthase 1; CerS2, ceramide synthase 2; CON, control; GAPDH, glyceraldehyde 3-phosphate dehydrogenase; HD, Huntington’s disease; P, pool.

The expression of the CerS1 primary band (∼40 kDa) was lower in Huntington’s disease caudate compared to controls when adjusted for both β-actin (−57.60%, *P* = 0.0025) (Fig. 5B) and GAPDH (−23.34%, *P* = 0.0098) (Fig. 5D). No differences were detected for CerS1 primary band in the putamen of Huntington’s disease subjects. Analysis of the secondary band observed at ∼45 kDa also revealed no differences between Huntington’s and control subjects in either the caudate or putamen.

**Figure 6:**
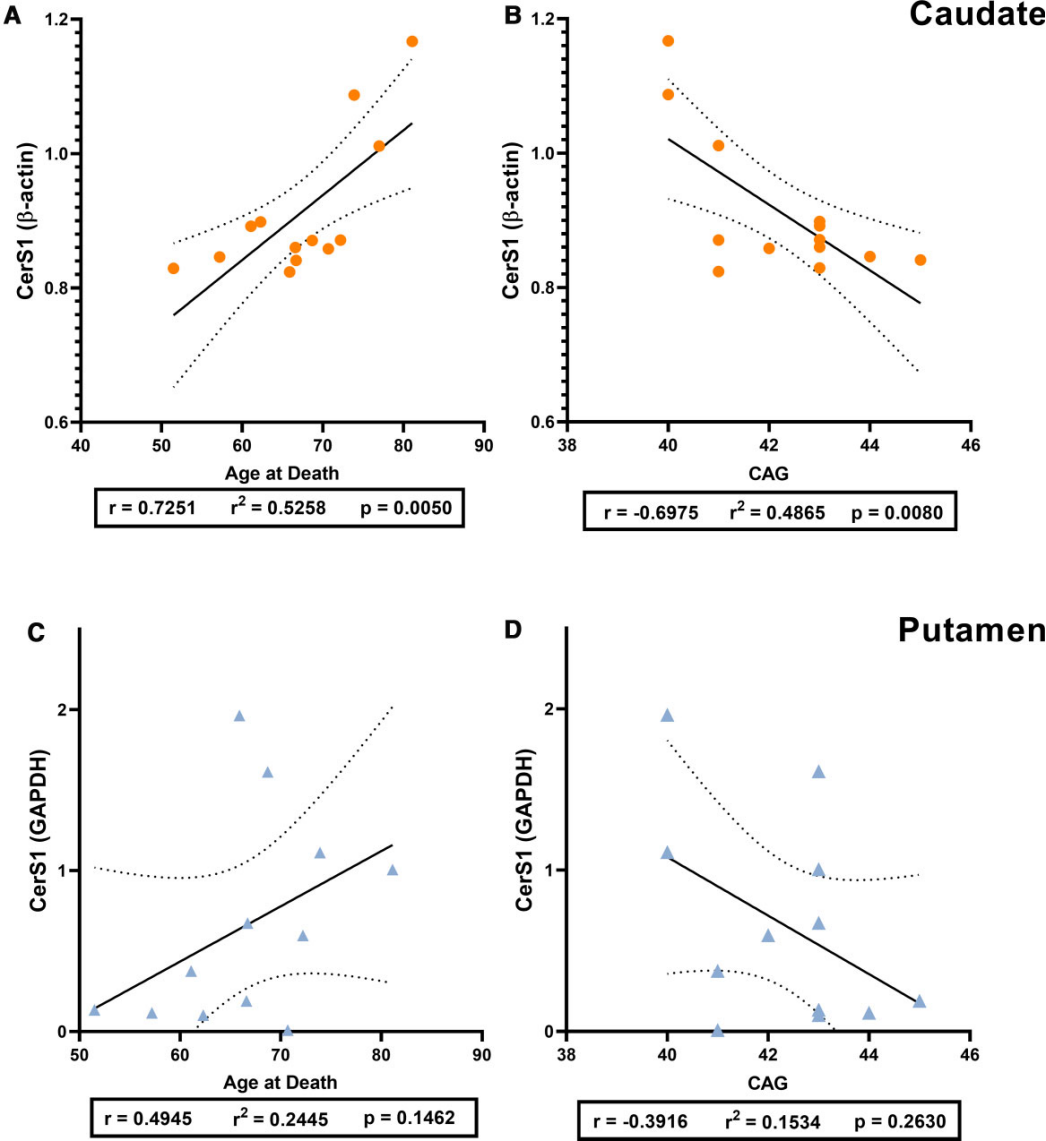
**The relationship of CerS1 expression with age at death and CAG repeat length in HD.** Relative expression of CerS1 (primary band, ∼40 kDa) to housekeeper expression is used. Pearson’s correlation analyses were used to determine correlations. Plots indicate the line of best fit (solid black line) with 95% confidence intervals (dotted black lines). CerS1 versus age at death in (**A**) HD caudate (*n* = 13) and (**C**) HD putamen (*n* = 12). CerS1 versus CAG repeat length in (**B**) HD caudate and (**D**) HD putamen. Correlation analysis values are provided in Supplementary Tables 30 and 31. CerS1, ceramide synthase 1; HD, Huntington’s disease.

The age at death and CAG repeat number are reported to be strongly correlated and we confirmed this by running Pearson’s correlation analysis using our data (*r* = −0.7443, *P* = 0.0086) (Supplementary Fig. 10). Pearson’s correlation analyses were used to examine the relationship between CerS1 expression (primary band; ∼40 kDa) and age at death or CAG repeat length in Huntington’s disease subjects (Fig. 6). A strong positive correlation between CerS1 expression and age at death (*r* = 0.7251, *P* = 0.0050; Fig. 6A) was identified in Huntington’s caudate, alongside a strong negative correlation between CerS1 expression and CAG repeat length (*r* = −0.6975, *P* = 0.0080; Fig. 6B). No relationship between CerS1 was identified with age at death (*r* = 0.4945, *P* = 0.1462; Fig. 6C) or CAG repeat length (*r* = −0.3916, *P* = 0.2630; Fig. 6D) in Huntington’s putamen. No relationship between CerS1 expression and age at death was found in controls for either caudate (*r* = 0.4873, *P* = 0.1081) or putamen (*r* = 0.5401, *P* = 0.0864) (Supplementary Tables 38 and 39).

Due to the specificity of CerS1 for C18 fatty acyl chains, Spearman’s correlation analyses were conducted to determine if a relationship existed between the expression of CerS1 (by β-actin) and sphingolipids with C18:0 fatty acyl chains. No correlations were found for any lipid type with CerS1 in control and Huntington’s disease subjects (Supplementary Excel File Table 7).

#### Ceramide synthase 2

**Figure 7:**
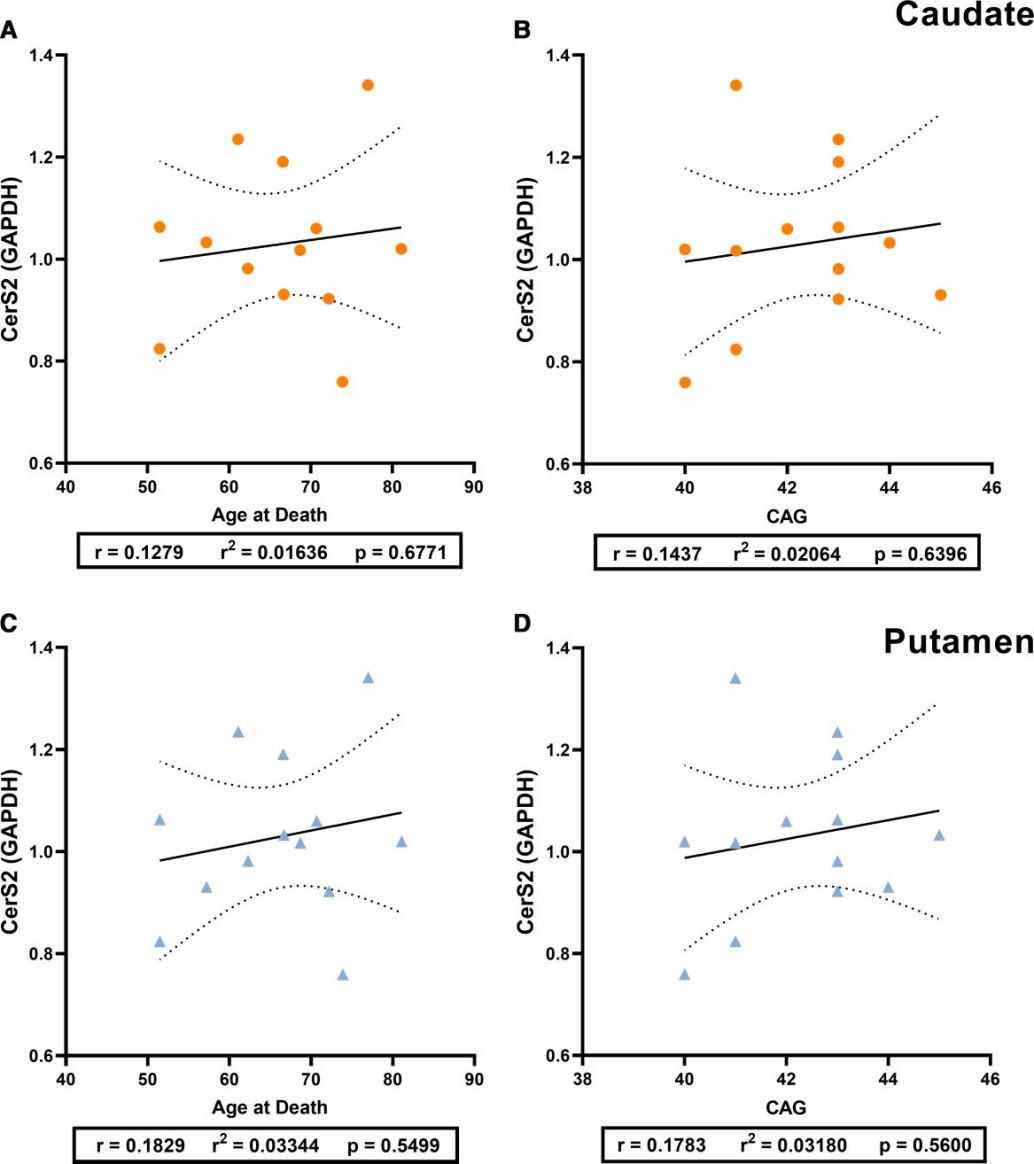
**The relationship of CerS2 expression with age at death and CAG repeat length in HD.** Relative expression of CerS2 (primary band, ∼40 kDa) to housekeeper expression is used. Pearson’s correlation analyses were used to determine correlations. Plots indicate the line of best fit (solid black line) with 95% confidence intervals (dotted black lines). CerS2 versus age at death in (**A**) HD caudate (*n* = 13) and (**C**) HD putamen (*n* = 13). CerS2 versus CAG repeat length in (**B**) HD caudate and (**D**) HD putamen. Correlation analysis values are provided in Supplementary Tables 30–32. CerS2, ceramide synthase 2; HD, Huntington’s disease.

No differences in the expression of the CerS2 primary band (∼40 kDa) between control and Huntington’s disease samples were identified in either the caudate or the putamen (Fig. 5F and H). CerS2 expression (∼40 kDa) was not correlated with age at death or CAG repeat length in Huntington’s disease subjects in either the caudate (Fig. 7A and B) or putamen (Fig. 7C and D). No correlations were identified between CerS2 expression (∼40 kDa) and age at death for controls (Supplementary Tables 38 and 39).

Additional banding of CerS2 was identified in 4 of the 13 Huntington’s disease subjects. No discernible underlying factor, including sex, age at death, post-mortem interval, brain pH or CAG repeat length, was associated with the expression of additional bands (Supplementary Table 40). A CerS2 knockout cell lysate was used as a negative control and wild-type cell lysate as a positive control to assess antibody specificity for the CerS2 protein. We confirmed the specificity of the antibody and the absence of disulphide bonding as a cause of the additional banding (Supplementary Method 1 and Fig. 1).

Due to the specificity of CerS2 for C22–C24 fatty acyl chains, Spearman’s correlations were used to determine relationships for both Huntington’s disease and control subjects. No correlations between CerS2 expression (by GAPDH) and C22–C24 fatty acyl chains for any sphingolipid were found (Supplementary Excel File Table 7).

## Discussion

A key finding of this investigation is the shift in fatty acyl chain length of sphingolipids in the caudate of clinically advanced Huntington’s disease patients. We were able to identify increased abundances of long-chain species and decreased abundances of very-long-chain species of Cer, SM and LacCer. We cannot exclude the possibility that these shifts are also occurring in glucosylceramide due to the significantly higher abundance of galactosylceramide in the brain^63^ and the inability of the applied mass spectrometric techniques to distinguish between galactosylceramide and glucosylceramide. Shifts in the ratio of long and very-long-chain sphingolipids in the brain indicate ‘immature myelin’^64^ and occur in adrenoleukodystrophy,^45,65,66^ frontotemporal dementia with Pick’s disease,^67^ multiple sclerosis^68^ and Parkinson’s disease.^37^ Parkinson’s disease and Huntington’s disease share a significant disturbance of the basal ganglia and dopaminergic pathways.^69,70^ In Parkinson’s disease, Cer 18:1;O2/18:0 and SM 18:1;O2/18:1 are increased, whilst Cer 18:1;O2/24:1, SM 18:1;O2/23:0, SM 18:1;O2/24:1 and SM 18:1;O2/26:1 are decreased in the grey matter of the anterior cingulate cortex.^37^ These disturbances do not occur in the white matter of the anterior cingulate cortex or the occipital cortex, showing a region-specific shift in the abundance of sphingolipid species by fatty acyl chain length, in an area significantly affected in Parkinson’s disease.^37^ The increased long chain and decreased very-long-chain sphingolipids identified in Huntington’s caudate were also region-specific and occurred in the most significantly affected brain region in Huntington’s disease.^9,47^ The alterations in the anterior cingulate cortex of Parkinson’s disease and now in the caudate of Huntington’s disease indicate that disturbances to sphingolipids in neurological disorders may not solely correlate with myelin disturbances. Our analysis did not find these shifts in the white matter of the frontal cortex, a region that is also severely affected in Huntington’s disease and is heavily myelinated.^71^ These increases in the abundance of long over very-long-chain sphingolipids may reflect changes to the lipid profile of neuronal membranes and/or synapses. Altering the lipid profile of neuronal membranes can have profound consequences for the fluidity and permeability of the membrane.^72^ These changes can influence the organization of the lipid membrane, including the placement of integral proteins, affecting numerous neuronal capabilities such as receptor binding.^24^ Since our lipid extractions are from whole brain tissue, it is difficult to determine whether these changes are due specifically to defects in axons, synapses or myelin.

The increases in long-chain Cer (16:0) in Huntington’s caudate may promote apoptosis in cells. Increases in long-chain Cer, specifically 16:0 species, are associated with dysregulated apoptosis.^73^ In immortalized human cervical cancer cells (HeLa cells), increased expression of CerS2 (C22–C24) has protective effects against apoptosis, whilst an increased expression of CerS5 (C16) promotes apoptosis.^74^ These cellular studies highlight the possible implications of *any* modifications to CerS2 in Huntington’s caudate. Possible modifications to CerS2 could interfere with the synthesis of very-long-chain Cer, affecting the downstream availability to lactosylceramide and SM. Disruptions in the balance of sphingolipid species could also contribute to the apoptotic susceptibility of cells. The use of CerS2 knockout cell lysates supported that the additional bands in the striatal western blots reported are related to the CerS2 protein (Supplementary Fig. 1). CerS2 can be regulated post-transcriptionally by phosphorylation^75^ or glycosylation^76^; however, the specific modifications to CerS2 indicated in the western blots have not been determined. CerS2 knockout mice have defective myelin sheaths and reduced very-long-chain Cer (24:0, 24:1) with a compensatory increase in long-chain Cer (C16, C18).^31^ CerS2 knockout human HeLa cell lines observed the same effect of increased C16 Cers and decreased 24:0 and 24:1 Cer, which increased the susceptibility of the cells to apoptosis.^77^ These results suggest when CerS2 activity is disturbed, long-chain Cer are synthesized in compensation. The balance of long and very-long-chain sphingolipid species could contribute to the susceptibility of a cell to apoptosis by influencing channel formation in the mitochondrial membrane and therefore, permeability to pro-apoptotic molecules.^74^ Mitochondrial dysfunction has been documented extensively in Huntington’s disease brain as well as rodent and cell models.^78–82^

Huntington’s disease subjects had a reduced expression of CerS1 localized to the caudate. The expression of CerS1 in the caudate was related to the age at death and the CAG repeat length of Huntington’s patients. CAG repeat length is inversely correlated with age of onset and age of death in Huntington’s disease. In our study, Huntington’s patients with longer CAG repeats and who died at an earlier age had lower CerS1 expression in the caudate. It is unclear as to why this relationship exists in Huntington’s disease. However, CerS1 has a high expression in neurons^35^ and so lower concentrations of this enzyme may simply reflect a greater degree of neuronal cell loss brought on by a longer CAG repeat and more severe clinical symptoms.^2,83^ The expression of neither CerS1 nor CerS2 correlated consistently with the concentrations of their respective sphingolipid species. CerS expression (mRNA) does not correlate directly with sphingolipid concentrations *in vivo*.^31,37,84^ The multiple pathways that feed into the production and recycling of Cer, as well as possible post-translational modifications and the overlapping specificity of CerS, mean that the expression of these enzymes cannot solely explain these sphingolipid changes. In addition, the expression of CerS proteins does not necessarily reflect the activity of these enzymes, which could influence sphingolipid concentrations. An increased expression of other relevant CerS isoforms (CerS4, CerS5, CerS6) may have also contributed.

The putamen and cerebellum had distinctive increases in sphingolipid species in Huntington’s disease patients. In Huntington’s, whilst the putamen shared increases in very-long-chain SM with the caudate, it had additional increases in long-chain SM species. As the synthesis of SM typically occurs in oligodendrocytes, the increases in SM species in the putamen may be the result of these cells.^85,86^ The cerebellum, which has a Purkinje cell dominant population, had elevations in several LacCer species. This region suffers varying degrees of atrophy and lipid disturbance in Huntington’s disease, and alterations to this region are typically more severe in juvenile cases. LacCer is an important mediator of astrogliosis (increase in astrocytes) and inflammation.^87^ In other pathologies such as inflammatory bowel diseases and emphysema, elevations occur alongside inflammation, apoptosis and autophagy.^88,89^ Astrogliosis occurs in multiple brain regions in Huntington’s disease, including the cerebellum.^90^

In this study, both the white and grey matter of a specific cortical functional region were used for comparative analysis. The dorsomedial prefrontal cortex is associated with social cognition,^91^ which is impaired early in Huntington’s disease.^3^ The cerebral cortex, although not as severely affected as the striatum in Huntington’s, still experiences mass losses of ∼30%, the frontal cortex being a prominent location of this loss.^9,71,92^ Therefore, the absence of alterations to sphingolipids in the cerebral cortex was an unanticipated occurrence. Decreases in several sphingolipid precursors have been identified in R6/2 transgenic mouse models,^19^ and there is limited information available in human post-mortem tissue. Our findings reflect only a small portion of the cerebral cortex, so we cannot rule out alterations to sphingolipid metabolism in other cortical regions (e.g. parietal, occipital). Previous analysis has identified that the overall lipid profile of the frontal cortex is relatively preserved in ageing,^93,94^ and cortical regions that are more proximal to the striatum (corpus callosum) typically degenerate earliest in Huntington’s disease.^92^ The cortical neurons of the dorsomedial prefrontal cortex may resist disturbances to sphingolipid homeostasis in Huntington’s, leaving it relatively preserved. Since rates of atrophy differ between the white and grey matter of the frontal cortex,^95–97^ it was expected that these regions would show unique differences in sphingolipid metabolism; however, this was not the case. The lipid concentrations of the grey matter in Huntington’s subjects were extremely variable (Supplementary Fig. 5), making statistical analysis challenging. Significant variations in the disturbance of cortical regions are an element of Huntington’s disease pathology.^98^

It should be acknowledged that the results presented are attributable to end-stage Huntington’s disease and cannot reflect the dynamic nature of sphingolipid metabolism at earlier stages. In addition, it should also be noted that these findings are applicable specifically to Vonsattel grade IV pathologies. Post-mortem analysis of Huntington’s disease brain is limited to end-stage, except in cases where an alternative cause of death is apparent (e.g. suicide). Transgenic mouse models are a valuable tool for examining the changes to sphingolipid metabolism at earlier disease stages and in tandem with disease progression. There is limited information on the changes to the lipids studied in this research in these models; however, the findings generally align with the current data in regard to the lipid species and proteins which are disturbed in Huntington’s disease. In R6/2 transgenic mice, very-long-chain Cer are increased in the striatum (Cer 24:0) and cortex (Cer 20:0, Cer 22:0, Cer 24:0, Cer 24:1).^99^ In the same models, the mRNA expression of CerS1 is lower in the striatum and cortex.^19^ In R6/1 mouse models, cerebrosides (HexCer) and sulphatides^22^ are decreased in the forebrain, and sulphatides decreased in cerebellum.^58^ Identified alterations in gangliosides in transgenic mouse models are difficult to compare against the results from this study, as individual species are often not reported.^22,41,58^ Future post-mortem studies would benefit from using larger sample sizes with the intention of examining possible gender effects on brain chemistry in Huntington’s disease. Due to limited statistical power (*n* = 8 males, *n* = 5 females per group), the influence of gender could not be determined.

Previous studies using *in vitro* models have shown polyQ length-dependent effects on the interaction of mHTT in lipid bilayers.^60^ Longer polyQ is reported to cause more significant disturbance and disorganization of these bilayers.^60^ Therefore, the relationship between the CAG repeat length of mutant huntingtin protein (*mHTT*) and the abundance of lipids and related proteins (CerS) was explored. As mentioned previously, CAG repeat length and age at death were related to CerS1 expression in the caudate of Huntington’s patients. However, no relationships were identified between CAG repeat length and any lipid abundance in any brain region of Huntington’s patients. Previous analysis of plasma from Huntington’s patients has also found CAG repeat length not to correlate with the abundance of lipid metabolites, instead, correlating with measures of functional capacity.^100^ It may be that mHTT interferes with lipids *in vivo* through interactions with lipid enzymes and not necessarily the lipids directly.

Changes to the neural sphingolipid composition in people with Huntington’s disease are determinant on the brain region. Although the striatum is traditionally considered a single region, the caudate and putamen have distinctive shifts to their sphingolipid profiles, despite sharing the same neural cell populations. For the first time, an increased abundance of long chain and a corresponding decreased abundance of very-long-chain Cer, SM and lactosylceramides has been identified in the caudate of clinically advanced Huntington’s patients. The possibility of post-translational modifications of CerS2 driving these changes cannot be excluded, as these may contribute to the striatum’s vulnerability to sphingolipid, and possibly mitochondrial dysfunction in Huntington’s disease. The unique neuronal cell populations of the cortex, striatum and cerebellum distinguish them from one another and this may contribute to their susceptibility to sphingolipid disturbance. Future studies are required to determine whether the detected changes in sphingolipids are casual or secondary to a pathology including neuronal loss or mitochondrial composition changes. However, the shifted sphingolipid profile of the caudate may provide clues as to how regional neural cell dysfunction occurs not only in Huntington’s disease but also in related neurodegenerative diseases.

## Supplementary Material

fcab303_Supplementary_Data

## Abbreviations

BHT =: butylated hydroxy-toluene
Cer =: ceramide
CerS =: ceramide synthase
ESI =: electrospray ionization
GAPDH =: glyceraldehyde 3-phosphate dehydrogenase
HD =: Huntington’s disease
HexCer =: hexosylceramide
Hex2Cer =: dihexosylceramide
HPLC =: high-performance liquid chromatography
HTT =: wild-type Huntingtin gene
*HTT* =: wild-type Huntingtin protein
LacCer =: lactosylceramide
LC =: liquid chromatography
LC-MS =: liquid chromatography-mass spectrometry
MS =: mass spectrometry
mHTT =: mutant huntingtin gene
*mHTT* =: mutant huntingtin protein
*OH*-SHexCer =: hydroxylated sulphatide
SHexCer =: sulphatide
SM =: sphingomyelin
TBST =: tris-buffered saline with Tween 20
