# Supplementary material for: The long and the short of Huntington’s disease: how the sphingolipid profile is shifted in the caudate of advanced clinical cases
Source: Brain Commun. 2021 Dec 23;4(1):fcab303. doi: 10.1093/braincomms/fcab303 (PMC8833324; doi:10.1093/braincomms/fcab303)
Supplement: fcab303_Supplementary_Data [file fcab303_supplementary_data.zip › Original Submission.pdf]

OXFORD UNIVERSITY PRESS | Brain Communications

The long and the short of Huntington's disease: how the sphingolipid profile is shifted in the caudate of advanced clinical cases

Journal:	*Brain Communications*
Manuscript ID	BRAINCOM-2021-206
Manuscript Type:	Original Article
Date Submitted by the Author:	14-Jun-2021
Complete List of Authors:	Phillips, Gabrielle; University of Wollongong Illawarra Health and Medical Research Institute; University of Wollongong, School of Medicine; University of Wollongong, Molecular Horizons<br>Saville, Jennifer; Women's and Children's Hospital Adelaide, Genetics and Molecular Pathology, SA Pathology<br>Hancock, Sarah; University of New South Wales, School of Medical Sciences<br>Brown, Simon; University of Wollongong, Molecular Horizons; University of Wollongong, School of Chemistry and Molecular Biosciences<br>Jenner, Andrew; University of New South Wales, Bioanalytical Mass Spectrometry Facility, Mark Wainright Analytical Centre<br>McLean, Catriona; Alfred Hospital, Anatomical Pathology<br>Fuller, Maria; Women's and Children's Hospital Adelaide, Genetics and Molecular Pathology, SA Pathology; The University of Adelaide, Adelaide Medical School<br>Newell, Kelly; University of Wollongong Illawarra Health and Medical Research Institute; University of Wollongong, School of Medicine; University of Wollongong, Molecular Horizons<br>Mitchell, Todd; University of Wollongong Illawarra Health and Medical Research Institute; University of Wollongong, School of Medicine; University of Wollongong, Molecular Horizons
Keywords:	sphingolipid, Huntington's disease, cortex, glycosphingolipid, striatum

SCHOLARONE™
Manuscripts

The long and the short of Huntington's disease: how the sphingolipid profile is shifted in the caudate of advanced clinical cases

Gabrielle R. Phillips[1, 2, 3], Jennifer T. Saville[4], Sarah E. Hancock[5], Simon HJ. Brown[3,6], Andrew M. Jenner[7], Catriona McLean[8], Maria Fuller[4,9], Kelly A. Newell[1,2,3], Todd Mitchell[1,2,3*]

Abstract

Huntington's disease is a devastating neurodegenerative disorder that onsets in late adulthood as progressive and terminal cognitive, psychiatric, and motor deficits. The disease is genetic, triggered by a polyQ (CAG) expansion mutation in the Huntingtin gene and resultant huntingtin protein. Although the mutant huntingtin protein is ubiquitously expressed, the striatum is uniquely targeted. The polyQ mutation at the N-terminus of the huntingtin protein alters its natural interactions with neural phospholipids *in vitro*, indicating that the specific lipid composition of brain regions can influence their vulnerability to interference by mutant huntingtin. Sphingolipids are critical cell signalling molecules, second messengers and membrane components. Despite evidence of sphingolipid disturbance in Huntington's mouse and cell models, there is limited knowledge of *how* these lipids are affected in human brain tissue. Using post-mortem brain tissue from five brain regions implicated in Huntington's disease (control n = 13, Huntington's n = 13), this study aimed to identify *where* and *how* sphingolipid species are affected in the brain of clinically advanced Huntington's cases. Sphingolipids were extracted from the tissue and analysed using targeted mass spectrometry analysis; proteins were analysed by western blot. The caudate, putamen and cerebellum had distinct sphingolipid changes in Huntington's brain whilst the white and grey frontal cortex were spared. The caudate of Huntington's patients had a shifted sphingolipid profile, favouring long (C13-C21) over very long chain (C22-C26) ceramides, sphingomyelins and lactosylceramides. Ceramide Synthase 1, which synthesises the long chain sphingolipids, had a reduced expression in Huntington's caudate, correlating positively with a younger age at death and a longer CAG repeat length of the Huntington's patients. The expression of Ceramide Synthase 2, which synthesises very long chain sphingolipids, was not different in Huntington's brain, although there was evidence of possible post-translational modifications in the

For Review Only

Huntington's patients only. Post-translational modifications to Ceramide Synthase 2 may be driving the distinctive sphingolipid profile shifts of the caudate in advanced Huntington's disease. This shift in sphingolipid profile is also found in the most severely affected brain regions of several other neurodegenerative conditions and may be an important feature of region-specific cell dysfunction in neurodegenerative disease.

Author affiliations:

[1]Illawarra Health and Medical Research Institute, Wollongong, 2522, NSW, Australia

[2]School of Medicine, University of Wollongong, Wollongong, 2522, NSW, Australia

[3]Molecular Horizons, University of Wollongong, Wollongong, 2522, NSW, Australia

[4]Genetics and Molecular Pathology, SA Pathology at Women's and Children's Hospital, North Adelaide, 5006, SA, Australia

[5]School of Medical Sciences, University of New South Wales, Sydney, 2052, NSW, Australia

[6]School of Chemistry and Molecular Biosciences, University of Wollongong, Wollongong, 2522, NSW, Australia

[7]Bioanalytical Mass Spectrometry Facility, Mark Wainwright Analytical Centre, University of New South Wales, Sydney, 2052, NSW, Australia

[8]Department of Anatomical Pathology, Alfred Health and Florey Neuroscience, Parkville, 3052, VIC, Australia

[9]Adelaide Medical School, University of Adelaide, Adelaide, 5000, SA, Australia

Email addresses of the authors

Gabrielle Phillips: gp791@uowmail.edu.au

Jennifer Saville: jennifer.saville@adelaide.edu.au

Sarah Hancock: sarah.hancock@unsw.edu.au

Simon Brown: simonb@uow.edu.au

Andrew Jenner: andrew.jenner@unsw.edu.au

Catriona McLean: Catriona.McLean@monash.edu

For Review Only

Maria Fuller: maria.fuller@adelaide.edu.au

Kelly Newell: knewell@uow.edu.au

Todd Mitchell: toddm@uow.edu.au

Correspondence to: Todd W. Mitchell

School of Medicine, University of Wollongong, Northfields Avenue, Wollongong, 2522, NSW, Australia

toddm@uow.edu.au

For Review Only

Running title: Chain length alterations to sphingolipids in Huntington's

Keywords: Huntington's; Glycosphingolipid; Sphingolipid; Mass Spectrometry; Striatum

Abbreviations: CerS = Ceramide Synthase; **HD** = Huntington's disease; **htt** = wild-type huntingtin; **LacCer** = Lactosylceramide; **mhtt** = mutant huntingtin.

Introduction

Huntington's disease is an autosomal, dominant, neurodegenerative disease caused by a mutation in the Huntingtin gene (HTT). This mutation results in a polyglutamine expansion (polyQ) at the N-terminus of the huntingtin protein (htt), referred to as mutant huntingtin (mhtt)[1]. The disease presents as progressive and terminal cognitive, psychiatric, and motor disturbances lasting approximately 15-20 years. Onset is typically in late adulthood; the age of onset influenced by the polyQ length. Longer polyQ mutations are associated with an earlier age of onset, more severe clinical expression and an earlier age of death[2,3]. Huntingtin appears to play an important role in neurodevelopment[4], however, it is still debated as to whether Huntington's disease results from a '*loss of function*' of htt or a pathological '*gain of function*' of mhtt.

Although mutant huntingtin is expressed ubiquitously in the brain, the striatum is uniquely affected and is the most severely degenerated region in Huntington's disease[5,6]. The striatum

includes three smaller subregions: the caudate nucleus, putamen, and nucleus accumbens. It is a component of the basal ganglia and has roles in voluntary movement, cognition, learning and memory[7,8]. The caudate and putamen atrophy differently in Huntington's disease[9,10], and correlate with different disease indices (caudate with the age of onset, putamen with disease severity)[6]. The underlying cause of the striatum's vulnerability to mutant huntingtin is unknown[11], although its dominant cell type, medium spiny neurons, are believed to be a considerable contributing factor. htt associates with phospholipids in neural cell cultures and its preferences and interactions are altered by the polyglutamine mutation on the N-terminus[12,13]. Brain regions have distinct lipid compositions[14], tailored by their unique cell populations, neural connections and functional requirements[15]. These compositions can create vulnerabilities to specific pathological triggers. For example, grey matter regions that are more caudal have a higher polyunsaturated fatty acid content and are therefore more susceptible to lipid oxidation and mitochondrial stress[14]. Region-specific alterations to lipids and their metabolic genes, including sphingolipids, are a feature of the striatum in Huntington's diease[16–19].

Sphingolipids are characterised by their sphingoid base and are involved in many key cellular and neural processes including cell signalling, membrane formation and organisation, inflammation, immune response, myelination, and regulation of neurotransmitters[20–24]. Ceramides are the key building block for complex sphingolipids and lie at the centre of multiple synthesis and recycling pathways (**Figure 1**). In the *de novo* synthesis pathway, ceramide is formed via the acylation of sphinganine by one of six tissue-specific isoforms of ceramide synthase (CerS) (**Figure 1**). Each of these isoforms has an affinity for different fatty acyl chain lengths: CerS1 (C18), CerS2 (C22-C24), CerS3 (C18 & C24), CerS4 (C18-C20), CerS5 and CerS6 (C14-C16)[24–26]. Fatty acids can be grouped according to their carbon chain length: short (≤C5), medium (C6-C12), long (C13-C21) and very long (≥22C). The length of the fatty acyl chain is an important factor contributing to a lipids' biological function and cellular location[27–29]. CerS1 (RNA) is the most expressed isoform in the central nervous system and is primarily found in neurons[30]. CerS2 is the second most abundant, with its high RNA expression observed in oligodendrocytes during periods of active myelination. CerS2 null mice show reduced abundances of very long chain sphingolipids[31]. However, current knowledge of CerS relies heavily on murine studies[25,32,33]. To date, few studies have examined the expression of CerS[34] in human brain tissue or Huntington's disease.

For Review Only

Neural ceramide concentrations are low due to their constant metabolism. Sphingomyelin is synthesised from ceramide via the addition of a phosphatidylcholine head group via sphingomyelin synthase (**Figure 1**) and has a much higher abundance in the brain. This makes sphingomyelin useful as a reserve for ceramide production. Consequently, changes in the sphingomyelin profile can greatly affect ceramide concentrations[23]. Sphingomyelin is primarily synthesised in oligodendrocytes and is an essential component of cell and myelin membranes. Due to increased oligodendroglial densities in Huntington's disease, there may be consequential increases in sphingomyelin[35,36].

Ceramide can also be metabolised into more complex glycosphingolipids (**Figure 1**). Glycosphingolipids are associated with crucial periods of neuronal development and myelination and act as second messengers for cell signalling[37]. Glucosylceramide and galactosylceramide, are the simplest glycosphingolipids, arising from the addition of either a glucose or galactose ring to the terminal hydroxyl group of ceramide[20]. The addition of galactose to the existing glucose ring of glucosylceramide creates lactosylceramide, an important precursor to gangliosides[20]. Galactosylceramide, on the other hand, enters a different synthesis pathway to be converted to sulfatides by the addition of a sulphur group[20]. Galactosylceramides and sulfatides contribute significantly to myelin and are proposed to assist in myelin membrane stability and saltatory conduction[37].

Multiple experimental models of Huntington's disease have disturbances to sphingolipid metabolism, notably to sphingosine-1-phosphate, a precursor to ceramide production[16]. Transgenic Huntington's mice (R6/1; polyQ=115), despite unchanged total abundances of glycosphingolipids, have reductions in specific glycosphingolipid species (GM1 gangliosides) in their neurons[19,38]. These mice also have alterations to several glycan-transferases, ganglioside degradation proteins[19,38] and CerS[16]. Post-mortem Huntington's disease frontal cortex and caudate has extracellular deposits of cerebrosides, akin to those found in cerebroside storage disorders (Gaucher's Disease, Krabbe's Disease) and multiple sclerosis patients[39]. The neighbouring subventricular zone, which borders the caudate, has increased abundancies of sphingomyelin and deficiencies of sulfatides in Huntington's disease patients[40].

Despite the many disturbances to sphingolipid metabolism observed in Huntington's disease models, an understanding of molecular-level and region-specific effects on the expression of sphingolipids in human Huntington's disease tissue is lacking. Region-specific alterations to cholesterol metabolism have already been identified in Huntington's striatum[17,41] and our

https://mc.manuscriptcentral.com/braincom

For Review Only

research aimed to determine if these alterations extended to region-specific effects on sphingolipids in Huntington's disease. We show for the first time, variations in the availability of sphingolipids governed by the length of their fatty acyl chain in Huntington's caudate, a feature consistent with several other neurodegenerative diseases[34,42,43].

Materials and methods

Human Brain Tissue

The Victorian Brain Bank provided post-mortem brain tissue from 13 advanced Huntington's disease subjects and 13 age and sex-matched controls (male n = 8, female n = 5/group). The tissue provided was from five key regions: caudate, putamen, cerebellum, and grey and white dorsomedial prefrontal cortex. Subject demographics are provided in **Table 1**. The clinical disease stage was measured using the Unified Huntington's Disease Rating Scale. All Huntington's disease tissue had a pathological Vonsattel grading of IV, the most severe[44]. The CAG repeat of Huntington's patients was assessed by the Victorian Clinical Genetics Service (2020). The post-mortem interval, brain pH and age were not significantly different between Huntington's disease and control brains[17]. Tissue was stored at -80°C until use. Ethics approval was obtained from the UOW Human Research Ethics Committee (HE10/327) and was carried out in accordance with the Declaration of Helsinki (2008).

For Review Only

Lipid Nomenclature

Lipid nomenclature and abbreviations are consistent with recommendations for sphingolipids[45]. Examples are provided in **Supplementary Table 1**. The fatty acid notation describes the number of carbons and double bond equivalents in the fatty acyl chain *i.e.* 16:1 has 16 carbons and 1 double bond equivalent. **Figure 1** highlights sphingolipid metabolic pathways. The molecular structure of analysed sphingolipid classes is provided in **Supplementary Figure S2**.

Lipid Extractions

Ceramide and Sphingomyelin

Lipids were extracted as described previously[46,47]. Brain tissue (10 mg) was homogenized using a bead homogenizer (FastPrep-24, MP Bio, Sydney, Australia) at 6 m/s for 40 seconds, using 600 mg of 1.4 mm ceramic beads in 300 µL of methanol (LC-MS grade; Bio-Strategy, Murarrie, Australia) containing 0.01% butylated hydroxyl-toluene (BHT; Sigma Aldrich, Missouri, USA) and internal standards (1 nmol ceramide 17:0, 5 nmol dihydrosphingomyelin d18:0/12:0; Avanti Polar Lipids, Alabama, USA). The homogenate was transferred into 2 mL glass vials, 920 µL Methyl tert-butyl ether (HPLC grade; Bio-Strategy, Murarrie, Australia) was added and the samples rotated for 1 hour at room temperature. Ammonium acetate (HPLC grade; Sigma Aldrich, Castle Hill, Australia) was added (230 µL of 150 mM) and the samples vortexed for 20 seconds before being centrifuged at 2000 × *g* for 5 minutes. The top organic phase was removed from each sample without disturbing the bottom aqueous phase and transferred into a new 2 mL glass vial before storage at -20°C.

To enhance sphingolipid detection, lipid extracts (300 µL) were subjected to base hydrolysis to remove glycerophospholipids[48,49]. To the extract, 90 µL of methanol (0.01% BHT) and 25 µL of 10 M sodium hydroxide (Bio-Strategy, Murarrie, Australia) was added, and the samples rotated overnight at 4°C. Following this, 90 µL of 150 mM ammonium acetate was added, and samples were vortexed before centrifugation at 2000 × g for 5 minutes. The top phase was removed (approximately 100 µL) and stored in new glass vials at -20°C. Extracts were diluted 100-fold in methanol:chloroform (LC-MS grade; Bio-Strategy, Murarrie, Australia) (2:1 v/v with 5 mM ammonium acetate) for mass spectrometric analysis of ceramide and sphingomyelin.

Glycosphingolipids

Glycosphingolipids were extracted from brain regions as previously described[50,51]. Brain tissue was homogenised by sonication probe (Misonix; Farmingdale, NY) in 0.02M Tris (pH 7) containing 0.5M NaCl and 0.1% Nonidet P-40. Total protein was determined by the method of Lowry et al. (1951)[52] and lipids were extracted from 0.1 mg protein in 10 µL by addition of 0.2 mL chloroform:methanol (2:1) containing 10 pmol of lactosylceramide 18:1;O2/16:0 (d_3), galactosylceramide 18:1;O2/15:0 and trihexosylceramide 18:1;O2/17:0 as internal standards. All standards were purchased from Matreya LLC (State College, PA). Samples were vortexed

For Review Only

before being shaken on a platform shaker (10 min, 150 opm), sonicated in a water bath for 30 min and allowed to stand at room temperature for 20 min. Samples were then centrifuged (10 min, 13,000 x g) to sediment protein and the supernatant was transferred to a 96 well plate and dried under a gentle stream of nitrogen at 40°C. Dried samples were stored at -20°C until analysis by liquid chromatography coupled with electrospray ionization tandem mass spectrometry (LC-ESI-MS/MS).

Mass Spectrometry

Ceramide and Sphingomyelin

Nanoelectrospray ionization mass spectrometry of lipid extracts was performed using a hybrid triple quadrupole linear ion trap mass spectrometer (QTRAP 5500, Sciex, Concord, Canada), equipped with an automated chip-based nanoelectrospray source (TriVersa Nanomate, Advion Biosciences, Ithaca, USA) as described previously[53]. Samples were loaded onto a 96-well plate (Eppendorf Twin-Tec 96) and sealed before direct infusion. Spray parameters were set at a gas pressure of 0.4 psi and a voltage of 1.2 kV[47,53]. Declustering potential was set to 100 V, collision cell exit potential 8 V, entrance potential 10 V and scan rate at 200 Da/s. Lipid data was acquired by targeted precursor ion scans as shown in **Supplementary Table S2.** Target lists for molecular lipid species within each class were generated after the manual review of spectra in Analyst (v1.6, Sciex, Ontario, Canada). Mass spectrometry data were analysed and quantified using LipidView software (v1.2, Sciex, Canada). Processing settings were set at a mass tolerance of 0.5 Da, and minimum signal/noise of 10. Smoothing and deisotoping were enabled. Lipids were quantified by comparison of peak areas to class-specific internal standards after isotope correction[54].

Glycosphingolipids

Glycosphingolipid analysis was performed by LC-ESI-MS/MS as previously described[51] using a Shimadzu Nexera *x*2 LC system (Shimadzu Corp., Kyoto, Japan) coupled with a SCIEX QTRAP 6500 triple quadrupole mass spectrometer (SCIEX, Framingham, MA). Samples were reconstituted in 100 µL of 10 mM ammonium formate in methanol and partial separation of the lipids was achieved by injection of 1 µL onto a Zorbax Eclipse C18 column (2.1 x 50 mm; 1.8 µm; Agilent Technologies) maintained at 40°C with an Agilent 1290 inline filter containing a 0.3 µm frit placed in front of the column. Mobile phase A contained water:acetonitrile (60:40)

https://mc.manuscriptcentral.com/braincom

and 10 mM ammonium formate, while mobile phase B contained isopropanol:acetonitrile (90:10) with 10 mM ammonium formate. The flow rate was 0.4 mL/min and the column was equilibrated at 10% mobile phase B before a linear ramp to 50% by 2 min and 100% B at 8 min. This was held for 0.5 min before a return to 10% B at 9 min and re-equilibration for 1 min before the next injection. The first 1 min was diverted to waste before being directed into the electrospray source (spray voltage 5.5 kV) in positive ion mode. Ion source temperature was 250°C, curtain gas was 25 units, collision gas set at medium; nebulizer gas 1 at 20 units and auxiliary gas 2 at 40 units.

In this method, the stereoisomers glucose and galactose cannot be separated and are reported using the generic term hexose (hex). Individual species of HexCer, Hex2Cer and SHexCer were quantified using scheduled multiple reaction monitoring with concentrations determined in Multiquant 3.0.1 software (SCIEX, Framingham, MA) by relating the peak area of the analyte to the peak area of the internal standard (noting that a trihexosylceramide internal standard was used to quantify SHexCer).

For Review Only

Western Blotting

Human brain samples were homogenised in buffer (0.1M Tris-HCl, 2mM EDTA, glycerol 10% v/v, 0.5 mM phenylmethylsulfonyl fluoride Protease Inhibitor Cocktail (P8340, Sigma, Australia) and Phosphatase Inhibitor Cocktail 2 (Sigma, Australia)) using a bead homogeniser (Fast-Prep 24, MP Bio, Sydney, Australia) for 40 seconds at 6 m/s. Samples (12.5 µg total protein for CerS2, 10 µg protein for CerS1) were loaded on a 4-12% Criterion Stain-Free (Bio-Rad, California, USA) gel in triplicate, with a pooled sample being run on each gel to standardise measurements between membranes. Electrophoresis was performed at 180 V for 50 minutes in SDS-PAGE buffer. The gels were washed in western transfer buffer (20% methanol) and transferred onto 0.2 µM polyvinylidene difluoride membranes at 100V for 1 hour. Membranes washed and blocked in 5% skim milk in tris buffered saline with Tween® 20 (TBST) for 1 hour at room temperature before overnight incubation at 4°C in the primary antibody ((anti-Ceramide Synthase 1 1:5,000, [Recombinant] (ab131169); Abcam, Cambridge, United Kingdom) or (anti-Ceramide Synthase 2 1:1,000 (ab176709), Abcam, Cambridge, United Kingdom)) in 2.5% milk in TBST. Membranes were then washed in TBST and incubated in secondary antibody (goat x anti-rabbit (AP307P) 1:5,000, Merck Millipore, Massachusetts, USA) in 2.5% milk in TBST for 1 hour at room temperature. Membranes were washed in TBST before being visualised by chemiluminescence. Membranes were stripped

with stripping buffer (ThermoFisher, Massachusetts, USA), washed in TBST, re-blocked and incubated with either glyceraldehyde 3-phosphate dehydrogenase (GAPDH) or β-actin before re-imaging. Expression of CerS1 and CerS2 was normalised to pooled samples and housekeepers. Detailed antibody information is available in **Supplementary Table S3**.

Statistical Analysis

Outliers were identified using a 2.2 interquartile range rule from the mean of each respective lipid/enzyme total concentration. Data was assessed for normality using a D'Agostino Pearson Omnibus test. Testing for relevant data is indicated in figure captions. For testing between HD and control, data was assessed using an unpaired, two-tailed t-test with Welch's correction or a Mann Whitney U test where appropriate. All means, standard error of the mean, and exact p values for this testing is available in relevant **Supplementary Tables S4-28** and is indicated in results. The significance level was set at $p<0.01$. Processed lipid and protein values for individual subjects are available in the **Supplementary Excel Data File**. Correlations were conducted using a Pearson's correlation where all data was normally distributed, and a Spearman's correlation where not all data was normally distributed. Correlations are reported with their respective r, r^2 and p values, and the 95% confidence intervals. All statistical tests were conducted using GraphPad Prism (v8, Massachusetts, USA) and SPSS (v25, USA).

For Review Only

Data availability

Processed lipid and protein values and large correlation tables are available in the **Supplementary Excel Data file.** Full western blots have been provided in **Supplementary Material (Figures).**

Results

Sphingolipid Class Totals

To determine if total lipid abundances differed between control regions, a one-way ANOVA was performed for each subclass of lipid (Cer, SM, HexCer, Hex2Cer, SHexCer, *OH*-SHexCer) (**Supplementary Figure S3**). For all lipid subclasses, the white cortex had significantly higher sphingolipid abundances than all grey matter regions, including the grey cortex (p=0.000). Typically, concentrations of sphingolipid classes were comparable across grey matter regions,

except for the putamen, which had higher concentrations of Cer (278 ± 23 *vs* 138 ± 11 pmol/mg tissue, p=0.006) (**Supplementary Figure 3A**) than the cerebellum. Additionally, the grey cortex had higher abundances of SM compared to the caudate (7,685 ± 945 *vs* 3,642 ± 162 pmol/mg tissue, p=0.003) (**Supplementary Figure 3B**). The caudate and putamen had the same concentrations of all lipid subclasses, while the cerebellum was the region with the lowest lipid abundances.

Total abundances of each lipid class were then compared with HD subjects. The only difference detected between the two groups was a higher abundance of Hex2Cer in the cerebellum of HD subjects (+65%, p=0.009).

Sphingolipid Species

For Review Only

In total, between the five regions, we identified sixty-five Cer, SM, HexCer, Hex2Cer and SHexCer species. The cortex had a more limited variety of ceramide and sphingomyelin species, containing 11 fewer species than the striatum and cerebellum. To determine the influence of acylation on each of the sphingolipid classes we assessed region-specific changes in sphingolipid species between Huntington's disease and control patients. Of the species identified in Huntington's patients; eighteen were significantly altered in the caudate (28%), nine in the putamen (14%), four in the cerebellum (1%) and none were altered in either the white or grey cortex.

Fatty acyl chain length alterations in Huntington's caudate

In the caudate, Huntington's disease subjects had an increased abundance of long chain sphingolipid species (C13-C21) alongside a decreased abundance of very long chain species (C22-C26) (**Figure 2**). This shift occurred predominately in sphingomyelin (**Figure 2B**) and Hex2Cer (**Figure 2D**). Although also occurring in ceramide, we did not detect it in as many species (**Figure 2A**). Long chain species of SHexCer, which are derived from galactosylceramide, were decreased in Huntington's caudate by 40-50%: SHex-Cer 18:1;O2/22:0 (-46%, p=0.009), SHex-Cer 18:1;O2/24:0 (-47%, p=0.007) and SHex-Cer 18:1;O2/24:1 (-53%, p=0.006) (**Figure 2E**). However, Hex2Cer, derived from glucosylceramide had the same alteration in sphingolipid chain length as sphingomyelin and ceramide (**Figure 2D**). Overall, in Huntington's caudate sphingolipids with C16:0 chains were increased in ceramides (+66%, p=0.009), sphingomyelins (+46%, p=0.001) and Hex2Cer (+36%, p=0.006), whilst those with C24:1 were decreased in ceramides (-29%, p=0.006),

sphingomyelins (-35%, p=0.003), Hex2Cer (-21%, p=0.000) and SHexCer (-53%, p=0.006). Due to the changes in sphingolipid fatty acyl chain length, CerS specific for these chain lengths were investigated in the striatum.

Increased SM species in Huntington's putamen

One species of Cer was found to be different in Huntington's putamen: Cer 18:1;O2/16:0 (+28%, p=0.001) (**Figure 3A**). Of the twenty-two SM species detected in the putamen, eight had an increased concentration in Huntington's disease subjects. These species were increased by between 50 and 100% compared to controls and included both long and very long chain species: SM 18:1;O2/14:0 (+62%, p=0.001), SM 18:1;O2/15:0 (+135%, p=0.001), SM 18:1;O2/16:0 (+52%, p=0.002), SM 18:1;O2/16:1 (+122%, p=0.000), SM 18:1;O2/17:0 (+91%, p=0.000), SM 18:1;O2/18:1 (+128%, p=0.000), SM 18:1;O2/22:1 (+119%, p=0.004) and SM 18:1;O2/26:2 (+79%, p=0.008) (**Figure 3B**). No differences in HexCer, Hex2Cer or SHexCer species was detected (**Figure 3C-F**).

Increased Hex2Cer species in Huntington's cerebellum

Huntington's disease patients had an increased abundance of Hex2Cer (**Figure 4D**) in the cerebellum driven by increases in three Hex2Cer species. The largest increase was in Hex2Cer 18:1;O2/16:0 (+145%, p=0.002), whilst the remaining species had increases of 78% (Hex2Cer 18:1;O2/22:0, p=0.006), and 85% (Hex2Cer 18:1;O2/24:1, p=0.006). Cer, SM, HexCer, SHexCer and OH-SHexCer species showed no remarkable differences in Huntington's disease subjects (**Figure 4A-C, E-F**).

The preservation of sphingolipid homeostasis in the dorsomedial prefrontal cortex of Huntington's patients

No differences in any sphingolipid species between Huntington's disease patients and controls were detected in either the white (**Supplementary Figure S4**) or grey (**Supplementary Figure S5**) matter of the dorsomedial prefrontal cortex.

Ceramide Synthases

To determine if the chain length alterations in sphingolipid species were a result of changes to CerS, we performed western blot analyses of two isoforms. CerS1 and CerS2 were chosen due to their specificity for the sphingolipid acyl chain lengths affected (CerS1 C18; CerS2 C22-

For Review Only

C26), the high abundance of these sphingolipid species in our analysis, and the high mRNA expression of these isoforms in mouse brain[30,55]. Before adjusting for CerS expression, the expression of housekeeping proteins was assessed to see if there were any significant differences between Huntington's subjects and controls. In the putamen, we found a significant difference in the expression of β-actin (-14 to -44% in Huntington's disease), so this housekeeper was not used for our analysis of the putamen (**Supplementary Table S29**). Full western blot images are available in **Supplementary Figures S5-S8**.

CerS1

The expression of the CerS1 primary band (~40 kDa) was lower in Huntington's disease caudate compared to controls when adjusted for both β-actin (-57.60%, p=0.003) (**Figure 5B**) and GAPDH (-23.34%, p=0.009) (**Figure 5D**). No differences were detected for CerS1 primary band in the putamen of Huntington's disease subjects. Analysis of the secondary band observed at ~45 kDa also revealed no differences between Huntington's and control subjects in either the caudate or putamen.

The Age at Death and CAG repeat number are reported to be strongly correlated, and we confirmed this by running a Pearson's correlation analysis using our data (r=-0.7443, p=0.009) (**Supplementary Figure S10**). Pearson's correlation analyses were used to examine the relationship between CerS1 expression and Age at Death or CAG repeat length in Huntington's disease subjects (**Figure 6**). A strong positive correlation between CerS1 expression and Age at Death (r=0.7251, p=0.005; **Figure 6A**) was identified in Huntington's caudate, alongside a strong negative correlation between CerS1 expression and CAG repeat length (r=-0.6975, p=0.008; **Figure 6B**). No relationship between CerS1 was identified with Age at Death (r=0.4945, p=0.146; **Figure 6C**) or CAG repeat length (r=-0.3916, p=0.263; **Figure 6D**) in Huntington's putamen. No relationship between CerS1 expression and Age at Death was found in controls for either caudate (r=0.4873, p=0.108) or putamen (r=0.5401, p=0.086) (**Supplementary Tables S30-S31**).

Due to the specificity of CerS1 for C18 fatty acyl chains, spearman's correlation analyses were conducted to determine if a relationship existed between the expression of CerS1 (by β-actin) and sphingolipids with C18:0 fatty acyl chains. No correlations were found for any lipid type with CerS1 in both control and Huntington's disease subjects (**Supplementary Excel File Tab 7**).

For Review Only

CerS2

No differences in the expression of CerS2 between control and HD samples were identified in either the caudate or the putamen (**Figure 5F, 5H**). CerS2 expression was not correlated with Age at Death or CAG repeat length in HD subjects in either the caudate (**Figure 7A-B**) or putamen (**Figure 7C-D**). No correlations were identified between CerS2 expression and Age at Death for controls (**Supplementary Tables S30-S31**).

Additional banding of CerS2 was identified in four of the thirteen HD subjects. No discernible underlying factor including sex, Age at Death, post-mortem interval, brain pH or CAG repeat length, was associated with the expression of additional bands (**Supplementary Table S32**). A CerS2 knockout cell lysate was used as a negative control, and wild type cell lysate as a positive control to assess antibody specificity for the CerS2 protein. We confirmed the specificity of the antibody and the absence of disulphide bonding as a cause of the additional banding (**Supplementary Methods & Figure S1**).

Due to the specificity of CerS2 for C22-C24 fatty acyl chains, spearman's correlations were used to determine relationships for both HD and control subjects. No correlations between CerS2 expression (by GAPDH) and C22-C24 fatty acyl chains for any sphingolipid were found (**Supplementary Excel File Tab 7**).

Discussion

A key finding of this investigation is the shift in fatty acyl chain length of sphingolipids in the caudate of clinically advanced Huntington's disease patients. We were able to identify increased abundances of long-chain species and decreased abundances of very-long-chain species of ceramide, sphingomyelin and lactosylceramide. We cannot exclude the possibility that these shifts are also occurring in glucosylceramide, due to the significantly higher abundance of galactosylceramide in the brain[56] and the inability of the applied mass spectrometric techniques to distinguish between galactosylceramide and glucosylceramide. Shifts in the ratio of long and very long chain sphingolipids in the brain are indicative of '*immature myelin*'[57] and occur in adrenoleukodystrophy[42,58,59], frontotemporal dementia with Pick's disease[60], multiple sclerosis[61] and Parkinson's disease[34]. Parkinson's disease and Huntington's disease share a significant disturbance of the basal ganglia and dopaminergic pathways[62,63]. In Parkinson's disease, Cer 18:1;O2/18:0 and SM 18:1;O2/18:1 are increased,

For Review Only

whilst Cer 18:1;O2/24:1, SM 18:1;O2/23:0, SM 18:1;O2/24:1 and SM 18:1;O2/26:1 are decreased in the grey matter of the anterior cingulate cortex[34]. These disturbances do not occur in the white matter of the anterior cingulate cortex or the occipital cortex, showing a region-specific shift in the abundance of sphingolipid species by fatty acyl chain length, in an area significantly affected in Parkinson's disease[34]. The increased long chain and decreased very long chain sphingolipids identified in Huntington's caudate were also region-specific and occurred in the most significantly affected brain region in Huntington's disease[6,44]. The alterations in the anterior cingulate cortex of Parkinson's disease and now in the caudate of Huntington's disease, indicate that disturbances to sphingolipids in neurological disorders may not solely correlate with myelin disturbances. Our analysis did not find these shifts in the white matter of the frontal cortex, a region that is also severely affected in Huntington's disease and is heavily myelinated[64]. These increases in the abundance of long over very long chain sphingolipids may reflect changes to the lipid profile of neuronal membranes and/or synapses[65]. Altering the lipid profile of neuronal membranes can have profound consequences for the fluidity and permeability of the membrane[66]. These changes can influence the organization of the lipid membrane, including the placement of integral proteins, affecting numerous neuronal capabilities such as receptor binding[21]. Since our lipid extractions are from whole brain tissue, it is difficult to determine whether these changes are due specifically to defects in axons, synapses, or myelin.

The increases in long-chain ceramide (16:0) in Huntington's caudate may promote apoptosis in cells. Increases in long-chain ceramides, specifically 16:0 species, are associated with dysregulated apoptosis[67]. In immortalized human cervical cancer cells (HeLa cells), increased expression of CerS2 (C22-C24) has protective effects against apoptosis, whilst an increased expression of CerS5 (C16) promotes apoptosis[68]. Possible modifications to CerS2 in Huntington's caudate may have diminished these protective effects and could underlie the disturbances to the synthesis of very-long-chain ceramides, affecting the downstream availability of very long chain sphingomyelin and lactosylceramide species. The use of CerS2 knockout cell lysates supported that the additional bands in the striatal western blots reported are related to the CerS2 protein (**Supplementary Figure S1**). CerS2 can be regulated post-transcriptionally by phosphorylation[69] or glycosylation[70], however, the specific modifications to CerS2 indicated in the western blots have not been determined. CerS2 knockout mice have defective myelin sheaths and reduced very-long-chain ceramides (24:0, 24:1) with a compensatory increase in long-chain ceramides (C16, C18)[28]. CerS2 knockout human HeLa

cell lines observed the same effect of increased C16 ceramides and decreased 24:0 and 24:1 ceramides, which increased the susceptibility of the cells to apoptosis[71]. These results suggest when CerS2 activity is disturbed, long-chain ceramides are synthesized in compensation. The balance of long and very-long-chain sphingolipid species may contribute to the susceptibility of a cell to apoptosis by influencing channel formation in the mitochondrial membrane and therefore permeability to pro-apoptotic molecules[68]. Mitochondrial dysfunction has been documented extensively in Huntington's disease brain as well as rodent and cell models[72–76]. The high energy demands of the striatal primary neuronal cell type, medium spiny neurons, are proposed to be the underlying reason for this region's susceptibility to mitochondrial dysfunction in Huntington's disease[77].

Huntington's disease subjects had a reduced expression of CerS1 localized to the caudate. The expression of CerS1 in the caudate was related to the age at death and the CAG repeat length of Huntington's patients. CAG repeat length is inversely correlated with age of onset and age of death in Huntington's disease. In our study, Huntington's patients with longer CAG repeats, and who died at an earlier age had lower CerS1 expression in the caudate. It is unclear as to why this relationship exists in HD. However, CerS1 has a high expression in neurons[32] and so lower concentrations of this enzyme may simply reflect a greater degree of neuronal cell loss brought on by a longer CAG repeat and more severe clinical symptoms[2,78]. The expression of neither CerS1 nor CerS2 correlated consistently with the concentrations of their respective sphingolipid species. CerS expression (mRNA) does not correlate directly with sphingolipid concentrations *in vivo*[28,34,79]. The multiple pathways that feed into the production and recycling of ceramide, as well as possible post-translational modifications and the overlapping specificity of CerS, mean that the expression of these enzymes cannot solely explain these sphingolipid changes. In addition, the expression of CerS proteins does not necessarily reflect the activity of these enzymes, which could influence sphingolipid concentrations. An increased expression of other relevant CerS isoforms (CerS4, CerS5, CerS6) may have also contributed.

The putamen and cerebellum had distinctive increases in sphingolipid species in Huntington's disease patients. In Huntington's whilst the putamen shared increases in very long chain sphingomyelin with the caudate, it had additional increases in long chain sphingomyelin species. Increased sphingomyelin content limits the insertion of htt (35Q) into lipid bilayers in cultured cells[80], so this may be a protective mechanism. The cerebellum, which has a Purkinje cell dominant population, had elevations in several lactosylceramide species. This region suffers varying degrees of atrophy and lipid disturbance in Huntington's disease, and

For Review Only

alterations to this region are typically more severe in juvenile cases. Lactosylceramide is an important mediator of astrogliosis (increase in astrocytes) and inflammation[81]. In other pathologies such as inflammatory bowel diseases and emphysema, elevations occur alongside inflammation, apoptosis and autophagy[82,83]. Astrogliosis occurs in multiple brain regions in Huntington's disease, including the cerebellum[84].

In this study, both the white and grey matter of a specific cortical functional region were used for comparative analysis. The dorsomedial prefrontal cortex is associated with social cognition[85] which is impaired early in Huntington's disease[3]. The cerebral cortex, although not as severely affected as the striatum in Huntington's, still experiences mass losses of approximately 30%, the frontal cortex being a predominant location of this loss[6,64,86]. Therefore, the absence of alterations to sphingolipids in the cerebral cortex was an unanticipated occurrence. Decreases in several sphingolipid precursors have been identified in R6/2 transgenic mouse models[16], and there is limited information available in human post-mortem tissue. Our findings reflect only a small portion of the cerebral cortex, so we cannot rule out alterations to sphingolipid metabolism in other cortical regions (i.e. parietal, occipital). Previous analysis has identified that the overall lipid profile of the frontal cortex is relatively preserved in aging[87,88], and cortical regions that are more proximal to the striatum (corpus callosum) typically degenerate earliest in Huntington's disease[86]. The cortical neurons of the dorsomedial prefrontal cortex may resist disturbances to sphingolipid homeostasis in Huntington's, leaving it relatively preserved. Since rates of atrophy differ between the white and grey matter of the frontal cortex[89–91], it was expected that these regions would show unique differences in sphingolipid metabolism, however, this was not the case. The lipid concentrations of the grey matter in Huntington's subjects were extremely variable (**Supplementary Figure S5)** making statistical analysis challenging. Significant variations in the disturbance of cortical regions is an element of Huntington's disease pathology[92].

Changes to the neural sphingolipid composition in people with Huntington's disease is determinant on the brain region. Although the striatum is traditionally considered a single region, the caudate and putamen have distinctive shifts to their sphingolipid profiles, despite sharing the same neural cell populations. For the first time, an increased abundance of long chain and a corresponding decreased abundance of very long chain ceramides, sphingomyelins and lactosylceramides, has been identified in the caudate of clinically advanced Huntington's patients. The possibility of post-translational modifications of CerS2 driving these changes cannot be excluded, as these may contribute to the striatum's vulnerability to sphingolipid, and

For Review Only

possibly mitochondrial dysfunction in Huntington's disease. The unique neuronal cell populations of the cortex, striatum and cerebellum distinguish them from one another, and this may contribute to their susceptibility to sphingolipid disturbance. The shifted sphingolipid profile of the caudate may provide clues as to how regional neural cell dysfunction occurs not only in Huntington's disease but in related neurodegenerative diseases.

Acknowledgements

The authors acknowledge the assistance of Dr Catriona Mclean in acquiring the human post-mortem tissue and providing pathological and clinical information. GRP would like to thank Mr Richard Miller for her Miller Bridge-Water Scholarship for Dementia Research. The authors would also like to thank the generous donor who supports this research and wishes to remain anonymous, as well as the Victorian Clinical Genetics Service for determining the polyQ length of our HD subjects.

For Review Only

Funding

This research is supported by an anonymous philanthropic donor. We thank her for her generosity and wonderful support.

Competing interests

The authors report no competing interests.

Supplementary material

Supplementary material is available at *Brain* online.

References

1. Macdonald M. A novel gene containing a trinucleotide repeat that is expanded and unstable on Huntington's disease chromosomes. *Cell*. 1993;72(6):971-983. doi:10.1016/0092-8674(93)90585-E

2. Keum JW, Shin A, Gillis T, et al. The HTT CAG-Expansion Mutation Determines Age at Death but Not Disease Duration in Huntington Disease. *The American Journal of Human Genetics*. 2016;98(2):287-298. doi:10.1016/j.ajhg.2015.12.018

3. Kirkwood SC, Su JL, Conneally PM, Foroud T. Progression of Symptoms in the Early and Middle Stages of Huntington Disease. *Arch Neurol*. 2001;58(2):273-278. doi:10.1001/archneur.58.2.273

4. Cattaneo E, Zuccato C, Tartari M. Normal huntingtin function: an alternative approach to Huntington's disease. *Nature Reviews Neuroscience*. 2005;6(12):919-930. doi:10.1038/nrn1806

5. Fossale E, Seong IS, Coser KR, et al. Differential effects of the Huntington's disease CAG mutation in striatum and cerebellum are quantitative not qualitative. *Human Molecular Genetics*. 2011;20(21):4258-4267. doi:10.1093/hmg/ddr355

6. Halliday GM, McRitchie DA, Macdonald V, Double KL, Trent RJ, McCusker E. Regional Specificity of Brain Atrophy in Huntington's Disease. *Experimental Neurology*. 1998;154(2):663-672. doi:10.1006/exnr.1998.6919

7. Báez-Mendoza R, Schultz W. The role of the striatum in social behavior. *Frontiers in Neuroscience*. 2013;7(233). doi:10.3389/fnins.2013.00233

8. Carpenter MB. Anatomy of the Corpus Striatum and Brain Stem Integrating Systems. In: Terjung R, ed. *Comprehensive Physiology*. John Wiley & Sons, Inc.; 2011. doi:10.1002/cphy.cp010219

9. Aylward EH. Change in MRI striatal volumes as a biomarker in preclinical Huntington's disease. *Brain Research Bulletin*. 2007;72(2-3):152-158. doi:10.1016/j.brainresbull.2006.10.028

10. Aylward EH, Sparks BF, Field KM, et al. Onset and rate of striatal atrophy in preclinical Huntington disease. *Neurology*. 2004;63(1):66-72. doi:10.1212/01.WNL.0000132965.14653.D1

11. Sieradzan KA, Mann DMA. The selective vulnerability of nerve cells in Huntington's disease. *Neuropathology and Applied Neurobiology*. 2001;27(1):1-21. doi:10.1046/j.0305-1846.2001.00299.x

12. Beasley M, Groover S, Valentine SJ, Legleiter J. Lipid headgroups alter huntingtin aggregation on membranes. *Biochimica et Biophysica Acta (BBA) - Biomembranes*. 2021;1863(1):183497. doi:10.1016/j.bbamem.2020.183497

13. Kegel KB, Sapp E, Yoder J, et al. Huntingtin Associates with Acidic Phospholipids at the Plasma Membrane. *Journal of Biological Chemistry*. 2005;280(43):36464-36473. doi:10.1074/jbc.M503672200

14. Naudí A, Cabré R, Dominguez-Gonzalez M, et al. Region-specific vulnerability to lipid peroxidation and evidence of neuronal mechanisms for polyunsaturated fatty acid biosynthesis in the healthy adult human central nervous system. *Biochimica et Biophysica Acta (BBA) - Molecular and Cell Biology of Lipids*. 2017;1862(5):485-495. doi:10.1016/j.bbalip.2017.02.001

For Review Only

15. Segatto M, Di Giovanni A, Marino M, Pallottini V. Analysis of the protein network of cholesterol homeostasis in different brain regions: An age and sex dependent perspective. *Journal of Cellular Physiology*. 2013;228(7):1561-1567. doi:10.1002/jcp.24315

16. Di Pardo A, Basit A, Armirotti A, et al. De novo Synthesis of Sphingolipids Is Defective in Experimental Models of Huntington's Disease. *Frontiers in Neuroscience*. 2017;11(698). doi:10.3389/fnins.2017.00698

17. Kreilaus F, Spiro AS, McLean CA, Garner B, Jenner AM. Evidence for altered cholesterol metabolism in Huntington's disease *post mortem* brain tissue: Altered cholesterol metabolism in Huntington's disease. *Neuropathology and Applied Neurobiology*. 2016;42(6):535-546. doi:10.1111/nan.12286

18. Phillips GR, Hancock SE, Brown SHJ, et al. Cholesteryl ester levels are elevated in the caudate and putamen of Huntington's disease patients. *Scientific Reports*. 2020;10(20314):11. doi:https://doi.org/10.1038/s41598-020-76973-8

19. Desplats PA, Denny CA, Kass KE, et al. Glycolipid and Ganglioside Metabolism Imbalances In Huntington's Disease. *Neurobiol Dis*. 2007;27(3):265-277. doi:10.1016/j.nbd.2007.05.003

20. D'Angelo G, Capasso S, Sticco L, Russo D. Glycosphingolipids: synthesis and functions. *FEBS Journal*. 2013;280(24):6338-6353. doi:10.1111/febs.12559

21. Fantini J, Barrantes FJ. Sphingolipid/cholesterol regulation of neurotransmitter receptor conformation and function. *Biochimica et Biophysica Acta (BBA) - Biomembranes*. 2009;1788(11):2345-2361. doi:10.1016/j.bbamem.2009.08.016

22. Gulati S, Liu Y, Munkacsi AB, Wilcox L, Sturley SL. Sterols and sphingolipids: Dynamic duo or partners in crime? *Progress in Lipid Research*. 2010;49(4):353-365. doi:10.1016/j.plipres.2010.03.003

23. Hannun YA, Obeid LM. Principles of bioactive lipid signalling: lessons from sphingolipids. *Nature Reviews Molecular Cell Biology*. 2008;9(2):139-150. doi:10.1038/nrm2329

24. Mencarelli C, Martinez–Martinez P. Ceramide function in the brain: when a slight tilt is enough. *Cellular and Molecular Life Sciences*. 2013;70(2):181-203. doi:10.1007/s00018-012-1038-x

25. Laviad EL. Characterization of Ceramide Synthase 2 TISSUE DISTRIBUTION, SUBSTRATE SPECIFICITY, AND INHIBITION BY SPHINGOSINE 1-PHOSPHATE. *The Journal of biological chemistry*. 2008;283(9):5677-5684. doi:10.1074/jbc.M707386200

26. Mullen TD, Hannun YA, Obeid LM. Ceramide synthases at the centre of sphingolipid metabolism and biology. *Biochem J*. 2012;441(3):789. doi:10.1042/BJ20111626

27. Agranoff BW, Benjamins JA, Hajra AK. Properties of Brain Lipids. Published online 1999. Accessed April 9, 2018. https://www.ncbi.nlm.nih.gov/books/NBK28219/

For Review Only

28. Grösch S, Schiffmann S, Geisslinger G. Chain length-specific properties of ceramides. *Progress in Lipid Research*. 2012;51(1):50-62. doi:10.1016/j.plipres.2011.11.001

29. Ben-David O, Futerman AH. The Role of the Ceramide Acyl Chain Length in Neurodegeneration: Involvement of Ceramide Synthases. *NeuroMolecular Medicine*. 2010;12(4):341-350. doi:10.1007/s12017-010-8114-x

30. Levy M, Futerman AH. Mammalian ceramide synthases. *IUBMB Life*. Published online 2010:NA-NA. doi:10.1002/iub.319

31. Schmitt S, Cantuti Castelvetri L, Simons M. Metabolism and functions of lipids in myelin. *Biochimica et Biophysica Acta (BBA) - Molecular and Cell Biology of Lipids*. 2015;1851(8):999-1005. doi:10.1016/j.bbalip.2014.12.016

32. Becker I, Wang-Eckhardt L, Yaghootfam A, Gieselmann V, Eckhardt M. Differential expression of (dihydro)ceramide synthases in mouse brain: oligodendrocyte-specific expression of CerS2/Lass2. *Histochem Cell Biol*. 2008;129(2):233-241. doi:10.1007/s00418-007-0344-0

33. Spassieva SD, Ji X, Liu Y, et al. Ectopic expression of ceramide synthase 2 in neurons suppresses neurodegeneration induced by ceramide synthase 1 deficiency. *Proceedings of the National Academy of Sciences*. 2016;113(21):5928-5933. doi:10.1073/pnas.1522071113

34. Abbott SK, Li H, Muñoz SS, et al. Altered ceramide acyl chain length and ceramide synthase gene expression in Parkinson's disease: Altered Ceramide in Parkinson's Disease. *Movement Disorders*. 2014;29(4):518-526. doi:10.1002/mds.25729

35. Gómez-Tortosa E, MacDonald ME, Friend JC, et al. Quantitative neuropathological changes in presymptomatic Huntington's disease. *Ann Neurol*. 2001;49(1):29-34.

36. Myers RH, Vonsattel JP, Paskevich PA, et al. Decreased neuronal and increased oligodendroglial densities in Huntington's disease caudate nucleus. *J Neuropathol Exp Neurol*. 1991;50(6):729-742. doi:10.1097/00005072-199111000-00005

37. Bosio A, Binczek E, Stoffel W. Functional breakdown of the lipid bilayer of the myelin membrane in central and peripheral nervous system by disrupted galactocerebroside synthesis. *Proceedings of the National Academy of Sciences*. 1996;93(23):13280-13285. doi:10.1073/pnas.93.23.13280

38. Maglione V, Marchi P, Di Pardo A, et al. Impaired Ganglioside Metabolism in Huntington's Disease and Neuroprotective Role of GM1. *Journal of Neuroscience*. 2010;30(11):4072-4080. doi:10.1523/JNEUROSCI.6348-09.2010

39. den Hartog Jager WA. A histochemical study in Huntington's disease and control cases. *Histochemistry*. 1978;58(4):273-280. doi:10.1007/BF00495383

40. Hunter M, Demarais NJ, Faull RLM, Grey AC, Curtis MA. Subventricular zone lipidomic architecture loss in Huntington's disease. *Journal of Neurochemistry*. 2018;146(5):613-630. doi:10.1111/jnc.14468

For Review Only

41. Phillips GR, Hancock SE, Brown SHJ, et al. Cholesteryl ester levels are elevated in the caudate and putamen of Huntington's disease patients. *Scientific Reports*. 2020;10(1):20314. doi:10.1038/s41598-020-76973-8

42. Molzer B, Bernheimer H, Budka H, Pilz P, Toifl K. Accumulation of very long chain fatty acids is common to 3 variants of adrenoleukodystrophy (ALD). *Journal of the Neurological Sciences*. 1981;51(2):301-310. doi:10.1016/0022-510X(81)90108-8

43. Theda C, Moser AB, Powers JM, Moser HW. Phospholipids in X-linked adrenoleukodystrophy white matter: fatty acid abnormalities before the onset of demyelination. *Journal of the Neurological Sciences*. 1992;110(1-2):195-204. doi:10.1016/0022-510X(92)90028-J

44. Vonsattel JP, Myers RH, Stevens TJ, Ferrante RJ, Bird ED, Richardson EP. Neuropathological classification of Huntington's disease. *J Neuropathol Exp Neurol*. 1985;44(6):559-577.

45. Liebisch G, Fahy E, Aoki J, et al. Update on LIPID MAPS classification, nomenclature, and shorthand notation for MS-derived lipid structures. *J Lipid Res*. 2020;61(12):1539-1555. doi:10.1194/jlr.S120001025

46. Matyash V, Liebisch G, Kurzchalia TV, Shevchenko A, Schwudke D. Lipid extraction by methyl- *tert* -butyl ether for high-throughput lipidomics. *Journal of Lipid Research*. 2008;49(5):1137-1146. doi:10.1194/jlr.D700041-JLR200

47. Abbott SK, Jenner AM, Mitchell TW, Brown SHJ, Halliday GM, Garner B. An Improved High-Throughput Lipid Extraction Method for the Analysis of Human Brain Lipids. *Lipids*. 2013;48(3):307-318. doi:10.1007/s11745-013-3760-z

48. Montgomery MK, Brown SHJ, Lim XY, et al. Regulation of glucose homeostasis and insulin action by ceramide acyl-chain length: A beneficial role for very long-chain sphingolipid species. *Biochimica et Biophysica Acta (BBA) - Molecular and Cell Biology of Lipids*. 2016;1861(11):1828-1839. doi:10.1016/j.bbalip.2016.08.016

49. Le Lay S, Li Q, Proschogo N, et al. Caveolin-1-dependent and -independent membrane domains. *Journal of Lipid Research*. 2009;50(8):1609-1620. doi:10.1194/jlr.M800601-JLR200

50. Saville JT, Fuller M. Sphingolipid dyshomeostasis in the brain of the mouse model of mucopolysaccharidosis type IIIA. *Mol Genet Metab*. 2020;129(2):111-116. doi:10.1016/j.ymgme.2019.08.008

51. Saville JT, Thai HN, Lehmann RJ, Derrick-Roberts ALK, Fuller M. Subregional brain distribution of simple and complex glycosphingolipids in the mucopolysaccharidosis type I (Hurler syndrome) mouse: impact of diet. *Journal of Neurochemistry*. 2017;141(2):287-295. doi:10.1111/jnc.13976

52. Lowry OH, Rosebrough NJ, Farr AL, Randall RJ. Protein Measurement with the Folin Phenol Reagent. *J Biol Chem*. 1951;193(1):265-275.

For Review Only

53. Tran PN, Brown SHJ, Mitchell TW, et al. A female gametocyte-specific ABC transporter plays a role in lipid metabolism in the malaria parasite. *Nature Communications*. 2014;5(1). doi:10.1038/ncomms5773

54. Deeley JM, Mitchell TW, Wei X, et al. Human lens lipids differ markedly from those of commonly used experimental animals. *Biochimica et Biophysica Acta (BBA) - Molecular and Cell Biology of Lipids*. 2008;1781(6-7):288-298. doi:10.1016/j.bbalip.2008.04.002

55. Schiffmann S, Birod K, Männich J, et al. Ceramide metabolism in mouse tissue. *The International Journal of Biochemistry & Cell Biology*. 2013;45(8):1886-1894. doi:10.1016/j.biocel.2013.06.004

56. Jones EE, Zhang W, Zhao X, et al. Tissue Localization of Glycosphingolipid Accumulation in a Gaucher Disease Mouse Brain by LC-ESI-MS/MS and High-Resolution MALDI Imaging Mass Spectrometry. *SLAS DISCOVERY: Advancing the Science of Drug Discovery*. 2017;22(10):1218-1228. doi:10.1177/2472555217719372

57. Stallberg-Stenhagen S, Svennerholm L. Fatty acid composition of human brain sphingomyelins: normal variation with age and changes during myelin disorders. *Journal of Lipid Research*. 1965;6:147-155.

58. Theda C, Moser AB, Powers JM, Moser HW. Phospholipids in X-linked adrenoleukodystrophy white matter: fatty acid abnormalities before the onset of demyelination. *Journal of the Neurological Sciences*. 1992;110(1):195-204. doi:10.1016/0022-510X(92)90028-J

59. Tsuji S, Ohno T, Miyatake T, Suzuki A, Yamakawa T. Fatty acid elongation activity in fibroblasts from patients with adrenoleukodystrophy (ALD). *Journal of Biochemistry*. 1984;96(4):1241-1247. doi:10.1093/oxfordjournals.jbchem.a134942

60. de Wit NM, den Hoedt S, Martinez-Martinez P, Rozemuller AJ, Mulder MT, de Vries HE. Astrocytic ceramide as possible indicator of neuroinflammation. *Journal of Neuroinflammation*. 2019;16(1):48. doi:10.1186/s12974-019-1436-1

61. Alling C, Vanier M-T, Svennerholm L. Lipid alterations in apparently normal white matter in multiple sclerosis. *Brain Research*. 1971;35(2):325-336. doi:10.1016/0006-8993(71)90478-1

62. Marsden CD. Dopamine and basal ganglia disorders in humans. *Seminars in Neuroscience*. 1992;4(2):171-178. doi:10.1016/1044-5765(92)90015-T

63. Pavese N, Andrews TC, Brooks DJ, et al. Progressive striatal and cortical dopamine receptor dysfunction in Huntington's disease: a PET study. *Brain*. 2003;126(5):1127-1135. doi:10.1093/brain/awg119

64. Estrada-Sánchez AM, Rebec GV. Role of cerebral cortex in the neuropathology of Huntington's disease. *Frontiers in Neural Circuits*. 2013;7. doi:10.3389/fncir.2013.00019

65. Calderon RO, Attema B, DeVries GH. Lipid Composition of Neuronal Cell Bodies and Neurites from Cultured Dorsal Root Ganglia. *Journal of Neurochemistry*. 1995;64(1):424-429. doi:https://doi.org/10.1046/j.1471-4159.1995.64010424.x

For Review Only

66. Sural-Fehr T, Bongarzone ER. How membrane dysfunction influences neuronal survival pathways in sphingolipid storage disorders. *Journal of Neuroscience Research*. 2016;94(11):1042-1048. doi:10.1002/jnr.23763

67. Rudd AK, Devaraj NK. Traceless synthesis of ceramides in living cells reveals saturation-dependent apoptotic effects. *PNAS*. 2018;115(29):7485-7490. doi:10.1073/pnas.1804266115

68. Stiban J, Perera M. Very long chain ceramides interfere with C16-ceramide-induced channel formation: A plausible mechanism for regulating the initiation of intrinsic apoptosis. *Biochimica et Biophysica Acta (BBA) - Biomembranes*. 2015;1848(2):561-567. doi:10.1016/j.bbamem.2014.11.018

69. Sassa T, Hirayama T, Kihara A. Enzyme Activities of the Ceramide Synthases CERS2–6 Are Regulated by Phosphorylation in the C-terminal Region. *Journal of Biological Chemistry*. 2016;291(14):7477-7487. doi:10.1074/jbc.M115.695858

70. Mizutani Y, Kihara A, Igarashi Y. Mammalian Lass6 and its related family members regulate synthesis of specific ceramides. *Biochem J*. 2005;390(Pt 1):263-271. doi:10.1042/BJ20050291

71. Sassa T, Suto S, Okayasu Y, Kihara A. A shift in sphingolipid composition from C24 to C16 increases susceptibility to apoptosis in HeLa cells. *Biochimica et Biophysica Acta (BBA) - Molecular and Cell Biology of Lipids*. 2012;1821(7):1031-1037. doi:10.1016/j.bbalip.2012.04.008

72. Carmo C, Naia L, Lopes C, Rego AC. Mitochondrial Dysfunction in Huntington's Disease. *Adv Exp Med Biol*. 2018;1049:59-83. doi:10.1007/978-3-319-71779-1_3

73. Ciarlo L, Manganelli V, Matarrese P, et al. Raft-like microdomains play a key role in mitochondrial impairment in lymphoid cells from patients with Huntington's disease. *Journal of Lipid Research*. 2012;53(10):2057-2068. doi:10.1194/jlr.M026062

74. Damiano M, Galvan L, Déglon N, Brouillet E. Mitochondria in Huntington's disease. *Biochimica et Biophysica Acta (BBA) - Molecular Basis of Disease*. 2010;1802(1):52-61. doi:10.1016/j.bbadis.2009.07.012

75. Guedes-Dias P, Pinho BR, Soares TR, de Proença J, Duchen MR, Oliveira JMA. Mitochondrial dynamics and quality control in Huntington's disease. *Neurobiology of Disease*. 2016;90:51-57. doi:10.1016/j.nbd.2015.09.008

76. Kim J, Moody JP, Edgerly CK, et al. Mitochondrial loss, dysfunction and altered dynamics in Huntington's disease. *Hum Mol Genet*. 2010;19(20):3919-3935. doi:10.1093/hmg/ddq306

77. Pickrell AM, Fukui H, Wang X, Pinto M, Moraes CT. The Striatum Is Highly Susceptible to Mitochondrial Oxidative Phosphorylation Dysfunctions. *J Neurosci*. 2011;31(27):9895-9904. doi:10.1523/JNEUROSCI.6223-10.2011

78. Langbehn DR, Stout JC, Gregory S, et al. Association of CAG Repeats With Long-term Progression in Huntington Disease. *JAMA Neurol*. 2019;76(11):1375. doi:10.1001/jamaneurol.2019.2368

For Review Only

79. de Wit NM, Snkhchyan H, den Hoedt S, et al. Altered Sphingolipid Balance in Capillary Cerebral Amyloid Angiopathy. *Journal of Alzheimer's disease : JAD*. 2017;60(3):795-807.

80. Chaibva M, Gao X, Jain P, Campbell WA, Frey SL, Legleiter J. Sphingomyelin and GM1 Influence Huntingtin Binding to, Disruption of, and Aggregation on Lipid Membranes. *ACS Omega*. 2018;3(1):273-285. doi:10.1021/acsomega.7b01472

81. Won J-S, Singh AK, Singh I. Lactosylceramide: a lipid second messenger in neuroinflammatory disease. *Journal of Neurochemistry*. 2007;103(s1):180-191. doi:10.1111/j.1471-4159.2007.04822.x

82. Bodas M, Min T, Vij N. Lactosylceramide-accumulation in lipid-rafts mediate aberrant-autophagy, inflammation and apoptosis in cigarette smoke induced emphysema. *Apoptosis*. 2015;20(5):725-739. doi:10.1007/s10495-015-1098-0

83. Filimoniuk A, Blachnio-Zabielska A, Imierska M, Lebensztejn DM, Daniluk U. Sphingolipid Analysis Indicate Lactosylceramide as a Potential Biomarker of Inflammatory Bowel Disease in Children. *Biomolecules*. 2020;10(7):1083. doi:10.3390/biom10071083

84. Rüb U, Hoche F, Brunt ER, et al. Degeneration of the cerebellum in Huntington's disease (HD): possible relevance for the clinical picture and potential gateway to pathological mechanisms of the disease process. *Brain pathology (Zurich, Switzerland)*. 2013;23(2):165-177. doi:10.1111/j.1750-3639.2012.00629.x

85. Eickhoff SB, Laird AR, Fox PT, Bzdok D, Hensel L. Functional Segregation of the Human Dorsomedial Prefrontal Cortex. *Cerebral Cortex*. 2016;26(1):304-321. doi:10.1093/cercor/bhu250

86. Matsui JT, Vaidya JG, Wassermann D, et al. Prefrontal cortex white matter tracts in prodromal Huntington disease: Prefrontal Cortex WM Tracts in Prodromal HD. *Human Brain Mapping*. 2015;36(10):3717-3732. doi:10.1002/hbm.22835

87. Hancock SE, Friedrich MG, Mitchell TW, Truscott RJW, Else PL. The phospholipid composition of the human entorhinal cortex remains relatively stable over 80 years of adult aging. *GeroScience*. 2017;39(1):73-82. doi:10.1007/s11357-017-9961-2

88. Cabré R, Naudí A, Dominguez-Gonzalez M, et al. Lipid Profile in Human Frontal Cortex Is Sustained Throughout Healthy Adult Life Span to Decay at Advanced Ages. *J Gerontol A Biol Sci Med Sci*. 2018;73(6):703-710. doi:10.1093/gerona/glx164

89. Fennema-Notestine C, Archibald SL, Jacobson MW, et al. In vivo evidence of cerebellar atrophy and cerebral white matter loss in Huntington disease. *Neurology*. 2004;63(6):989-995. doi:10.1212/01.WNL.0000138434.68093.67

90. Kassubek J, Juengling FD, Kioschies T, et al. Topography of cerebral atrophy in early Huntington's disease: a voxel based morphometric MRI study. *Journal of Neurology, Neurosurgery & Psychiatry*. 2004;75(2):213-220. doi:10.1136/jnnp.2002.009019

For Review Only

91. Faria AV, Ratnanather JT, Tward DJ, et al. Linking white matter and deep gray matter alterations in premanifest Huntington disease. *NeuroImage: Clinical*. 2016;11:450-460. doi:10.1016/j.nicl.2016.02.014

92. Rosas HD, Salat DH, Lee SY, et al. Cerebral cortex and the clinical expression of Huntington's disease: complexity and heterogeneity. *Brain*. 2008;131(Pt 4):1057-1068. doi:10.1093/brain/awn025

Figure legends

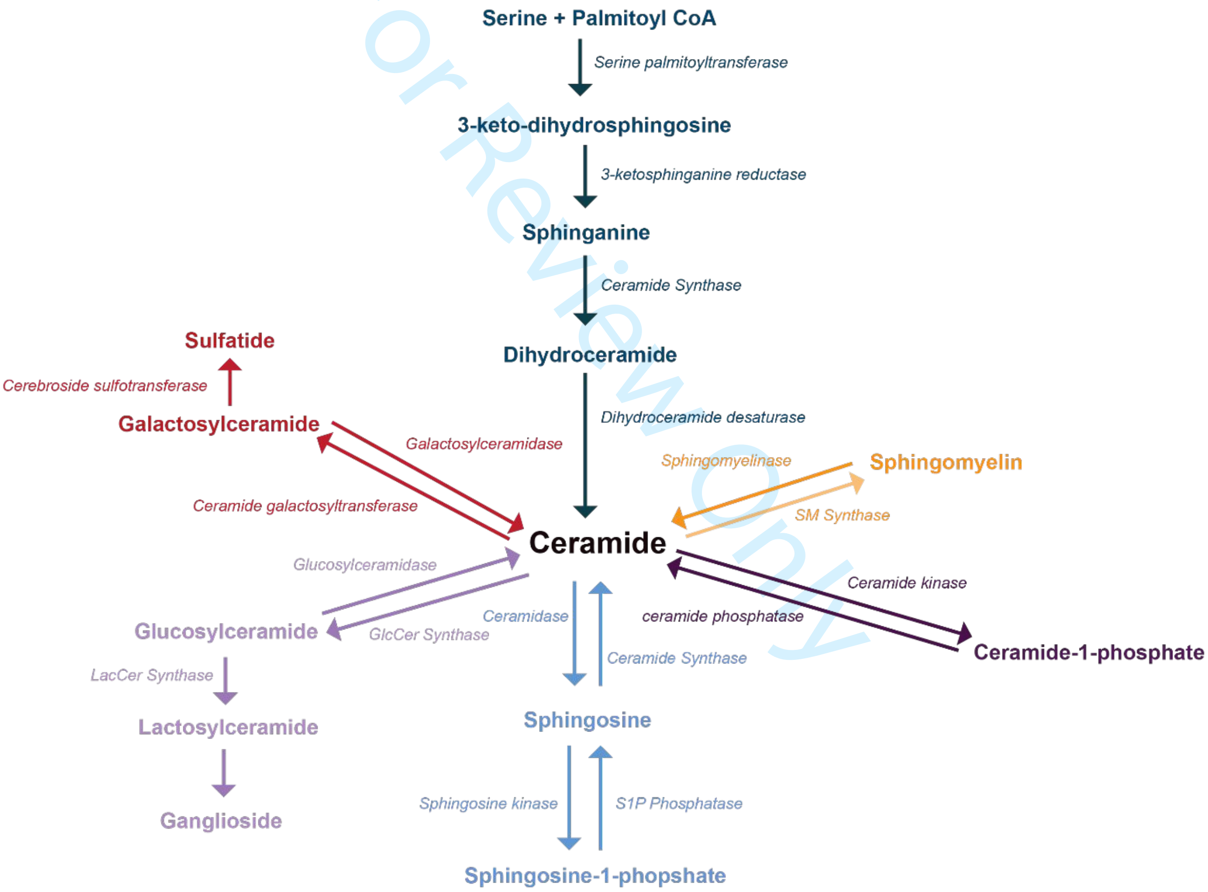

Figure 1 Sphingolipid metabolic pathway. Ceramide is the metabolic hub with synthesis available via the *de novo* pathway (dark blue), via the hydrolysis of sphingomyelin (orange), or through the salvage of more complex sphingolipids (sky blue, lavender, red). Ceramides become glycosphingolipids via either conversion to glucosylceramide (lavender) or galactosylceramide (red). **Abbreviations: GluCer** Glucosylceramide, **LacCer** Lactosylceramide, **S1P** Sphingosine-1-phosphate, **SM** Sphingomyelin.

https://mc.manuscriptcentral.com/braincom

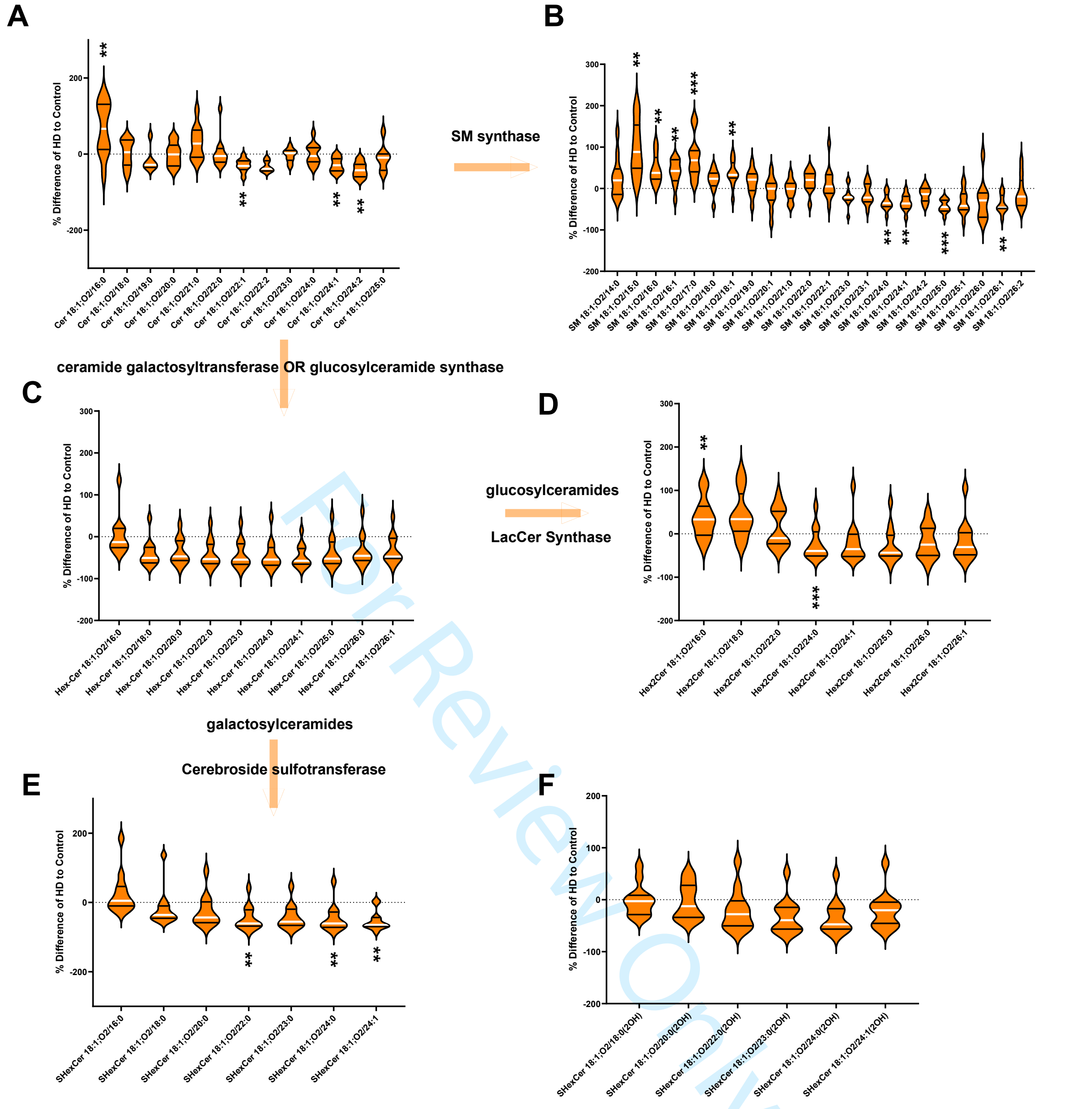

Figure 2 Percentage differences of sphingolipid species in HD subjects (*n* = 12) to controls (*n* = 12) for (A) ceramides, (B) sphingomyelins, (C) monohexosylceramides (glucosylceramide, galactosylceramide), (D) dihexosylceramides (lactosylceramides), (E) sulfatides and (F) *OH*-sulfatide in the caudate. Arrows indicate metabolic pathways. Violin plot displays spread of values, medians (white line) and quartiles. Data was assessed for normality using a D'Agostino Pearson Omnibus test and analysed using an unpaired t-test or Mann Whitney U test where appropriate. **p<0.01, ***p<0.001. **Cer** Ceramide, **HD** Huntington's disease, **SM** Sphingomyelin.

For Review Only

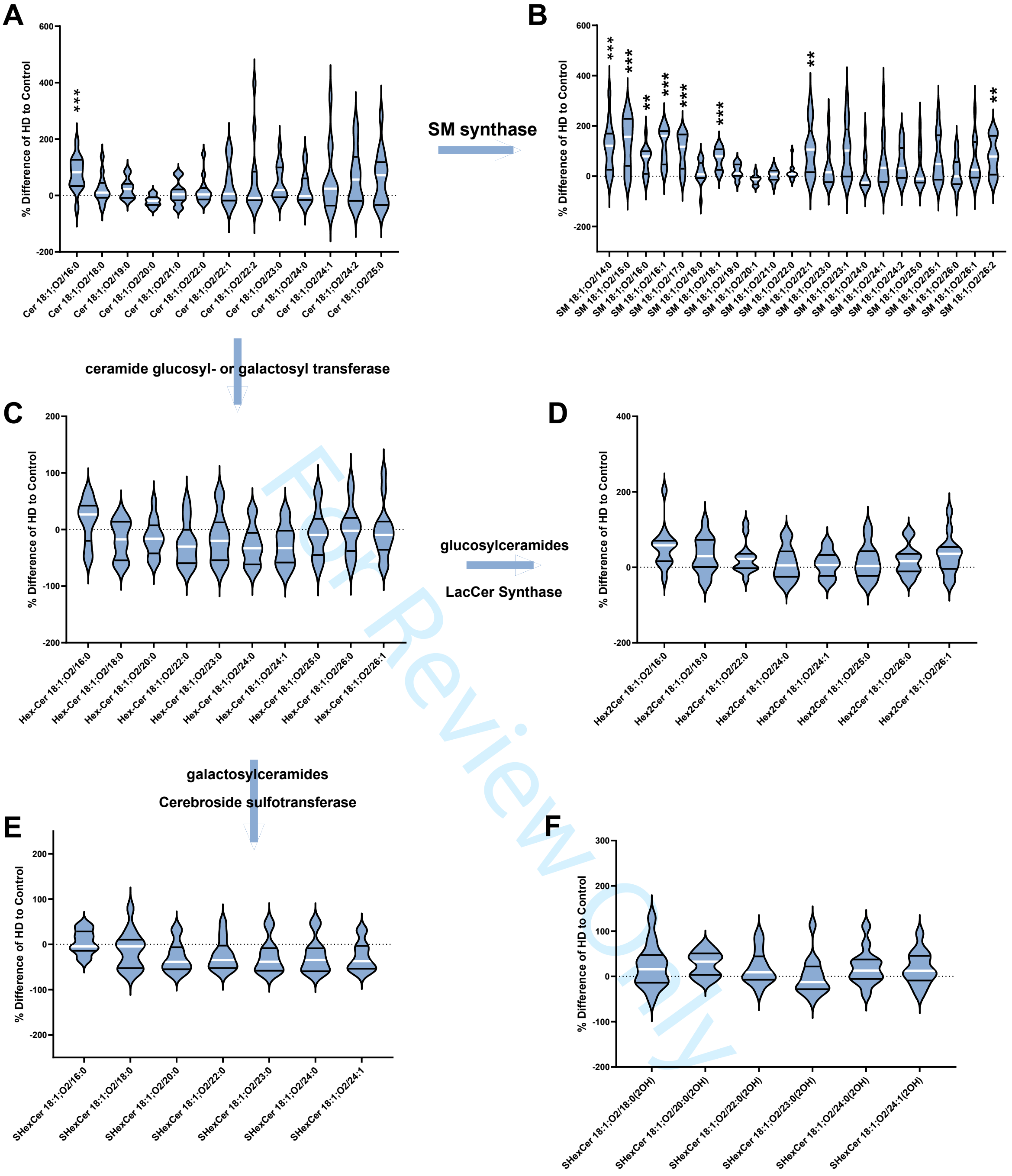

For Review Only

Figure 3 Percentage differences of sphingolipid species in HD (*n* = 13) subjects to controls (*n* = 12) for (A) ceramides, (B) sphingomyelins, (C) monohexosylceramides (glucosylceramide, galactosylceramide), (D) dihexosylceramides (lactosylceramides), (E) sulfatides and (F) hydroxylated sulfatide in the putamen. Arrows indicate metabolic pathways. Violin plot displays spread of values, medians (white line) and quartiles. Data was assessed for normality using a D'Agostino Pearson Omnibus test and analysed using an unpaired t-test or Mann Whitney U test where appropriate. **p<0.01, ***p<0.001. **Cer** Ceramide, **HD** Huntington's disease, **SM** Sphingomyelin, **SHexCer** Sulfatide, **Hex-Cer** Hexosylceramide (glucosylceramide & galactosylceramide), **Hex2Cer** (lactosylceramide).

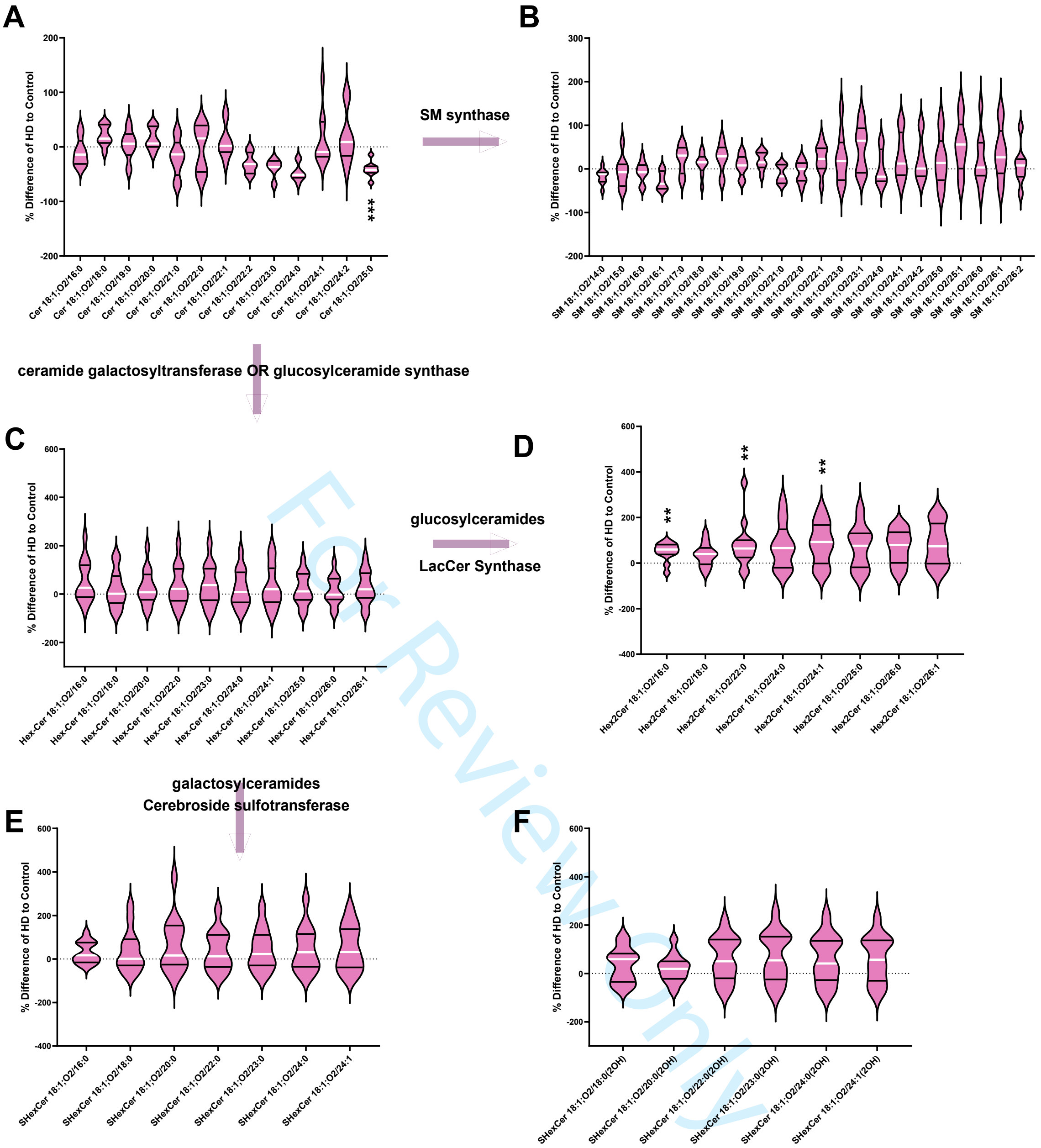

For Review Only

Figure 4 Percentage differences of sphingolipid species in HD (*n* = 13) subjects to controls (*n* = 13) for (A) ceramides, (B) sphingomyelins, (C) monohexosylceramides (glucosylceramide, galactosylceramide), (D) dihexosylceramides (lactosylceramides), (E) sulfatides and (F) *OH*-sulfatide in the cerebellum. Arrows indicate metabolic pathways. Violin plot displays spread of values, medians (white line) and quartiles. Data was assessed for normality using a D'Agostino Pearson Omnibus test and analysed using an unpaired t-test or Mann Whitney U test where appropriate. $**p<0.01$, $***p<0.001$. **Cer** Ceramide, **HD** Huntington's disease, **SM** Sphingomyelin.

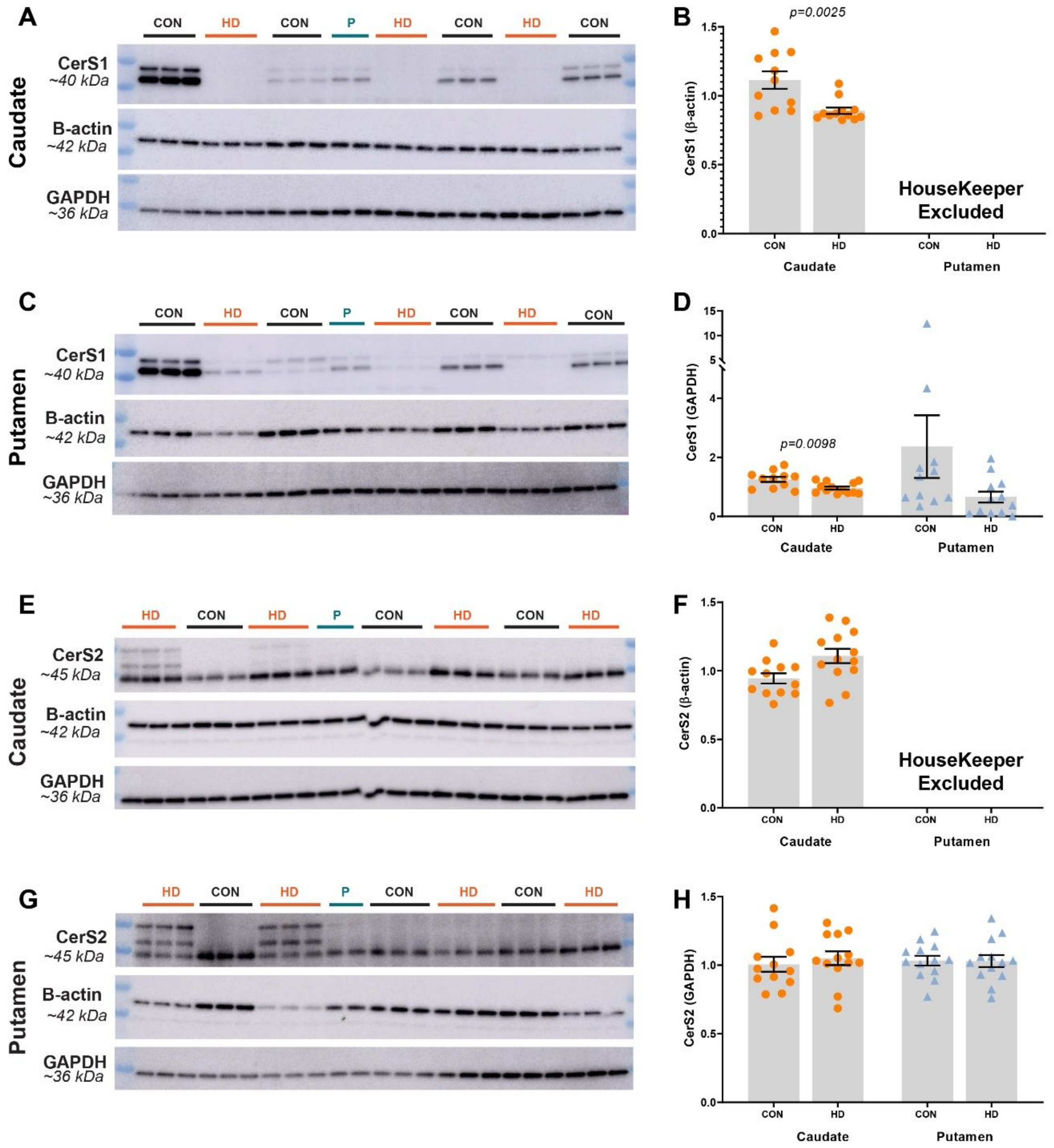

Figure 5 The expression of CerS1 and CerS2 in control and HD striatum. The mean expression of CerS1 between control and HD subjects adjusted by (B) β-actin and (D) GAPDH in striatal subregions. The expression of CerS2 between control and HD subjects (F) β-actin and (H) GAPDH in the same regions. Graphs display the mean expression, the standard error, and the individual subject values. Significant p values are indicated. Representative western blots of CerS1 and CerS2 in the (A, E) caudate and (C, G) putamen with β-actin and GAPDH housekeepers. β-actin was excluded as a housekeeper for putamen samples as the expression was found to be significantly different in HD subjects as compared with controls. Full western blots are provided in **Supplementary Figures S6-S9**. **CerS1** Ceramide Synthase 1, **CerS2** Ceramide Synthase 2, **CON** Control, **GAPDH** Glyceraldehyde 3-phosphate dehydrogenase, **HD** Huntington's Disease, **P** Pool.

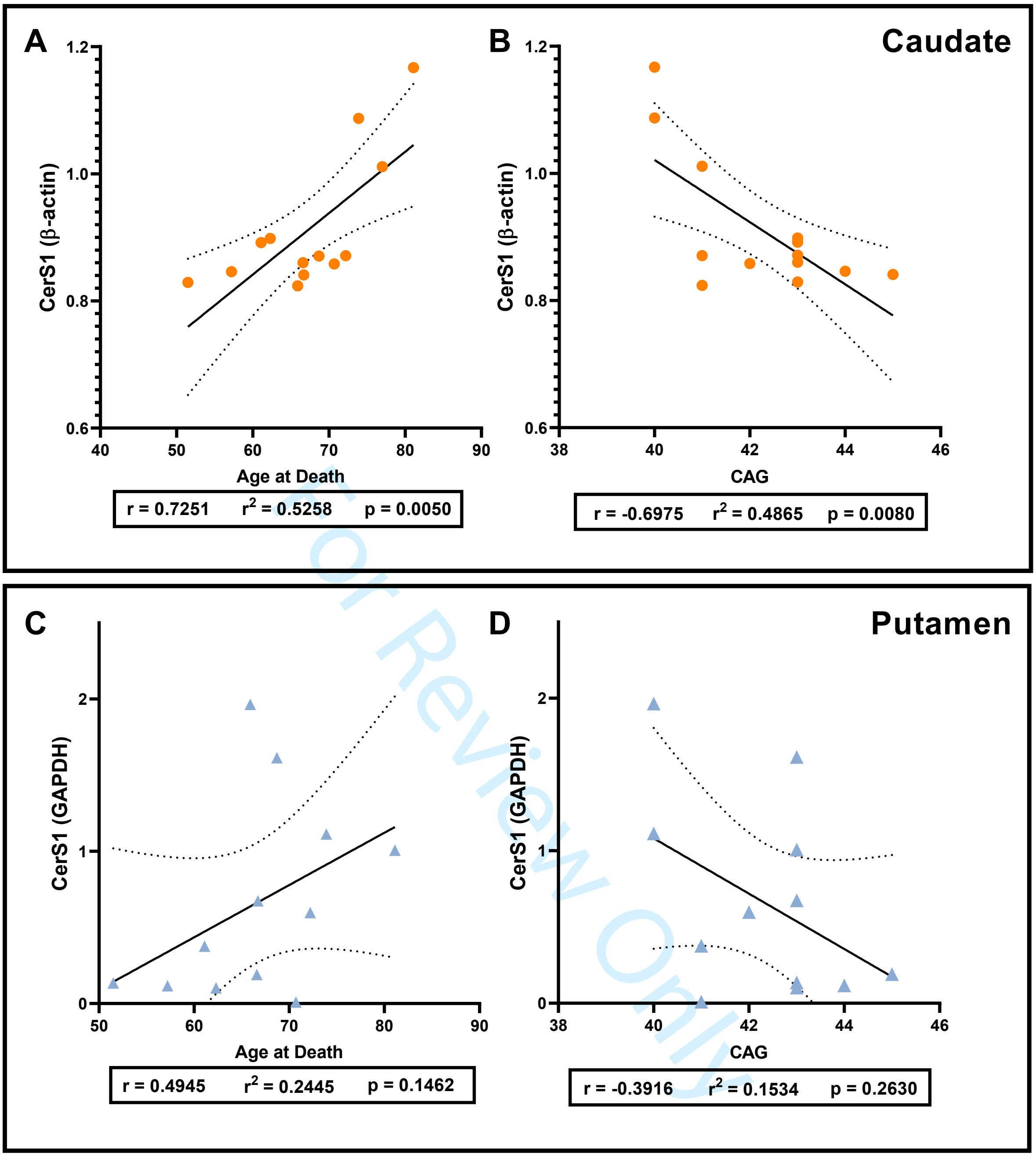

Figure 6 The relationship of CerS1 expression with Age at Death and CAG repeat length in HD subjects. Pearson's correlation analyses were used to determine correlations. Plots indicate the line of best fit (solid black line) with 95% confidence intervals (dotted black lines). CerS1 *vs* Age at Death in (A) HD caudate and (C) HD putamen. CerS1 vs CAG repeat length in (B) HD caudate and (D) HD putamen. Correlation analysis values are provided in **Supplementary Tables S30-31**. **CerS1** Ceramide Synthase 1, **HD** Huntington's disease.

For Review Only

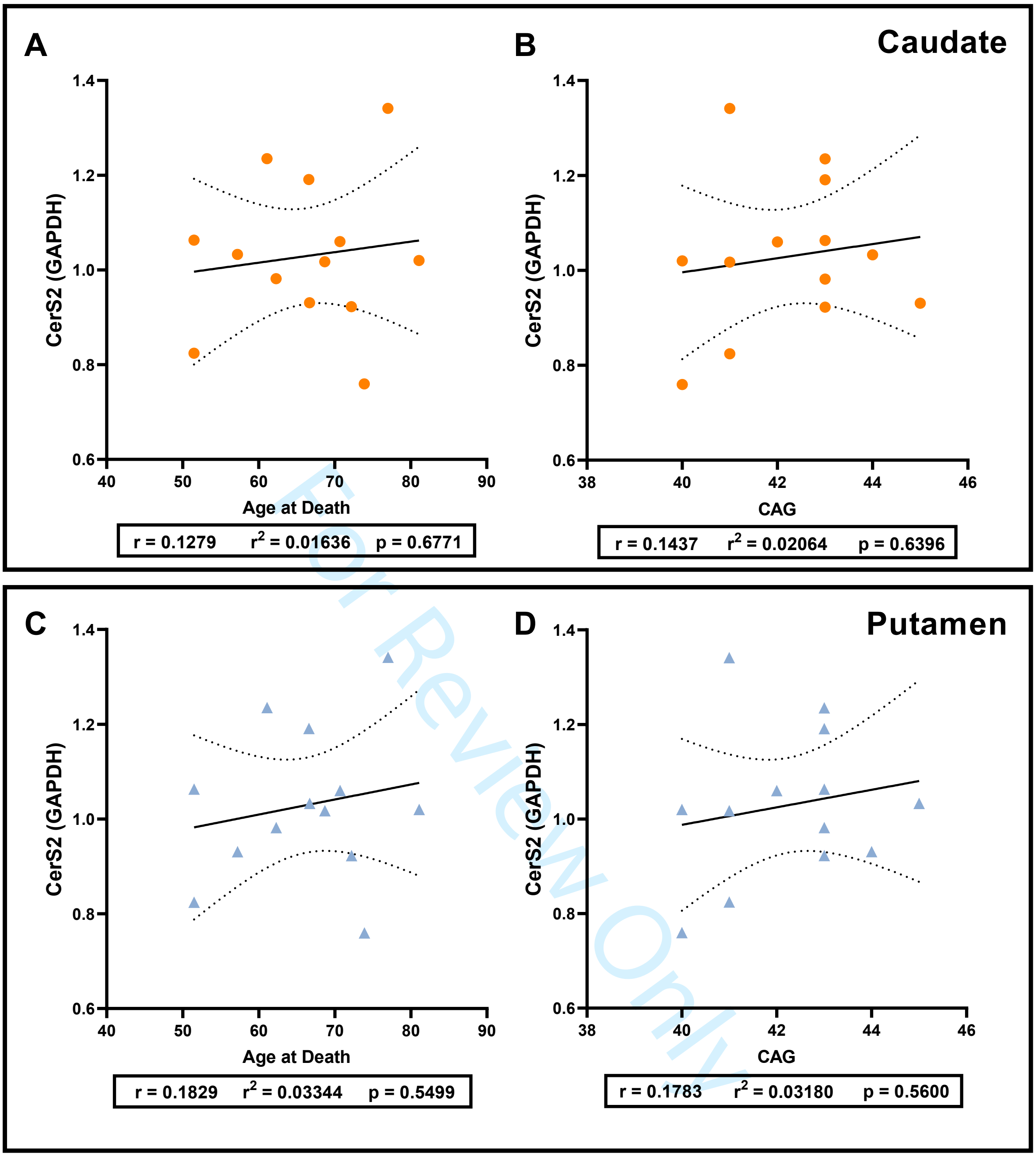

Figure 7 The relationship of CerS2 expression with Age at Death and CAG repeat length in HD subjects. Pearson's correlation analyses were used to determine correlations. Plots indicate the line of best fit (solid black line) with 95% confidence intervals (dotted black lines). CerS2 *vs* Age at Death in (A) HD caudate and (C) HD putamen. CerS2 vs CAG repeat length in (B) HD caudate and (D) HD putamen. Correlation analysis values are provided in **Supplementary Tables S30-32**. **CerS2** Ceramide Synthase 2, **HD** Huntington's disease.

For Review Only

Table 1 Subject Demographics of Post-Mortem Tissue.

Subject	Sex	Age at Death (years)	Post-Mortem Interval (hours)	pH	CAG
CON 1	M	78.3	46.0	6.54	-
CON 2	M	69.1	34.0	6.31	-
CON 3	M	63.9	54.5	6.51	-
CON 4	M	81.0	36.5	6.56	-
CON 5	M	64.1	24.0	6.56	-
CON 6	F	59.0	30.0	6.84	-
CON 7	F	67.3	30.0	6.23	-
CON 8	F	74.8	61.5	6.24	-
CON 9	F	68.3	71.5	6.34	-
CON 10	F	60.4	49.0	6.23	-
CON 11	M	63.9	32.0	6.47	-
CON 12	M	69.4	24.0	6.27	-
CON 13	M	75.6	46.0	6.57	-
Mean (SEM)		68.8 (1.9)	41.5 (4.1)	6.44 (0.05)	-
HD 1	M	77.0	8.5	6.57	41
HD 2	M	68.7	72.0	6.32	41
HD 3	M	61.1	17.0	6.54	43
HD 4	M	81.1	50.5	6.23	40
HD 5	M	66.6	37.0	6.26	43
HD 6	F	57.2	22.0	6.22	44
HD 7	F	66.7	18.5	6.21	45
HD 8	F	72.2	22.0	6.43	44
HD 9	F	70.7	70.0	6.16	42
HD 10	F	51.5	63.5	6.54	43
HD 11	M	62.3	21.5	6.62	43
HD 12	M	65.9	58.0	6.33	41
HD 13	M	73.9	26.5	6.31	40
Mean (SEM)		67.3 (2.2)	37.5 (6.2)	6.36 (0.04)	42.31 (0.44)

Sex, Age at Death, Post-Mortem Interval, pH and Vonsattel pathological grade were provided by the Victorian Brain Bank. CAG repeat lengths were determined by the Victorian Clinical Genetics Service. CAG repeat lengths are specific to the HD causing allele (>39 CAG) only and therefore controls are not included. **Abbreviations: CON** Control, **HD** Huntington's disease, **SEM** Standard Error of Mean.

For Review Only

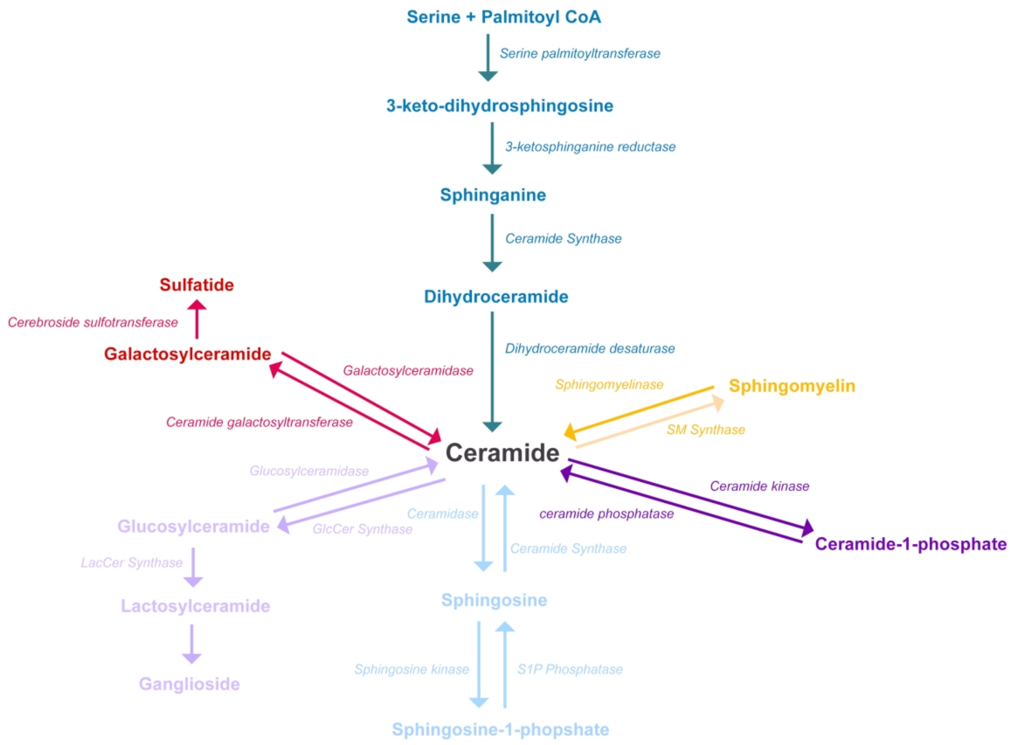

Figure 1 Sphingolipid metabolic pathway. Ceramide is the metabolic hub with synthesis available via the de novo pathway (dark blue), via the hydrolysis of sphingomyelin (orange), or through the salvage of more complex sphingolipids (sky blue, lavender, red). Ceramides become glycosphingolipids via either conversion to glucosylceramide (lavender) or galactosylceramide (red). Abbreviations: GluCer Glucosylceramide, LacCer Lactosylceramide, S1P Sphingosine-1-phosphate, SM Sphingomyelin.

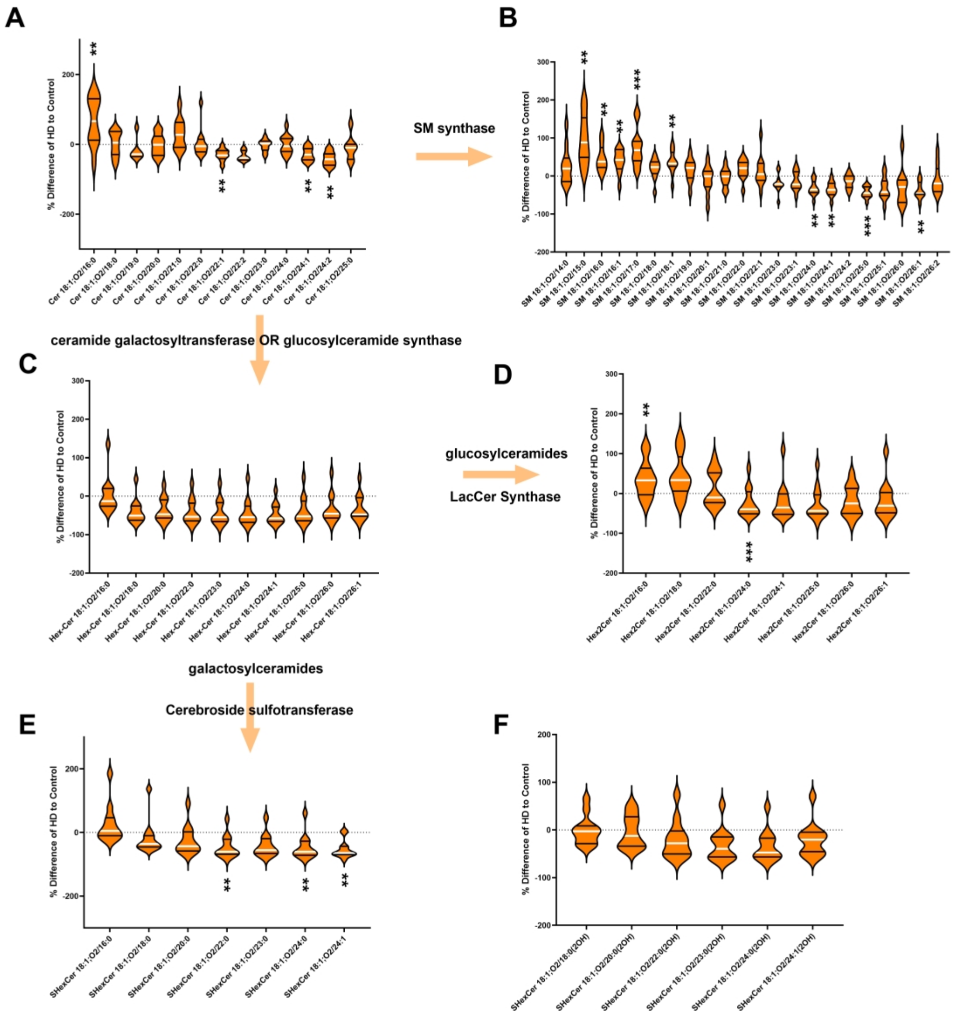

Figure 2 Percentage differences of sphingolipid species in HD subjects (n = 12) to controls (n = 12) for (A) ceramides, (B) sphingomyelins, (C) monohexosylceramides (glucosylceramide, galactosylceramide), (D) dihexosylceramides (lactosylceramides), (E) sulfatides and (F) OH-sulfatide in the caudate. Arrows indicate metabolic pathways. Violin plot displays spread of values, medians (white line) and quartiles. Data was assessed for normality using a D'Agostino Pearson Omnibus test and analysed using an unpaired t-test or Mann Whitney U test where appropriate. $^{**}p<0.01$, $^{***}p<0.001$. Cer Ceramide, HD Huntington's disease, SM Sphingomyelin.

203x214mm (600 x 600 DPI)

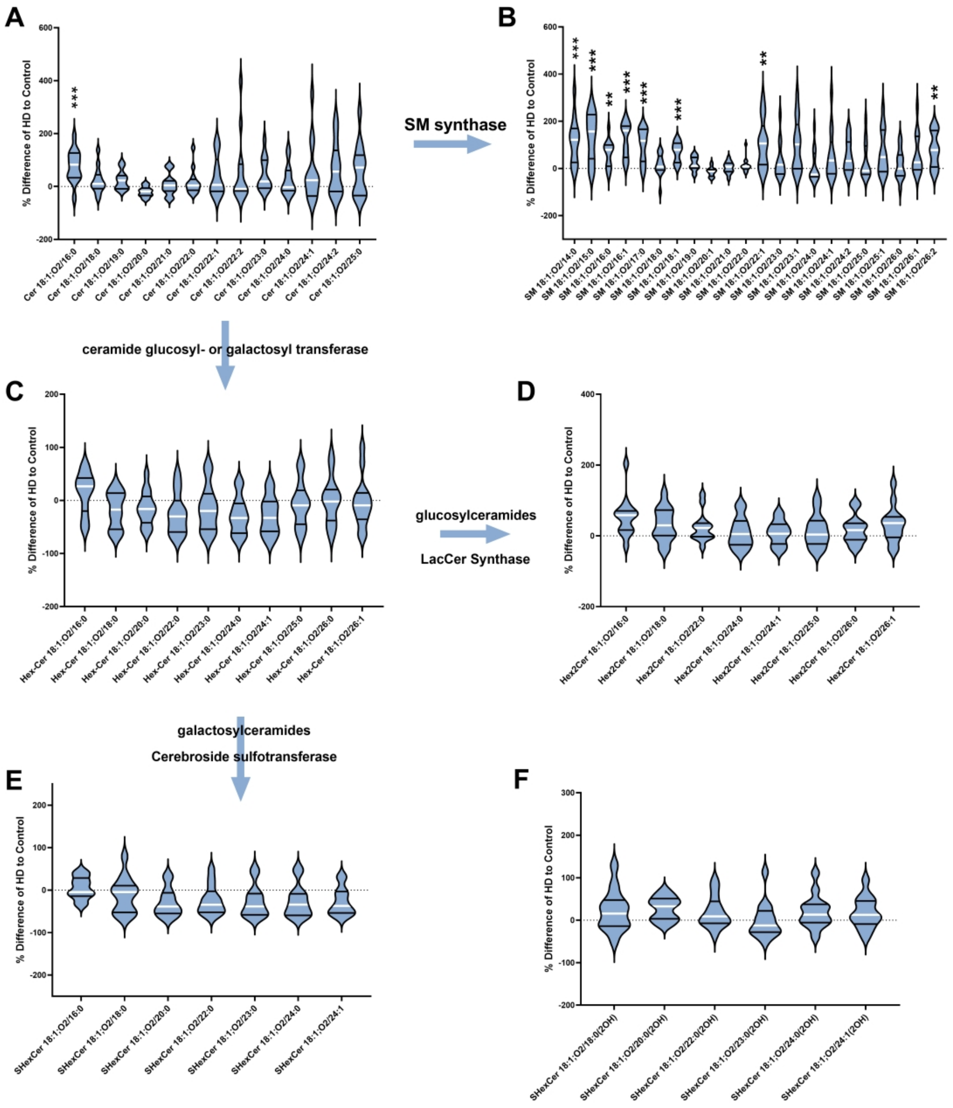

Figure 3 Percentage differences of sphingolipid species in HD (n = 13) subjects to controls (n = 12) for (A) ceramides, (B) sphingomyelins, (C) monohexosylceramides (glucosylceramide, galactosylceramide), (D) dihexosylceramides (lactosylceramides), (E) sulfatides and (F) hydroxylated sulfatide in the putamen. Arrows indicate metabolic pathways. Violin plot displays spread of values, medians (white line) and quartiles. Data was assessed for normality using a D'Agostino Pearson Omnibus test and analysed using an unpaired t-test or Mann Whitney U test where appropriate. **p<0.01, ***p<0.001. Cer Ceramide, HD Huntington's disease, SM Sphingomyelin, SHexCer Sulfatide, Hex-Cer Hexosylceramide (glucosylceramide & galactosylceramide), Hex2Cer (lactosylceramide).

206x235mm (600 x 600 DPI)

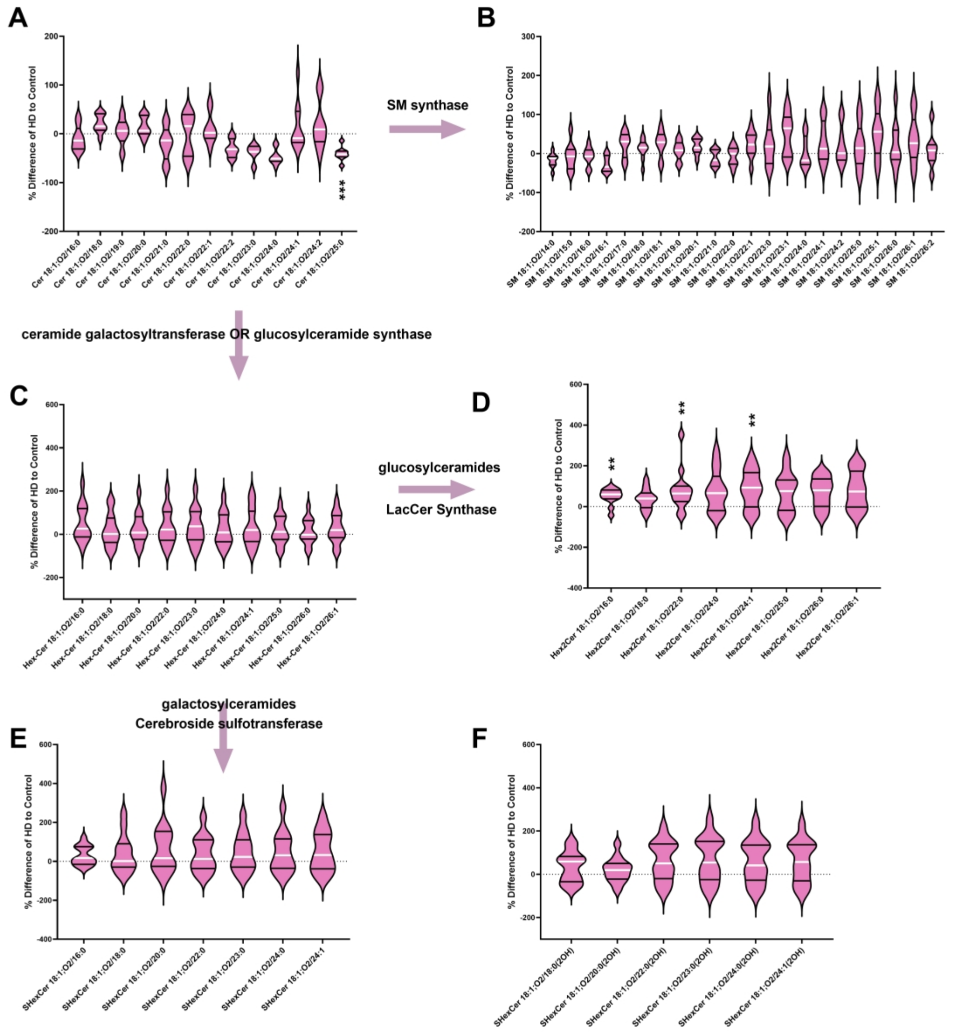

Figure 4 Percentage differences of sphingolipid species in HD (n = 13) subjects to controls (n = 13) for (A) ceramides, (B) sphingomyelins, (C) monohexosylceramides (glucosylceramide, galactosylceramide), (D) dihexosylceramides (lactosylceramides), (E) sulfatides and (F) OH-sulfatide in the cerebellum. Arrows indicate metabolic pathways. Violin plot displays spread of values, medians (white line) and quartiles. Data was assessed for normality using a D’Agostino Pearson Omnibus test and analysed using an unpaired t-test or Mann Whitney U test where appropriate. $^{**}p<0.01$, $^{***}p<0.001$. Cer Ceramide, HD Huntington’s disease, SM Sphingomyelin.

204x218mm (600 x 600 DPI)

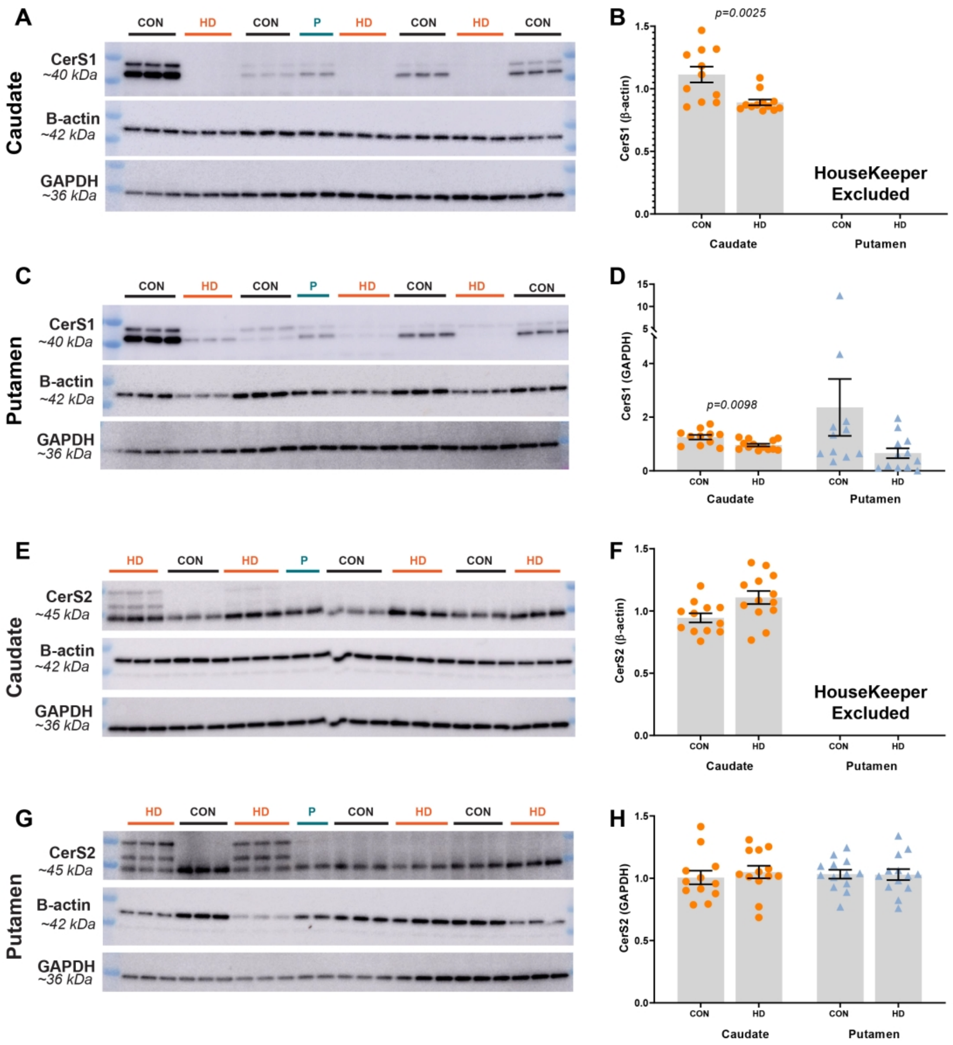

Figure 5 The expression of CerS1 and CerS2 in control and HD striatum. The mean expression of CerS1 between control and HD subjects adjusted by (B) β-actin and (D) GAPDH in striatal subregions. The expression of CerS2 between control and HD subjects (F) β-actin and (H) GAPDH in the same regions. Graphs display the mean expression, the standard error, and the individual subject values. Significant p values are indicated. Representative western blots of CerS1 and CerS2 in the (A, E) caudate and (C, G) putamen with β-actin and GAPDH housekeepers. β-actin was excluded as a housekeeper for putamen samples as the expression was found to be significantly different in HD subjects as compared with controls. Full western blots are provided in Supplementary Figures S6-S9. CerS1 Ceramide Synthase 1, CerS2 Ceramide Synthase 2, CON Control, GAPDH Glyceraldehyde 3-phosphate dehydrogenase, HD Huntington's Disease, P Pool.

240x261mm (300 x 300 DPI)

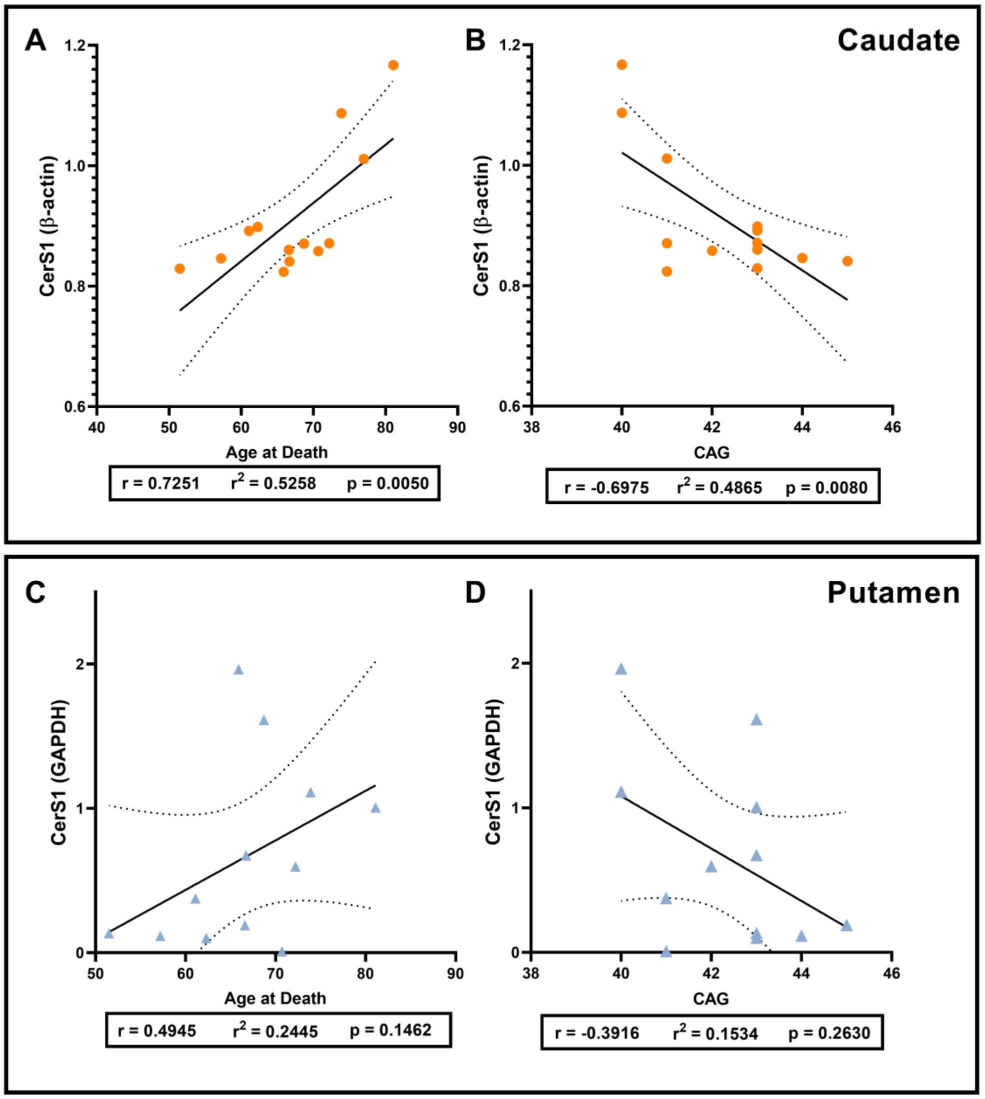

Figure 6 The relationship of CerS1 expression with Age at Death and CAG repeat length in HD subjects. Pearson's correlation analyses were used to determine correlations. Plots indicate the line of best fit (solid black line) with 95% confidence intervals (dotted black lines). CerS1 vs Age at Death in (A) HD caudate and (C) HD putamen. CerS1 vs CAG repeat length in (B) HD caudate and (D) HD putamen. Correlation analysis values are provided in Supplementary Tables S30-31. CerS1 Ceramide Synthase 1, HD Huntington's disease.

185x205mm (600 x 600 DPI)

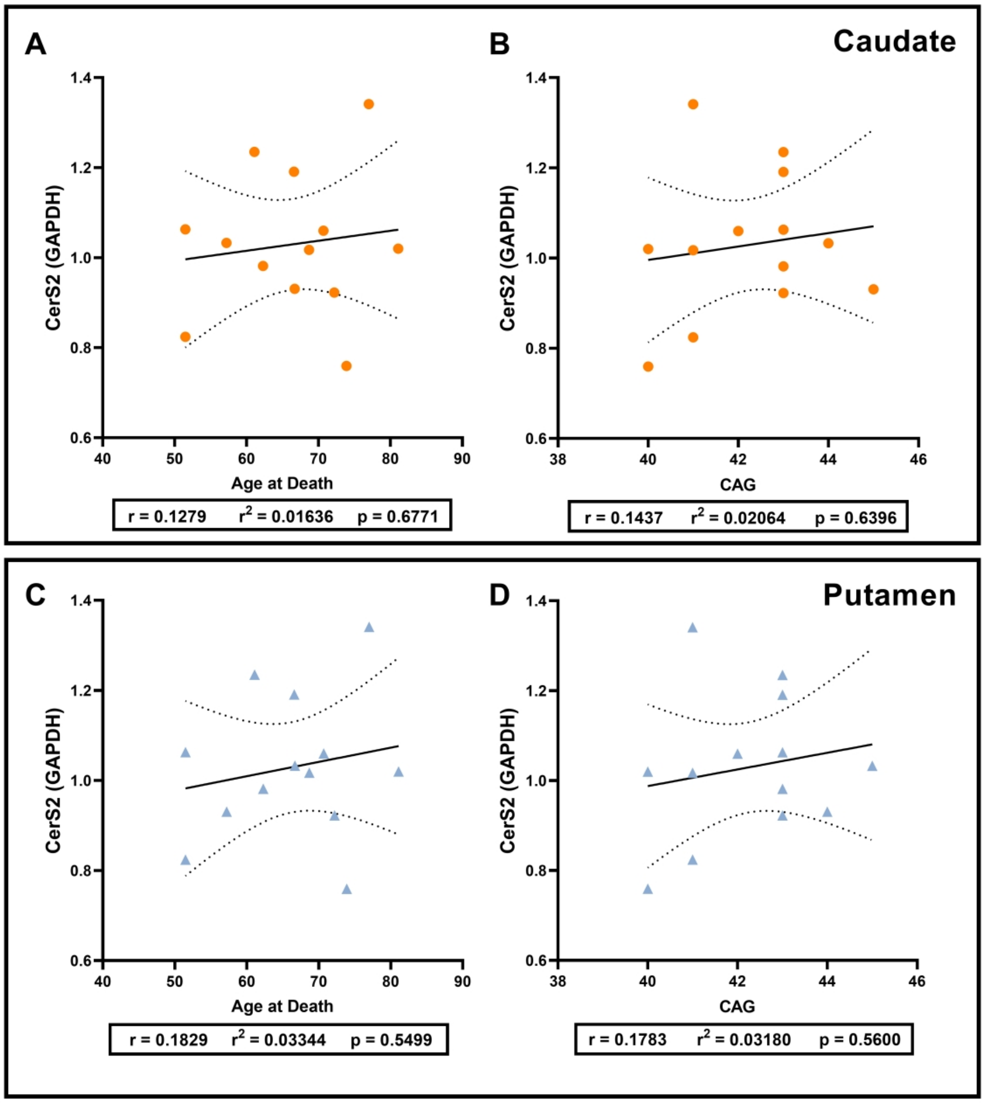

Figure 7 The relationship of CerS2 expression with Age at Death and CAG repeat length in HD subjects. Pearson's correlation analyses were used to determine correlations. Plots indicate the line of best fit (solid black line) with 95% confidence intervals (dotted black lines). CerS2 vs Age at Death in (A) HD caudate and (C) HD putamen. CerS2 vs CAG repeat length in (B) HD caudate and (D) HD putamen. Correlation analysis values are provided in Supplementary Tables S30-32. CerS2 Ceramide Synthase 2, HD Huntington's disease.

184x206mm (600 x 600 DPI)

Supplementary Material (Figures)

The Long and the Short of Huntington's Disease: How the Sphingolipid Profile is Shifted in the Caudate of Advanced Clinical Cases

Gabrielle R. Phillips[1,2,3], Jennifer T. Saville[4], Sarah E. Hancock[5], Simon HJ. Brown[3,6], Andrew M. Jenner[7], Catriona McLean[8], Maria Fuller[4,9], Kelly A. Newell[1,2,3], Todd Mitchell[1,2,3*]

RUNNING TITLE

Chain length alterations to sphingolipids in Huntington's

AFFLIATIONS

[1]Illawarra Health and Medical Research Institute, Wollongong, 2522, NSW, Australia

[2]School of Medicine, University of Wollongong, Wollongong, 2522, NSW, Australia

[3]Molecular Horizons, University of Wollongong, Wollongong, 2522, NSW, Australia

[4]Genetics and Molecular Pathology, SA Pathology at Women's and Children's Hospital, North Adelaide, 5006, SA, Australia

[5]School of Medical Sciences, University of New South Wales, Sydney, 2052, NSW, Australia

[6]School of Chemistry and Molecular Biosciences, University of Wollongong, Wollongong, 2522, NSW, Australia

[7]Bioanalytical Mass Spectrometry Facility, Mark Wainwright Analytical Centre, University of New South Wales, Sydney, 2052, NSW, Australia

[8]Department of Anatomical Pathology, Alfred Health and Florey Neuroscience, Parkville, 3052, VIC, Australia

[9]Adelaide Medical School, University of Adelaide, Adelaide, 5000, SA, Australia

For Review Only

Supplementary Materials S1 Additional Banding of CerS2 in Western Blots

Supplementary bands were identified above the primary CerS2 band (~40 kDa) in HD samples only. To further investigate these bands, two additional experiments were conducted. The first experiment was to check the specificity of the CerS2 antibody using a human CerS2 knockout cell lysate (HEK293T, ab258814, Abcam) as a negative control and a wild type human cell lysate (HEK293T, ab258814, Abcam) as a positive control. All cell and post-mortem samples were loaded at 12.5 µg protein. The knockout blot (**Figure S1a**) supported the specificity of CerS2, showing the presence of CerS2 in wild type cells and the absence in CerS2 knockout cells at or above 40 kDa (**Figure S1b**). The additional banding below 40 kDa appeared in the knockout cells and is therefore unlikely to be a related CerS2 protein and was therefore excluded from the analysis. **Figure S1a** shows the two additional bands which appear only in HD subjects and have a clearer expression in the HD putamen samples. The second experiment was to examine if the reduction of the sample with β-mercaptoethanol would eliminate the additional banding via the breakage of disulphide bonds. HD and control samples were duplicated, with one set reduced with heat (70°C) and β-mercaptoethanol, and the other not. Samples were loaded at 12.5 µg protein. No difference in additional banding above CerS2 primary band was found between the reduced and non-reduced samples indicating their presence is not due to disulphide bonding (**Figure S1b**).

For Review Only

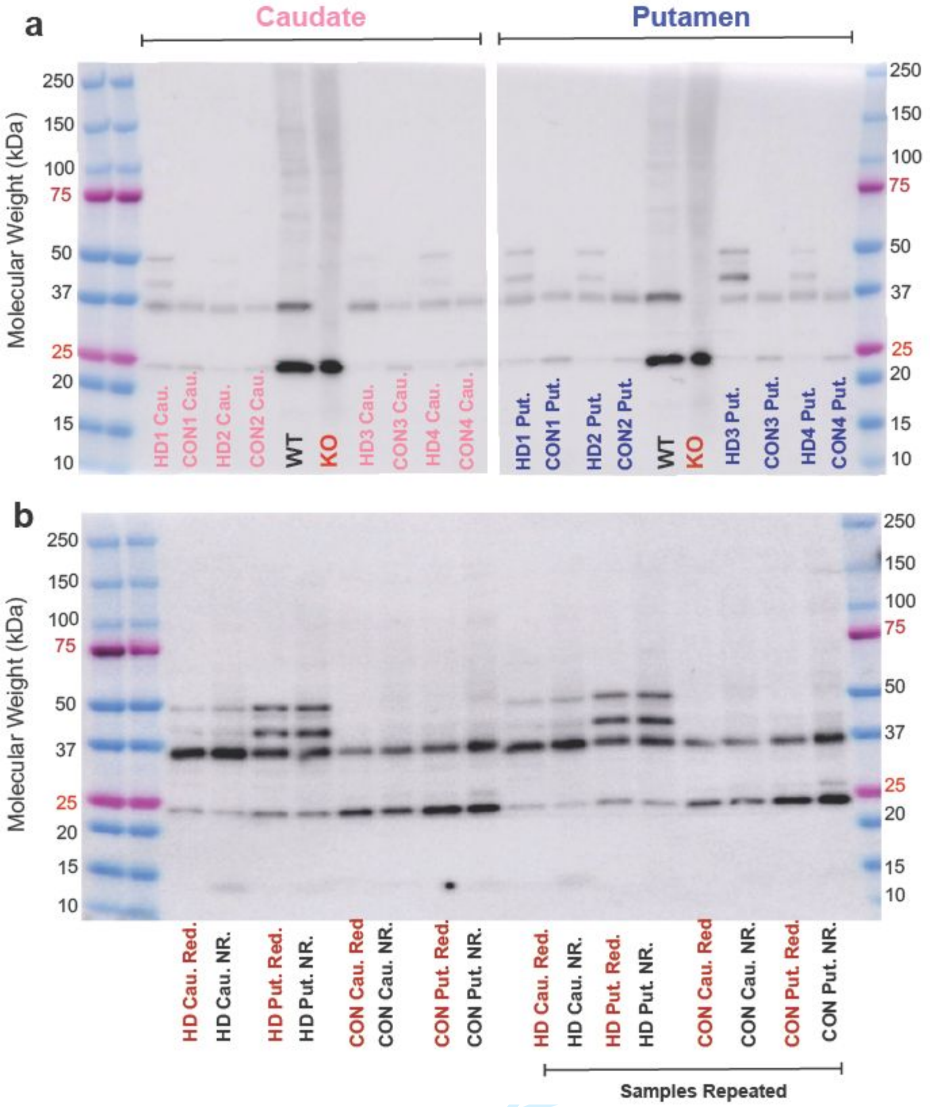

Supplementary Figure S1 CerS2 Experimental Western Blots (a) CerS2 Knockout Cell Blot. HD and Control samples from the caudate and putamen were loaded alongside CerS2 Knockout and Wild Type Cell Human Lines to check for the specificity of the CerS2 antibody. Samples were loaded at 12.5 µg protein. Knockout cells did not show bands at the molecular weight of CerS2. (b) CerS2 Reduction Blot. To further investigate additional banding of CerS2, samples were both reduced and not-reduced with β-mercaptoethanol to determine if disulphide bonds were present. HD and Control samples from the caudate and putamen were loaded at 12.5 µg in duplicate. Neither condition resulted in the disappearance of additional banding. **CerS2** Ceramide Synthase 2, **CON** Control, **HD** Huntington's Disease, **KO** Knockout, **WT** Wild Type.

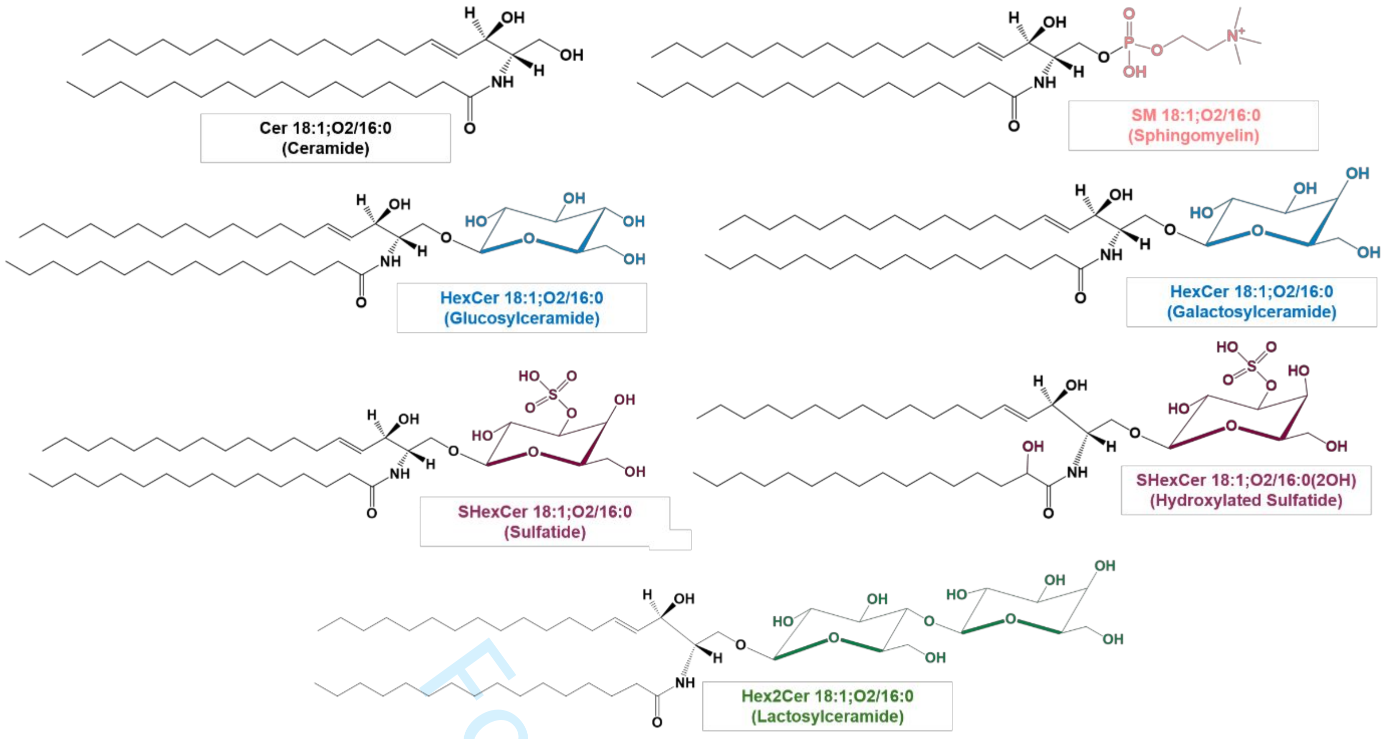

Supplementary Figure S2 Chemical structure of sphingolipid and glycosphingolipid classes analysed. All lipids are shown to have an 18:1 sphingosine back bone and a 16:0 fatty acyl chain. Structures shown are ceramide (black), sphingomyelin (peach), glucosylceramide and galactosylceramide (monohexosylceramides; blue), sulfatide and hydroxylated sulfatide (magenta), and lactosylceramide (dihexosylceramide, green).

For Review Only

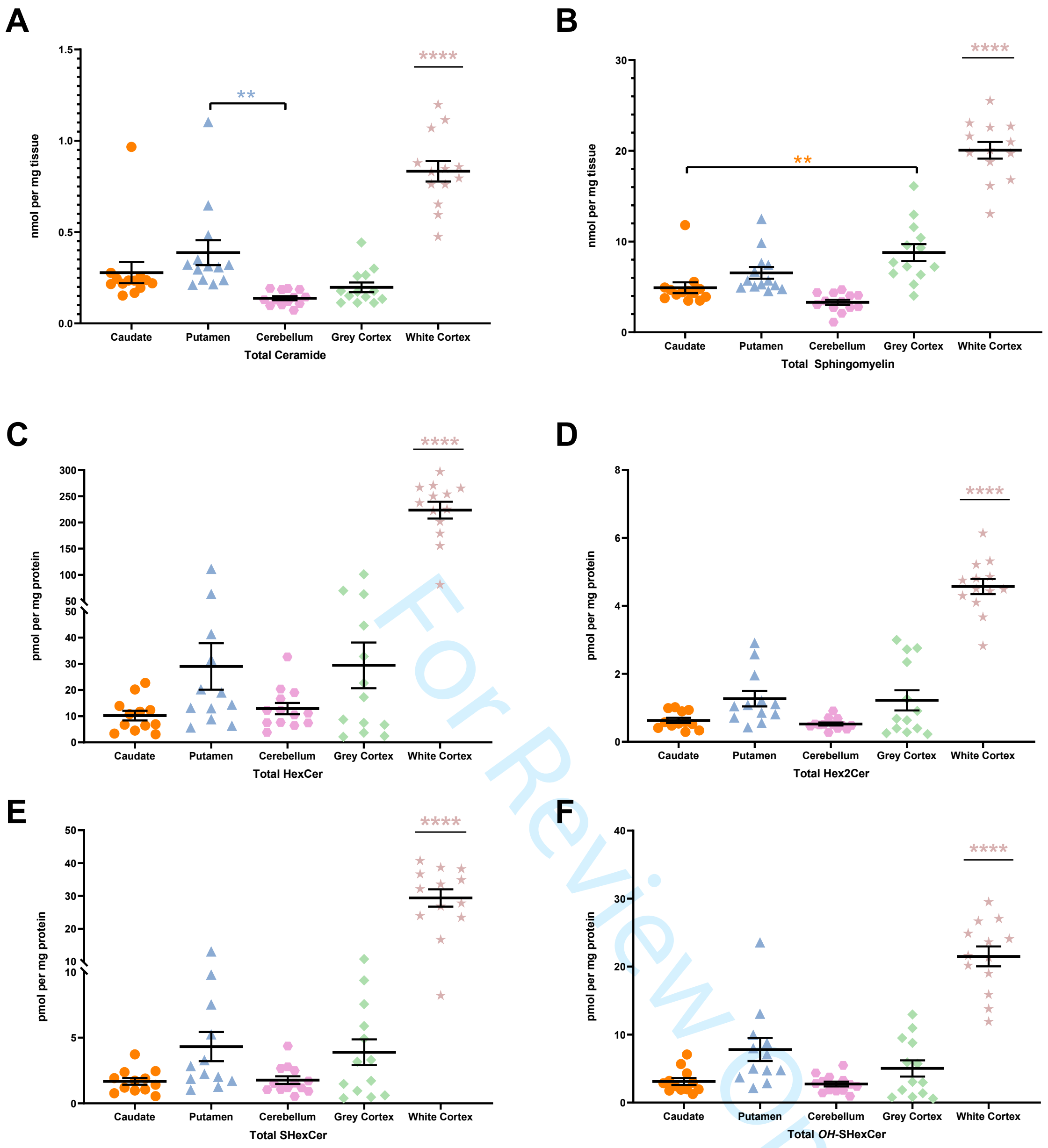

For Review Only

Supplementary Figure S3 Total concentrations of (A) ceramides, (B) sphingomyelin, (C) HexCer, (D) Hex2Cer, (E) SHexCer and (F) *OH*-SHexCer in control regions (*n* = 13). Bars show mean ± SEM in nmol lipid per mg tissue (A, B) and pmol lipid per mg protein (C, D, E and F). A one-way ANOVA with a Tukey's test was used to identify differences in lipids between regions. Indicated significance above column indicates a significant difference to all other regions. Bars are used to indicate differences between the two regions. **$p<0.01$, ***$p<0.001$, ****$p<0.0001$.

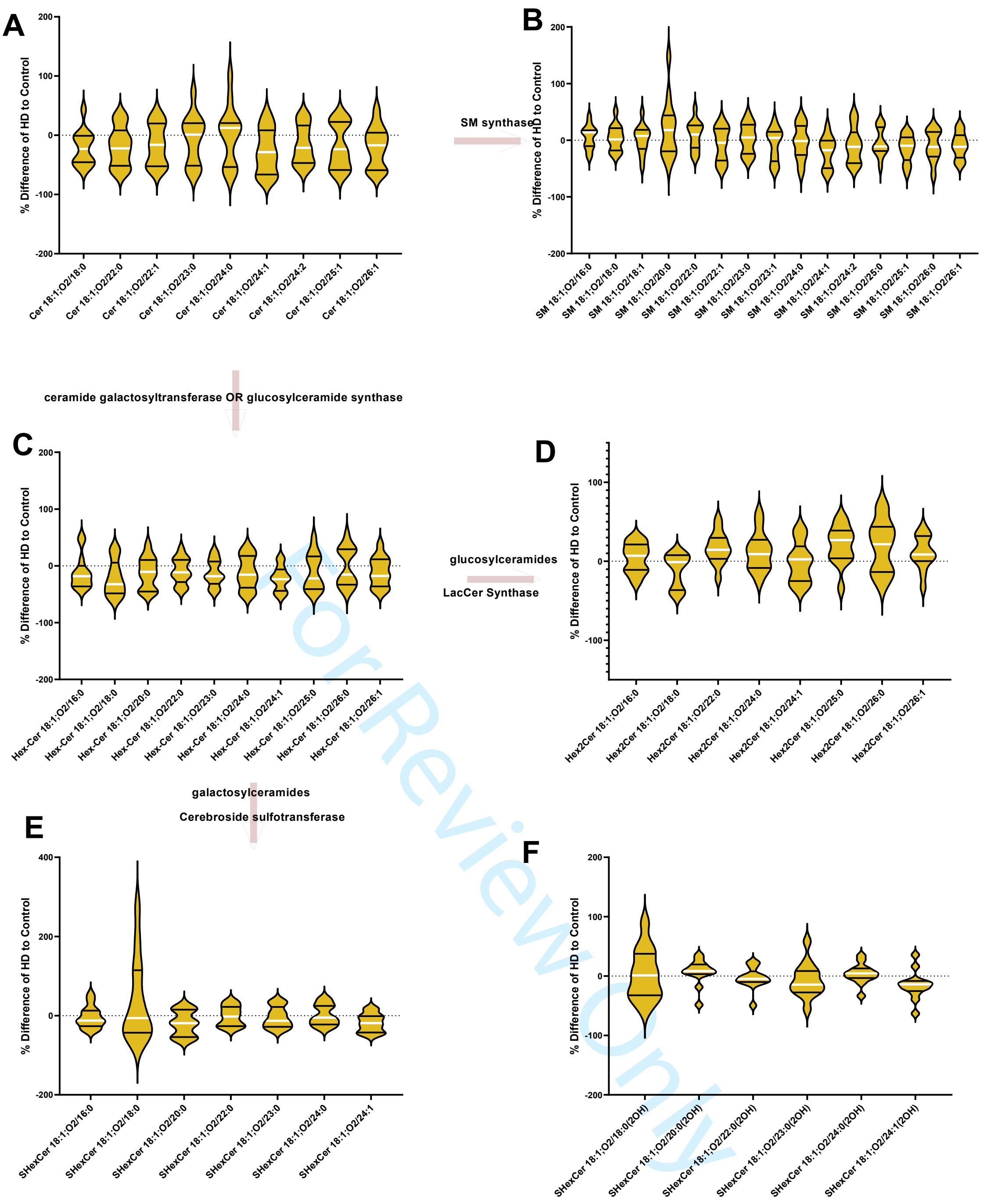

For Review Only

Supplementary Figure S4 Percentage differences of sphingolipid species in HD subjects to controls for (A) ceramides, (B) sphingomyelins, (C) monohexosylceramides, (D) dihexosylceramides, (E) sulfatides and (F) OH-Sulfatide in the white dmPFC. Arrows indicate metabolic pathways. Violin plot displays spread of values, medians (white line) and quartiles. Data was assessed for normality using a D'Agostino Pearson Omnibus test and analysed using an unpaired t-test or Mann Whitney U test where appropriate. Means, SEM, SD, and exact p values are available in **Supplementary Tables S18-S22**. **Cer** Ceramide, **HD** Huntington's disease, **LacCer** Lactosylceramide, **SM** Sphingomyelin.

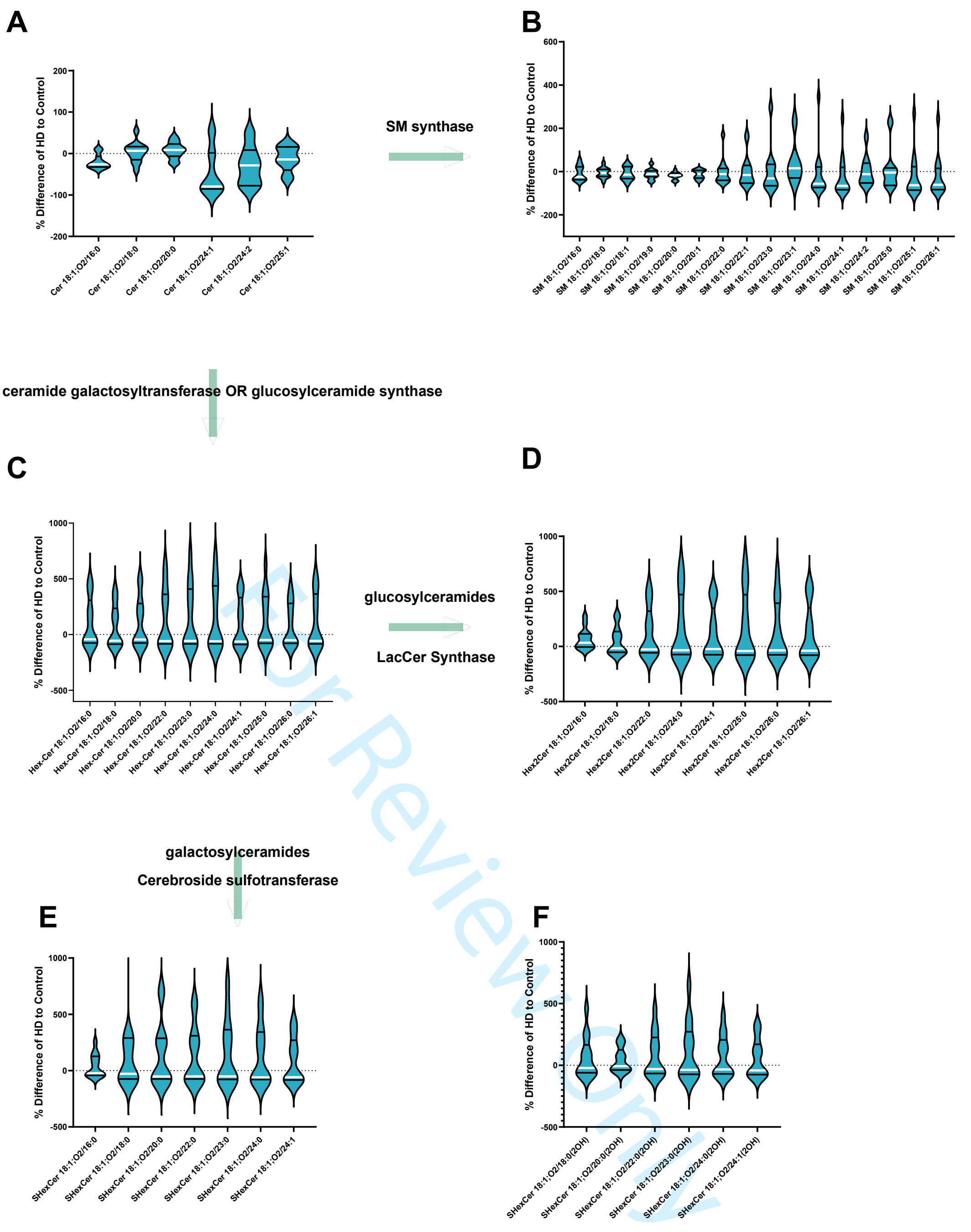

For Review Only

Supplementary Figure S5 Percentage differences of sphingolipid species in HD subjects to controls for (A) ceramides, (B) sphingomyelins, (C) monohexosylceramides, (D) dihexosylceramides, (E) sulfatides and (F) OH-sulfatide in the grey dmPFC. Arrows indicate metabolic pathways. Violin plot displays spread of values, medians (white line) and quartiles. Data was assessed for normality using a D'Agostino Pearson Omnibus test and analysed using an unpaired t-test or Mann Whitney U test where appropriate. Means, SEM, SD, and exact p values are available in **Supplementary Tables S23-S27**. **Cer** Ceramide, **HD** Huntington's disease, **LacCer** Lactosylceramide, **SM** Sphingomyelin.

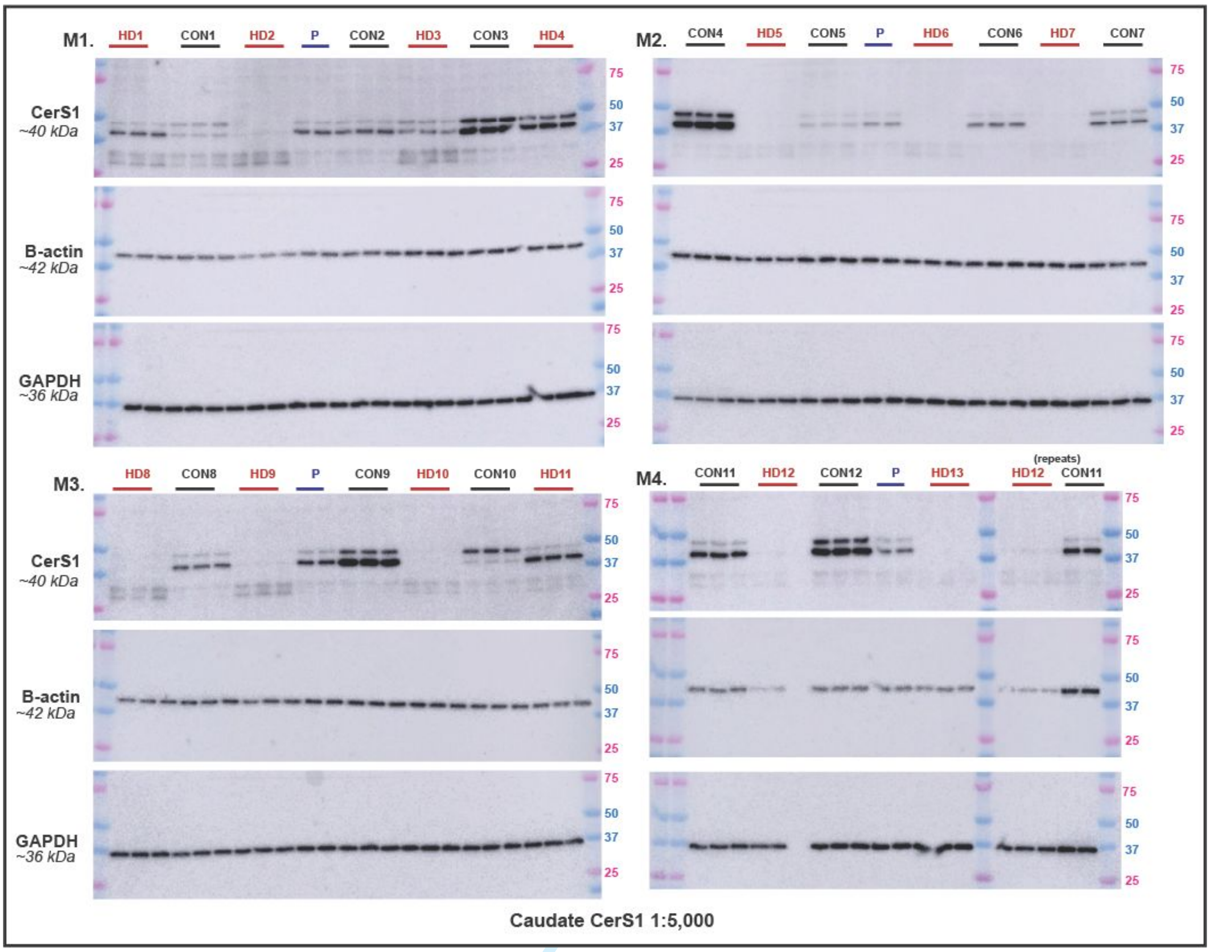

Supplementary Figure S6 Western Blot of CerS1 (1:5,000; ab131169 [Recombinant]) in Control and HD Caudate. Membranes have been cut to show 25-75 kDa. Western blot of β-actin (1:100,000; MAB1501) and GAPDH (1:50,000; Rb659-060908-WS) have been included below their respective CerS1 membranes. Samples were loaded at 10 µg protein in triplicate. Values were averaged and adjusted for Pool samples and to housekeepers. **CerS1** Ceramide Synthase 1, **CON** Control, **GAPDH** Glyceraldehyde 3-phosphate dehydrogenase, **HD** Huntington's Disease, **P** Pool, **TBST** Tris-Buffered Saline with Tween 20.

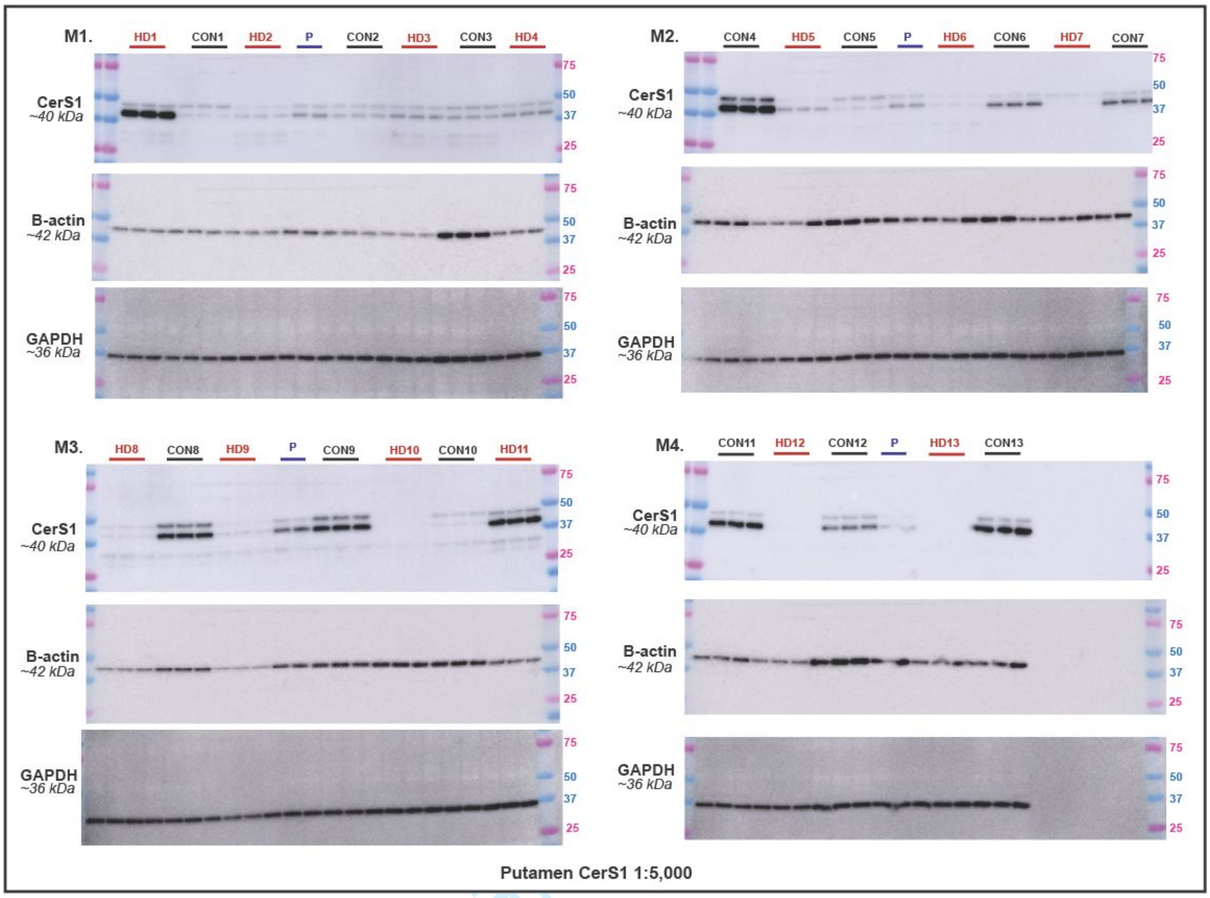

Supplementary Figure S7 Western Blot of CerS1 (1:5,000; ab131169 [Recombinant]) in Control and HD Putamen. Membranes have been cut to show 25-75 kDa. Western blot of β-actin (1:100,000; MAB1501) and GAPDH (1:50,000; Rb659-060908-WS) have been included below their respective CerS1 membranes. Samples were loaded at 10 µg protein in triplicate. Values were averaged and adjusted for Pool samples and to housekeepers. M1-M4 Indicates Membrane Number. **CerS1** Ceramide Synthase 1, **CON** Control, **GAPDH** Glyceraldehyde 3-phosphate dehydrogenase, **HD** Huntington's Disease, **P** Pool, **TBST** Tris-Buffered Saline with Tween 20.

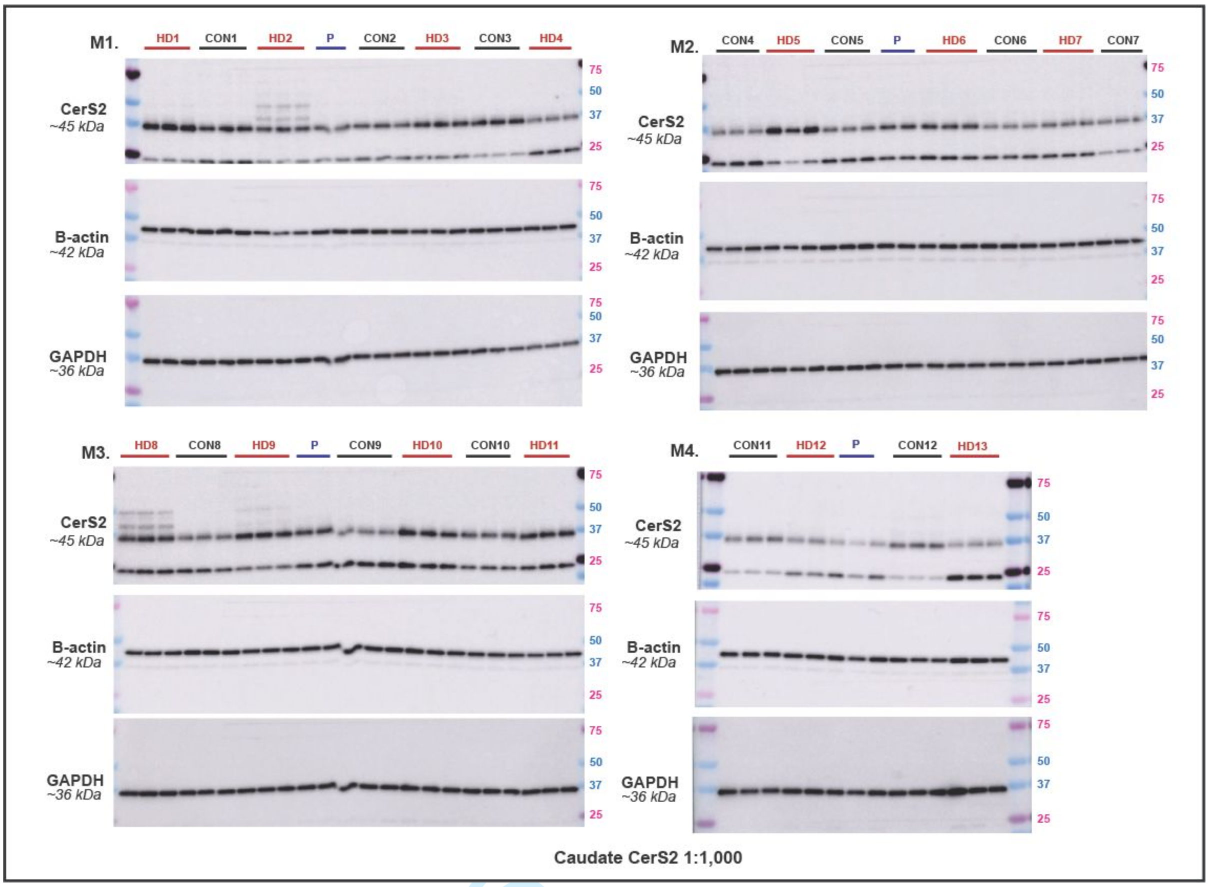

Supplementary Figure S8 Western Blot of CerS2 (1:1,000; ab176709, Abcam, Cambridge United Kingdom) in Control and HD Caudate. Membranes have been cut to show 25-75 kDa. Western blot of β-actin (1:100,000; MAB1501) and GAPDH (1:50,000; Rb659-060908-WS) have been included below their respective CerS1 membranes. Samples were loaded at 12.5 µg protein in triplicate. Values were averaged and adjusted for Pool samples and to housekeepers. **CerS2** Ceramide Synthase 2, **CON** Control, **GAPDH** Glyceraldehyde 3-phosphate dehydrogenase, **HD** Huntington's Disease, **P** Pool, **TBST** Tris-Buffered Saline with Tween 20

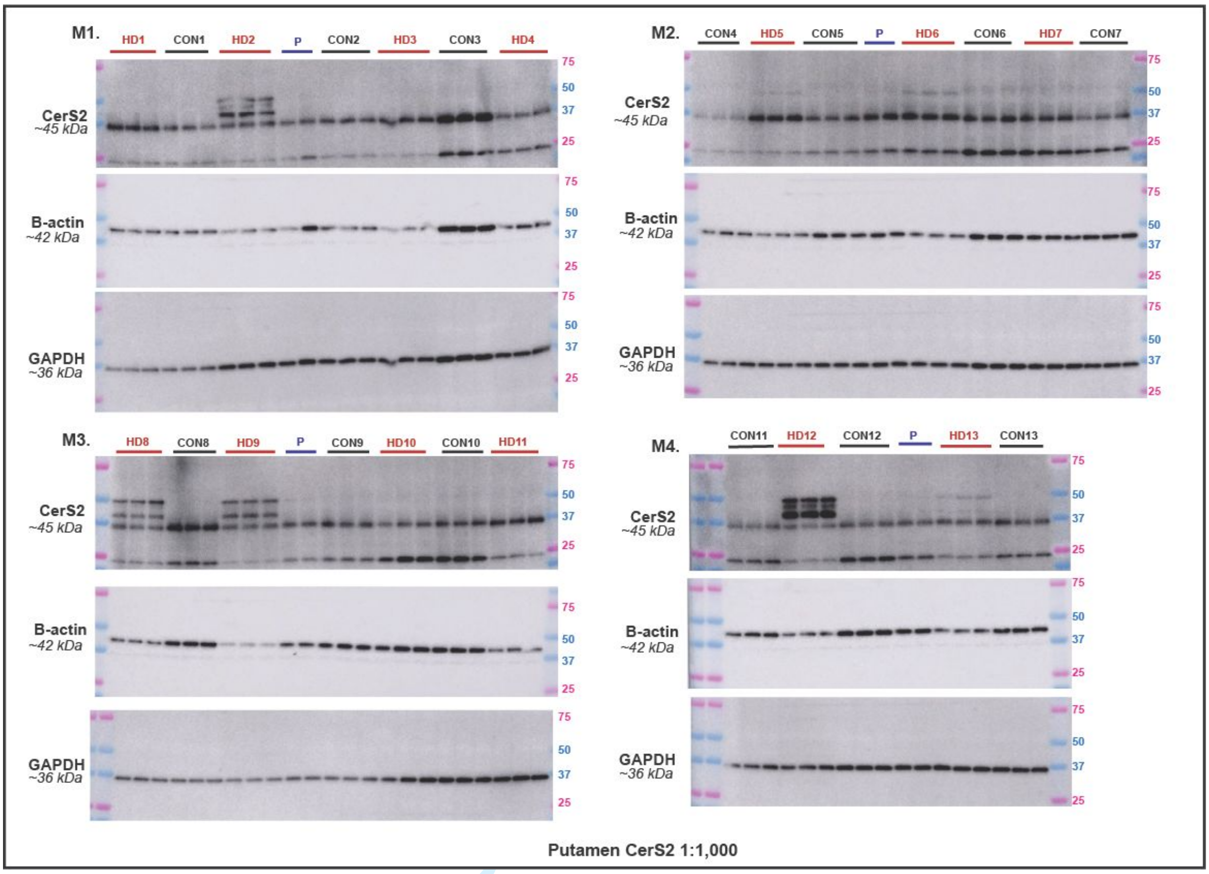

Supplementary Figure S9 Western Blot of CerS2 (1:1,000; ab176709, Abcam, Cambridge United Kingdom) in Control and HD Putamen. Membranes have been cut to show 25-75 kDa. Western blot of β-actin (1:100,000; MAB1501) and GAPDH (1:50,000; Rb659-060908-WS) have been included below their respective CerS1 membranes. Samples were loaded at 12.5 µg protein in triplicate. Values were averaged and adjusted for Pool samples and to housekeepers. **CerS2** Ceramide Synthase 2, **CON** Control, **GAPDH** Glyceraldehyde 3-phosphate dehydrogenase, **HD** Huntington's Disease, **P** Pool, **TBST** Tris-Buffered Saline with Tween 20

Review Only

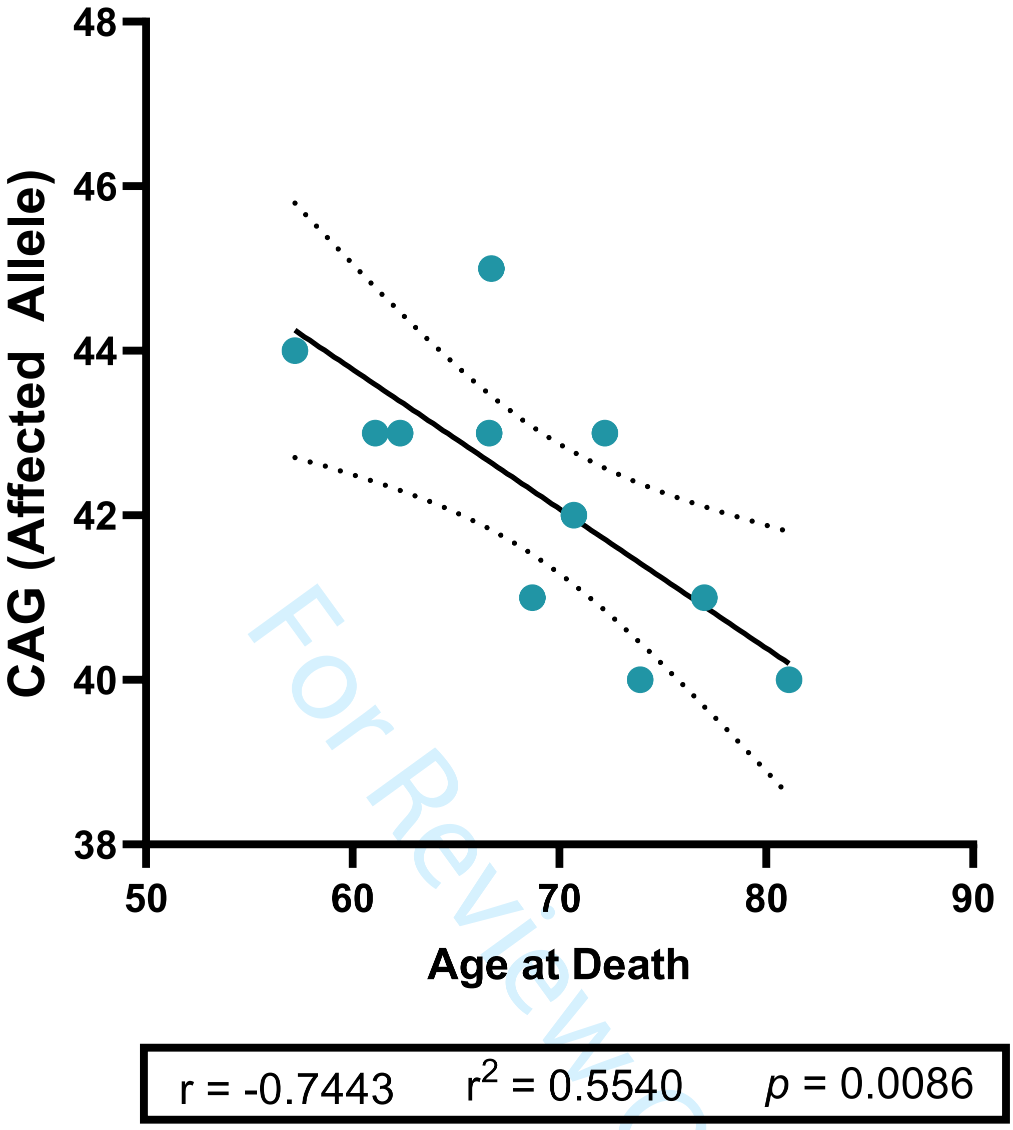

Figure S10 Pearson's correlation analysis of Age at Death *vs* CAG repeat length in HD subjects. CAG repeat length was found to have a strong negative correlation with Age of Death (r=-0.7443, p=0.0086). Dotted lines indicate 95% confidence intervals. Two subjects were removed from the analysis due to the cause of death. Only subjects with a clinical history of the cause of death as Huntington's disease were included. Correlations were completed using the disease-specific allele (>39 CAG).

For Review Only

Supplementary Material (Tables & Methods)

The Long and the Short of Huntington's Disease: How the Sphingolipid Profile is Shifted in the Caudate of Advanced Clinical Cases

Gabrielle R. Phillips[1,2,3], Jennifer T. Saville[4], Sarah E. Hancock[5], Simon HJ. Brown[3,6], Andrew M. Jenner[7], Catriona McLean[8], Maria Fuller[4,9], Kelly A. Newell[1,2,3], Todd Mitchell[1,2,3]*

RUNNING TITLE

Chain length alterations to sphingolipids in Huntington's

AFFLIATIONS

[1]Illawarra Health and Medical Research Institute, Wollongong, 2522, NSW, Australia

[2]School of Medicine, University of Wollongong, Wollongong, 2522, NSW, Australia

[3]Molecular Horizons, University of Wollongong, Wollongong, 2522, NSW, Australia

[4]Genetics and Molecular Pathology, SA Pathology at Women's and Children's Hospital, North Adelaide, 5006, SA, Australia

[5]School of Medical Sciences, University of New South Wales, Sydney, 2052, NSW, Australia

[6]School of Chemistry and Molecular Biosciences, University of Wollongong, Wollongong, 2522, NSW, Australia

[7]Bioanalytical Mass Spectrometry Facility, Mark Wainwright Analytical Centre, University of New South Wales, Sydney, 2052, NSW, Australia

[8]Department of Anatomical Pathology, Alfred Health and Florey Neuroscience, Parkville, 3052, VIC, Australia

[9]Adelaide Medical School, University of Adelaide, Adelaide, 5000, SA, Australia

For Review Only

1. Supplementary Methods

1.1 Sphingolipid Nomenclature

Table S1 Lipid abbreviations as recommended by Liebisch et al., 2020[45].

Lipid	Abbreviation	Example
Ceramide	Cer	Cer 18:1;O2/16:0
Sphingomyelin	SM	SM 18:1;O2/16:0
Hexosylceramides (glucosylceramides & galactosylceramides)	Hex-Cer	Hex-Cer 18:1;O2/16:0
Dihexosylceramides (Lactosylceramides)	Hex2Cer	Hex2Cer 18:1;O2/16:0
Sulfatides	SHexCer	SHexCer 18:1;O2/16:0
Hydroxylated Sulfatides	SHexCer (2OH)	SHexCer 18:1;O2/16:0(2OH)

For Review Only

1.2 Mass Spectrometry Scans

Table S2 Precursor Scans used for Lipid Identifications

Lipid Class	Scan	Collision Energy
Ceramides	+Prec. m/z 184.1	40
Sphingomyelins	+Prec. m/z 264.4	35

Abbreviations: Prec. Precursor

For Review Only

1.3 Western Blotting

Table S3 Antibody Information for Western Blot Experiments

Antibody	Function	Concentration	Supplier
Anti-Ceramide Synthase 1 (ab131169) [Recombinant] Rabbit Monoclonal	Synthesises Cer C18 species	1:5,000 (2.5% milk in TBST)	Abcam, Cambridge, United Kingdom
Anti-Ceramide Synthase 2 (ab176709) Rabbit Polyclonal	Synthesises Cer C22-C24 species	1:1,000 (2.5% milk in TBST)	Abcam, Cambridge, United Kingdom
GAPDH (Rb659-060908-WS) Rabbit	Housekeeper (Glycolysis)	1:50,000 (1% milk in TBST)	Osenses, Keswick, South Australia, Australia
β-actin (MAB1501) Mouse	Housekeeper (Cell Structure)	1:100,000 (1% milk in TBST)	Merck & Co. (Formally Merck Millipore), New Jersey, United States
Goat x Rabbit Secondary (AP307P)	Secondary Antibody	1:5,000 (2.5% milk in TBST)	Merck & Co. (Formally Merck Millipore), New Jersey, United States
Donkey x Mouse Secondary (AP308P)	Secondary Antibody	1:5,000 (1% milk in TBST)	Merck & Co. (Formally Merck Millipore), New Jersey, United States

Abbreviations: Cer Ceramide, GAPDH Glyceraldehyde 3-phosphate dehydrogenase

For Review Only

Lipid Concentrations and Statistics

2.1 Caudate

Table S4 Concentrations of ceramide species in the caudate. Data was assessed for normality using a D'Agostino Pearson Omnibus test. Data which was normally distributed was analysed using an unpaired t-test with Welch's correction, whilst data that did not fit normality assumptions was analysed using a Mann Whitney U test. P values are shown with corresponding significance (**p<0.01). Data is in pmol lipid per mg tissue.

	Control			HD				
Caudate	Mean	SEM	N	Mean	SEM	N	% DIFF	P
Cer 18:1;O2/16:0	6.16	0.33	11	10.27	1.27	11	66.59	0.0095**
Cer 18:1;O2/18:0	131.89	8.84	11	134.30	14.31	11	1.83	0.8879
Cer 18:1;O2/19:0	2.26	0.12	10	1.89	0.30	6	-16.42	0.0420
Cer 18:1;O2/20:0	11.96	0.72	11	11.85	1.10	11	-0.92	0.9343
Cer 18:1;O2/21:0	2.31	0.31	10	3.00	0.32	10	30.16	0.133
Cer 18:1;O2/22:0	5.20	0.33	11	5.37	0.66	11	3.31	0.6994
Cer 18:1;O2/22:1	3.24	0.20	11	2.28	0.19	9	-29.56	0.0026**
Cer 18:1;O2/22:2	2.28	0.32	9	1.52	0.13	6	-33.13	0.0256
Cer 18:1;O2/23:0	3.85	0.19	11	3.74	0.21	10	-2.96	0.6932
Cer 18:1;O2/24:0	3.54	0.25	11	3.53	0.29	11	-0.24	0.9828
Cer 18:1;O2/24:1	34.22	2.42	11	24.48	2.01	11	-28.48	0.0059**
Cer 18:1;O2/24:2	10.14	0.91	11	5.94	0.62	11	-41.42	0.0013**
Cer 18:1;O2/25:0	1.75	0.18	9	1.59	0.24	7	-9.13	0.3652

For Review Only

Total Ceramide	217.65	10.64	11	206.60	18.71	11	-5.08	0.6149

Abbreviations: Cer Ceramide, HD Huntington's Disease, SEM Standard Error of Mean, % DIFF Percentage difference of HD compared to controls.

Table S5 Concentrations of sphingomyelin species in the Putamen. Data was assessed for normality using a D'Agostino Pearson Omnibus test. Data which was normally distributed was analysed using an unpaired t-test with Welch's correction, whilst data that did not fit normality assumptions was analysed using a Mann Whitney U test. P values are shown with corresponding significance (**p<0.01, ***p<0.001). Data is in nmol lipid per mg tissue.

For Review Only

		Control			HD			
Caudate	Mean	SEM	N	Mean	SEM	N	% DIFF	P
SM 18:1;O2/14:0	8.49	0.59	11	10.74	1.25	11	26.49	0.1178
SM 18:1;O2/15:0	3.85	0.79	10	7.50	0.76	11	95.05	0.0011**
SM 18:1;O2/16:0	219.90	16.93	11	321.63	23.00	11	46.26	0.0010**
SM 18:1;O2/16:1	6.47	0.40	11	8.96	0.60	11	38.40	0.0026**
SM 18:1;O2/17:0	28.07	1.68	11	49.23	4.32	11	75.38	0.0002***
SM 18:1;O2/18:0	2074.25	100.61	11	2478.91	169.08	11	19.51	0.0530
SM 18:1;O2/18:1	194.40	11.94	11	264.34	17.90	11	35.98	0.0040**
SM 18:1;O2/19:0	29.57	1.38	11	34.00	2.66	11	14.99	0.1546
SM 18:1;O2/20:1	26.22	1.98	11	23.65	2.91	11	-9.81	0.4725
SM 18:1;O2/21:0	7.79	0.49	9	7.49	0.60	10	-3.83	0.7104
SM 18:1;O2/22:0	52.40	3.23	11	61.61	4.02	11	17.58	0.0892
SM 18:1;O2/22:1	19.05	1.94	11	21.39	2.26	11	12.26	0.4009
SM 18:1;O2/23:0	32.16	2.02	11	25.91	2.36	11	-19.42	0.0582
SM 18:1;O2/23:1	25.98	2.07	11	22.56	1.95	11	-13.15	0.2703
SM 18:1;O2/24:0	80.34	5.45	11	54.06	4.78	11	-32.72	0.0017**
SM 18:1;O2/24:1	536.40	45.49	11	351.00	30.87	11	-34.56	0.0030**

SM 18:1;O2/24:2	26.33	2.43	11	22.55	1.44	11	-14.36	0.1963
SM 18:1;O2/25:0	28.55	2.26	11	16.25	1.32	11	-43.08	0.0001***
SM 18:1;O2/25:1	139.09	13.38	11	94.36	12.53	11	-32.16	0.0241
SM 18:1;O2/26:0	7.81	0.61	11	5.57	1.27	9	-28.61	0.0310
SM 18:1;O2/26:1	88.81	7.29	11	54.79	5.96	11	-38.31	0.0017**
SM 18:1;O2/26:2	8.18	0.60	11	7.34	0.99	10	-10.29	0.4666
Total SM	3642.34	162.19	11	3941.48	257.29	11	8.21	0.3371

Abbreviations: HD Huntington's disease, SEM Standard Error of Mean, SM Sphingomyelin. % DIFF Percentage difference of HD compared to controls.

Table S6 Concentrations of HexCer species in caudate. Data was assessed for normality using a D'Agostino Pearson Omnibus test. Data which was normally distributed was analysed using an unpaired t-test with Welch's correction, whilst data that did not fit normality assumptions was analysed using a Mann Whitney U test. P values are shown with corresponding significance. Data is in pmol lipid per mg protein.

For Review Only

	CONTROL			HD				
Caudate	Mean	SEM	N	Mean	SEM	N	% DIF	*P*
Hex-Cer 18:1;O2/16:0	64.34	9.25	12	65.52	8.75	12	1.83	0.9999
Hex-Cer 18:1;O2/18:0	1609.68	275.83	12	962.82	146.32	12	-40.19	0.1005
Hex-Cer 18:1;O2/20:0	165.31	27.29	12	104.90	15.02	12	-36.55	0.0664
Hex-Cer 18:1;O2/22:0	278.12	47.45	12	163.78	26.49	12	-41.11	0.0471
Hex-Cer 18:1;O2/23:0	436.51	70.58	12	248.25	41.80	12	-43.13	0.0316
Hex-Cer 18:1;O2/24:0	990.11	161.44	12	549.23	100.18	12	-44.53	0.0597
Hex-Cer 18:1;O2/24:1	5127.63	1028.27	12	2682.25	402.95	12	-47.69	0.0375
Hex-Cer 18:1;O2/25:0	424.00	65.56	12	256.05	44.15	12	-39.61	0.0597
Hex-Cer 18:1;O2/26:0	101.36	15.03	12	66.59	11.04	12	-34.30	0.1100
Hex-Cer 18:1;O2/26:1	1026.13	163.48	12	700.32	101.35	12	-31.75	0.1041
Total HexCer	10223.18	1833.50	12	5353.58	929.11	13	-47.63	0.0453

Abbreviations: HexCer Monohexosylceramide, HD Huntington's Disease, SEM Standard Error of Mean, % DIFF Percentage difference of HD compared to controls.

Table S7 Concentrations of Hex2Cer species in the caudate. Data was assessed for normality using a D'Agostino Pearson Omnibus test. Data which was normally distributed was analysed using an unpaired t-test with Welch's correction, whilst data that did not fit normality assumptions was analysed using a Mann Whitney U test. P values are shown with corresponding significance (**p<0.01, ***p<0.001). Data is in pmol lipid per mg protein.

		CONTROL			HD			
Caudate	Mean	SEM	N	Mean	SEM	N	% DIF	*P*
Hex2Cer 18:1;O2/16:0	28.97	6.21	12	39.31	3.81	12	35.67	0.0058**
Hex2Cer 18:1;O2/18:0	143.25	13.66	12	206.37	22.31	12	44.07	0.0246
Hex2Cer 18:1;O2/22:0	21.15	2.59	12	24.02	2.76	12	13.56	0.4767
Hex2Cer 18:1;O2/24:0	56.21	7.16	12	42.78	5.94	12	-23.90	0.1926
Hex2Cer 18:1;O2/24:1	266.87	39.10	12	211.11	35.91	12	-20.89	0.0001***
Hex2Cer 18:1;O2/25:0	26.64	3.49	12	19.37	3.16	12	-27.28	0.0656
Hex2Cer 18:1;O2/26:0	8.05	0.92	12	6.41	0.89	12	-20.38	0.2149
Hex2Cer 18:1;O2/26:1	77.75	11.24	12	63.97	10.44	12	-17.73	0.3777
Total Hex2Cer	628.89	75.14	12	566.15	71.76	13	-9.9754	0.8711

Abbreviations: Hex2Cer Dihexosylceramide, HD Huntington's Disease, SEM Standard Error of Mean, % DIFF Percentage difference of HD compared to controls.

For Review Only

Table S8 Concentrations of sulfatide species in the caudate. Data was assessed for normality using a D'Agostino Pearson Omnibus test. Data which was normally distributed was analysed using an unpaired t-test with Welch's correction, whilst data that did not fit normality assumptions was analysed using a Mann Whitney U test. P values are shown with corresponding significance (**p<0.01). Data is in pmol lipid per mg protein.

	CONTROL			HD				
	Mean	SEM	N	Mean	SEM	N	% DIF	*P*
SHexCer 18:1;O2/16:0	6.55	0.55	12	8.31	1.14	12	26.82	0.2828
SHexCer 18:1;O2/18:0	135.89	20.33	12	110.57	20.52	12	-18.63	0.2718
SHexCer 18:1;O2/20:0	28.33	4.52	12	21.02	3.69	12	-25.81	0.1554
SHexCer 18:1;O2/22:0	124.29	17.85	12	66.88	12.71	12	-46.19	0.0095**
SHexCer 18:1;O2/23:0	169.53	25.72	12	96.74	17.28	12	-42.94	0.0196
SHexCer 18:1;O2/24:0	586.80	85.52	12	308.23	66.54	12	-47.47	0.0068**
SHexCer 18:1;O2/24:1	622.30	105.42	12	292.04	51.02	12	-53.07	0.0057**
SHexCer 18:1;O2/18:0(2OH)	17.45	2.50	12	17.28	1.56	12	-1.00	0.5587
SHexCer 18:1;O2/20:0(2OH)	55.96	6.90	12	53.67	5.58	12	-4.10	0.8029
SHexCer 18:1;O2/22:0(2OH)	245.06	37.82	12	194.17	27.88	12	-20.77	0.3259
SHexCer 18:1;O2/23:0(2OH)	498.59	84.96	12	338.28	48.74	12	-32.15	0.1240
SHexCer 18:1;O2/24:0(2OH)	1158.54	171.19	12	759.43	109.73	12	-34.45	0.0387
SHexCer 18:1;O2/24:1(2OH)	1131.80	204.93	12	891.60	115.62	12	-21.22	0.5137
Total Sulfatide	1673.69	256.19	12	834.26	169.26	12	-50.15	0.0121
Total *OH*-Sulfatide	3107.41	504.90	12	2081.01	328.85	12	-33.03	0.1649
Total Combined Sulfatide	4781.10	728.80	12	2915.27	468.93	12	-39.03	0.0721

Abbreviations: HD Huntington's Disease, SEM Standard Error of Mean, % DIFF Percentage difference of HD compared to controls.

For Review Only

2.2 Putamen

Table S9 Concentrations of ceramide species in the putamen. Data was assessed for normality using a D'Agostino Pearson Omnibus test. Data which was normally distributed was analysed using an unpaired t-test with Welch's correction, whilst data that did not fit normality assumptions was analysed using a Mann Whitney U test. P values are shown with corresponding significance (***p<0.001). Data is in pmol lipid per mg tissue.

	Control			HD				
Putamen	Mean	SEM	N	Mean	SEM	N	% DIFF	P
Cer 18:1;O2/16:0	8.16	0.37	11	10.46	0.99	13	28.14	0.0006***
Cer 18:1;O2/18:0	149.49	6.75	11	192.53	21.62	13	28.79	0.1584
Cer 18:1;O2/19:0	2.82	0.20	11	3.22	0.28	11	14.23	0.1164
Cer 18:1;O2/20:0	61.66	4.33	11	14.75	1.00	13	-76.08	0.9547
Cer 18:1;O2/21:0	2.93	0.38	9	3.56	0.41	11	21.27	0.6713
Cer 18:1;O2/22:0	7.24	0.57	11	7.53	0.86	13	3.96	0.4940
Cer 18:1;O2/22:1	4.30	1.19	11	8.31	1.26	13	93.38	0.1674
Cer 18:1;O2/22:2	5.49	0.51	11	5.24	1.27	12	-4.48	0.1693
Cer 18:1;O2/23:0	4.98	0.66	10	7.22	0.86	13	45.05	0.0303
Cer 18:1;O2/24:0	10.44	0.85	11	6.82	0.87	13	-34.63	0.2871
Cer 18:1;O2/24:1	50.08	12.22	11	94.83	19.54	13	89.37	0.2284
Cer 18:1;O2/24:2	14.82	4.13	11	33.08	5.48	13	123.20	0.0933
Cer 18:1;O2/25:0	4.16	0.46	10	5.28	0.88	13	27.08	0.2080
Total Ceramide	321.59	23.25	11	391.39	45.26	13	21.70	0.0864

Abbreviations: Cer Ceramide, HD Huntington's Disease, SEM Standard Error of Mean, % DIFF Percentage difference of HD compared to controls.

For Review Only

Table S10 Concentrations of sphingomyelin species in the putamen. Data was assessed for normality using a D'Agostino Pearson Omnibus test. Data which was normally distributed was analysed using an unpaired t-test with Welch's correction, whilst data that did not fit normality assumptions was analysed using a Mann Whitney U test. P values are shown with corresponding significance (**p<0.01, ***p<0.001). Data is in pmol lipid per mg tissue.

	Control			HD				
Putamen	Mean	SEM	N	Mean	SEM	N	% DIFF	P
SM 18:1;O2/14:0	13.93	0.71	11	22.55	2.80	13	61.92	0.0008***
SM 18:1;O2/15:0	3.77	0.41	10	8.87	1.05	13	135.24	0.0005***
SM 18:1;O2/16:0	263.37	10.76	11	398.71	38.84	13	51.39	0.0019**
SM 18:1;O2/16:1	6.82	0.32	10	15.18	1.37	13	122.49	0.0000***
SM 18:1;O2/17:0	33.87	2.18	11	64.69	6.41	13	91.02	0.0002***
SM 18:1;O2/18:0	2329.13	90.53	11	2886.31	330.35	13	23.92	0.3445
SM 18:1;O2/18:1	176.51	11.45	11	403.03	28.25	13	128.34	0.0000***
SM 18:1;O2/19:0	37.77	2.09	11	44.48	3.03	13	17.78	0.0632
SM 18:1;O2/20:1	23.79	1.55	11	28.24	1.80	13	18.70	0.0743
SM 18:1;O2/21:0	12.09	0.86	10	12.51	0.83	13	3.49	0.7308
SM 18:1;O2/22:0	54.79	7.03	11	81.68	6.11	13	49.08	0.1674
SM 18:1;O2/22:1	26.69	3.15	11	58.52	8.19	13	119.24	0.0044**
SM 18:1;O2/23:0	52.81	7.74	11	70.08	11.23	13	32.70	0.3311
SM 18:1;O2/23:1	40.82	5.51	11	91.89	14.25	13	125.10	0.0115
SM 18:1;O2/24:0	100.75	21.45	11	134.47	23.40	13	33.48	0.9095
SM 18:1;O2/24:1	952.04	133.75	11	1455.92	274.56	13	52.93	0.3031
SM 18:1;O2/24:2	37.28	5.34	11	68.00	8.65	13	82.38	0.0432
SM 18:1;O2/25:0	30.87	6.56	11	58.45	9.55	13	89.32	0.2505
SM 18:1;O2/25:1	238.22	33.52	11	438.97	68.42	13	84.27	0.0393
SM 18:1;O2/26:0	12.25	2.15	9	14.08	2.12	13	15.00	0.5626
SM 18:1;O2/26:1	145.90	17.65	10	212.57	32.62	13	45.70	0.0672
SM 18:1;O2/26:2	13.75	1.90	10	24.57	2.85	13	78.61	0.0077**
Total Sphingomyelin	4558.54	296.17	11	6593.78	753.73	13	44.65	0.0641

Abbreviations: HD Huntington's Disease, SEM Standard Error of Mean, SM Sphingomyelin. % DIFF Percentage difference of HD compared to controls.

For Review Only

Table S11 Concentrations of HexCer species in the putamen. Data was assessed for normality using a D'Agostino Pearson Omnibus test. Data which was normally distributed was analysed using an unpaired t-test with Welch's correction, whilst data that did not fit normality assumptions was analysed using a Mann Whitney U test. Data is in pmol lipid per mg protein.

	CON			HD				
Putamen	Mean	SEM	N	Mean	SEM	N	% DIFF	P
Hex-Cer 18:1;O2/16:0	156.60	36.40	12	179.63	16.98	13	14.71	0.2305
Hex-Cer 18:1;O2/18:0	4715.43	1289.18	12	3864.50	449.48	13	-18.05	0.6885
Hex-Cer 18:1;O2/20:0	381.75	92.42	12	324.08	33.75	13	-15.11	0.6885
Hex-Cer 18:1;O2/22:0	833.82	258.90	12	620.66	89.50	13	-25.56	0.7283
Hex-Cer 18:1;O2/23:0	1239.92	363.71	12	1057.70	148.82	13	-14.70	0.6885
Hex-Cer 18:1;O2/24:0	2773.73	924.73	12	1983.09	273.48	13	-28.50	0.7283
Hex-Cer 18:1;O2/24:1	15392.13	5099.78	12	11049.42	1525.19	13	-28.21	0.8100
Hex-Cer 18:1;O2/25:0	863.37	216.78	12	806.53	102.00	13	-6.58	0.4783
Hex-Cer 18:1;O2/26:0	178.16	40.98	12	178.07	21.72	13	-0.05	0.3544
Hex-Cer 18:1;O2/26:1	2440.47	666.04	12	2359.52	321.57	13	-3.32	0.4059
Total HexCer	28975.36	8894.69	12	22423.22	2904.70	13	-22.61	0.7689

Abbreviations: HexCer Monohexosylceramide, HD Huntington's Disease, SEM Standard Error of Mean, % DIFF Percentage difference of HD compared to controls.

For Review Only

Table S12 Concentrations of Hex2Cer species in the putamen. Data was assessed for normality using a D'Agostino Pearson Omnibus test. Data which was normally distributed was analysed using an unpaired t-test with Welch's correction, whilst data that did not fit normality assumptions was analysed using a Mann Whitney U test. P values are shown with corresponding significance. Data is in pmol lipid per mg protein.

	CON			HD				
Putamen	Mean	SEM	N	Mean	SEM	N	% DIFF	P
Hex2Cer 18:1;O2/16:0	32.33	4.08	12	49.70	4.99	13	53.72	0.0128
Hex2Cer 18:1;O2/18:0	274.45	47.15	12	359.80	36.18	13	31.10	0.3659
Hex2Cer 18:1;O2/22:0	40.99	6.52	12	50.95	4.69	13	24.31	0.8519
Hex2Cer 18:1;O2/24:0	114.16	21.99	12	125.57	13.83	13	10.00	0.6051
Hex2Cer 18:1;O2/24:1	574.74	112.21	12	623.17	55.12	13	8.43	0.2051
Hex2Cer 18:1;O2/25:0	57.06	10.68	12	65.29	7.34	13	14.43	0.7797
Hex2Cer 18:1;O2/26:0	12.87	1.99	12	15.12	1.46	13	17.52	0.8680
Hex2Cer 18:1;O2/26:1	161.81	29.22	12	213.95	23.25	13	32.23	0.6117
Total Hex2Cer	1268.40	228.59	12	1503.56	131.56	13	18.54	0.3848

Abbreviations: Hex2Cer Dihexosylceramide, HD Huntington's Disease, SEM Standard Error of Mean, % DIFF Percentage difference of HD compared to controls.

For Review Only

Table S13 Concentrations of sulfatide species in the putamen. Data was assessed for normality using a D'Agostino Pearson Omnibus test. Data which was normally distributed was analysed using an unpaired t-test with Welch's correction, whilst data that did not fit normality assumptions was analysed using a Mann Whitney U test. P values are shown with corresponding significance. Data is in pmol lipid per mg protein.

	CON			HD				
Putamen	Mean	SEM	N	Mean	SEM	N	% DIFF	P
SHexCer 18:1;O2/16:0	10.44	1.49	12	10.91	0.78	13	4.52	0.8354
SHexCer 18:1;O2/18:0	370.18	95.10	12	324.71	43.09	13	-12.28	0.2976
SHexCer 18:1;O2/20:0	91.41	26.12	12	64.98	8.47	13	-28.92	0.1749
SHexCer 18:1;O2/22:0	291.72	68.85	12	222.13	27.85	13	-23.85	0.1724
SHexCer 18:1;O2/23:0	465.13	127.91	12	340.53	48.40	13	-26.79	0.1796
SHexCer 18:1;O2/24:0	1440.74	396.38	12	1066.60	151.24	13	-25.97	0.9787
SHexCer 18:1;O2/24:1	1651.78	413.80	12	1199.34	150.02	13	-27.39	0.1532
SHexCer 18:1;O2/18:0(2OH)	42.47	10.82	12	52.05	5.64	13	22.58	0.0948
SHexCer 18:1;O2/20:0(2OH)	93.52	10.64	12	119.12	7.15	13	27.37	0.0332
SHexCer 18:1;O2/22:0(2OH)	577.87	116.38	12	687.27	59.54	13	18.93	0.1519
SHexCer 18:1;O2/23:0(2OH)	1440.13	371.85	12	1435.94	170.06	13	-0.29	0.3760
SHexCer 18:1;O2/24:0(2OH)	2548.04	441.65	12	3065.92	278.29	13	20.32	0.6684
SHexCer 18:1;O2/24:1(2OH)	3101.88	766.63	12	3653.81	317.16	13	17.79	0.0768
Total Sulfatide	4321.39	1113.10	12	3229.20	416.85	13	-25.27	0.3737
Total OH-Sulfatide	7803.91	1698.79	12	9014.11	812.69	13	15.51	0.1683
Total Combined Sulfatide	12125.29	2600.98	12	12243.31	1181.16	13	0.97	0.9676

Abbreviations: HD Huntington's Disease, SEM Standard Error of Mean, % DIFF Percentage difference of HD compared to controls.

For Review Only

2.3 Cerebellum

Table S14 Concentrations of ceramide species in the cerebellum. Data was assessed for normality using a D'Agostino Pearson Omnibus test. Data which was normally distributed was analysed using an unpaired t-test with Welch's correction, whilst data that did not fit normality assumptions was analysed using a Mann Whitney U test. P values are shown with corresponding significance (***p<0.001). Data is in pmol lipid per mg protein.

	Control			HD				
Cerebellum	Mean	SEM	N	Mean	SEM	N	% DIFF	P
Cer 18:1;O2/16:0	5.81	0.73	13	5.23	0.49	9	-9.86	0.8446
Cer 18:1;O2/18:0	80.41	6.56	13	97.20	5.07	9	20.88	0.0563
Cer 18:1;O2/19:0	1.69	0.18	7	1.75	0.19	6	3.96	0.6282
Cer 18:1;O2/20:0	9.50	0.70	13	10.94	0.65	9	15.19	0.1452
Cer 18:1;O2/21:0	2.78	0.21	13	2.22	0.32	9	-20.10	0.1645
Cer 18:1;O2/22:0	3.81	0.56	13	3.73	0.53	9	-1.97	0.9236
Cer 18:1;O2/22:1	2.15	0.28	11	2.39	0.22	9	11.15	0.5105
Cer 18:1;O2/22:2	2.34	0.45	8	1.66	0.16	7	-29.16	0.2810
Cer 18:1;O2/23:0	2.22	0.74	5	1.37	0.19	5	-38.29	0.4206
Cer 18:1;O2/24:0	4.70	1.11	13	2.50	0.25	9	-46.65	0.0708
Cer 18:1;O2/24:1	18.79	2.74	13	21.60	3.08	9	14.94	0.2624
Cer 18:1;O2/24:2	6.12	0.96	13	7.28	0.97	9	18.83	0.4085
Cer 18:1;O2/25:0	2.35	0.54	7	1.39	0.11	8	-40.99	0.0002***
Total Ceramide	138.20	11.05	13	157.56	8.40	9	14.00	0.1785

Abbreviations: Cer Ceramide, HD Huntington's Disease, SEM Standard Error of Mean, % DIFF Percentage difference of HD compared to controls.

For Review Only

Table S15 Concentrations of sphingomyelin species in the cerebellum. Data was assessed for normality using a D'Agostino Pearson Omnibus test. Data which was normally distributed was analysed using an unpaired t-test with Welch's correction, whilst data that did not fit normality assumptions was analysed using a Mann Whitney U test. P values are shown with corresponding significance (*p<0.05). Data is in pmol lipid per mg tissue.

	Control			HD				
Cerebellum	Mean	SEM	N	Mean	SEM	N	% DIFF	P
SM 18:1;O2/14:0	15.32	1.29	13	12.63	0.88	9	-17.59	0.1334
SM 18:1;O2/15:0	3.78	0.55	10	3.55	0.47	8	-6.34	0.7527
SM 18:1;O2/16:0	214.26	19.06	13	199.02	17.01	9	-7.11	0.7438
SM 18:1;O2/16:1	7.25	1.81	13	5.27	0.51	9	-27.29	0.6948
SM 18:1;O2/17:0	17.97	1.45	13	21.90	1.92	9	21.86	0.1120
SM 18:1;O2/18:0	2030.61	184.45	13	2249.65	172.79	9	10.79	0.4179
SM 18:1;O2/18:1	114.92	13.24	13	142.82	12.86	9	24.28	0.1619
SM 18:1;O2/19:0	16.36	1.40	13	17.65	1.33	9	7.90	0.5293
SM 18:1;O2/20:1	10.54	0.91	13	12.58	0.74	9	19.33	0.3632
SM 18:1;O2/21:0	3.72	0.41	10	3.30	0.29	7	-11.30	0.5837
SM 18:1;O2/22:0	40.75	4.79	13	38.01	3.26	9	-6.71	0.8446
SM 18:1;O2/22:1	14.37	1.73	13	17.77	1.60	9	23.68	0.6731
SM 18:1;O2/23:0	19.47	2.22	13	24.26	3.70	9	24.59	0.1848
SM 18:1;O2/23:1	13.41	1.46	12	19.83	2.37	9	47.83	0.2517
SM 18:1;O2/24:0	60.82	7.81	13	62.44	8.07	9	2.67	0.0257
SM 18:1;O2/24:1	331.26	47.18	13	425.01	58.37	9	28.30	0.8896
SM 18:1;O2/24:2	18.71	2.46	13	22.87	3.07	9	22.23	0.3237
SM 18:1;O2/25:0	18.17	2.84	12	21.66	3.50	9	19.17	0.2996
SM 18:1;O2/25:1	67.96	11.26	13	103.06	13.95	9	51.65	0.4449
SM 18:1;O2/26:0	5.45	0.85	9	6.68	1.19	8	22.51	0.0630
SM 18:1;O2/26:1	46.73	7.87	13	64.14	9.46	9	37.27	0.4075
SM 18:1;O2/26:2	5.47	0.56	8	5.95	0.96	7	8.83	0.9999
Total SM	3069.35	265.29	13	3476.85	285.11	9	13.28	0.7481

Abbreviations: HD Huntington's Disease, SEM Standard Error of Mean, SM Sphingomyelin. % DIFF Percentage difference of HD compared to controls.

For Review Only

Table S16 Concentrations of HexCer species in the cerebellum. Data was assessed for normality using a D'Agostino Pearson Omnibus test. Data which was normally distributed was analysed using an unpaired t-test with Welch's correction, whilst data that did not fit normality assumptions was analysed using a Mann Whitney U test. Data is in pmol lipid per mg protein.

	CONTROL			HD				
Cerebellum	Mean	SEM	N	Mean	SEM	N	% DIFF	P
Hex-Cer 18:1;O2/16:0	82.45	11.83	13	125.11	19.74	13	51.74	0.0761
Hex-Cer 18:1;O2/18:0	2302.47	374.13	13	2748.51	486.05	13	19.37	0.4743
Hex-Cer 18:1;O2/20:0	180.85	22.13	13	230.10	36.79	13	27.24	0.2628
Hex-Cer 18:1;O2/22:0	315.40	46.63	13	416.17	72.23	13	31.95	0.2533
Hex-Cer 18:1;O2/23:0	501.47	76.96	13	674.88	115.50	13	34.58	0.3551
Hex-Cer 18:1;O2/24:0	1295.41	189.44	13	1582.66	271.92	13	22.17	0.3947
Hex-Cer 18:1;O2/24:1	6313.88	1131.22	13	8150.00	1540.56	13	29.08	0.5114
Hex-Cer 18:1;O2/25:0	534.27	78.17	13	631.93	98.17	13	18.28	0.5200
Hex-Cer 18:1;O2/26:0	129.02	19.35	13	144.79	21.14	13	12.22	0.6139
Hex-Cer 18:1;O2/26:1	1229.97	218.75	13	1539.47	235.16	13	25.16	0.2428
Total HexCer	12885.20	2144.62	13	16243.63	2863.08	13	26.06	0.4793

Abbreviations: HexCer Monohexosylceramide, HD Huntington's Disease, SEM Standard Error of Mean, % DIFF Percentage difference of HD compared to controls.

For Review Only

Table S17 Concentrations of Hex2Cer species in the cerebellum. Data was assessed for normality using a D'Agostino Pearson Omnibus test. Data which was normally distributed was analysed using an unpaired t-test with Welch's correction, whilst data that did not fit normality assumptions was analysed using a Mann Whitney U test. P values are shown with corresponding significance (**p<0.01). Data is in pmol lipid per mg protein.

	CONTROL			HD				
Cerebellum	Mean	SEM	N	Mean	SEM	N	% DIFF	P
Hex2Cer 18:1;O2/16:0	36.62	3.26	13	89.65	34.95	13	144.85	0.0020**
Hex2Cer 18:1;O2/18:0	242.77	20.19	13	330.94	33.71	13	36.32	0.0343
Hex2Cer 18:1;O2/22:0	15.26	1.28	13	27.13	4.24	13	77.75	0.0057**
Hex2Cer 18:1;O2/24:0	33.19	3.19	13	58.65	9.21	13	76.74	0.0155
Hex2Cer 18:1;O2/24:1	136.95	15.17	13	253.11	35.24	13	84.82	0.0058**
Hex2Cer 18:1;O2/25:0	14.93	1.61	13	25.33	3.92	13	69.69	0.0655
Hex2Cer 18:1;O2/26:0	4.42	0.49	13	7.39	0.90	13	67.36	0.0169
Hex2Cer 18:1;O2/26:1	37.97	4.92	13	68.97	9.85	13	81.62	0.0212
Total Hex2Cer	522.10	45.32	13	861.18	94.15	13	64.94	0.0090**

Abbreviations: Hex2Cer Dihexosylceramide, HD Huntington's Disease, SEM Standard Error of Mean, % DIFF Percentage difference of HD compared to controls.

For Review Only

Table S18 Concentrations of sulfatide species in the cerebellum. Data was assessed for normality using a D'Agostino Pearson Omnibus test. Data which was normally distributed was analysed using an unpaired t-test with Welch's correction, whilst data that did not fit normality assumptions was analysed using a Mann Whitney U test. P values are shown with corresponding significance. Data is in pmol lipid per mg protein.

	CONTROL			HD				
Cerebellum	Mean	SEM	N	Mean	SEM	N	% DIFF	P
SHexCer 18:1;O2/16:0	7.68	0.70	13	9.98	1.02	13	30.03	0.0930
SHexCer 18:1;O2/18:0	192.34	30.07	13	266.12	51.46	13	38.36	0.2276
SHexCer 18:1;O2/20:0	30.85	4.06	13	51.42	10.87	13	66.70	0.0906
SHexCer 18:1;O2/22:0	125.53	19.43	13	171.71	31.96	13	36.79	0.2301
SHexCer 18:1;O2/23:0	177.62	30.07	13	252.18	47.02	13	41.97	0.1936
SHexCer 18:1;O2/24:0	598.93	94.23	13	863.47	172.87	13	44.17	0.3622
SHexCer 18:1;O2/24:1	637.28	113.10	13	944.93	182.49	13	48.27	0.2642
SHexCer 18:1;O2/18:0(2OH)	21.46	2.36	13	28.78	4.17	13	34.08	0.2808
SHexCer 18:1;O2/20:0(2OH)	63.42	5.92	13	76.02	9.93	13	19.86	0.3043
SHexCer 18:1;O2/22:0(2OH)	216.90	25.30	13	351.74	55.02	13	62.17	0.0354
SHexCer 18:1;O2/23:0(2OH)	384.48	47.44	13	653.92	111.07	13	70.08	0.0353
SHexCer 18:1;O2/24:0(2OH)	1034.28	126.84	13	1660.61	284.25	13	60.56	0.0555
SHexCer 18:1;O2/24:1(2OH)	1013.50	147.87	13	1640.25	272.94	13	61.84	0.0548
Total Sulfatide	1770.22	289.79	13	2559.81	493.56	13	44.60	0.3107
Total OH-Sulfatide	2734.04	352.19	13	4411.31	733.84	13	61.35	0.0548
Total Combined Sulfatide	4504.26	623.66	13	6971.13	1213.74	13	54.77	0.0874

Abbreviations: HD Huntington's Disease, SEM Standard Error of Mean, % DIFF Percentage difference of HD compared to controls.

For Review Only

For Review Only

2.4 White Cortex

Table S19 Concentrations of ceramide species in the white matter of the dorsomedial prefrontal cortex. Data was assessed for normality using a D'Agostino Pearson Omnibus test. Data which was normally distributed was analysed using an unpaired t-test with Welch's correction, whilst data that did not fit normality assumptions was analysed using a Mann Whitney U test. P values are shown with corresponding significance. Data is in pmol lipid per mg tissue.

	CONTROL			HD				
White Cortex	Mean	SEM	N	Mean	SEM	N	% DIFF	P
Cer 18:1;O2/18:0	199.24	11.99	13	155.97	16.13	13	-21.71	0.0416
Cer 18:1;O2/22:0	22.38	1.99	10	18.18	2.08	13	-18.79	0.1685
Cer 18:1;O2/22:1	16.35	1.23	8	13.78	1.93	9	-15.72	0.2921
Cer 18:1;O2/23:0	21.19	1.59	13	19.74	2.46	13	-6.84	0.6245
Cer 18:1;O2/24:0	24.26	2.12	13	24.38	3.34	13	0.51	0.9756
Cer 18:1;O2/24:1	446.58	33.00	13	324.10	46.74	13	-27.43	0.0427
Cer 18:1;O2/24:2	48.67	2.40	13	40.77	4.47	13	-16.23	0.1327
Cer 18:1;O2/25:1	51.52	4.08	13	42.04	5.45	13	-18.40	0.1765
Cer 18:1;O2/26:1	19.05	1.38	10	15.17	1.84	13	-20.34	0.1249
Total Ceramide	833.38	56.60	13	649.90	81.35	13	-22.02	0.0765

Abbreviations: Cer Ceramide, HD Huntington's Disease, SEM Standard Error of Mean, % DIFF Percentage difference of HD compared to controls.

For Review Only

Table S20 Concentrations of sphingomyelin species in the white matter of the dorsomedial prefrontal cortex. Data was assessed for normality using a D'Agostino Pearson Omnibus test. Data which was normally distributed was analysed using an unpaired t-test with Welch's correction, whilst data that did not fit normality assumptions was analysed using a Mann Whitney U test. P values are shown with corresponding significance. Data is in pmol lipid per mg tissue.

	CONTROL			HD				
White Cortex	Mean	SEM	N	Mean	SEM	N	% DIFF	P
SM 18:1;O2/16:0	479.31	30.22	13	505.05	27.65	13	5.37	0.5356
SM 18:1;O2/18:0	3613.76	205.41	13	3759.29	227.14	13	4.03	0.6390
SM 18:1;O2/18:1	417.19	26.90	13	422.98	29.22	13	1.39	0.8853
SM 18:1;O2/20:0	355.65	20.97	13	428.14	50.93	13	20.38	0.3897
SM 18:1;O2/22:0	292.49	18.04	13	317.78	20.79	13	8.65	0.3673
SM 18:1;O2/22:1	228.44	10.92	13	213.43	19.05	13	-6.57	0.8403
SM 18:1;O2/23:0	363.99	20.98	13	373.76	26.75	13	2.69	0.7762
SM 18:1;O2/23:1	361.11	17.50	13	332.53	29.16	13	-7.91	0.7241
SM 18:1;O2/24:0	1124.59	77.23	13	1082.90	95.76	13	-3.71	0.7376
SM 18:1;O2/24:1	9556.72	484.82	13	7607.06	725.95	13	-20.40	0.0351
SM 18:1;O2/24:2	273.31	15.53	13	249.28	23.99	13	-8.79	0.4087
SM 18:1;O2/25:0	342.28	25.41	13	327.20	23.67	13	-4.41	0.6680
SM 18:1;O2/25:1	1592.11	67.87	13	1355.02	105.42	13	-14.89	0.0708
SM 18:1;O2/26:0	85.78	5.00	8	77.02	7.49	10	-10.20	0.3717
SM 18:1;O2/26:1	1006.68	46.59	13	910.94	63.92	13	-9.51	0.2379
Total Sphingomyelin	20060.39	920.83	13	17944.59	1280.17	13	-10.55	0.1950

Abbreviations: HD Huntington's Disease, SEM Standard Error of Mean, SM Sphingomyelin. % DIFF Percentage difference of HD compared to controls.

For Review Only

Table S21 Concentrations of HexCer species in the white matter of the dorsomedial prefrontal cortex. Data was assessed for normality using a D'Agostino Pearson Omnibus test. Data which was normally distributed was analysed using an unpaired t-test with Welch's correction, whilst data that did not fit normality assumptions was analysed using a Mann Whitney U test. P values are shown with corresponding significance. Data is in pmol lipid per mg protein.

	CONTROL			HD				
White Cortex	Mean	SEM	N	Mean	SEM	N	% DIFF	P
Hex-Cer 18:1;O2/16:0	891.50	73.05	13	789.82	73.24	13	-11.41	0.3360
Hex-Cer 18:1;O2/18:0	28981.23	2563.46	13	22738.77	2502.73	13	-21.54	0.0942
Hex-Cer 18:1;O2/20:0	2367.57	209.58	13	2073.46	183.28	13	-12.42	0.3017
Hex-Cer 18:1;O2/22:0	6779.04	517.58	13	6163.65	431.57	13	-9.08	0.3703
Hex-Cer 18:1;O2/23:0	11553.46	956.64	13	10136.08	749.66	13	-12.27	0.2550
Hex-Cer 18:1;O2/24:0	30910.85	2943.13	13	27283.08	2492.27	13	-11.74	0.3563
Hex-Cer 18:1;O2/24:1	108904.62	7199.75	13	84561.54	6559.08	13	-22.35	0.0197
Hex-Cer 18:1;O2/25:0	9178.58	1020.22	13	8221.58	824.49	13	-10.43	0.4728
Hex-Cer 18:1;O2/26:0	1541.46	186.57	13	1516.10	145.27	13	-1.65	0.9154
Hex-Cer 18:1;O2/26:1	22359.38	1866.69	13	19965.00	1595.59	13	-10.71	0.2226
Total HexCer	223467.68	16074.57	13	183449.07	14524.15	13	-17.91	0.0568

Abbreviations: HexCer Monohexosylceramide, HD Huntington's Disease, SEM Standard Error of Mean, % DIFF Percentage difference of HD compared to controls.

For Review Only

Table S22 Concentrations of Hex2Cer species in the white matter of the dorsomedial prefrontal cortex. Data was assessed for normality using a D'Agostino Pearson Omnibus test. Data which was normally distributed was analysed using an unpaired t-test with Welch's correction, whilst data that did not fit normality assumptions was analysed using a Mann Whitney U test. P values are shown with corresponding significance. Data is in pmol lipid per mg protein.

	CONTROL			HD				
White Cortex	Mean	SEM	N	Mean	SEM	N	% DIFF	P
Hex2Cer 18:1;O2/16:0	84.12	5.98	13	87.68	4.40	13	4.24	0.6292
Hex2Cer 18:1;O2/18:0	923.83	51.31	13	816.79	53.92	13	-11.59	0.1628
Hex2Cer 18:1;O2/22:0	149.30	8.14	13	174.81	8.29	13	17.08	0.0387
Hex2Cer 18:1;O2/24:0	618.62	42.80	13	694.89	42.26	13	12.33	0.2175
Hex2Cer 18:1;O2/24:1	1875.27	96.67	13	1856.69	132.74	13	-0.99	0.9103
Hex2Cer 18:1;O2/25:0	271.18	18.55	13	326.80	18.42	13	20.51	0.0435
Hex2Cer 18:1;O2/26:0	68.26	7.02	13	81.46	6.25	13	19.33	0.1727
Hex2Cer 18:1;O2/26:1	578.45	31.63	13	641.13	34.09	13	10.84	0.1893
Total Hex2Cer	4569.03	223.57	13	4680.26	264.78	13	2.43	0.7516

Abbreviations: Hex2Cer Dihexosylceramide, HD Huntington's Disease, SEM Standard Error of Mean, % DIFF Percentage difference of HD compared to controls.

For Review Only

Table S23 Concentrations of sulfatide species in the white matter of the dorsomedial prefrontal cortex. Data was assessed for normality using a D'Agostino Pearson Omnibus test. Data which was normally distributed was analysed using an unpaired t-test with Welch's correction, whilst data that did not fit normality assumptions was analysed using a Mann Whitney U test. Data is in pmol lipid per mg protein.

	CONTROL			HD				
White Cortex	Mean	SEM	N	Mean	SEM	N	% DIFF	P
SHexCer 18:1;O2/16:0	31.91	2.58	13	30.70	2.47	13	-3.79	0.7333
SHexCer 18:1;O2/18:0	1908.87	202.04	13	2647.47	556.30	13	38.69	0.2242
SHexCer 18:1;O2/20:0	731.32	98.83	13	585.41	63.88	13	-19.95	0.2267
SHexCer 18:1;O2/22:0	2023.12	170.22	13	1991.46	142.09	13	-1.56	0.8875
SHexCer 18:1;O2/23:0	3579.60	344.10	13	3428.92	265.10	13	-4.21	0.7318
SHexCer 18:1;O2/24:0	11307.62	1108.88	13	11450.62	832.41	13	1.26	0.9188
SHexCer 18:1;O2/24:1	9793.23	803.03	13	7981.50	630.84	13	-18.50	0.0887
SHexCer 18:1;O2/18:0(2OH)	88.07	7.83	13	90.81	10.43	13	3.11	0.8380
SHexCer 18:1;O2/20:0(2OH)	126.17	6.29	13	134.85	7.47	13	6.88	0.4483
SHexCer 18:1;O2/22:0(2OH)	1707.78	117.07	13	1652.13	87.78	13	-3.26	0.7068
SHexCer 18:1;O2/23:0(2OH)	5002.58	485.30	13	4659.77	393.91	13	-6.85	0.5884
SHexCer 18:1;O2/24:0(2OH)	7228.15	452.68	13	7513.96	338.71	13	3.95	0.6178
SHexCer 18:1;O2/24:1(2OH)	7350.19	484.46	13	6230.12	488.08	13	-15.24	0.1164
Total Sulfatide	29375.66	2636.52	13	28116.08	2298.64	13	-4.29	0.7219
Total OH-Sulfatide	21502.93	1467.28	13	20281.64	1225.73	13	-5.68	0.5292
Total Combined Sulfatide	50878.59	3990.42	13	48397.71	3084.49	13	-4.88	0.6275

Abbreviations: HD Huntington's Disease, SEM Standard Error of Mean, % DIFF Percentage difference of HD compared to controls.

For Review Only

2.5 Grey Cortex

Table S24 Concentrations of ceramide species in the grey matter of the dorsomedial prefrontal cortex. Data was assessed for normality using a D'Agostino Pearson Omnibus test. Data which was normally distributed was analysed using an unpaired t-test with Welch's correction, whilst data that did not fit normality assumptions was analysed using a Mann Whitney U test. Data is in pmol lipid per mg tissue.

	CONTROL			HD				
Grey Cortex	Mean	SEM	N	Mean	SEM	N	% DIFF	P
Cer 18:1;O2/16:0	6.26	1.24	3	4.97	0.32	11	-20.60	0.2253
Cer 18:1;O2/18:0	102.49	5.56	13	105.87	7.17	12	3.30	0.7128
Cer 18:1;O2/20:0	7.89	0.44	13	8.49	0.45	12	7.57	0.3552
Cer 18:1;O2/24:1	61.37	16.19	13	34.39	9.92	12	-43.96	0.1095
Cer 18:1;O2/24:2	16.56	3.95	12	11.66	2.95	7	-29.55	0.4320
Cer 18:1;O2/25:1	12.82	2.90	9	11.22	1.78	5	-12.50	0.6787
Total Ceramide	197.35	26.63	13	164.79	16.49	12	-16.50	0.6114

Abbreviations: Cer Ceramide, HD Huntington's Disease, SEM Standard Error of Mean, % DIFF Percentage difference of HD compared to controls.

For Review Only

Table S25 Concentrations of sphingomyelin species in the grey matter of the dorsomedial prefrontal cortex. Data was assessed for normality using a D'Agostino Pearson Omnibus test. Data which was normally distributed was analysed using an unpaired t-test with Welch's correction, whilst data that did not fit normality assumptions was analysed using a Mann Whitney U test. P values are shown with corresponding significance. Data is in pmol lipid per mg tissue.

	CONTROL			HD				
Grey Cortex	Mean	SEM	N	Mean	SEM	N	% DIFF	P
SM 18:1;O2/16:0	279.17	24.36	13	255.45	26.87	13	-8.50	0.4444
SM 18:1;O2/18:0	3390.80	167.73	13	3173.67	221.87	13	-6.40	0.3686
SM 18:1;O2/18:1	371.56	31.87	13	335.53	32.02	13	-9.70	0.3996
SM 18:1;O2/19:0	43.21	2.59	12	38.34	3.11	11	-11.26	0.2110
SM 18:1;O2/20:0	1046.44	77.12	13	841.07	47.39	13	-19.63	0.0324
SM 18:1;O2/20:1	52.48	3.07	12	45.67	3.12	12	-12.98	0.1228
SM 18:1;O2/22:0	134.20	12.48	13	132.06	21.97	13	-1.59	0.6276
SM 18:1;O2/22:1	78.01	13.42	8	79.22	20.14	8	1.56	0.7812
SM 18:1;O2/23:0	116.26	20.04	12	123.21	40.94	10	5.98	0.6567
SM 18:1;O2/23:1	98.46	20.37	10	139.00	40.31	6	41.17	0.3123
SM 18:1;O2/24:0	271.42	49.27	13	246.84	86.77	13	-9.06	0.3638
SM 18:1;O2/24:1	2006.89	459.82	13	1568.22	530.03	13	-21.86	0.2693
SM 18:1;O2/24:2	101.61	15.42	10	104.07	23.99	9	2.42	0.8000
SM 18:1;O2/25:0	118.06	18.69	10	130.19	45.42	7	10.28	0.8157
SM 18:1;O2/25:1	448.04	104.50	13	361.20	125.46	13	-19.38	0.2914
SM 18:1;O2/26:1	319.49	62.35	12	252.22	88.24	12	-21.05	0.1490
Total Sphingomyelin	7685.35	944.84	13	6730.23	1201.56	13	-12.43	0.2597

For Review Only

Abbreviations: HD Huntington's Disease, SEM Standard Error of Mean, SM Sphingomyelin. % DIFF Percentage difference of HD compared to controls.

For Review Only

Table S26 Concentrations of HexCer species in the grey matter of the dorsomedial prefrontal cortex. Data was assessed for normality using a D'Agostino Pearson Omnibus test. Data which was normally distributed was analysed using an unpaired t-test with Welch's correction, whilst data that did not fit normality assumptions was analysed using a Mann Whitney U test. Data is in pmol lipid per mg protein.

	CONTROL			HD				
Grey Cortex	Mean	SEM	N	Mean	SEM	N	% DIFF	P
Hex-Cer 18:1;O2/16:0	129.25	33.06	13	265.68	78.22	13	105.55	0.1211
Hex-Cer 18:1;O2/18:0	3933.08	1064.10	13	6843.03	2051.33	13	73.99	0.2201
Hex-Cer 18:1;O2/20:0	324.93	76.56	13	675.10	198.88	13	107.77	0.1133
Hex-Cer 18:1;O2/22:0	834.43	236.28	13	1998.84	623.34	13	139.55	0.0935
Hex-Cer 18:1;O2/23:0	1249.45	352.03	13	3127.80	998.12	13	150.33	0.0886
Hex-Cer 18:1;O2/24:0	2786.17	780.36	13	7132.05	2273.72	13	155.98	0.0832
Hex-Cer 18:1;O2/24:1	16183.39	5099.03	13	31372.34	9803.10	13	93.86	0.1820
Hex-Cer 18:1;O2/25:0	957.26	249.14	13	2085.43	646.57	13	117.85	0.1165
Hex-Cer 18:1;O2/26:0	208.33	53.01	13	380.22	111.47	13	82.51	0.1769
Hex-Cer 18:1;O2/26:1	2796.15	863.98	13	5942.17	1868.74	13	112.51	0.5114
Total HexCer	29402.45	8728.61	13	59822.66	18553.60	13	103.46	0.1509

Abbreviations: HexCer Monohexosylceramide, HD Huntington's Disease, SEM Standard Error of Mean, % DIFF Percentage difference of HD compared to controls.

For Review Only

Table S27 Concentrations of Hex2Cer species in the grey matter of the dorsomedial prefrontal cortex. Data was assessed for normality using a D'Agostino Pearson Omnibus test. Data which was normally distributed was analysed using an unpaired t-test with Welch's correction, whilst data that did not fit normality assumptions was analysed using a Mann Whitney U test. P values are shown with corresponding significance. Data is in pmol lipid per mg protein.

	CONTROL			HD				
Grey Cortex	Mean	SEM	N	Mean	SEM	N	% DIFF	P
Hex2Cer 18:1;O2/16:0	38.28	6.70	13	64.00	9.85	13	67.21	0.0410
Hex2Cer 18:1;O2/18:0	311.42	61.00	13	462.32	108.58	13	48.45	0.2382
Hex2Cer 18:1;O2/22:0	39.46	9.12	13	88.55	24.66	13	124.37	0.0740
Hex2Cer 18:1;O2/24:0	110.88	28.50	13	300.86	94.10	13	171.33	0.0650
Hex2Cer 18:1;O2/24:1	505.93	136.75	13	1078.04	327.91	13	113.08	0.1203
Hex2Cer 18:1;O2/25:0	54.07	14.61	13	146.34	47.30	13	170.66	0.0746
Hex2Cer 18:1;O2/26:0	12.44	3.12	13	29.20	9.20	13	134.61	0.0979
Hex2Cer 18:1;O2/26:1	146.31	41.11	13	321.40	101.42	13	119.68	0.1226
Total Hex2Cer	1218.80	298.89	13	2490.70	714.11	13	104.36	0.1135

Abbreviations: Hex2Cer Dihexosylceramide, HD Huntington's Disease, SEM Standard Error of Mean, % DIFF Percentage difference of HD compared to controls.

For Review Only

Table S28 Concentrations of sulfatide species in the grey matter of the dorsomedial prefrontal cortex. Data was assessed for normality using a D'Agostino Pearson Omnibus test. Data which was normally distributed was analysed using an unpaired t-test with Welch's correction, whilst data that did not fit normality assumptions was analysed using a Mann Whitney U test. Data is in pmol lipid per mg protein.

	CONTROL			HD				
Grey Cortex	Mean	SEM	N	Mean	SEM	N	% DIFF	P
SHexCer 18:1;O2/16:0	10.47	1.08	13	14.70	2.91	13	40.39	0.1786
SHexCer 18:1;O2/18:0	278.29	64.34	13	771.46	342.13	13	177.22	0.5114
SHexCer 18:1;O2/20:0	69.66	17.99	13	172.53	55.88	13	147.67	0.0925
SHexCer 18:1;O2/22:0	270.73	63.03	13	635.35	194.29	13	134.68	0.0869
SHexCer 18:1;O2/23:0	420.52	106.43	13	1064.65	344.53	13	153.17	0.0867
SHexCer 18:1;O2/24:0	1350.56	329.45	13	3137.35	985.66	13	132.30	0.0985
SHexCer 18:1;O2/24:1	1492.15	405.33	13	2780.53	841.34	13	86.34	0.1804
SHexCer 18:1;O2/18:0(2OH)	21.59	4.78	13	35.30	10.04	13	63.48	0.2317
SHexCer 18:1;O2/20:0(2OH)	53.63	8.15	13	76.43	14.72	13	42.50	0.1912
SHexCer 18:1;O2/22:0(2OH)	399.41	89.02	13	720.96	199.96	13	80.50	0.1547
SHexCer 18:1;O2/23:0(2OH)	875.32	215.94	13	1855.52	570.02	13	111.98	0.1210
SHexCer 18:1;O2/24:0(2OH)	1786.12	383.31	13	3011.34	828.27	13	68.60	0.1920
SHexCer 18:1;O2/24:1(2OH)	1886.21	499.41	13	2892.17	807.29	13	53.33	0.2998
Total Sulfatide	3892.38	982.58	13	8576.57	2680.78	13	120.34	0.1139
Total OH-Sulfatide	5022.28	1188.05	13	8591.71	2412.36	13	71.07	0.1969
Total Combined Sulfatide	8914.66	2151.03	13	17168.28	5046.26	13	92.58	0.1455

Abbreviations: HD Huntington's Disease, SEM Standard Error of Mean, % DIFF Percentage difference of HD compared to controls.

For Review Only

For Review Only

Table S29 Expression of housekeeping proteins before adjustment for ceramide synthase expression. Data was assessed for normality using a D'Agostino Pearson Omnibus test and then tested using an unpaired t-test with Welch's correction or a Mann Whitney U test where appropriate. **p<0.01, ***p<0.001.

Housekeeper Expression		CON Mean	SEM	N	HD Mean	SEM	N	Normality	*p*	Sig
Caudate (CerS1)	**β** actin	1.067	0.104	12	0.874	0.069	13	N (CON)	0.0868	ns
	GAPDH	0.905	0.049	12	0.973	0.045	12	Y	0.3143	ns
Putamen (CerS1)	**β** actin	1.170	0.139	13	0.654	0.074	13	N (HD)	0.0008	***
	GAPDH	1.025	0.088	13	1.005	0.070	13	Y	0.8582	ns
Caudate (CerS2)	**β** actin	0.954	0.033	12	1.053	0.037	13	Y	0.0581	ns
	GAPDH	1.015	0.053	12	0.995	0.028	13	N (CON)	0.9362	ns
Putamen (CerS2)	**β** actin	1.067	0.038	13	0.916	0.026	13	N (HD)	0.0015	**
	GAPDH	0.994	0.035	13	0.992	0.037	13	Y	0.9749	ns

Abbreviations: CerS1 Ceramide Synthase 1, CerS2 Ceramide Synthase 2, CON Control, HD Huntington's disease, GAPDH Glyceraldehyde 3-phosphate dehydrogenase, SEM Standard Error of Mean, Sig Significance.

For Review Only

For Review Only

Table S30 Pearson's correlations of ceramide synthases with Age at Death for control and HD subjects in the caudate. Ceramide synthase expression adjusted for β-actin for CerS1 and GAPDH for CerS2.

Caudate	N	R	R^2	95% CI	P value
CerS1 vs Age at Death (CON)	12	0.4873	0.2374	-0.1202 to 0.8293	0.1081
CerS1 vs Age at Death (HD)	13	0.7251	0.5258	0.2900 to 0.9118	0.0050**
CerS2 vs Age at Death (CON)	12	-0.2251	0.0507	-0.7076 to 0.4006	0.4818
CerS2 vs Age at Death (HD)	13	0.1279	0.0164	-0.4551 to 0.6342	0.6771

Abbreviations: CerS1 Ceramide Synthase 1, CerS2 Ceramide Synthase 2, CI Confidence Interval, CON Control, HD Huntington's Disease

Table S31 Pearson's correlations of ceramide synthases with Age at Death for control and HD subjects in the putamen. Ceramide synthase expression adjusted for GAPDH.

Putamen	N	R	R^2	95% CI	P value

CerS1 vs Age at Death (CON)	11	0.5401	0.2917	-0.0885 to 0.8610	0.0864
CerS1 vs Age at Death (HD)	12	0.4203	0.1766	-0.2024 to 0.8010	0.1737
CerS2 vs Age at Death (CON)	13	0.4267	0.1820	-0.1625 to 0.7916	0.1460
CerS2 vs Age at Death (HD)	13	0.1829	0.0334	-0.4094 to 0.6667	0.5499

Abbreviations: CerS1 Ceramide Synthase 1, CerS2 Ceramide Synthase 2, CI Confidence Interval, CON Control, HD Huntington's Disease

For Review Only

Supplementary Table S32 Correlations for Additional Banding of CerS2 in HD Subjects. A Pearson's correlation matrix was used to determine relationships. No significant correlations were identified for subjects with additional banding.

Caudate Correlations for CerS2

Pearson (α=0.05)	CerS2 (Primary)	*vs* Age at Death	*vs* Brain pH	*vs* Post-Mortem Interval	*vs* CAG
r		0.4315	-0.1768	-0.0342	-0.3471
95% confidence interval		-0.1567 to 0.7938	-0.6632 to 0.4145	-0.5744 to 0.5267	-0.7539 to 0.2521
R squared		0.1862	0.0313	0.0012	0.1205
P value					
P (two-tailed)		0.1409	0.5633	0.9116	0.2453
Sig.		*ns*	*ns*	*ns*	*ns*
Number of XY Pairs		13	13	13	13
	CerS2 (Secondary)	*vs* Age at Death	*vs* Brain pH	*vs* Post-Mortem Interval	*vs* CAG
r		-0.1706	-0.2279	0.3343	0.2216
95% confidence interval		-0.6596 to 0.4199	-0.6920 to 0.3695	-0.2657 to 0.7476	-0.3752 to 0.6885
R squared		0.02909	0.05194	0.1117	0.04909
P value					
P (two-tailed)		0.5775	0.4539	0.2643	0.4669
Sig.		*ns*	*ns*	*ns*	*ns*
Number of XY Pairs		13	13	13	13
	CerS2 (Tertiary)	*vs* Age at Death	*vs* Brain pH	*vs* Post-Mortem Interval	*vs* CAG
r		0.07784	-0.2094	0.3992	-0.05728
95% confidence interval		-0.4943 to 0.6030	-0.6817 to 0.3861	-0.1946 to 0.7788	-0.5897 to 0.5098
R squared		0.006059	0.04385	0.1593	0.003281
P (two-tailed)		0.8005	0.4923	0.1767	0.8525
Sig.		*ns*	*ns*	*ns*	*ns*
Number of XY Pairs		13	13	13	13

Abbreviations: CerS2 Ceramide Synthase 2, HD Huntington's disease, *ns* not significant, Sig. Significance.

For Review Only
