# Supplementary material for: The long and the short of Huntington’s disease: how the sphingolipid profile is shifted in the caudate of advanced clinical cases
Source: Brain Commun. 2021 Dec 23;4(1):fcab303. doi: 10.1093/braincomms/fcab303 (PMC8833324; doi:10.1093/braincomms/fcab303)
Supplement: fcab303_Supplementary_Data [file fcab303_supplementary_data.zip › Supplementary_Material.pdf]

Supplementary Material

The Long and the Short of Huntington's Disease: How the Sphingolipid Profile is Shifted in the Caudate of Advanced Clinical Cases

Gabrielle R. Phillips[1,2,3], Jennifer T. Saville[4], Sarah E. Hancock[5], Simon HJ. Brown[3,6], Andrew M. Jenner[7], Catriona McLean[8], Maria Fuller[4,9], Kelly A. Newell[1,2,3], Todd Mitchell[1,2,3*]

RUNNING TITLE

Chain length alterations to sphingolipids in Huntington's

AFFLIATIONS

[1]Illawarra Health and Medical Research Institute, Wollongong, 2522, NSW, Australia

[2]School of Medicine, University of Wollongong, Wollongong, 2522, NSW, Australia

[3]Molecular Horizons, University of Wollongong, Wollongong, 2522, NSW, Australia

[4]Genetics and Molecular Pathology, SA Pathology at Women's and Children's Hospital, North Adelaide, 5006, SA, Australia

[5]School of Medical Sciences, University of New South Wales, Sydney, 2052, NSW, Australia

[6]School of Chemistry and Molecular Biosciences, University of Wollongong, Wollongong, 2522, NSW, Australia

[7]Bioanalytical Mass Spectrometry Facility, Mark Wainwright Analytical Centre, University of New South Wales, Sydney, 2052, NSW, Australia

[8]Department of Anatomical Pathology, Alfred Health and Florey Neuroscience, Parkville, 3052, VIC, Australia

[9]Adelaide Medical School, University of Adelaide, Adelaide, 5000, SA, Australia

Supplementary Material 1 Additional Banding of CerS2 in Western Blots

Supplementary bands were identified above the primary CerS2 band (~40 kDa) in HD samples only. To further investigate these bands, two additional experiments were conducted. The first experiment was to check the specificity of the CerS2 antibody using a human CerS2 knockout cell lysate (HEK293T, ab258814, Abcam) as a negative control and a wild type human cell lysate (HEK293T, ab258814, Abcam) as a positive control. All cell and post-mortem samples were loaded at 12.5 µg protein. The knockout blot (**Figure S1a**) supported the specificity of CerS2, showing the presence of CerS2 in wild type cells and the absence in CerS2 knockout cells at or above 40 kDa (**Figure S1b**). The additional banding below 40 kDa appeared in the knockout cells and is therefore unlikely to be a related CerS2 protein and was therefore excluded from the analysis. **Figure S1a** shows the two additional bands which appear only in HD subjects and have a clearer expression in the HD putamen samples. The second experiment was to examine if the reduction of the sample with β-mercaptoethanol would eliminate the additional banding via the breakage of disulphide bonds. HD and control samples were duplicated, with one set reduced with heat (70°C) and β-mercaptoethanol, and the other not. Samples were loaded at 12.5 µg protein. No difference in additional banding above CerS2 primary band was found between the reduced and non-reduced samples indicating their presence is not due to disulphide bonding (**Figure S1b**).

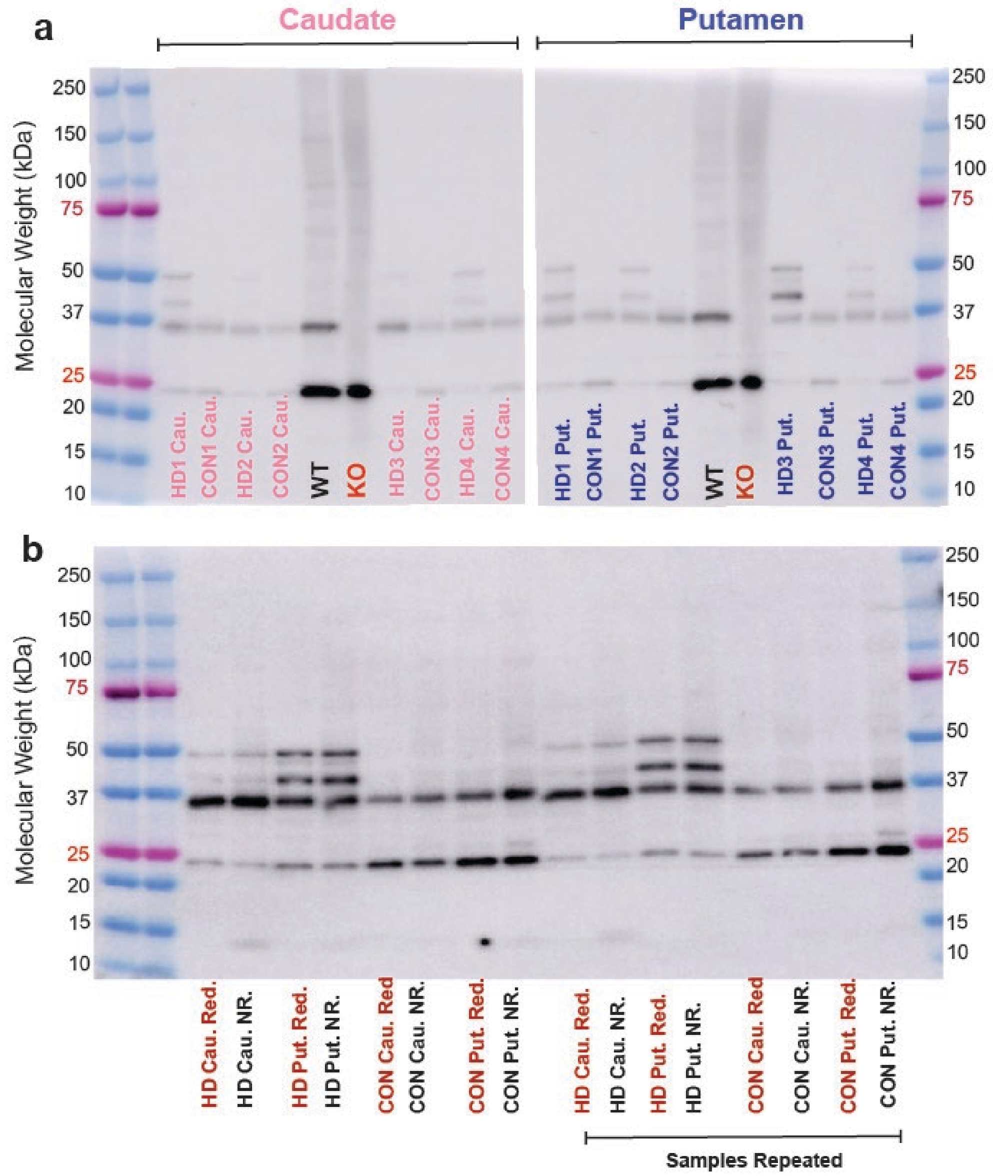

Supplementary Fig. 1 CerS2 Experimental Western Blots (a) CerS2 Knockout Cell Blot. HD and Control samples from the caudate and putamen were loaded alongside CerS2 Knockout and Wild Type Cell Human Lines to check for the specificity of the CerS2 antibody. Samples were loaded at 12.5 µg protein. Knockout cells did not show bands at the molecular weight of CerS2. (b) CerS2 Reduction Blot. To further investigate additional banding of CerS2, samples were both reduced and not-reduced with β-mercaptoethanol to determine if disulphide bonds were present. HD and Control samples from the caudate and putamen were loaded at 12.5 µg in duplicate. Neither condition resulted in the disappearance of additional banding. **CerS2** Ceramide Synthase 2, **CON** Control, **HD** Huntington's Disease, **KO** Knockout, **WT** Wild Type.

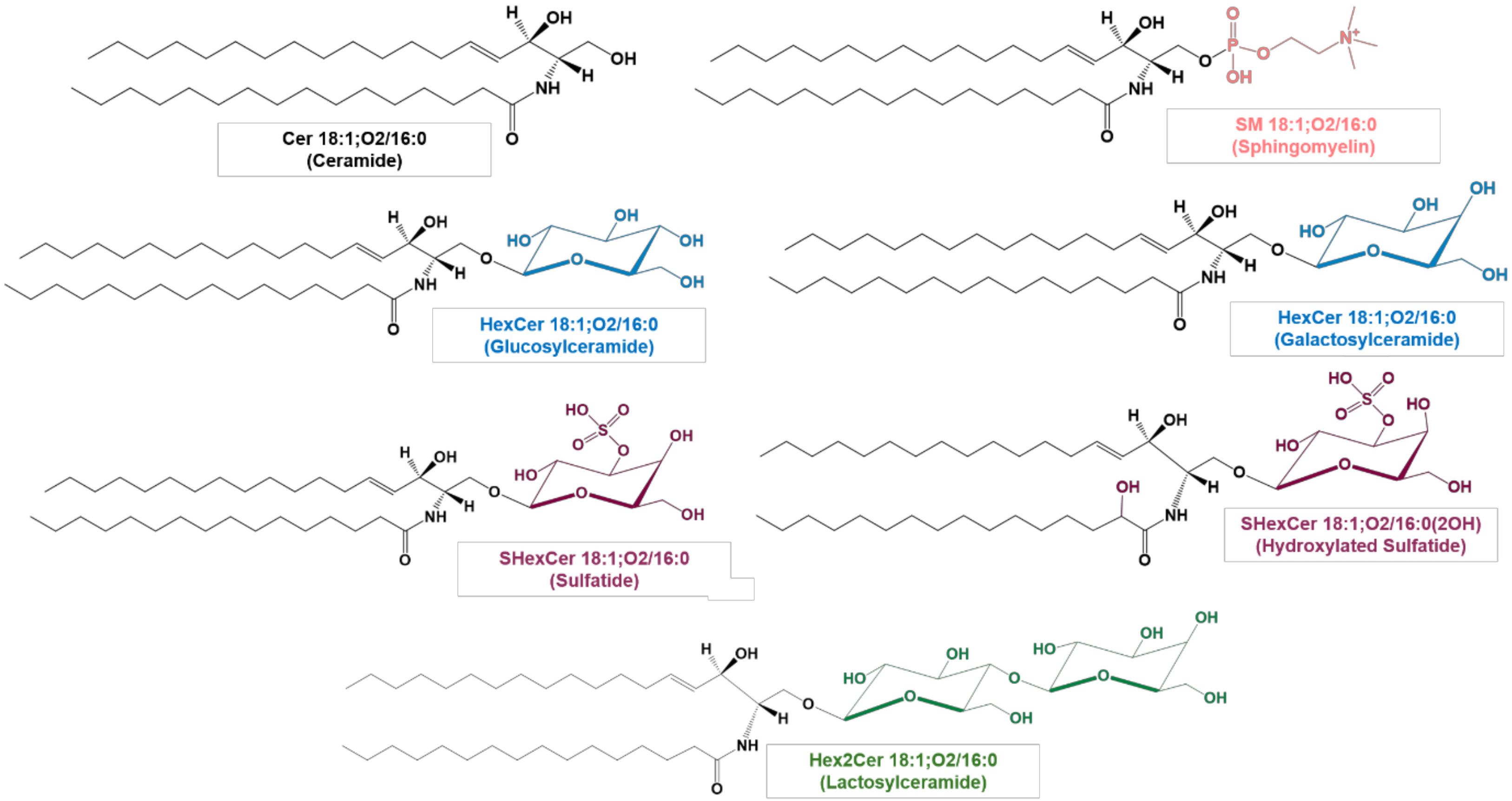

Supplementary Fig. 2 Chemical structure of sphingolipid and glycosphingolipid classes analysed. All lipids are shown to have an 18:1 sphingosine back bone and a 16:0 fatty acyl chain. Structures shown are ceramide (black), sphingomyelin (peach), glucosylceramide and galactosylceramide (monohexosylceramides; blue), sulfatide and hydroxylated sulfatide (magenta), and lactosylceramide (dihexosylceramide, green).

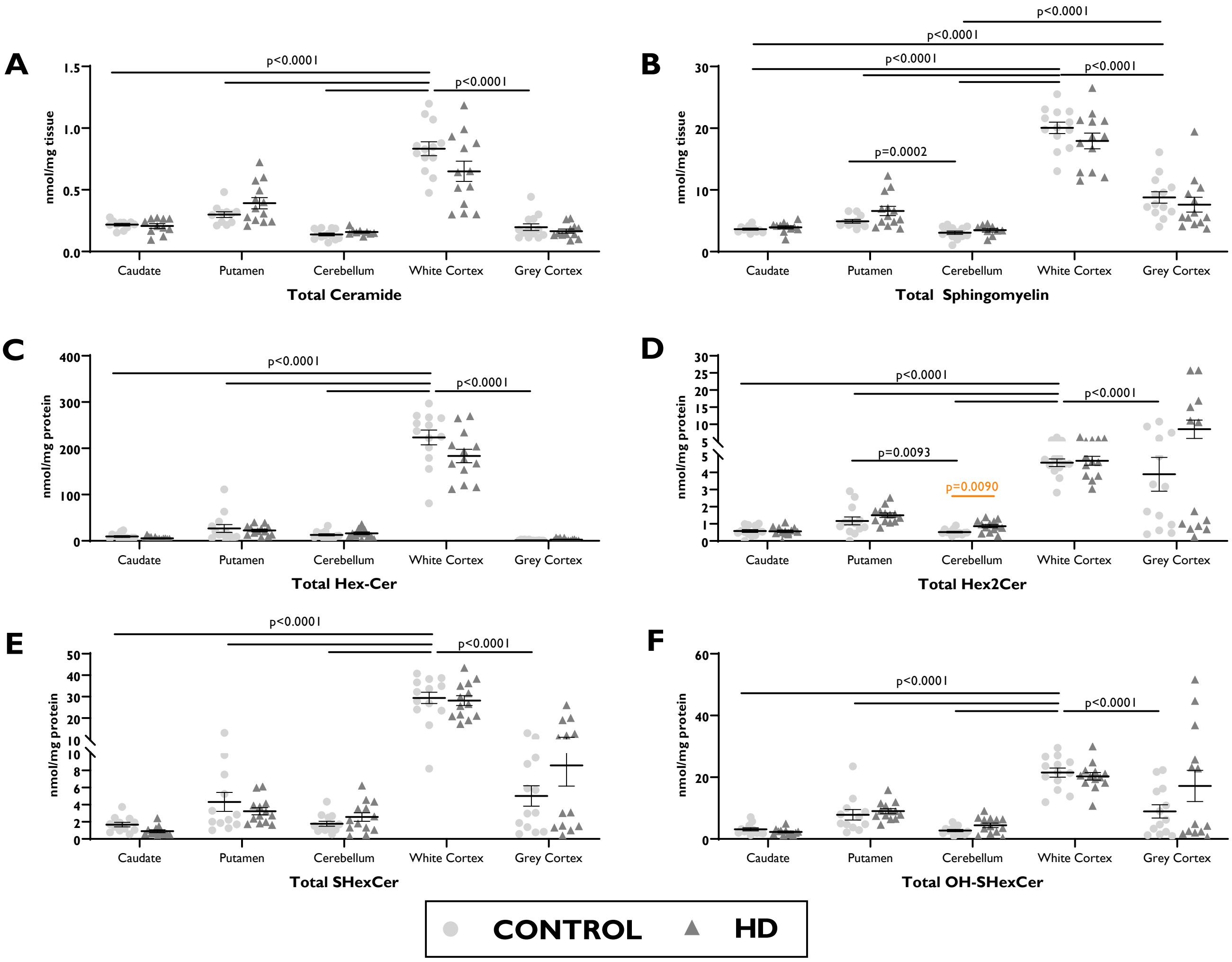

Supplementary Fig. 3 Sphingolipid Class Totals in Control (n=13) *vs* HD (n=12-13) Brain. Bars indicate the mean and standard error of mean. Control regions were compared using a Brown Forsythe ANOVA with a Welch's correction (black bars). Controls were compared to HD subjects using either an unpaired t-test with Welch's correction or a Mann Whitney U test after being assessed for normality with a 'Agostino Pearson Omnibus test (orange bars) . Significance level was set at p<0.01. Exact p values are indicated. **HD** Huntington's Disease.

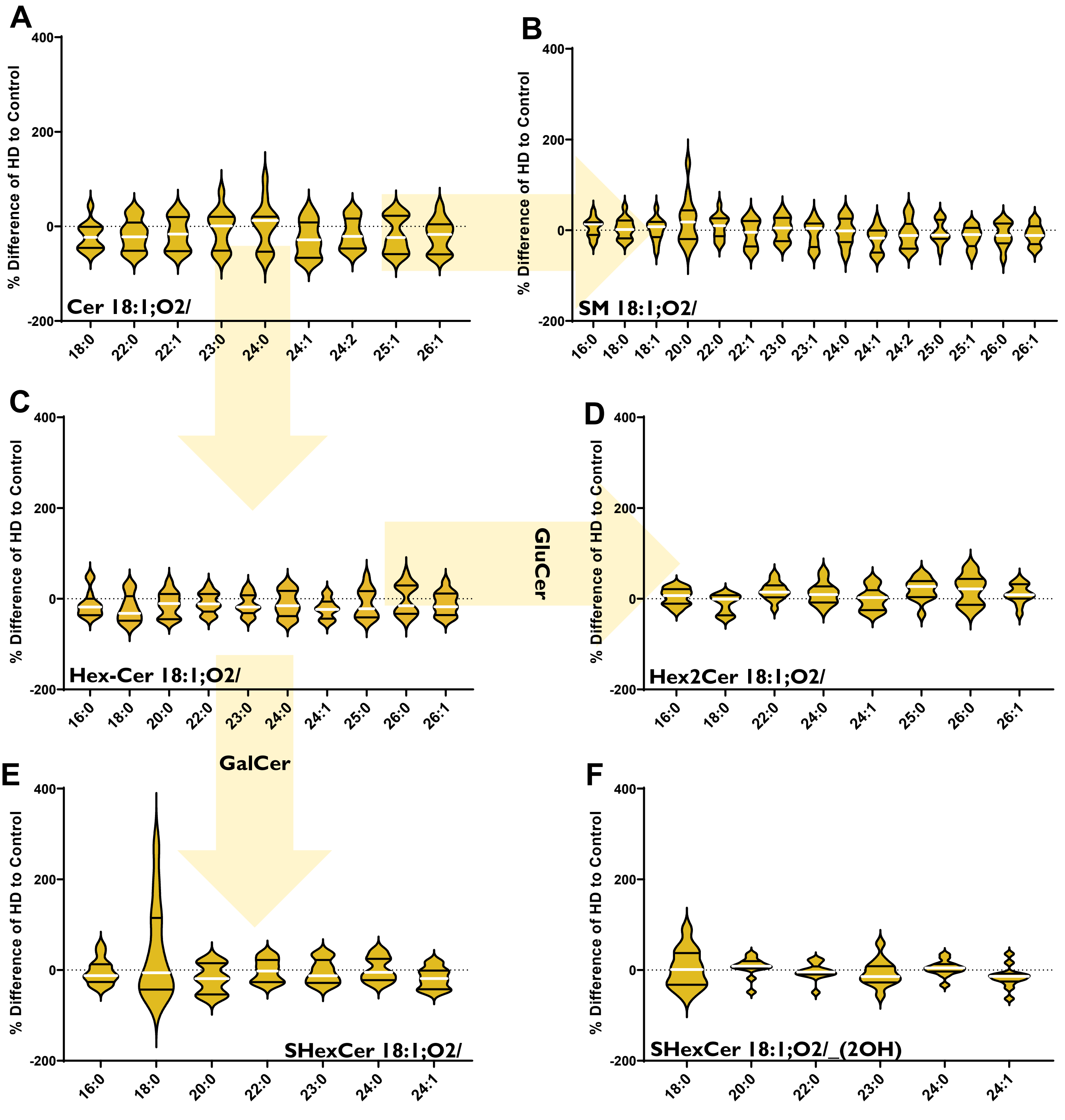

Supplementary Fig. 4 Percentage differences of sphingolipid species in HD subjects to controls for (A) ceramides, (B) sphingomyelins, (C) monohexosylceramides, (D) dihexosylceramides, (E) sulfatides and (F) OH-Sulfatide in the white dmPFC. Arrows indicate metabolic pathways. Violin plot displays spread of values, medians (white line) and quartiles. Data was assessed for normality using a D'Agostino Pearson Omnibus test and analysed using an unpaired t-test or Mann Whitney U test where appropriate. Means, SEM, SD, and exact p values are available in **Supplementary Tables S18-S22**. **Cer** Ceramide, **HD** Huntington's disease, **LacCer** Lactosylceramide, **SM** Sphingomyelin.

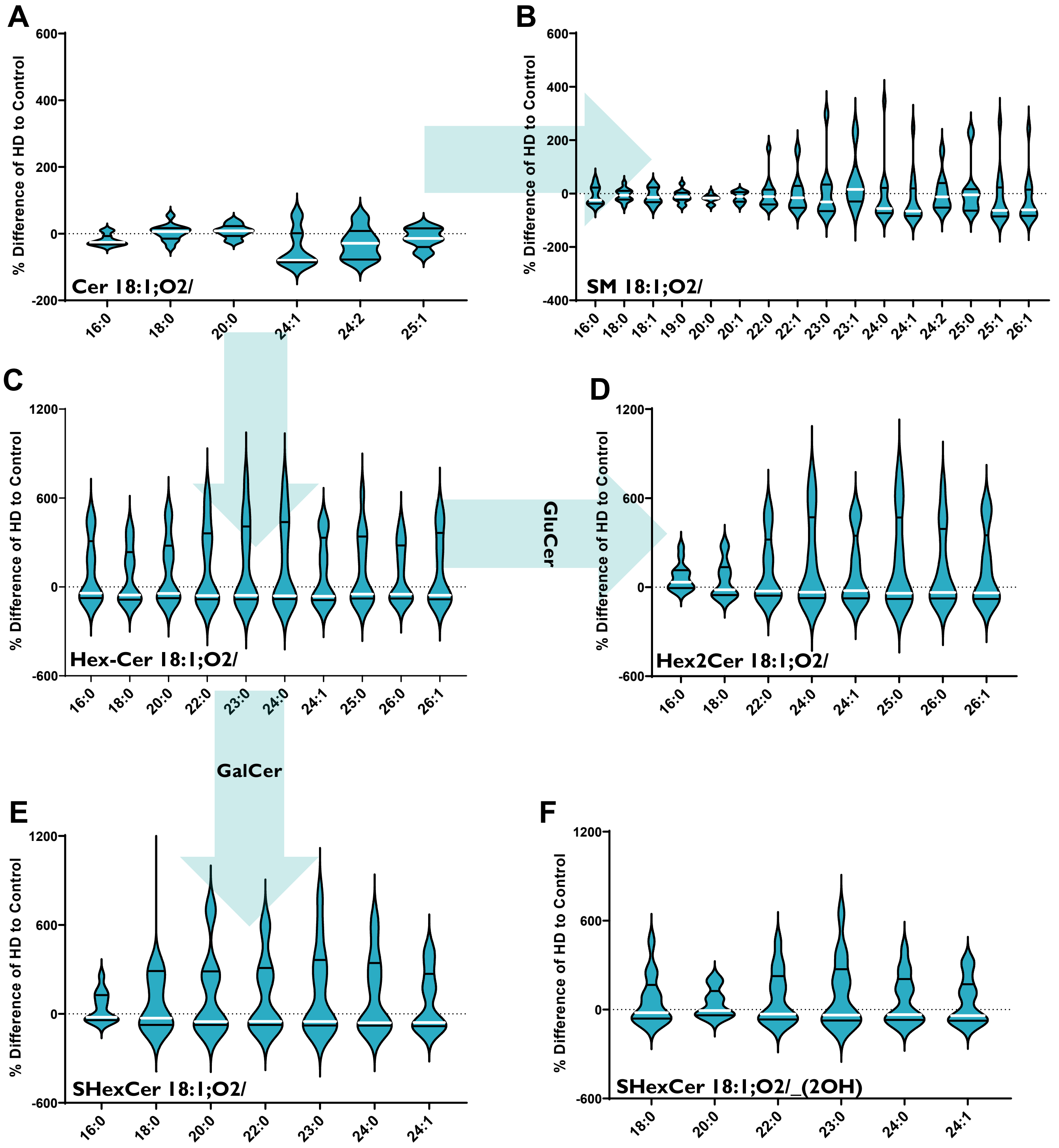

Supplementary Fig. 5 Percentage differences of sphingolipid species in HD subjects to controls for (A) ceramides, (B) sphingomyelins, (C) monohexosylceramides, (D) dihexosylceramides, (E) sulfatides and (F) OH-sulfatide in the grey dmPFC. Arrows indicate metabolic pathways. Violin plot displays spread of values, medians (white line) and quartiles. Data was assessed for normality using a D'Agostino Pearson Omnibus test and analysed using an unpaired t-test or Mann Whitney U test where appropriate. Means, SEM, SD, and exact p values are available in **Supplementary Tables S23-S27**. **Cer** Ceramide, **HD** Huntington's disease, **LacCer** Lactosylceramide, **SM** Sphingomyelin.

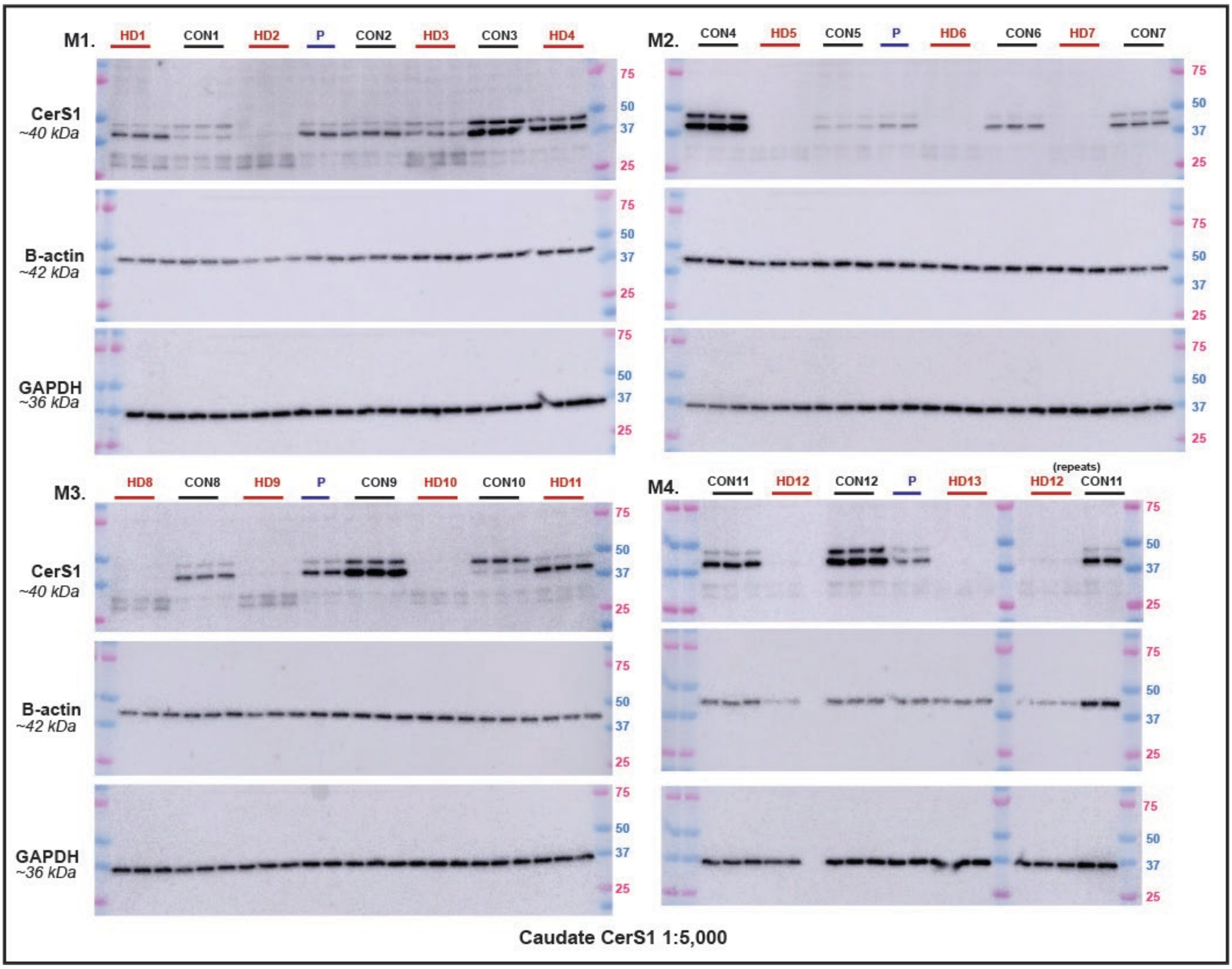

Supplementary Fig. 6 Western Blot of CerS1 (1:5,000; ab131169 [Recombinant]) in Control and HD Caudate. Membranes have been cut to show 25-75 kDa. Western blot of β-actin (1:100,000; MAB1501) and GAPDH (1:50,000; Rb659-060908-WS) have been included below their respective CerS1 membranes. Samples were loaded at 10 µg protein in triplicate. Values were averaged and adjusted for Pool samples and to housekeepers. **CerS1** Ceramide Synthase 1, **CON** Control, **GAPDH** Glyceraldehyde 3-phosphate dehydrogenase, **HD** Huntington's Disease, **P** Pool, **TBST** Tris-Buffered Saline with Tween 20.

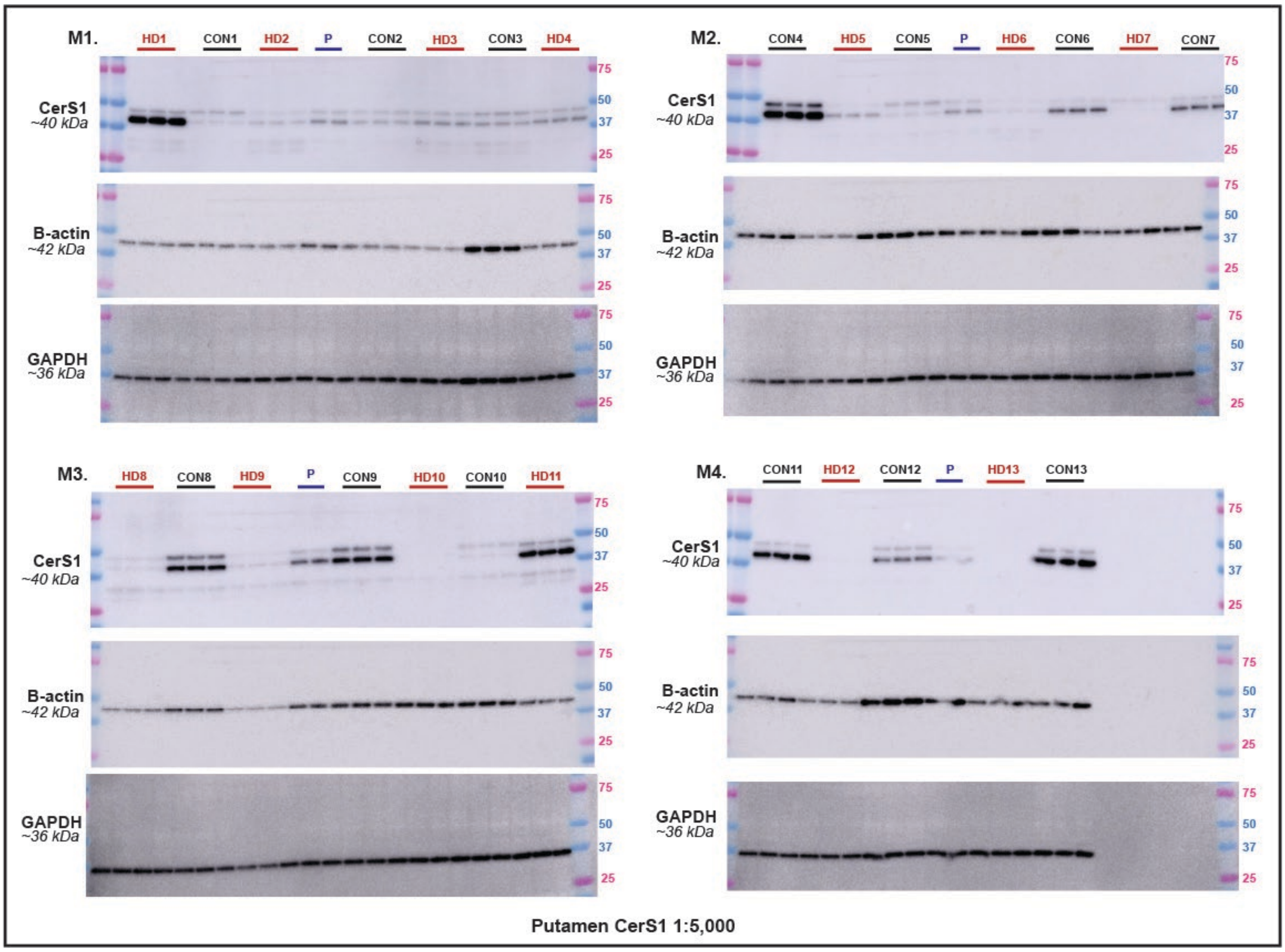

Supplementary Fig. 7 Western Blot of CerS1 (1:5,000; ab131169 [Recombinant]) in Control and HD Putamen. Membranes have been cut to show 25-75 kDa. Western blot of β-actin (1:100,000; MAB1501) and GAPDH (1:50,000; Rb659-060908-WS) have been included below their respective CerS1 membranes. Samples were loaded at 10 μg protein in triplicate. Values were averaged and adjusted for Pool samples and to housekeepers. M1-M4 Indicates Membrane Number. **CerS1** Ceramide Synthase 1, **CON** Control, **GAPDH** Glyceraldehyde 3-phosphate dehydrogenase, **HD** Huntington's Disease, **P** Pool, **TBST** Tris-Buffered Saline with Tween 20.

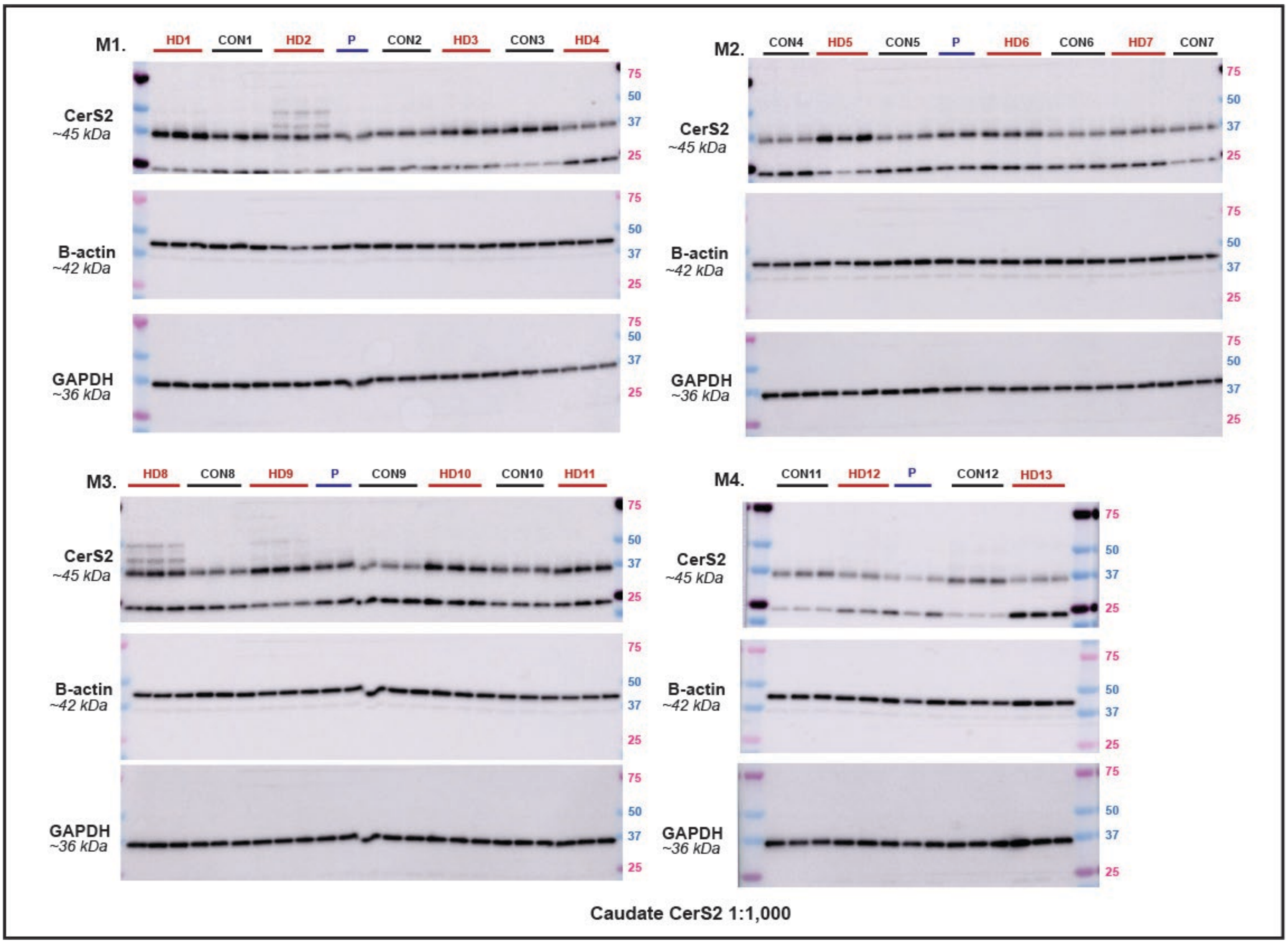

Supplementary Fig. 8 Western Blot of CerS2 (1:1,000; ab176709, Abcam, Cambridge United Kingdom) in Control and HD Caudate. Membranes have been cut to show 25-75 kDa. Western blot of β-actin (1:100,000; MAB1501) and GAPDH (1:50,000; Rb659-060908-WS) have been included below their respective CerS1 membranes. Samples were loaded at 12.5 μg protein in triplicate. Values were averaged and adjusted for Pool samples and to housekeepers. **CerS2** Ceramide Synthase 2, **CON** Control, **GAPDH** Glyceraldehyde 3-phosphate dehydrogenase, **HD** Huntington's Disease, **P** Pool, **TBST** Tris-Buffered Saline with Tween 20.

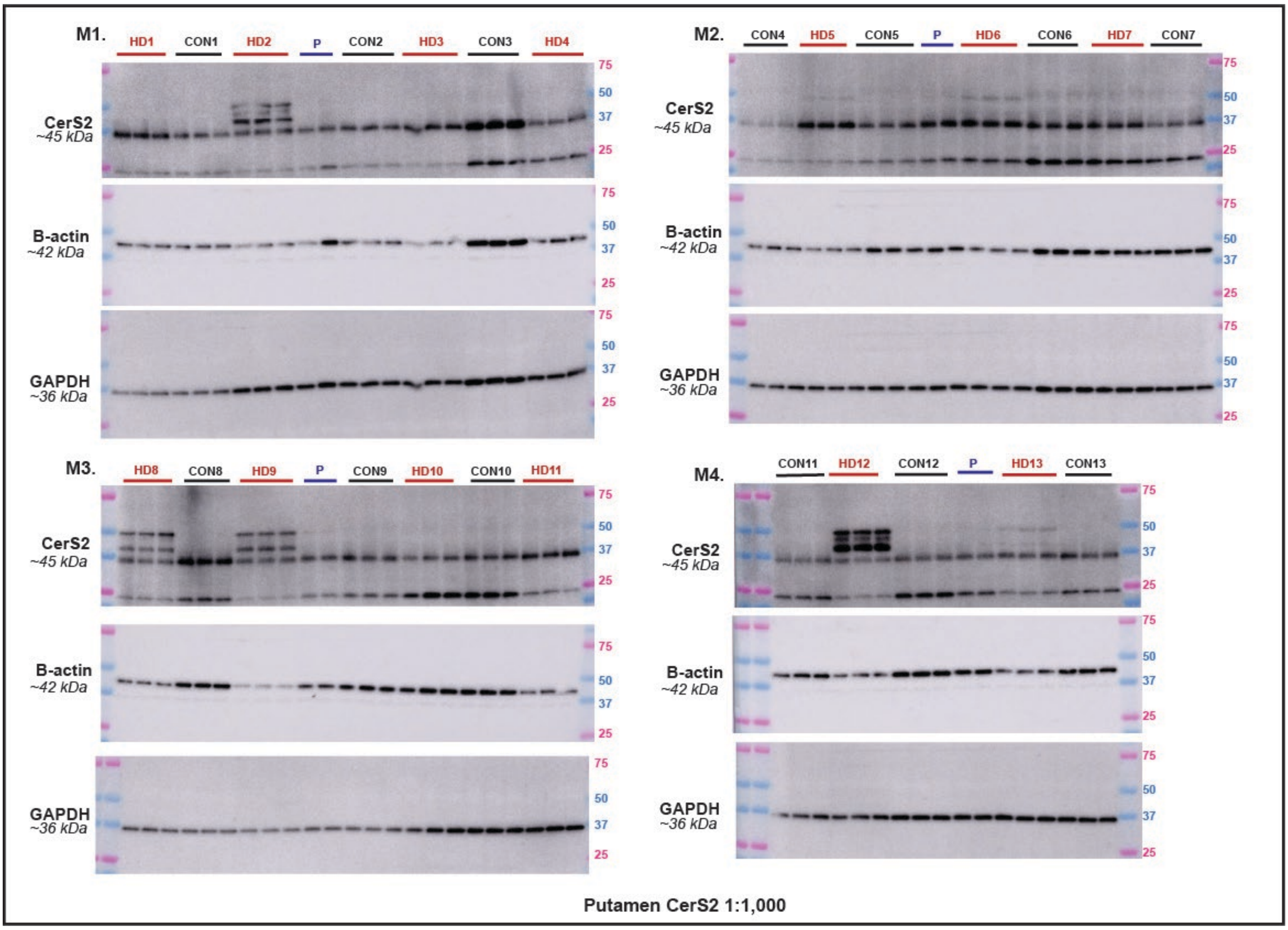

Supplementary Fig. 9 Western Blot of CerS2 (1:1,000; ab176709, Abcam, Cambridge United Kingdom) in Control and HD Putamen. Membranes have been cut to show 25-75 kDa. Western blot of β-actin (1:100,000; MAB1501) and GAPDH (1:50,000; Rb659-060908-WS) have been included below their respective CerS1 membranes. Samples were loaded at 12.5 µg protein in triplicate. Values were averaged and adjusted for Pool samples and to housekeepers. **CerS2** Ceramide Synthase 2, **CON** Control, **GAPDH** Glyceraldehyde 3-phosphate dehydrogenase, **HD** Huntington's Disease, **P** Pool, **TBST** Tris-Buffered Saline with Tween 20

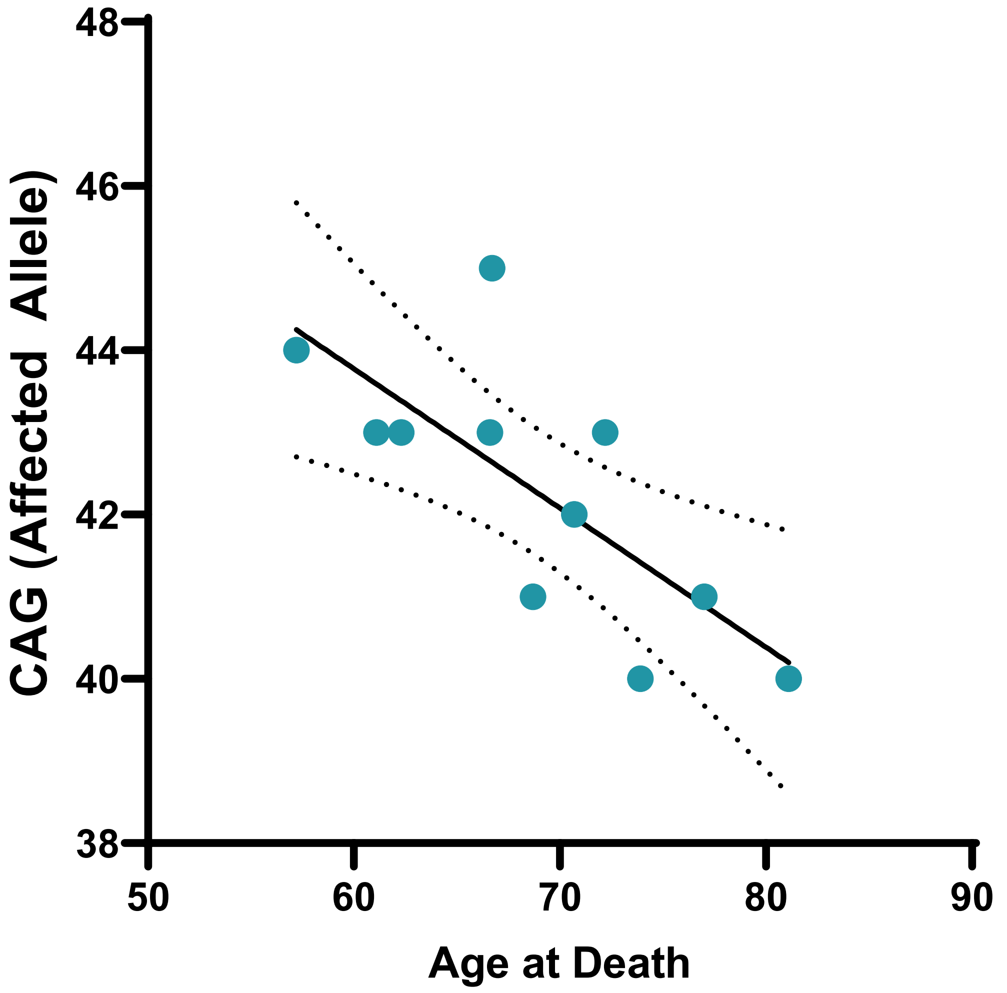

r = -0.7443	r^2 = 0.5540	*p* = 0.0086

Supplementary Fig. 10 Pearson's correlation analysis of Age at Death *vs* CAG repeat length in HD subjects. CAG repeat length was found to have a strong negative correlation with Age of Death (r=-0.7443, p=0.0086). Dotted lines indicate 95% confidence intervals. Two subjects were removed from the analysis due to the cause of death. Only subjects with a clinical history of the cause of death as Huntington's disease were included. Correlations were completed using the disease-specific allele (>39 CAG).

1. Supplementary Tables

1.1 Sphingolipid Nomenclature

Supplementary Table 1 Lipid abbreviations as recommended by Liebisch et al., 2020[45].

Lipid	Abbreviation	Example
Ceramide	Cer	Cer 18:1;O2/16:0
Sphingomyelin	SM	SM 18:1;O2/16:0
Hexosylceramides (glucosylceramides & galactosylceramides)	Hex-Cer	Hex-Cer 18:1;O2/16:0
Dihexosylceramides (Lactosylceramides)	Hex2Cer	Hex2Cer 18:1;O2/16:0
Sulfatides	SHexCer	SHexCer 18:1;O2/16:0
Hydroxylated Sulfatides	SHexCer (2OH)	SHexCer 18:1;O2/16:0(2OH)

1.2 Mass Spectrometry Scans

Supplementary Table 2 Precursor Scans used for Lipid Identifications

Lipid Class	Scan	Collision Energy
Ceramides	+Prec. m/z 184.1	40
Sphingomyelins	+Prec. m/z 264.4	35

Abbreviations: Prec. Precursor.

1.3 Western Blotting

Supplementary Table 3 Antibody Information for Western Blot Experiments

Antibody	Function	Concentration	Supplier
**Anti-Ceramide Synthase 1** (ab131169) [Recombinant] Rabbit Monoclonal	Synthesises Cer C18 species	1:5,000 (2.5% milk in TBST)	Abcam, Cambridge, United Kingdom
**Anti-Ceramide Synthase 2** (ab176709) Rabbit Polyclonal	Synthesises Cer C22-C24 species	1:1,000 (2.5% milk in TBST)	Abcam, Cambridge, United Kingdom
**GAPDH** (Rb659-060908-WS) Rabbit	Housekeeper (Glycolysis)	1:50,000 (1% milk in TBST)	Osenses, Keswick, South Australia, Australia
**β-actin** (MAB1501) Mouse	Housekeeper (Cell Structure)	1:100,000 (1% milk in TBST)	Merck & Co. (Formally Merck Millipore), New Jersey, United States
**Goat x Rabbit Secondary** (AP307P)	Secondary Antibody	1:5,000 (2.5% milk in TBST)	Merck & Co. (Formally Merck Millipore), New Jersey, United States
**Donkey x Mouse Secondary** (AP308P)	Secondary Antibody	1:5,000 (1% milk in TBST)	Merck & Co. (Formally Merck Millipore), New Jersey, United States

Abbreviations: Cer Ceramide, GAPDH Glyceraldehyde 3-phosphate dehydrogenase.

Lipid Concentrations and Statistics

2.1 Controls

Supplementary Table 4 Ceramides in Control Brain Regions. Data was analysed using a Brown Forsythe and Welch's ANOVA with a two-stage linear step-up Benjamini, Krieger and Yekutieli adjustment for multiple comparisons. Both original (p) and adjusted p values (q) are provided. Data is in nmol lipid per mg tissue.

Test details	Mean 1	Mean 2	Mean Diff.	SE of diff.	n1	n2	t	DF	p	q
Caudate vs. Putamen	0.28	0.39	-0.11	0.09	13	13	1.22	23.43	0.2344	0.1183
Caudate vs. Cerebellum	0.28	0.14	0.14	0.06	13	13	2.37	12.87	0.0344	0.0249
Caudate vs. Grey Cortex	0.28	0.20	0.08	0.06	13	13	1.26	16.82	0.2236	0.1183
Caudate vs. White Cortex	0.28	0.83	-0.56	0.08	13	13	6.84	23.98	<0.0001	<0.0001
Putamen vs. Cerebellum	0.39	0.14	0.25	0.07	13	13	3.61	12.63	0.0033	0.0033
Putamen vs. Grey Cortex	0.39	0.20	0.19	0.07	13	13	2.60	15.59	0.0196	0.0165
Putamen vs. White Cortex	0.39	0.83	-0.45	0.09	13	13	5.04	23.23	<0.0001	<0.0001
Cerebellum vs. Grey Cortex	0.14	0.20	-0.06	0.03	13	13	2.05	16.01	0.0569	0.0359
Cerebellum vs. White Cortex	0.14	0.83	-0.70	0.06	13	13	12.05	12.91	<0.0001	<0.0001
Grey Cortex vs. White Cortex	0.20	0.83	-0.64	0.06	13	13	10.17	17.06	<0.0001	<0.0001

Abbreviations: DF degrees of freedom; **Diff.** Difference; **SE of diff** Standard Error of Difference.

Supplementary Table 5 Sphingomyelin in Control Brain Regions. Data was analysed using a Brown Forsythe and Welch's ANOVA with a two-stage linear step-up Benjamini, Krieger and Yekutieli adjustment for multiple comparisons. Both original (p) and adjusted p values (q) are provided. Data is in nmol lipid per mg tissue.

Test details	Mean 1	Mean 2	Mean Diff.	SE of diff.	n1	n2	t	DF	p	q
Caudate vs. Putamen	3.64	4.91	-1.26	0.34	11	11	3.74	15.50	0.0019	0.0002
Caudate vs. Cerebellum	3.64	3.07	0.57	0.31	11	13	1.84	19.40	0.0807	0.0082
Caudate vs. Grey Cortex	3.64	8.79	-5.15	0.94	11	13	5.46	12.73	0.0001	<0.0001
Caudate vs. White Cortex	3.64	20.06	-16.42	0.94	11	13	17.56	12.74	<0.0001	<0.0001
Putamen vs. Cerebellum	4.91	3.07	1.84	0.40	11	13	4.62	21.14	0.0001	<0.0001
Putamen vs. Grey Cortex	4.91	8.79	-3.88	0.98	11	13	3.98	14.38	0.0013	0.0002
Putamen vs. White Cortex	4.91	20.06	-15.15	0.97	11	13	15.67	14.43	<0.0001	<0.0001
Cerebellum vs. Grey Cortex	3.07	8.79	-5.72	0.97	13	13	5.92	13.94	<0.0001	<0.0001
Cerebellum vs. White Cortex	3.07	20.06	-16.99	0.96	13	13	17.73	13.98	<0.0001	<0.0001
Grey Cortex vs. White Cortex	8.79	20.06	-11.27	1.31	13	13	8.62	24.00	<0.0001	<0.0001

Abbreviations: DF degrees of freedom; **Diff.** Difference; **SE of diff** Standard Error of Difference.

Supplementary Table 6 Hex-Cer in Control Brain Regions. Data was analysed using a Brown Forsythe and Welch's ANOVA with a two-stage linear step-up Benjamini, Krieger and Yekutieli adjustment for multiple comparisons. Both original (p) and adjusted p values (q) are provided. Data is in nmol lipid per mg protein.

Test details	Mean 1	Mean 2	Mean Diff.	SE of diff.	n1	n2	t	DF	p	q
Caudate vs. Putamen	10.22	28.98	-18.75	9.08	12	12	2.07	11.93	0.0614	0.0620
Caudate vs. Cerebellum	10.22	12.89	-2.66	2.82	12	13	0.94	22.71	0.3554	0.2393
Caudate vs. Grey Cortex	10.22	29.40	-19.18	8.92	12	13	2.15	13.05	0.0508	0.0616
Caudate vs. White Cortex	10.22	223.50	-213.20	16.18	12	13	13.18	12.31	<0.0001	<0.0001
Putamen vs. Cerebellum	28.98	12.89	16.09	9.15	12	13	1.76	12.28	0.1035	0.0784
Putamen vs. Grey Cortex	28.98	29.40	-0.43	12.46	12	13	0.03	22.91	0.973	0.5896
Putamen vs. White Cortex	28.98	223.50	-194.50	18.37	12	13	10.59	18.57	<0.0001	<0.0001
Cerebellum vs. Grey Cortex	12.89	29.40	-16.52	8.99	13	13	1.84	13.44	0.0883	0.0765
Cerebellum vs. White Cortex	12.89	223.50	-210.60	16.22	13	13	12.99	12.43	<0.0001	<0.0001
Grey Cortex vs. White Cortex	29.40	223.50	-194.10	18.29	13	13	10.61	18.51	<0.0001	<0.0001

Abbreviations: DF degrees of freedom; **Diff.** Difference; **SE of diff** Standard Error of Difference.

Supplementary Table 7 Hex2Cer in Control Brain Regions. Data was analysed using a Brown Forsythe and Welch's ANOVA with a two-stage linear step-up Benjamini, Krieger and Yekutieli adjustment for multiple comparisons. Both original (p) and adjusted p values (q) are provided. Data is in nmol lipid per mg protein.

Test details	Mean 1	Mean 2	Mean Diff.	SE of diff.	n1	n2	t	DF	p	q
Caudate vs. Putamen	0.63	1.27	-0.64	0.24	12	12	2.66	13.35	0.0194	0.0196
Caudate vs. Cerebellum	0.63	0.52	0.11	0.09	12	13	1.22	18.25	0.2391	0.1610
Caudate vs. Grey Cortex	0.63	1.22	-0.59	0.31	12	13	1.91	13.51	0.0770	0.0584
Caudate vs. White Cortex	0.63	4.57	-3.94	0.24	12	13	16.71	14.66	<0.0001	<0.0001
Putamen vs. Cerebellum	1.27	0.52	0.75	0.23	12	13	3.20	11.86	0.0077	0.0093
Putamen vs. Grey Cortex	1.27	1.22	0.05	0.38	12	13	0.13	21.95	0.8963	0.5432
Putamen vs. White Cortex	1.27	4.57	-3.30	0.32	12	13	10.32	22.90	<0.0001	<0.0001
Cerebellum vs. Grey Cortex	0.52	1.22	-0.70	0.30	13	13	2.31	12.55	0.0390	0.0337
Cerebellum vs. White Cortex	0.52	4.57	-4.05	0.23	13	13	17.74	12.98	<0.0001	<0.0001
Grey Cortex vs. White Cortex	1.22	4.57	-3.35	0.37	13	13	8.98	22.23	<0.0001	<0.0001

Abbreviations: DF degrees of freedom; **Diff.** Difference; **SE of diff** Standard Error of Difference.

Supplementary Table 8 SHexCer in Control Brain Regions. Data was analysed using a Brown Forsythe and Welch's ANOVA with a two-stage linear step-up Benjamini, Krieger and Yekutieli adjustment for multiple comparisons. Both original (p) and adjusted p values (q) are provided. Data is in nmol lipid per mg protein.

Test details	Mean 1	Mean 2	Mean Diff.	SE of diff.	n1	n2	t	DF	p	q
Caudate vs. Putamen	1.67	4.32	-2.65	1.14	12	12	2.32	12.16	0.0386	0.0406
Caudate vs. Cerebellum	1.67	1.77	-0.10	0.39	12	13	0.25	22.86	0.8052	0.4879
Caudate vs. Grey Cortex	1.67	3.89	-2.22	1.02	12	13	2.19	13.62	0.0469	0.0406
Caudate vs. White Cortex	1.67	29.38	-27.70	2.65	12	13	10.46	12.23	<0.0001	<0.0001
Putamen vs. Cerebellum	4.32	1.77	2.55	1.15	12	13	2.22	12.49	0.0458	0.0406
Putamen vs. Grey Cortex	4.32	3.89	0.43	1.49	12	13	0.29	22.37	0.7753	0.4879
Putamen vs. White Cortex	4.32	29.38	-25.05	2.86	12	13	8.76	16.10	<0.0001	<0.0001
Cerebellum vs. Grey Cortex	1.77	3.89	-2.12	1.02	13	13	2.07	14.07	0.0572	0.0433
Cerebellum vs. White Cortex	1.77	29.38	-27.61	2.65	13	13	10.41	12.29	<0.0001	<0.0001
Grey Cortex vs. White Cortex	3.89	29.38	-25.48	2.81	13	13	9.06	15.27	<0.0001	<0.0001

Abbreviations: DF degrees of freedom; **Diff.** Difference; **SE of diff** Standard Error of Difference.

Supplementary Table 9 OH-SHexCer in Control Brain Regions. Data was analysed using a Brown Forsythe and Welch's ANOVA with a two-stage linear step-up Benjamini, Krieger and Yekutieli adjustment for multiple comparisons. Both original (p) and adjusted p values (q) are provided. Data is in nmol lipid per mg protein.

Test details	Mean 1	Mean 2	Mean Diff.	SE of diff.	n1	n2	t	DF	p	q
Caudate vs. Putamen	3.11	7.80	-4.70	1.77	12	12	2.65	12.93	0.0201	0.0203
Caudate vs. Cerebellum	3.11	2.73	0.37	0.62	12	13	0.61	19.97	0.5510	0.3339
Caudate vs. Grey Cortex	3.11	5.02	-1.92	1.29	12	13	1.48	16.15	0.1572	0.1191
Caudate vs. White Cortex	3.11	21.50	-18.40	1.55	12	13	11.85	14.78	<0.0001	<0.0001
Putamen vs. Cerebellum	7.80	2.73	5.07	1.74	12	13	2.92	11.95	0.0128	0.0156
Putamen vs. Grey Cortex	7.80	5.02	2.78	2.07	12	13	1.34	20.00	0.1947	0.1311
Putamen vs. White Cortex	7.80	21.50	-13.70	2.25	12	13	6.10	22.21	<0.0001	<0.0001
Cerebellum vs. Grey Cortex	2.73	5.02	-2.29	1.24	13	13	1.85	14.09	0.0859	0.0744
Cerebellum vs. White Cortex	2.73	21.50	-18.77	1.51	13	13	12.44	13.38	<0.0001	<0.0001
Grey Cortex vs. White Cortex	5.02	21.50	-16.48	1.89	13	13	8.73	23.00	<0.0001	<0.0001

Abbreviations: DF degrees of freedom; **Diff.** Difference; **SE of diff** Standard Error of Difference.

2.1 Caudate

Supplementary Table 10 Concentrations of ceramide species in the caudate. Data was assessed for normality using a D'Agostino Pearson Omnibus test. Data which was normally distributed was analysed using an unpaired t-test with Welch's correction, whilst data that did not fit normality assumptions was analysed using a Mann Whitney U test. P values are shown with corresponding significance (**p<0.01). Data is in pmol lipid per mg tissue. The adjusted p values (q values) for multiple comparisons corrections using a two-stage linear step-up Benjamini, Krieger, and Yekutieli method have been included for the readers information.

	CON			HD								
	**Mean**	**SEM**	**n**	**Mean**	**SEM**	**n**	**PD (%)**	**t ratio**	**df**	**MW-U**	**p**	**q**
Cer d18:1/16:0	6.16	0.33	11	10.27	1.27	11	66.59	3.12	11.37		0.0095**	0.2804
Cer d18:1/18:0	131.89	8.84	11	134.30	14.31	11	1.83	0.14	16.66		0.8879	0.9998
Cer d18:1/19:0	2.26	0.12	10	1.89	0.30	6	-16.42			11.00	0.0420	0.5550
Cer d18:1/20:0	11.96	0.72	11	11.85	1.10	11	-0.92	0.09	17.27		0.9343	0.9998
Cer d18:1/21:0	2.31	0.31	10	3.00	0.32	10	30.16	1.58	17.99		0.1330	0.9201
Cer d18:1/22:0	5.20	0.33	11	5.37	0.66	11	3.31			55.00	0.6994	0.9775
Cer d18:1/22:1	3.24	0.20	11	2.28	0.19	9	-29.56	3.44	17.86		0.0026**	0.1109
Cer d18:1/22:2	2.28	0.32	9	1.52	0.13	6	-33.13			8.50	0.0256	0.5004
Cer d18:1/23:0	3.85	0.19	11	3.74	0.21	10	-2.96	0.38	18.58		0.6932	0.9998
Cer d18:1/24:0	3.54	0.25	11	3.53	0.29	11	-0.24	0.02	19.63		0.9828	0.9998
Cer d18:1/24:1	34.22	2.42	11	24.48	2.01	11	-28.48	3.10	19.34		0.0059**	0.1895
Cer d18:1/24:2	10.14	0.91	11	5.94	0.62	11	-41.42	3.79	17.55		0.0013**	0.0588
Cer d18:1/25:0	1.75	0.18	9	1.59	0.24	7	-9.13			24.00	0.3652	0.9775
**Total Ceramide**	**217.65**	**10.64**	**11**	**206.60**	**18.71**	**11**	**-5.08**	**0.51**	**15.86**		**0.6149**	0.9995

Abbreviations: df degrees of freedom; **HD** Huntington's Disease; **MW-U** Mann Whitney U Rank; **PD** Percentage Difference (HD to Control); **SEM** Standard Error of Mean.

Supplementary Table 11 Concentrations of sphingomyelin species in the caudate. Data was assessed for normality using a D'Agostino Pearson Omnibus test. Data which was normally distributed was analysed using an unpaired t-test with Welch's correction, whilst data that did not fit normality assumptions was analysed using a Mann Whitney U test. P values are shown with corresponding significance (**p<0.01, ***p<0.001). Data is in nmol lipid per mg tissue. The adjusted p values (q values) for multiple comparisons corrections using a two-stage linear step-up Benjamini, Krieger, and Yekutieli method have been included for the readers information.

	CON			HD								
**Caudate**	**Mean**	**SEM**	**n**	**Mean**	**SEM**	**n**	**PD (%)**	**t ratio**	**df**	**MW-U**	**p**	**q**
SM d18:1/14:0	8.49	0.59	11	10.74	1.25	11	26.49	1.65	14.28		0.1178	0.9121
SM d18:1/15:0	3.85	0.79	10	7.50	0.76	11	95.05			11.50	0.0011**	0.0376
SM d18:1/16:0	219.90	16.93	11	321.63	23.00	11	46.26			13.00	0.0010**	0.0338
SM d18:1/16:1	6.47	0.40	11	8.96	0.60	11	38.40	3.41	17.34		0.0026**	0.1188
SM d18:1/17:0	28.07	1.68	11	49.23	4.32	11	75.38	4.57	12.95		0.0002***	0.0231
SM d18:1/18:0	2074.25	100.61	11	2478.91	169.08	11	19.51	2.06	16.29		0.0530	0.7637
SM d18:1/18:1	194.40	11.94	11	264.34	17.90	11	35.98	3.25	17.43		0.0040**	0.1565
SM d18:1/19:0	29.57	1.38	11	34.00	2.66	11	14.99	1.48	15.04		0.1546	0.9468
SM d18:1/20:1	26.22	1.98	11	23.65	2.91	11	-9.81	0.73	17.62		0.4725	0.9993
SM d18:1/21:0	7.79	0.49	9	7.49	0.60	10	-3.83	0.36	16.61		0.7104	0.9998
SM d18:1/22:0	52.40	3.23	11	61.61	4.02	11	17.58	1.78	19.11		0.0892	0.8629
SM d18:1/22:1	19.05	1.94	11	21.39	2.26	11	12.26			47.00	0.4009	0.9775
SM d18:1/23:0	32.16	2.02	11	25.91	2.36	11	-19.42	2.01	19.55		0.0582	0.7654
SM d18:1/23:1	25.98	2.07	11	22.56	1.95	11	-13.15			43.50	0.2703	0.9747
SM d18:1/24:0	80.34	5.45	11	54.06	4.78	11	-32.72	3.62	19.68		0.0017**	0.0700
SM d18:1/24:1	536.40	45.49	11	351.00	30.87	11	-34.56	3.37	17.60		0.0030**	0.1239
SM d18:1/24:2	26.33	2.43	11	22.55	1.44	11	-14.36	1.34	16.26		0.1963	0.9645
SM d18:1/25:0	28.55	2.26	11	16.25	1.32	11	-43.08	4.69	16.14		0.0001***	0.0108
SM d18:1/25:1	139.09	13.38	11	94.36	12.53	11	-32.16	2.44	19.91		0.0241	0.5431
SM d18:1/26:0	7.81	0.61	11	5.57	1.27	9	-28.61			21.00	0.0310	0.5307
SM d18:1/26:1	88.81	7.29	11	54.79	5.96	11	-38.31	3.62	19.24		0.0017**	0.0718
SM d18:1/26:2	8.18	0.60	11	7.34	0.99	10	-10.29	0.75	15.12		0.4666	0.9993
**Total SM**	**3642.34**	**162.19**	**11**	**3941.48**	**257.29**	**11**	**8.21**	**0.98**	**16.86**		**0.3371**	0.9954

Abbreviations: df degrees of freedom; **HD** Huntington's Disease; **MW-U** Mann Whitney U Rank; **PD** Percentage Difference (HD to Control); **SEM** Standard Error of Mean.

Supplementary Table 12 Concentrations of HexCer species in caudate. Data was assessed for normality using a D'Agostino Pearson Omnibus test. Data which was normally distributed was analysed using an unpaired t-test with Welch's correction, whilst data that did not fit normality assumptions was analysed using a Mann Whitney U test. P values are shown with corresponding significance. Data is in pmol lipid per mg protein. The adjusted p values (q values) for multiple comparisons corrections using a two-stage linear step-up Benjamini, Krieger, and Yekutieli method have been included for the readers information.

	CON			HD								
	**Mean**	**SEM**	**n**	**Mean**	**SEM**	**n**	**PD (%)**	**t ratio**	**df**	**MW-U**	**p**	**q**
HexCer d18:1/16:0	64.34	9.25	12	65.52	8.75	12	1.83			72.0	0.9999	>0.9999
HexCer d18:1/18:0	1609.68	275.83	12	962.82	146.32	12	-40.19			43.0	0.1005	0.8515
HexCer d18:1/20:0	165.31	27.29	12	104.90	15.02	12	-36.55	1.94	17.11		0.0664	0.8073
HexCer d18:1/22:0	278.12	47.45	12	163.78	26.49	12	-41.11	2.10	17.25		0.0471	0.7484
HexCer d18:1/23:0	436.51	70.58	12	248.25	41.80	12	-43.13	2.30	17.87		0.0316	0.6464
HexCer d18:1/24:0	990.11	161.44	12	549.23	100.18	12	-44.53			39.0	0.0597	0.7252
HexCer d18:1/24:1	5127.63	1028.27	12	2682.25	402.95	12	-47.69	2.21	14.30		0.0375	0.7250
HexCer d18:1/25:0	424.00	65.56	12	256.05	44.15	12	-39.61			39.0	0.0597	0.7252
HexCer d18:1/26:0	101.36	15.03	12	66.59	11.04	12	-34.30			44.0	0.1100	0.8710
HexCer d18:1/26:1	1026.13	163.48	12	700.32	101.35	12	-31.75	1.69	18.37		0.1041	0.8964
**Total HexCer**	**10223.18**	**1833.50**	**12**	**5353.58**	**929.11**	**13**	**-47.63**	**2.17**	**15.87**		**0.0453**	**0.7272**

Abbreviations: df degrees of freedom; **HD** Huntington's Disease; **MW-U** Mann Whitney U Rank; **PD** Percentage Difference (HD to Control); **SEM** Standard Error of Mean.

Supplementary Table 13 Concentrations of Hex2Cer species in the caudate. Data was assessed for normality using a D'Agostino Pearson Omnibus test. Data which was normally distributed was analysed using an unpaired t-test with Welch's correction, whilst data that did not fit normality assumptions was analysed using a Mann Whitney U test. P values are shown with corresponding significance (**p<0.01, ***p<0.001). Data is in pmol lipid per mg protein. The adjusted p values (q values) for multiple comparisons corrections using a two-stage linear step-up Benjamini, Krieger, and Yekutieli method have been included for the readers information.

	CON			HD								
**Caudate**	**Mean**	**SEM**	**n**	**Mean**	**SEM**	**n**	**PD (%)**	**t ratio**	**df**	**MW-U**	**p**	**q**
Hex2Cer d18:1/16:0	28.97	6.21	12	39.31	3.81	12	35.67			26.0	0.0058**	0.1909
Hex2Cer d18:1/18:0	143.25	13.66	12	206.37	22.31	12	44.07	2.41	18.23		0.0246	0.5652
Hex2Cer d18:1/22:0	21.15	2.59	12	24.02	2.76	12	13.56	0.76	21.91		0.4767	0.9993
Hex2Cer d18:1/24:0	56.21	7.16	12	42.78	5.94	12	-23.90			49.0	0.1926	0.9543
Hex2Cer d18:1/24:1	266.87	39.10	12	211.11	35.91	12	-20.89			57.5	0.0001***	0.9775
Hex2Cer d18:1/25:0	26.64	3.49	12	19.37	3.16	12	-27.28			39.5	0.0656	0.7252
Hex2Cer d18:1/26:0	8.05	0.92	12	6.41	0.89	12	-20.38	1.29	21.98		0.2149	0.9645
Hex2Cer d18:1/26:1	77.75	11.24	12	63.97	10.44	12	-17.73			56.0	0.3777	0.9775
**Total Hex2Cer**	**628.89**	**75.14**	**12**	**566.15**	**71.76**	**13**	**-9.98**	**0.16**	**20.8**		**0.8711**	**0.9998**

Abbreviations: df degrees of freedom; **HD** Huntington's Disease; **MW-U** Mann Whitney U Rank; **PD** Percentage Difference (HD to Control); **SEM** Standard Error of Mean.

Supplementary Table 14 Concentrations of sulfatide species in the caudate. Data was assessed for normality using a D'Agostino Pearson Omnibus test. Data which was normally distributed was analysed using an unpaired t-test with Welch's correction, whilst data that did not fit normality assumptions was analysed using a Mann Whitney U test. P values are shown with corresponding significance (**p<0.01). Data is in pmol lipid per mg protein. The adjusted p values (q values) for multiple comparisons corrections using a two-stage linear step-up Benjamini, Krieger, and Yekutieli method have been included for the readers information.

	CON			HD								
**Caudate**	**Mean**	**SEM**	**n**	**Mean**	**SEM**	**n**	**PD (%)**	**t ratio**	**df**	**MW-U**	**p**	**q**
(3'-sulfo)Galβ-Cer(d18:1/16:0)	6.55	0.55	12	8.31	1.14	12	26.82			51.0	0.2828	0.9695
(3'-sulfo)Galβ-Cer(d18:1/18:0)	135.89	20.33	12	110.57	20.52	12	-18.63			53.0	0.2718	0.9747
(3'-sulfo)Galβ-Cer(d18:1/20:0)	28.33	4.52	12	21.02	3.69	12	-25.81			46.0	0.1554	0.8947
(3'-sulfo)Galβ-Cer(d18:1/22:0)	124.29	17.85	12	66.88	12.71	12	-46.19			28.0	0.0095**	0.2462
(3'-sulfo)Galβ-Cer(d18:1/23:0)	169.53	25.72	12	96.74	17.28	12	-42.94			31.5	0.0196	0.3725
(3'-sulfo)Galβ-Cer(d18:1/24:0)	586.80	85.52	12	308.23	66.54	12	-47.47			26.0	0.0068**	0.1909
(3'-sulfo)Galβ-Cer(d18:1/24:1)	622.30	105.42	12	292.04	51.02	12	-53.07			26.0	0.0057**	0.1909
(3'-sulfo)Galβ-Cer(d18:1/18:0(2OH))	17.45	2.50	12	17.28	1.56	12	-1.00			60.5	0.5587	0.9775
(3'-sulfo)Galβ-Cer(18:1/20:0(2OH))	55.96	6.90	12	53.67	5.58	12	-4.10	0.26	21.09		0.8029	0.9998
(3'-sulfo)Galβ-Cer(d18:1/22:0(2OH))	245.06	37.82	12	194.17	27.88	12	-20.77			54.0	0.3259	0.9747
(3'-sulfo)Galβ-Cer(d18:1/23:0(2OH))	498.59	84.96	12	338.28	48.74	12	-32.15			45.0	0.1240	0.8876
(3'-sulfo)Galβ-Cer(d18:1/24:0(2OH))	1158.54	171.19	12	759.43	109.73	12	-34.45			36.0	0.0387	0.5805
(3'-sulfo)Galβ-Cer(d18:1/24:1(2OH))	1131.80	204.93	12	891.60	115.62	12	-21.22			60.0	0.5137	0.9775
**Total Sulfatide**	**1673.69**	**256.19**	**12**	**834.26**	**169.26**	**12**	**-50.15**			**29.0**	**0.0121**	**0.2800**
**Total OH-Sulfatide**	**3107.41**	**504.90**	**12**	**2081.01**	**328.85**	**12**	**-33.03**	**1.45**	**18.04**		**0.1649**	**0.9468**
**Total Combined Sulfatide**	**4781.10**	**728.80**	**12**	**2915.27**	**468.93**	**12**	**-39.03**	**1.91**	**17.98**		**0.0721**	**0.8073**

Abbreviations: df degrees of freedom; **HD** Huntington's Disease; **MW-U** Mann Whitney U Rank; **PD** Percentage Difference (HD to Control); **SEM** Standard Error of Mean.

2.2 Putamen

Supplementary Table 15 Concentrations of ceramide species in the putamen. Data was assessed for normality using a D'Agostino Pearson Omnibus test. Data which was normally distributed was analysed using an unpaired t-test with Welch's correction, whilst data that did not fit normality assumptions was analysed using a Mann Whitney U test. P values are shown with corresponding significance (***p<0.001). Data is in pmol lipid per mg tissue. The adjusted p values (q values) for multiple comparisons corrections using a two-stage linear step-up Benjamini, Krieger, and Yekutieli method have been included for the readers information.

	CON			HD								
**Putamen**	**Mean**	**SEM**	**n**	**Mean**	**SEM**	**n**	**PD(%)**	**t ratio**	**df**	**MW-U**	**p**	**q**
Cer d18:1/16:0	8.16	0.37	11	10.46	0.99	13	28.14	4.26	15.27		0.0006***	0.0048
Cer d18:1/18:0	149.49	6.75	11	192.53	21.62	13	28.79	1.49	14.29		0.1584	0.3169
Cer d18:1/19:0	2.82	0.20	11	3.22	0.28	11	14.23			36.00	0.1164	0.4761
Cer d18:1/20:0	61.66	4.33	11	14.75	1.00	13	-76.08			70.00	0.9547	0.9795
Cer d18:1/21:0	2.93	0.38	9	3.56	0.41	11	21.27	0.43	17.96		0.6713	0.6404
Cer d18:1/22:0	7.24	0.57	11	7.53	0.86	13	3.96			59.00	0.494	0.7320
Cer d18:1/22:1	4.30	1.19	11	8.31	1.26	13	93.38			47.00	0.1674	0.4761
Cer d18:1/22:2	5.49	0.51	11	5.24	1.27	12	-4.48			45.00	0.1693	0.4761
Cer d18:1/23:0	4.98	0.66	10	7.22	0.86	13	45.05			30.50	0.0303	0.3617
Cer d18:1/24:0	10.44	0.85	11	6.82	0.87	13	-34.63	1.10	21.91		0.2871	0.4370
Cer d18:1/24:1	50.08	12.22	11	94.83	19.54	13	89.37			50.00	0.2284	0.4761
Cer d18:1/24:2	14.82	4.13	11	33.08	5.48	13	123.20			42.50	0.0933	0.4761
Cer d18:1/25:0	4.16	0.46	10	5.28	0.88	13	27.08			44.00	0.208	0.4761
**Total Ceramide**	**321.59**	**23.25**	**11**	**391.39**	**45.26**	**13**	**21.70**	**1.82**	**17.69**		**0.0864**	**0.1847**

Abbreviations: df degrees of freedom; **HD** Huntington's Disease; **MW-U** Mann Whitney U Rank; **PD** Percentage Difference (HD to Control); **SEM** Standard Error of Mean.

Supplementary Table 16 Concentrations of sphingomyelin species in the putamen. Data was assessed for normality using a D'Agostino Pearson Omnibus test. Data which was normally distributed was analysed using an unpaired t-test with Welch's correction, whilst data that did not fit normality assumptions was analysed using a Mann Whitney U test. P values are shown with corresponding significance (**p<0.01, ***p<0.001). Data is in pmol lipid per mg tissue. The adjusted p values (q values) for multiple comparisons corrections using a two-stage linear step-up Benjamini, Krieger, and Yekutieli method have been included for the readers information.

	CON			HD								
**Putamen**	**Mean**	**SEM**	**n**	**Mean**	**SEM**	**n**	**PD(%)**	**t ratio**	**df**	**MW-U**	**p**	**q**
SM d18:1/14:0	13.93	0.71	11	22.55	2.80	13	61.92	4.20	13.53		0.0008***	0.0058
SM d18:1/15:0	3.77	0.41	10	8.87	1.05	13	135.24	4.54	15.39		0.0005***	0.0033
SM d18:1/16:0	263.37	10.76	11	398.71	38.84	13	51.39	3.81	13.81		0.0019**	0.0102
SM d18:1/16:1	6.82	0.32	10	15.18	1.37	13	122.49	5.92	13.28		0.0000***	0.0010
SM d18:1/17:0	33.87	2.18	11	64.69	6.41	13	91.02	4.74	14.68		0.0002***	0.0033
SM d18:1/18:0	2329.13	90.53	11	2886.31	330.35	13	23.92	1.04	13.78		0.3445	0.4370
SM d18:1/18:1	176.51	11.45	11	403.03	28.25	13	128.34	5.44	15.76		0.0000***	0.0010
SM d18:1/19:0	37.77	2.09	11	44.48	3.03	13	17.78	2.03	20.56		0.0632	0.1347
SM d18:1/20:1	23.79	1.55	11	28.24	1.80	13	18.70	1.92	21.92		0.0743	0.1549
SM d18:1/21:0	12.09	0.86	10	12.51	0.83	13	3.49	0.34	20.28		0.7308	0.6874
SM d18:1/22:0	54.79	7.03	11	81.68	6.11	13	49.08			47.00	0.1674	0.4761
SM d18:1/22:1	26.69	3.15	11	58.52	8.19	13	119.24	3.39	15.42		0.0044**	0.0177
SM d18:1/23:0	52.81	7.74	11	70.08	11.23	13	32.70			54.00	0.3311	0.5733
SM d18:1/23:1	40.82	5.51	11	91.89	14.25	13	125.10	2.95	15.44		0.0115	0.0353
SM d18:1/24:0	100.75	21.45	11	134.47	23.40	13	33.48			69.00	0.9095	0.9726
SM d18:1/24:1	952.04	133.75	11	1455.92	274.56	13	52.93			53.00	0.3031	0.5733
SM d18:1/24:2	37.28	5.34	11	68.00	8.65	13	82.38	2.24	19.49		0.0432	0.1111
SM d18:1/25:0	30.87	6.56	11	58.45	9.55	13	89.32	1.23	20.52		0.2505	0.3862
SM d18:1/25:1	238.22	33.52	11	438.97	68.42	13	84.27	2.32	17.26		0.0393	0.1082
SM d18:1/26:0	12.25	2.15	9	14.08	2.12	13	15.00	0.61	19.07		0.5626	0.5895
SM d18:1/26:1	145.90	17.65	10	212.57	32.62	13	45.70	2.11	18.00		0.0672	0.1347
SM d18:1/26:2	13.75	1.90	10	24.57	2.85	13	78.61	3.16	19.82		0.0077**	0.0201
**Total SM**	**4558.54**	**296.17**	**11**	**6593.78**	**753.73**	**13**	**44.65**	**2.08**	**15.55**		**0.0641**	**0.1347**

Abbreviations: df degrees of freedom; **HD** Huntington's Disease; **MW-U** Mann Whitney U Rank; **PD** Percentage Difference (HD to Control); **SEM** Standard Error of Mean.

Supplementary Table 17 Concentrations of HexCer species in the putamen. Data was assessed for normality using a D'Agostino Pearson Omnibus test. Data which was normally distributed was analysed using an unpaired t-test with Welch's correction, whilst data that did not fit normality assumptions was analysed using a Mann Whitney U test. Data is in pmol lipid per mg protein. The adjusted p values (q values) for multiple comparisons corrections using a two-stage linear step-up Benjamini, Krieger, and Yekutieli method have been included for the readers information.

	CON			HD								
**Putamen**	**Mean**	**SEM**	**n**	**Mean**	**SEM**	**n**	**PD (%)**	**t ratio**	**df**	**MW-U**	**p**	**q**
HexCer d18:1/16:0	156.60	36.40	12	179.63	16.98	13	14.71			55.0	0.2305	0.4761
HexCer d18:1/18:0	4715.43	1289.18	12	3864.50	449.48	13	-18.05			70.0	0.6885	0.8543
HexCer d18:1/20:0	381.75	92.42	12	324.08	33.75	13	-15.11			70.0	0.6885	0.8543
HexCer d18:1/22:0	833.82	258.90	12	620.66	89.50	13	-25.56			71.0	0.7283	0.8543
HexCer d18:1/23:0	1239.92	363.71	12	1057.70	148.82	13	-14.70			70.0	0.6885	0.8543
HexCer d18:1/24:0	2773.73	924.73	12	1983.09	273.48	13	-28.50			71.0	0.7283	0.8543
HexCer d18:1/24:1	15392.13	5099.78	12	11049.42	1525.19	13	-28.21			73.0	0.8100	0.8925
HexCer d18:1/25:0	863.37	216.78	12	806.53	102.00	13	-6.58			65.0	0.4783	0.7320
HexCer d18:1/26:0	178.16	40.98	12	178.07	21.72	13	-0.05			59.5	0.3544	0.5733
HexCer d18:1/26:1	2440.47	666.04	12	2359.52	321.57	13	-3.32			62.0	0.4059	0.6417
**Total HexCer**	**28975.36**	**8894.69**	**12**	**22423.22**	**2904.70**	**13**	**-22.61**			**72.0**	**0.7689**	**0.8736**

Abbreviations: df degrees of freedom; **HD** Huntington's Disease; **MW-U** Mann Whitney U Rank; **PD** Percentage Difference (HD to Control); **SEM** Standard Error of Mean.

Supplementary Table 18 Concentrations of Hex2Cer species in the putamen. Data was assessed for normality using a D'Agostino Pearson Omnibus test. Data which was normally distributed was analysed using an unpaired t-test with Welch's correction, whilst data that did not fit normality assumptions was analysed using a Mann Whitney U test. P values are shown with corresponding significance. Data is in pmol lipid per mg protein. The adjusted p values (q values) for multiple comparisons corrections using a two-stage linear step-up Benjamini, Krieger, and Yekutieli method have been included for the readers information.

	CON			HD								
**Putamen**	**Mean**	**SEM**	**n**	**Mean**	**SEM**	**n**	**PD (%)**	**t ratio**	**df**	**MW-U**	**p**	**q**
Hex2Cer d18:1/16:0	32.33	4.08	12	49.70	4.99	13	53.72			35.0	0.0128	0.3617
Hex2Cer d18:1/18:0	274.45	47.15	12	359.80	36.18	13	31.10	1.44	21.07		0.3659	0.3169
Hex2Cer d18:1/22:0	40.99	6.52	12	50.95	4.69	13	24.31	1.24	20.33		0.8519	0.3862
Hex2Cer d18:1/24:0	114.16	21.99	12	125.57	13.83	13	10.00	0.44	18.73		0.6051	0.6404
Hex2Cer d18:1/24:1	574.74	112.21	12	623.17	55.12	13	8.43			54.0	0.2051	0.4761
Hex2Cer d18:1/25:0	57.06	10.68	12	65.29	7.34	13	14.43	0.63	19.80		0.7797	0.5873
Hex2Cer d18:1/26:0	12.87	1.99	12	15.12	1.46	13	17.52	0.91	20.60		0.8680	0.4370
Hex2Cer d18:1/26:1	161.81	29.22	12	213.95	23.25	13	32.23	1.40	21.46		0.6117	0.3216
**Total Hex2Cer**	**1268.40**	**228.59**	**12**	**1503.56**	**131.56**	**13**	**18.54**	**0.89**	**17.71**		**0.3848**	**0.4370**

Abbreviations: df degrees of freedom; **HD** Huntington's Disease; **MW-U** Mann Whitney U Rank; **PD** Percentage Difference (HD to Control); **SEM** Standard Error of Mean.

Supplementary Table 19 Concentrations of sulfatide species in the putamen. Data was assessed for normality using a D'Agostino Pearson Omnibus test. Data which was normally distributed was analysed using an unpaired t-test with Welch's correction, whilst data that did not fit normality assumptions was analysed using a Mann Whitney U test. P values are shown with corresponding significance. Data is in pmol lipid per mg protein. The adjusted p values (q values) for multiple comparisons corrections using a two-stage linear step-up Benjamini, Krieger, and Yekutieli method have been included for the readers information.

	CON			HD								
**Putamen**	**Mean**	**SEM**	**n**	**Mean**	**SEM**	**n**	**PD (%)**	**t ratio**	**df**	**MW-U**	**p**	**q**
(3'-sulfo)Galβ-Cer(d18:1/16:0)	10.44	1.49	12	10.91	0.78	13	4.52	0.27	16.77		0.8354	0.7166
(3'-sulfo)Galβ-Cer(d18:1/18:0)	370.18	95.10	12	324.71	43.09	13	-12.28	0.44	15.38		0.2976	0.6404
(3'-sulfo)Galβ-Cer(d18:1/20:0)	91.41	26.12	12	64.98	8.47	13	-28.92	0.96	13.30		0.1749	0.4370
(3'-sulfo)Galβ-Cer(d18:1/22:0)	291.72	68.85	12	222.13	27.85	13	-23.85	0.94	14.54		0.1724	0.4370
(3'-sulfo)Galβ-Cer(d18:1/23:0)	465.13	127.91	12	340.53	48.40	13	-26.79	0.91	14.11		0.1796	0.4370
(3'-sulfo)Galβ-Cer(d18:1/24:0)	1440.74	396.38	12	1066.60	151.24	13	-25.97			77.0	0.9787	0.9885
(3'-sulfo)Galβ-Cer(d18:1/24:1)	1651.78	413.80	12	1199.34	150.02	13	-27.39	1.03	13.86		0.1532	0.4370
(3'-sulfo)Galβ-Cer(d18:1/18:0(2OH))	42.47	10.82	12	52.05	5.64	13	22.58			47.0	0.0948	0.4761
(3'-sulfo)Galβ-Cer(18:1/20:0(2OH))	93.52	10.64	12	119.12	7.15	13	27.37			40.0	0.0332	0.3617
(3'-sulfo)Galβ-Cer(d18:1/22:0(2OH))	577.87	116.38	12	687.27	59.54	13	18.93			51.0	0.1519	0.4761
(3'-sulfo)Galβ-Cer(d18:1/23:0(2OH))	1440.13	371.85	12	1435.94	170.06	13	-0.29			61.0	0.3760	0.6215
(3'-sulfo)Galβ-Cer(d18:1/24:0(2OH))	2548.04	441.65	12	3065.92	278.29	13	20.32	0.99	18.76		0.6684	0.4370
(3'-sulfo)Galβ-Cer(d18:1/24:1(2OH))	3101.88	766.63	12	3653.81	317.16	13	17.79			45.0	0.0768	0.4761
**Total Sulfatide**	**4321.39**	**1113.10**	**12**	**3229.20**	**416.85**	**13**	**-25.27**	**0.92**	**14.05**		**0.3737**	**0.4370**
**Total OH-Sulfatide**	**7803.91**	**1698.79**	**12**	**9014.11**	**812.69**	**13**	**15.51**			**52.0**	**0.1683**	**0.4761**
**Total Combined Sulfatide**	**12125.29**	**2600.98**	**12**	**12243.31**	**1181.16**	**13**	**0.97**	**0.04**	**15.40**		**0.9676**	**0.8376**

Abbreviations: df degrees of freedom; **HD** Huntington's Disease; **MW-U** Mann Whitney U Rank; **PD** Percentage Difference (HD to Control); **SEM** Standard Error of Mean.

2.3 Cerebellum

Supplementary Table 20 Concentrations of ceramide species in the cerebellum. Data was assessed for normality using a D'Agostino Pearson Omnibus test. Data which was normally distributed was analysed using an unpaired t-test with Welch's correction, whilst data that did not fit normality assumptions was analysed using a Mann Whitney U test. P values are shown with corresponding significance (***p<0.001). Data is in pmol lipid per mg protein. The adjusted p values (q values) for multiple comparisons corrections using a two-stage linear step-up Benjamini, Krieger, and Yekutieli method have been included for the readers information.

	CON			HD								
**Cerebellum**	**Mean**	**SEM**	**n**	**Mean**	**SEM**	**n**	**PD(%)**	**t ratio**	**df**	**MW-U**	**p**	**q**
Cer d18:1/16:0	5.81	0.73	13	5.23	0.49	9	-9.86			55.00	0.8446	0.8824
Cer d18:1/18:0	80.41	6.56	13	97.20	5.07	9	20.88	2.03	19.94		0.0563	0.2453
Cer d18:1/19:0	1.69	0.18	7	1.75	0.19	6	3.96			17.00	0.6282	0.7440
Cer d18:1/20:0	9.50	0.70	13	10.94	0.65	9	15.19	1.52	19.70		0.1452	0.3103
Cer d18:1/21:0	2.78	0.21	13	2.22	0.32	9	-20.10	1.45	14.86		0.1645	0.3174
Cer d18:1/22:0	3.81	0.56	13	3.73	0.53	9	-1.97	0.10	19.63		0.9236	0.9304
Cer d18:1/22:1	2.15	0.28	11	2.39	0.22	9	11.15	0.65	17.76		0.5105	0.5674
Cer d18:1/22:2	2.34	0.45	8	1.66	0.16	7	-29.16			18.50	0.281	0.6075
Cer d18:1/23:0	2.22	0.74	5	1.37	0.19	5	-38.29			8.00	0.4206	0.6075
Cer d18:1/24:0	4.70	1.11	13	2.50	0.25	9	-46.65			30.50	0.0708	0.2857
Cer d18:1/24:1	18.79	2.74	13	21.60	3.08	9	14.94			41.00	0.2624	0.6075
Cer d18:1/24:2	6.12	0.96	13	7.28	0.97	9	18.83	0.83	19.08		0.4085	0.5036
Cer d18:1/25:0	2.35	0.54	7	1.39	0.11	8	-40.99			8.50	0.0002***	0.1328
**Total Ceramide**	**138.20**	**11.05**	**13**	**157.56**	**8.40**	**9**	**14.00**	**1.39**	**19.91**		**0.1785**	0.3174

Abbreviations: df degrees of freedom; **HD** Huntington's Disease; **MW-U** Mann Whitney U Rank; **PD** Percentage Difference (HD to Control); **SEM** Standard Error of Mean.

Supplementary Table 21 Concentrations of sphingomyelin species in the cerebellum. Data was assessed for normality using a D'Agostino Pearson Omnibus test. Data which was normally distributed was analysed using an unpaired t-test with Welch's correction, whilst data that did not fit normality assumptions was analysed using a Mann Whitney U test. P values are shown with corresponding significance (*p<0.05). Data is in pmol lipid per mg tissue. The adjusted p values (q values) for multiple comparisons corrections using a two-stage linear step-up Benjamini, Krieger, and Yekutieli method have been included for the readers information.

	**CON**			**HD**								
**Cerebellum**	**Mean**	**SEM**	**n**	**Mean**	**SEM**	**n**	**PD(%)**	**t ratio**	**df**	**MW-U**	**p**	**q**
SM d18:1/14:0	15.32	1.29	13	12.63	0.88	9	-17.59	1.72	19.45		0.1334	0.2519
SM d18:1/15:0	3.78	0.55	10	3.55	0.47	8	-6.34	0.34	15.99		0.7527	0.7849
SM d18:1/16:0	214.26	19.06	13	199.02	17.01	9	-7.11			53.00	0.7438	0.8347
SM d18:1/16:1	7.25	1.81	13	5.27	0.51	9	-27.29			52.50	0.6948	0.8241
SM d18:1/17:0	17.97	1.45	13	21.90	1.92	9	21.86	1.63	16.23		0.1120	0.2845
SM d18:1/18:0	2030.61	184.45	13	2249.65	172.79	9	10.79	0.87	19.63		0.4179	0.5035
SM d18:1/18:1	114.92	13.24	13	142.82	12.86	9	24.28	1.51	19.41		0.1619	0.3103
SM d18:1/19:0	16.36	1.40	13	17.65	1.33	9	7.90	0.67	19.54		0.5293	0.5674
SM d18:1/20:1	10.54	0.91	13	12.58	0.74	9	19.33	1.73	20.00		0.3632	0.2519
SM d18:1/21:0	3.72	0.41	10	3.30	0.29	7	-11.30			29.00	0.5837	0.7440
SM d18:1/22:0	40.75	4.79	13	38.01	3.26	9	-6.71			55.00	0.8446	0.8824
SM d18:1/22:1	14.37	1.73	13	17.77	1.60	9	23.68	1.44	19.73		0.6731	0.3174
SM d18:1/23:0	19.47	2.22	13	24.26	3.70	9	24.59	1.11	13.63		0.1848	0.3961
SM d18:1/23:1	13.41	1.46	12	19.83	2.37	9	47.83	2.30	13.78		0.2517	0.2453
SM d18:1/24:0	60.82	7.81	13	62.44	8.07	9	2.67	0.15	18.94		0.0257	0.9145
SM d18:1/24:1	331.26	47.18	13	425.01	58.37	9	28.30	1.25	17.02		0.8896	0.3547
SM d18:1/24:2	18.71	2.46	13	22.87	3.07	9	22.23			42.50	0.3237	0.6075
SM d18:1/25:0	18.17	2.84	12	21.66	3.50	9	19.17	0.77	16.73		0.2996	0.5294
SM d18:1/25:1	67.96	11.26	13	103.06	13.95	9	51.65	1.96	17.01		0.4449	0.2471
SM d18:1/26:0	5.45	0.85	9	6.68	1.19	8	22.51	0.83	12.87		0.0630	0.5036
SM d18:1/26:1	46.73	7.87	13	64.14	9.46	9	37.27	1.41	17.36		0.4075	0.3174
SM d18:1/26:2	5.47	0.56	8	5.95	0.96	7	8.83			28.00	0.9999	>0.9999
**Total SM**	**3069.35**	**265.29**	**13**	**3476.85**	**285.11**	**9**	**13.28**	**1.05**	**18.57**		**0.7481**	0.4159

Abbreviations: df degrees of freedom; **HD** Huntington's Disease; **MW-U** Mann Whitney U Rank; **PD** Percentage Difference (HD to Control); **SEM** Standard Error of Mean.

Supplementary Table 22 Concentrations of HexCer species in the cerebellum. Data was assessed for normality using a D'Agostino Pearson Omnibus test. Data which was normally distributed was analysed using an unpaired t-test with Welch's correction, whilst data that did not fit normality assumptions was analysed using a Mann Whitney U test. Data is in pmol lipid per mg protein. The adjusted p values (q values) for multiple comparisons corrections using a two-stage linear step-up Benjamini, Krieger, and Yekutieli method have been included for the readers information.

	**CON**			**HD**								
**Cerebellum**	**Mean**	**SEM**	**n**	**Mean**	**SEM**	n	**PD (%)**	**t ratio**	**df**	**MW-U**	**p**	**q**
HexCer d18:1/16:0	82.45	11.83	13	125.11	19.74	13	51.74	1.85	19.63		0.0761	0.2503
HexCer d18:1/18:0	2302.47	374.13	13	2748.51	486.05	13	19.37	0.73	22.53		0.4743	0.5408
HexCer d18:1/20:0	180.85	22.13	13	230.10	36.79	13	27.24	1.15	19.68		0.2628	0.3800
HexCer d18:1/22:0	315.40	46.63	13	416.17	72.23	13	31.95	1.17	20.52		0.2533	0.3772
HexCer d18:1/23:0	501.47	76.96	13	674.88	115.50	13	34.58			66.0	0.3551	0.6075
HexCer d18:1/24:0	1295.41	189.44	13	1582.66	271.92	13	22.17	0.87	21.43		0.3947	0.5035
HexCer d18:1/24:1	6313.88	1131.22	13	8150.00	1540.56	13	29.08			71.0	0.5114	0.7044
HexCer d18:1/25:0	534.27	78.17	13	631.93	98.17	13	18.28			71.0	0.5200	0.7044
HexCer d18:1/26:0	129.02	19.35	13	144.79	21.14	13	12.22			74.0	0.6139	0.7440
HexCer d18:1/26:1	1229.97	218.75	13	1539.47	235.16	13	25.16			61.0	0.2428	0.6075
**Total HexCer**	**12885.20**	**2144.62**	**13**	**16243.63**	**2863.08**	**13**	**26.06**			**70.0**	**0.4793**	**0.7044**

Abbreviations: df degrees of freedom; **HD** Huntington's Disease; **MW-U** Mann Whitney U Rank; **PD** Percentage Difference (HD to Control); **SEM** Standard Error of Mean.

Supplementary Table 23 Concentrations of Hex2Cer species in the cerebellum. Data was assessed for normality using a D'Agostino Pearson Omnibus test. Data which was normally distributed was analysed using an unpaired t-test with Welch's correction, whilst data that did not fit normality assumptions was analysed using a Mann Whitney U test. P values are shown with corresponding significance (**p<0.01). Data is in pmol lipid per mg protein. The adjusted p values (q values) for multiple comparisons corrections using a two-stage linear step-up Benjamini, Krieger, and Yekutieli method have been included for the readers information.

	CON			HD								
**Cerebellum**	**Mean**	**SEM**	**n**	**Mean**	**SEM**	n	**PD (%)**	**t ratio**	**df**	**MW-U**	**p**	**q**
Hex2Cer d18:1/16:0	36.62	3.26	13	89.65	34.95	13	144.85			27.0	0.0020**	0.0029
Hex2Cer d18:1/18:0	242.77	20.19	13	330.94	33.71	13	36.32	2.24	19.63		0.0343	0.2453
Hex2Cer d18:1/22:0	15.26	1.28	13	27.13	4.24	13	77.75			32.0	0.0057**	0.0878
Hex2Cer d18:1/24:0	33.19	3.19	13	58.65	9.21	13	76.74	2.61	14.82		0.0155	0.2453
Hex2Cer d18:1/24:1	136.95	15.17	13	253.11	35.24	13	84.82	3.03	16.30		0.0058**	0.1751
Hex2Cer d18:1/25:0	14.93	1.61	13	25.33	3.92	13	69.69			48.5	0.0655	0.2857
Hex2Cer d18:1/26:0	4.42	0.49	13	7.39	0.90	13	67.36			40.0	0.0169	0.1328
Hex2Cer d18:1/26:1	37.97	4.92	13	68.97	9.85	13	81.62			39.5	0.0212	0.1328
**Total Hex2Cer**	**522.10**	**45.32**	**13**	**861.18**	**94.15**	**13**	**64.94**	**3.25**	**17.28**		**0.0090****	**0.1751**

Abbreviations: df degrees of freedom; **HD** Huntington's Disease; **MW-U** Mann Whitney U Rank; **PD** Percentage Difference (HD to Control); **SEM** Standard Error of Mean.

Supplementary Table 24 Concentrations of sulfatide species in the cerebellum. Data was assessed for normality using a D'Agostino Pearson Omnibus test. Data which was normally distributed was analysed using an unpaired t-test with Welch's correction, whilst data that did not fit normality assumptions was analysed using a Mann Whitney U test. P values are shown with corresponding significance. Data is in pmol lipid per mg protein. The adjusted p values (q values) for multiple comparisons corrections using a two-stage linear step-up Benjamini, Krieger, and Yekutieli method have been included for the readers information.

	CON			HD								
**Cerebellum**	**Mean**	**SEM**	**n**	**Mean**	**SEM**	n	**PD (%)**	**t ratio**	**df**	**MW-U**	**p**	**q**
(3'-sulfo)Galβ-Cer(d18:1/16:0)	7.68	0.70	13	9.98	1.02	13	30.03	1.87	21.30		0.0930	0.2503
(3'-sulfo)Galβ-Cer(d18:1/18:0)	192.34	30.07	13	266.12	51.46	13	38.36	1.24	19.34		0.2276	0.3547
(3'-sulfo)Galβ-Cer(d18:1/20:0)	30.85	4.06	13	51.42	10.87	13	66.70	1.77	15.27		0.0906	0.2519
(3'-sulfo)Galβ-Cer(d18:1/22:0)	125.53	19.43	13	171.71	31.96	13	36.79	1.23	19.80		0.2301	0.3547
(3'-sulfo)Galβ-Cer(d18:1/23:0)	177.62	30.07	13	252.18	47.02	13	41.97	1.34	20.41		0.1936	0.3355
(3'-sulfo)Galβ-Cer(d18:1/24:0)	598.93	94.23	13	863.47	172.87	13	44.17			66.0	0.3622	0.6075
(3'-sulfo)Galβ-Cer(d18:1/24:1)	637.28	113.10	13	944.93	182.49	13	48.27			62.0	0.2642	0.6075
(3'-sulfo)Galβ-Cer(d18:1/18:0(2OH))	21.46	2.36	13	28.78	4.17	13	34.08			63.5	0.2808	0.6075
(3'-sulfo)Galβ-Cer(18:1/20:0(2OH))	63.42	5.92	13	76.02	9.93	13	19.86			64.0	0.3043	0.6075
(3'-sulfo)Galβ-Cer(d18:1/22:0(2OH))	216.90	25.30	13	351.74	55.02	13	62.17	2.23	16.86		0.0354	0.2453
(3'-sulfo)Galβ-Cer(d18:1/23:0(2OH))	384.48	47.44	13	653.92	111.07	13	70.08	2.23	16.24		0.0353	0.2453
(3'-sulfo)Galβ-Cer(d18:1/24:0(2OH))	1034.28	126.84	13	1660.61	284.25	13	60.56	2.01	16.60		0.0555	0.2453
(3'-sulfo)Galβ-Cer(d18:1/24:1(2OH))	1013.50	147.87	13	1640.25	272.94	13	61.84	2.02	18.49		0.0548	0.2453
**Total Sulfatide**	**1770.22**	**289.79**	**13**	**2559.81**	**493.56**	**13**	**44.60**			**64.0**	**0.3107**	**0.6075**
**Total OH-Sulfatide**	**2734.04**	**352.19**	**13**	**4411.31**	**733.84**	**13**	**61.35**	**2.06**	**17.25**		**0.0548**	**0.2453**
**Total Combined Sulfatide**	**4504.26**	**623.66**	**13**	**6971.13**	**1213.74**	**13**	**54.77**	**1.81**	**17.92**		**0.0874**	**0.2519**

Abbreviations: df degrees of freedom; **HD** Huntington's Disease; **MW-U** Mann Whitney U Rank; **PD** Percentage Difference (HD to Control); **SEM** Standard Error of Mean.

2.4 White Cortex

Supplementary Table 25 Concentrations of ceramide species in the white matter of the dorsomedial prefrontal cortex. Data was assessed for normality using a D'Agostino Pearson Omnibus test. Data which was normally distributed was analysed using an unpaired t-test with Welch's correction, whilst data that did not fit normality assumptions was analysed using a Mann Whitney U test. P values are shown with corresponding significance. Data is in pmol lipid per mg tissue. The adjusted p values (q values) for multiple comparisons corrections using a two-stage linear step-up Benjamini, Krieger, and Yekutieli method have been included for the readers information.

	CON			HD								
**White Cortex**	**Mean**	**SEM**	**n**	**Mean**	**SEM**	**n**	**PD(%)**	**t ratio**	**df**	**MW-U**	**p**	**q**
Cer d18:1/18:0	199.24	11.99	13	155.97	16.13	13	-21.71	2.15	22.16		0.0416	0.4137
Cer d18:1/22:0	22.38	1.99	10	18.18	2.08	13	-18.79	1.46	20.79		0.1685	0.5760
Cer d18:1/22:1	16.35	1.23	8	13.78	1.93	9	-15.72	1.12	13.36		0.2921	0.6387
Cer d18:1/23:0	21.19	1.59	13	19.74	2.46	13	-6.84	0.49	20.50		0.6245	0.8815
Cer d18:1/24:0	24.26	2.12	13	24.38	3.34	13	0.51	0.03	20.31		0.9756	0.9868
Cer d18:1/24:1	446.58	33.00	13	324.10	46.74	13	-27.43	2.14	21.58		0.0427	0.4137
Cer d18:1/24:2	48.67	2.40	13	40.77	4.47	13	-16.23	1.56	18.38		0.1327	0.5760
Cer d18:1/25:1	51.52	4.08	13	42.04	5.45	13	-18.40	1.39	22.24		0.1765	0.5760
Cer d18:1/26:1	19.05	1.38	10	15.17	1.84	13	-20.34	1.68	20.60		0.1249	0.5488
**Total Ceramide**	**833.38**	**56.60**	**13**	**649.90**	**81.35**	**13**	**-22.02**	**1.85**	**21.41**		**0.0765**	**0.5330**

Abbreviations: df degrees of freedom; **HD** Huntington's Disease; **MW-U** Mann Whitney U Rank; **PD** Percentage Difference (HD to Control); **SEM** Standard Error of Mean.

Supplementary Table 26 Concentrations of sphingomyelin species in the white matter of the dorsomedial prefrontal cortex. Data was assessed for normality using a D'Agostino Pearson Omnibus test. Data which was normally distributed was analysed using an unpaired t-test with Welch's correction, whilst data that did not fit normality assumptions was analysed using a Mann Whitney U test. P values are shown with corresponding significance. Data is in pmol lipid per mg tissue. The adjusted p values (q values) for multiple comparisons corrections using a two-stage linear step-up Benjamini, Krieger, and Yekutieli method have been included for the readers information.

	CON			HD								
**White Cortex**	**Mean**	**SEM**	**n**	**Mean**	**SEM**	**n**	**PD(%)**	**t ratio**	**df**	**MW-U**	**p**	**q**
SM d18:1/16:0	479.31	30.22	13	505.05	27.65	13	5.37	0.63	23.81		0.5356	0.8654
SM d18:1/18:0	3613.76	205.41	13	3759.29	227.14	13	4.03	0.48	23.76		0.6390	0.8815
SM d18:1/18:1	417.19	26.90	13	422.98	29.22	13	1.39	0.15	23.84		0.8853	0.9448
SM d18:1/20:0	355.65	20.97	13	428.14	50.93	13	20.38			67.00	0.3897	0.5510
SM d18:1/22:0	292.49	18.04	13	317.78	20.79	13	8.65	0.92	23.53		0.3673	0.6760
SM d18:1/22:1	228.44	10.92	13	213.43	19.05	13	-6.57			80.00	0.8403	0.8487
SM d18:1/23:0	363.99	20.98	13	373.76	26.75	13	2.69	0.29	22.71		0.7762	0.8957
SM d18:1/23:1	361.11	17.50	13	332.53	29.16	13	-7.91			77.00	0.7241	0.8487
SM d18:1/24:0	1124.59	77.23	13	1082.90	95.76	13	-3.71	0.34	22.97		0.7376	0.8850
SM d18:1/24:1	9556.72	484.82	13	7607.06	725.95	13	-20.40	2.23	20.93		0.0351	0.4137
SM d18:1/24:2	273.31	15.53	13	249.28	23.99	13	-8.79	0.84	20.56		0.4087	0.7251
SM d18:1/25:0	342.28	25.41	13	327.20	23.67	13	-4.41	0.43	23.88		0.6680	0.8850
SM d18:1/25:1	1592.11	67.87	13	1355.02	105.42	13	-14.89	1.89	20.49		0.0708	0.5330
SM d18:1/26:0	85.78	5.00	8	77.02	7.49	10	-10.20	0.97	14.98		0.3717	0.6760
SM d18:1/26:1	1006.68	46.59	13	910.94	63.92	13	-9.51	1.21	21.94		0.2379	0.5879
**Total Sphingomyelin**	**20060.39**	**920.83**	**13**	**17944.59**	**1280.17**	**13**	**-10.55**	**1.34**	**21.80**		**0.1950**	**0.5760**

Abbreviations: df degrees of freedom; **HD** Huntington's Disease; **MW-U** Mann Whitney U Rank; **PD** Percentage Difference (HD to Control); **SEM** Standard Error of Mean.

Supplementary Table 27 Concentrations of HexCer species in the white matter of the dorsomedial prefrontal cortex. Data was assessed for normality using a D'Agostino Pearson Omnibus test. Data which was normally distributed was analysed using an unpaired t-test with Welch's correction, whilst data that did not fit normality assumptions was analysed using a Mann Whitney U test. P values are shown with corresponding significance. Data is in pmol lipid per mg protein. The adjusted p values (q values) for multiple comparisons corrections using a two-stage linear step-up Benjamini, Krieger, and Yekutieli method have been included for the readers information.

	CON			HD								
**White Cortex**	**Mean**	**SEM**	**n**	**Mean**	**SEM**	n	**PD (%)**	**t ratio**	**df**	**MW-U**	**p**	**q**
HexCer d18:1/16:0	891.50	73.05	13	789.82	73.24	13	-11.41	0.98	24.00		0.3360	0.6760
HexCer d18:1/18:0	28981.23	2563.46	13	22738.77	2502.73	13	-21.54	1.74	23.99		0.0942	0.5330
HexCer d18:1/20:0	2367.57	209.58	13	2073.46	183.28	13	-12.42	1.06	23.58		0.3017	0.6559
HexCer d18:1/22:0	6779.04	517.58	13	6163.65	431.57	13	-9.08	0.91	23.25		0.3703	0.6760
HexCer d18:1/23:0	11553.46	956.64	13	10136.08	749.66	13	-12.27	1.17	22.70		0.2550	0.6025
HexCer d18:1/24:0	30910.85	2943.13	13	27283.08	2492.27	13	-11.74	0.94	23.37		0.3563	0.6760
HexCer d18:1/24:1	108904.62	7199.75	13	84561.54	6559.08	13	-22.35	2.50	23.79		0.0197	0.4137
HexCer d18:1/25:0	9178.58	1020.22	13	8221.58	824.49	13	-10.43	0.73	22.99		0.4728	0.8107
HexCer d18:1/26:0	1541.46	186.57	13	1516.10	145.27	13	-1.65	0.11	22.64		0.9154	0.9448
HexCer d18:1/26:1	22359.38	1866.69	13	19965.00	1595.59	13	-10.71			60.0	0.2226	0.3934
**Total HexCer**	**223467.68**	**16074.57**	**13**	**183449.07**	**14524.15**	**13**	**-17.91**			**47.0**	**0.0568**	**0.2734**

Abbreviations: df degrees of freedom; **HD** Huntington's Disease; **MW-U** Mann Whitney U Rank; **PD** Percentage Difference (HD to Control); **SEM** Standard Error of Mean.

Supplementary Table 28 Concentrations of Hex2Cer species in the white matter of the dorsomedial prefrontal cortex. Data was assessed for normality using a D'Agostino Pearson Omnibus test. Data which was normally distributed was analysed using an unpaired t-test with Welch's correction, whilst data that did not fit normality assumptions was analysed using a Mann Whitney U test. P values are shown with corresponding significance. Data is in pmol lipid per mg protein. The adjusted p values (q values) for multiple comparisons corrections using a two-stage linear step-up Benjamini, Krieger, and Yekutieli method have been included for the readers information.

	**CON**			**HD**								
**White Cortex**	**Mean**	**SEM**	**n**	**Mean**	**SEM**	n	**PD (%)**	**t ratio**	**df**	**MW-U**	**p**	**q**
Hex2Cer d18:1/16:0	84.12	5.98	13	87.68	4.40	13	4.24	0.48	22.07		0.6292	0.8815
Hex2Cer d18:1/18:0	923.83	51.31	13	816.79	53.92	13	-11.59	1.44	23.94		0.1628	0.5760
Hex2Cer d18:1/22:0	149.30	8.14	13	174.81	8.29	13	17.08	2.20	23.99		0.0387	0.4137
Hex2Cer d18:1/24:0	618.62	42.80	13	694.89	42.26	13	12.33	1.27	24.00		0.2175	0.5879
Hex2Cer d18:1/24:1	1875.27	96.67	13	1856.69	132.74	13	-0.99	0.11	21.93		0.9103	0.9448
Hex2Cer d18:1/25:0	271.18	18.55	13	326.80	18.42	13	20.51	2.13	24.00		0.0435	0.4137
Hex2Cer d18:1/26:0	68.26	7.02	13	81.46	6.25	13	19.33	1.40	23.68		0.1727	0.5760
Hex2Cer d18:1/26:1	578.45	31.63	13	641.13	34.09	13	10.84	1.35	23.87		0.1893	0.5760
**Total Hex2Cer**	**4569.03**	**223.57**	**13**	**4680.26**	**264.78**	**13**	**2.43**	**0.32**	**23.34**		**0.7516**	**0.8850**

Abbreviations: df degrees of freedom; **HD** Huntington's Disease; **MW-U** Mann Whitney U Rank; **PD** Percentage Difference (HD to Control); **SEM** Standard Error of Mean.

Supplementary Table 29 Concentrations of sulfatide species in the white matter of the dorsomedial prefrontal cortex. Data was assessed for normality using a D'Agostino Pearson Omnibus test. Data which was normally distributed was analysed using an unpaired t-test with Welch's correction, whilst data that did not fit normality assumptions was analysed using a Mann Whitney U test. Data is in pmol lipid per mg protein. The adjusted p values (q values) for multiple comparisons corrections using a two-stage linear step-up Benjamini, Krieger, and Yekutieli method have been included for the readers information.

	**CON**			**HD**								
**White Cortex**	**Mean**	**SEM**	**n**	**Mean**	**SEM**	n	**PD (%)**	**t ratio**	**df**	**MW-U**	**p**	**q**
(3'-sulfo)Galβ-Cer(d18:1/16:0)	31.91	2.58	13	30.70	2.47	13	-3.79	0.34	23.95		0.7333	0.8850
(3'-sulfo)Galβ-Cer(d18:1/18:0)	1908.87	202.04	13	2647.47	556.30	13	38.69	1.25	15.11		0.2242	0.5879
(3'-sulfo)Galβ-Cer(d18:1/20:0)	731.32	98.83	13	585.41	63.88	13	-19.95	1.24	20.54		0.2267	0.5879
(3'-sulfo)Galβ-Cer(d18:1/22:0)	2023.12	170.22	13	1991.46	142.09	13	-1.56	0.14	23.26		0.8875	0.9448
(3'-sulfo)Galβ-Cer(d18:1/23:0)	3579.60	344.10	13	3428.92	265.10	13	-4.21	0.35	22.53		0.7318	0.8850
(3'-sulfo)Galβ-Cer(d18:1/24:0)	11307.62	1108.88	13	11450.62	832.41	13	1.26	0.10	22.26		0.9188	0.9448
(3'-sulfo)Galβ-Cer(d18:1/24:1)	9793.23	803.03	13	7981.50	630.84	13	-18.50	1.77	22.73		0.0887	0.5330
(3'-sulfo)Galβ-Cer(d18:1/18:0(2OH))	88.07	7.83	13	90.81	10.43	13	3.11	0.21	22.27		0.8380	0.9448
(3'-sulfo)Galβ-Cer(18:1/20:0(2OH))	126.17	6.29	13	134.85	7.47	13	6.88			53.5	0.4483	0.2734
(3'-sulfo)Galβ-Cer(d18:1/22:0(2OH))	1707.78	117.07	13	1652.13	87.78	13	-3.26	0.38	22.25		0.7068	0.8850
(3'-sulfo)Galβ-Cer(d18:1/23:0(2OH))	5002.58	485.30	13	4659.77	393.91	13	-6.85	0.55	23.03		0.5884	0.8815
(3'-sulfo)Galβ-Cer(d18:1/24:0(2OH))	7228.15	452.68	13	7513.96	338.71	13	3.95	0.51	22.23		0.6178	0.8815
(3'-sulfo)Galβ-Cer(d18:1/24:1(2OH))	7350.19	484.46	13	6230.12	488.08	13	-15.24	1.63	24.00		0.1164	0.5488
**Total Sulfatide**	**29375.66**	**2636.52**	**13**	**28116.08**	**2298.64**	**13**	**-4.29**	**0.36**	**23.56**		**0.7219**	**0.8850**
**Total OH-Sulfatide**	**21502.93**	**1467.28**	**13**	**20281.64**	**1225.73**	**13**	**-5.68**	**0.64**	**23.26**		**0.5292**	**0.8654**
**Total Combined Sulfatide**	**50878.59**	**3990.42**	**13**	**48397.71**	**3084.49**	**13**	**-4.88**	**0.49**	**22.57**		**0.6275**	**0.8815**

Abbreviations: df degrees of freedom; **HD** Huntington's Disease; **MW-U** Mann Whitney U Rank; **PD** Percentage Difference (HD to Control); **SEM** Standard Error of Mean.

2.5 Grey Cortex

Supplementary Table 30 Concentrations of ceramide species in the grey matter of the dorsomedial prefrontal cortex. Data was assessed for normality using a D'Agostino Pearson Omnibus test. Data which was normally distributed was analysed using an unpaired t-test with Welch's correction, whilst data that did not fit normality assumptions was analysed using a Mann Whitney U test. Data is in pmol lipid per mg tissue. The adjusted p values (q values) for multiple comparisons corrections using a two-stage linear step-up Benjamini, Krieger, and Yekutieli method have been included for the readers information.

	CON			HD								
**Grey Cortex**	**Mean**	**SEM**	**n**	**Mean**	**SEM**	**n**	**PD(%)**	**t ratio**	**df**	**MW-U**	**p**	**q**
Cer d18:1/16:0	6.26	1.24	3	4.97	0.32	11	-20.60			8.00	0.2253	0.7426
Cer d18:1/18:0	102.49	5.56	13	105.87	7.17	12	3.30	0.37	21.19		0.7128	0.7368
Cer d18:1/20:0	7.89	0.44	13	8.49	0.45	12	7.57	0.92	22.92		0.3552	0.4161
Cer d18:1/24:1	61.37	16.19	13	34.39	9.92	12	-43.96			48.50	0.1095	0.7426
Cer d18:1/24:2	16.56	3.95	12	11.66	2.95	7	-29.55			32.00	0.4320	0.7633
Cer d18:1/25:1	12.82	2.90	9	11.22	1.78	5	-12.50			21.00	0.6787	0.9071
**Total Ceramide**	**197.35**	**26.63**	**13**	**164.79**	**16.49**	**12**	**-16.50**			**68.00**	**0.6114**	0.7940

Abbreviations: df degrees of freedom; **HD** Huntington's Disease; **MW-U** Mann Whitney U Rank; **PD** Percentage Difference (HD to Control); **SEM** Standard Error of Mean.

Supplementary Table 31 Concentrations of sphingomyelin species in the grey matter of the dorsomedial prefrontal cortex. Data was assessed for normality using a D'Agostino Pearson Omnibus test. Data which was normally distributed was analysed using an unpaired t-test with Welch's correction, whilst data that did not fit normality assumptions was analysed using a Mann Whitney U test. P values are shown with corresponding significance. Data is in pmol lipid per mg tissue. The adjusted p values (q values) for multiple comparisons corrections using a two-stage linear step-up Benjamini, Krieger, and Yekutieli method have been included for the readers information.

	**CON**			**HD**								
**Grey Cortex**	**Mean**	**SEM**	**n**	**Mean**	**SEM**	**n**	**PD(%)**	**t ratio**	**df**	**MW-U**	**p**	**q**
SM d18:1/16:0	279.17	24.36	13	255.45	26.87	13	-8.50	0.65	23.77		0.4444	0.5494
SM d18:1/18:0	3390.80	167.73	13	3173.67	221.87	13	-6.40	0.78	22.34		0.3686	0.4804
SM d18:1/18:1	371.56	31.87	13	335.53	32.02	13	-9.70	0.80	24.00		0.3996	0.4804
SM d18:1/19:0	43.21	2.59	12	38.34	3.11	11	-11.26	1.20	19.94		0.2110	0.2919
SM d18:1/20:0	1046.44	77.12	13	841.07	47.39	13	-19.63	2.27	19.93		0.0324	0.2642
SM d18:1/20:1	52.48	3.07	12	45.67	3.12	12	-12.98	1.55	21.99		0.1228	0.2642
SM d18:1/22:0	134.20	12.48	13	132.06	21.97	13	-1.59			63.00	0.6276	0.7426
SM d18:1/22:1	78.01	13.42	8	79.22	20.14	8	1.56			25.00	0.7812	0.7940
SM d18:1/23:0	116.26	20.04	12	123.21	40.94	10	5.98			50.00	0.6567	0.7940
SM d18:1/23:1	98.46	20.37	10	139.00	40.31	6	41.17			21.00	0.3123	0.7426
SM d18:1/24:0	271.42	49.27	13	246.84	86.77	13	-9.06			62.00	0.3638	0.7426
SM d18:1/24:1	2006.89	459.82	13	1568.22	530.03	13	-21.86			61.00	0.2693	0.7426
SM d18:1/24:2	101.61	15.42	10	104.07	23.99	9	2.42			41.00	0.8000	0.8343
SM d18:1/25:0	118.06	18.69	10	130.19	45.42	7	10.28			31.00	0.8157	0.8343
SM d18:1/25:1	448.04	104.50	13	361.20	125.46	13	-19.38			65.00	0.2914	0.7426
SM d18:1/26:1	319.49	62.35	12	252.22	88.24	12	-21.05			47.00	0.1490	0.7426
**Total Sphingomyelin**	**7685.35**	**944.84**	**13**	**6730.23**	**1201.56**	**13**	**-12.43**			**58.00**	**0.2597**	**0.7426**

Abbreviations: df degrees of freedom; **HD** Huntington's Disease; **MW-U** Mann Whitney U Rank; **PD** Percentage Difference (HD to Control); **SEM** Standard Error of Mean.

Supplementary Table 32 Concentrations of HexCer species in the grey matter of the dorsomedial prefrontal cortex. Data was assessed for normality using a D'Agostino Pearson Omnibus test. Data which was normally distributed was analysed using an unpaired t-test with Welch's correction, whilst data that did not fit normality assumptions was analysed using a Mann Whitney U test. Data is in pmol lipid per mg protein. The adjusted p values (q values) for multiple comparisons corrections using a two-stage linear step-up Benjamini, Krieger, and Yekutieli method have been included for the readers information.

	**CON**			**HD**								
**Grey Cortex**	**Mean**	**SEM**	**n**	**Mean**	**SEM**	n	**PD (%)**	**t ratio**	**df**	**MW-U**	**p**	**q**
HexCer d18:1/16:0	129.25	33.06	13	265.68	78.22	13	105.55	1.61	16.15		0.1211	0.2642
HexCer d18:1/18:0	3933.08	1064.10	13	6843.03	2051.33	13	73.99	1.26	18.02		0.2201	0.2919
HexCer d18:1/20:0	324.93	76.56	13	675.10	198.88	13	107.77	1.64	15.48		0.1133	0.2642
HexCer d18:1/22:0	834.43	236.28	13	1998.84	623.34	13	139.55	1.75	15.38		0.0935	0.2642
HexCer d18:1/23:0	1249.45	352.03	13	3127.80	998.12	13	150.33	1.78	14.94		0.0886	0.2642
HexCer d18:1/24:0	2786.17	780.36	13	7132.05	2273.72	13	155.98	1.81	14.79		0.0832	0.2642
HexCer d18:1/24:1	16183.39	5099.03	13	31372.34	9803.10	13	93.86	1.38	18.05		0.1820	0.2713
HexCer d18:1/25:0	957.26	249.14	13	2085.43	646.57	13	117.85	1.63	15.49		0.1165	0.2642
HexCer d18:1/26:0	208.33	53.01	13	380.22	111.47	13	82.51			73.0	0.1769	0.7940
HexCer d18:1/26:1	2796.15	863.98	13	5942.17	1868.74	13	112.51	1.53	16.91		0.5114	0.2642
**Total HexCer**	**29402.45**	**8728.61**	**13**	**59822.66**	**18553.60**	**13**	**103.46**	**1.64**	**16.08**		**0.1509**	**0.2642**

Abbreviations: df degrees of freedom; **HD** Huntington's Disease; **MW-U** Mann Whitney U Rank; **PD** Percentage Difference (HD to Control); **SEM** Standard Error of Mean.

Supplementary Table 33 Concentrations of Hex2Cer species in the grey matter of the dorsomedial prefrontal cortex. Data was assessed for normality using a D'Agostino Pearson Omnibus test. Data which was normally distributed was analysed using an unpaired t-test with Welch's correction, whilst data that did not fit normality assumptions was analysed using a Mann Whitney U test. P values are shown with corresponding significance. Data is in pmol lipid per mg protein. The adjusted p values (q values) for multiple comparisons corrections using a two-stage linear step-up Benjamini, Krieger, and Yekutieli method have been included for the readers information.

	CON			HD								
**Grey Cortex**	**Mean**	**SEM**	**n**	**Mean**	**SEM**	n	**PD (%)**	**t ratio**	**df**	**MW-U**	**p**	**q**
Hex2Cer d18:1/16:0	38.28	6.70	13	64.00	9.85	13	67.21	2.16	21.13		0.0410	0.2919
Hex2Cer d18:1/18:0	311.42	61.00	13	462.32	108.58	13	48.45	1.21	18.89		0.2382	0.2642
Hex2Cer d18:1/22:0	39.46	9.12	13	88.55	24.66	13	124.37	1.87	15.22		0.0740	0.2642
Hex2Cer d18:1/24:0	110.88	28.50	13	300.86	94.10	13	171.33	1.93	14.18		0.0650	0.2642
Hex2Cer d18:1/24:1	505.93	136.75	13	1078.04	327.91	13	113.08	1.61	16.05		0.1203	0.2642
Hex2Cer d18:1/25:0	54.07	14.61	13	146.34	47.30	13	170.66	1.86	14.27		0.0746	0.2642
Hex2Cer d18:1/26:0	12.44	3.12	13	29.20	9.20	13	134.61	1.72	14.72		0.0979	0.2642
Hex2Cer d18:1/26:1	146.31	41.11	13	321.40	101.42	13	119.68	1.60	15.84		0.1226	0.8343
**Total Hex2Cer**	**1218.80**	**298.89**	**13**	**2490.70**	**714.11**	**13**	**104.36**	**1.64**	**15.17**		**0.1135**	**0.2642**

Abbreviations: df degrees of freedom; **HD** Huntington's Disease; **MW-U** Mann Whitney U Rank; **PD** Percentage Difference (HD to Control); **SEM** Standard Error of Mean.

Supplementary Table 34 Concentrations of sulfatide species in the grey matter of the dorsomedial prefrontal cortex. Data was assessed for normality using a D'Agostino Pearson Omnibus test. Data which was normally distributed was analysed using an unpaired t-test with Welch's correction, whilst data that did not fit normality assumptions was analysed using a Mann Whitney U test. Data is in pmol lipid per mg protein. The adjusted p values (q values) for multiple comparisons corrections using a two-stage linear step-up Benjamini, Krieger, and Yekutieli method have been included for the readers information.

	**CON**			**HD**								
**Grey Cortex**	**Mean**	**SEM**	**n**	**Mean**	**SEM**	n	**PD (%)**	**t ratio**	**df**	**MW-U**	**p**	**q**
(3'-sulfo)Galβ-Cer(d18:1/16:0)	10.47	1.08	13	14.70	2.91	13	40.39			76.5	0.1786	0.2713
(3'-sulfo)Galβ-Cer(d18:1/18:0)	278.29	64.34	13	771.46	342.13	13	177.22	1.42	12.85		0.5114	0.2642
(3'-sulfo)Galβ-Cer(d18:1/20:0)	69.66	17.99	13	172.53	55.88	13	147.67	1.75	14.46		0.0925	0.2642
(3'-sulfo)Galβ-Cer(d18:1/22:0)	270.73	63.03	13	635.35	194.29	13	134.68	1.79	14.50		0.0869	0.2642
(3'-sulfo)Galβ-Cer(d18:1/23:0)	420.52	106.43	13	1064.65	344.53	13	153.17	1.79	14.27		0.0867	0.2642
(3'-sulfo)Galβ-Cer(d18:1/24:0)	1350.56	329.45	13	3137.35	985.66	13	132.30	1.72	14.65		0.0985	0.2713
(3'-sulfo)Galβ-Cer(d18:1/24:1)	1492.15	405.33	13	2780.53	841.34	13	86.34	1.38	17.29		0.1804	0.2919
(3'-sulfo)Galβ-Cer(d18:1/18:0(2OH))	21.59	4.78	13	35.30	10.04	13	63.48	1.23	17.18		0.2317	0.2713
(3'-sulfo)Galβ-Cer(18:1/20:0(2OH))	53.63	8.15	13	76.43	14.72	13	42.50	1.35	18.72		0.1912	0.2642
(3'-sulfo)Galβ-Cer(d18:1/22:0(2OH))	399.41	89.02	13	720.96	199.96	13	80.50	1.47	16.58		0.1547	0.2642
(3'-sulfo)Galβ-Cer(d18:1/23:0(2OH))	875.32	215.94	13	1855.52	570.02	13	111.98	1.61	15.37		0.1210	0.2713
(3'-sulfo)Galβ-Cer(d18:1/24:0(2OH))	1786.12	383.31	13	3011.34	828.27	13	68.60	1.34	16.91		0.1920	0.3531
(3'-sulfo)Galβ-Cer(d18:1/24:1(2OH))	1886.21	499.41	13	2892.17	807.29	13	53.33	1.06	20.01		0.2998	0.2713
**Total Sulfatide**	**3892.38**	**982.58**	**13**	**8576.57**	**2680.78**	**13**	**120.34**	**1.33**	**17.50**		**0.1139**	**0.2713**
**Total OH-Sulfatide**	**5022.28**	**1188.05**	**13**	**8591.71**	**2412.36**	**13**	**71.07**	**1.51**	**16.22**		**0.1969**	**0.2642**
**Total Combined Sulfatide**	**8914.66**	**2151.03**	**13**	**17168.28**	**5046.26**	**13**	**92.58**	**1.51**	**16.22**		**0.1455**	**0.2642**

Abbreviations: df degrees of freedom; **HD** Huntington's Disease; **MW-U** Mann Whitney U Rank; **PD** Percentage Difference (HD to Control); **SEM** Standard Error of Mean.

Supplementary Table 35 Expression of housekeeping proteins before adjustment for ceramide synthase expression. Data was assessed for normality using a D'Agostino Pearson Omnibus test and then tested using an unpaired t-test with Welch's correction or a Mann Whitney U test where appropriate. **p<0.01, ***p<0.001. The adjusted p values (q values) for multiple comparisons corrections using a two-stage linear step-up Benjamini, Krieger, and Yekutieli method have been included for the readers information.

**Housekeeper Expression**		**CON**			**HD**							
		**Mean**	**SEM**	**N**	**Mean**	**SEM**	**N**	**t ratio**	**df**	**MW-U**	**p**	**q**
Caudate (CerS1)	β actin	1.067	0.104	12	0.874	0.069	13			46	0.2351	0.1583
	GAPDH	0.905	0.049	12	0.973	0.045	12	1.03	21.82		0.3143	0.6349
Putamen (CerS1)	β actin	1.170	0.139	13	0.654	0.074	13			22	0.0008**	0.0016
	GAPDH	1.025	0.088	13	1.005	0.070	13	0.1807	22.82		0.8582	0.9847
Caudate (CerS2)	β actin	0.954	0.033	12	1.053	0.037	13	2.201	21.46		0.0388	0.1569
	GAPDH	1.015	0.053	12	0.995	0.028	13			60	0.3475	0.1755
Putamen (CerS2)	β actin	1.067	0.038	13	0.916	0.026	13			25	0.0016**	0.0016
	GAPDH	0.994	0.035	13	0.992	0.037	13	0.03178	23.94		0.9749	0.9847

Abbreviations: **CerS1** Ceramide Synthase 1, **CerS2** Ceramide Synthase 2, **CON** Control, **HD** Huntington's disease, **GAPDH** Glyceraldehyde 3-phosphate dehydrogenase, **SEM** Standard Error of Mean.

Supplementary Table 36 Relative Expression of Ceramide Synthases in Control and HD Caudate. Data was assessed for normality using a D'Agostino Pearson Omnibus test and then tested using an unpaired t-test with Welch's correction or a Mann Whitney U test where appropriate. **p<0.01. The adjusted p values (q values) for multiple comparisons corrections using a two-stage linear step-up Benjamini, Krieger, and Yekutieli method have been included for the readers information.

	**CON**			**HD**								
	**Mean**	**SEM**	**n**	**Mean**	**SEM**	**n**	**PD (%)**	**t ratio**	**df**	**MW-U**	**p**	**q**
Ceramide Synthase 1 (β-actin)	2.15	0.72	12	0.91	0.03	13	-57.60			57.0	0.0025**	0.9747
Ceramide Synthase 2 (β-actin)	0.95	0.04	12	1.11	0.05	13	17.27	2.54	21.08		0.0189	0.4673
Ceramide Synthase 1 (GAPDH)	1.25	0.09	11	0.96	0.05	13	-23.34	2.92	16.50		0.0098**	0.2865
Ceramide Synthase 2 (GAPDH)	1.01	0.05	12	1.05	0.05	13	4.41	0.60	22.62		0.5558	0.9993

Abbreviations: **CON** Control; **HD** Huntington's disease; **df** degrees of freedom; **GAPDH** Glyceraldehyde 3-phosphate dehydrogenase; **MW-U** Mann Whitney U Rank; **PD** Percentage Difference (HD to Control); **SEM** Standard Error of Mean.

Supplementary Table 37 Relative Expression of Ceramide Synthases in Control and HD Putamen. Data was assessed for normality using a D'Agostino Pearson Omnibus test and then tested using an unpaired t-test with Welch's correction or a Mann Whitney U test where appropriate. The adjusted p values (q values) for multiple comparisons corrections using a two-stage linear step-up Benjamini, Krieger, and Yekutieli method have been included for the readers information.

	CON			HD								
	**Mean**	**SEM**	**n**	**Mean**	**SEM**	**n**	**PD (%)**	**t ratio**	**df**	**MW-U**	**p**	**q**
Ceramide Synthase 1 (β-actin)	2.08	0.96	11	2.16	1.08	13	4.10			61.0	0.3470	0.4904
Ceramide Synthase 2 (β-actin)	1.01	0.03	13	1.03	0.05	13	2.45	0.45	20.13		0.6533	0.6404
Ceramide Synthase 1 (GAPDH)	2.36	1.06	11	0.66	0.19	12	-72.17			43.0	0.0439	0.3617
Ceramide Synthase 2 (GAPDH)	1.03	0.04	13	1.03	0.04	13	-0.38	0.07	22.90		0.9460	0.8376

Values which were excluded from analysis due to housekeepers are highlighted. **Abbreviations**: **CON** Control, **HD** Huntington's disease, df degrees of freedom; **GAPDH** Glyceraldehyde 3-phosphate dehydrogenase, **MW-U** Mann Whitney U Rank; **PD** Percentage Difference (HD to Control); **SEM** Standard Error of Mean.

Supplementary Table 38 Pearson's correlations of ceramide synthases with Age at Death for control and HD subjects in the caudate. Ceramide synthase expression adjusted for β-actin for CerS1 and GAPDH for CerS2.

Caudate	N	R	R^2	95% CI	P value
CerS1 vs Age at Death (CON)	12	0.4873	0.2374	-0.1202 to 0.8293	0.1081
CerS1 vs Age at Death (HD)	13	0.7251	0.5258	0.2900 to 0.9118	0.0050**
CerS2 vs Age at Death (CON)	12	-0.2251	0.0507	-0.7076 to 0.4006	0.4818
CerS2 vs Age at Death (HD)	13	0.1279	0.0164	-0.4551 to 0.6342	0.6771

Abbreviations: CerS1 Ceramide Synthase 1, **CerS2** Ceramide Synthase 2, **CI** Confidence Interval, **CON** Control, **HD** Huntington's Disease

Supplementary Table 39 Pearson's correlations of ceramide synthases with Age at Death for control and HD subjects in the putamen. Ceramide synthase expression adjusted for GAPDH.

Putamen	N	R	R^2	95% CI	P value
CerS1 vs Age at Death (CON)	11	0.5401	0.2917	-0.0885 to 0.8610	0.0864
CerS1 vs Age at Death (HD)	12	0.4945	0.2445	-0.2024 to 0.8010	0.1462
CerS2 vs Age at Death (CON)	13	0.4267	0.1820	-0.1625 to 0.7916	0.1460
CerS2 vs Age at Death (HD)	13	0.1829	0.0334	-0.4094 to 0.6667	0.5499

Abbreviations: CerS1 Ceramide Synthase 1, **CerS2** Ceramide Synthase 2, **CI** Confidence Interval, **CON** Control, **HD** Huntington's Disease.

Supplementary Table 40 Correlations for Additional Banding of CerS2 in HD Subjects. A Pearson's correlation matrix was used to determine relationships. No significant correlations were identified for subjects with additional banding.

**Caudate Correlations for CerS2**					
**Pearson (α=0.05)**	**CerS2 (Primary)**	***vs* Age at Death**	***vs* Brain pH**	***vs* Post-Mortem Interval**	***vs* CAG**
**r**		0.4315	-0.1768	-0.0342	-0.3471
**95% confidence interval**		-0.1567 to 0.7938	-0.6632 to 0.4145	-0.5744 to 0.5267	-0.7539 to 0.2521
**R squared**		0.1862	0.0313	0.0012	0.1205
**P value**					
**P (two-tailed)**		0.1409	0.5633	0.9116	0.2453
**Sig.**		*ns*	*ns*	*ns*	*ns*
**Number of XY Pairs**		13	13	13	13
	**CerS2 (Secondary)**	***vs* Age at Death**	***vs* Brain pH**	***vs* Post-Mortem Interval**	***vs* CAG**
**r**		-0.1706	-0.2279	0.3343	0.2216
**95% confidence interval**		-0.6596 to 0.4199	-0.6920 to 0.3695	-0.2657 to 0.7476	-0.3752 to 0.6885
**R squared**		0.02909	0.05194	0.1117	0.04909
**P value**					
**P (two-tailed)**		0.5775	0.4539	0.2643	0.4669
**Sig.**		*ns*	*ns*	*ns*	*ns*
**Number of XY Pairs**		13	13	13	13
	**CerS2 (Tertiary)**	***vs* Age at Death**	***vs* Brain pH**	***vs* Post-Mortem Interval**	***vs* CAG**
**r**		0.07784	-0.2094	0.3992	-0.05728
**95% confidence interval**		-0.4943 to 0.6030	-0.6817 to 0.3861	-0.1946 to 0.7788	-0.5897 to 0.5098
**R squared**		0.006059	0.04385	0.1593	0.003281
**P (two-tailed)**		0.8005	0.4923	0.1767	0.8525
**Sig.**		*ns*	*ns*	*ns*	*ns*
**Number of XY Pairs**		13	13	13	13

Abbreviations: CerS2 Ceramide Synthase 2, HD Huntington's disease, *ns* not significant, Sig. Significance.
