# Supplementary material for: The long and the short of Huntington’s disease: how the sphingolipid profile is shifted in the caudate of advanced clinical cases
Source: Brain Commun. 2021 Dec 23;4(1):fcab303. doi: 10.1093/braincomms/fcab303 (PMC8833324; doi:10.1093/braincomms/fcab303)
Supplement: fcab303_Supplementary_Data [file Original Submission-Authors Response.pdf › Original Submission-Authors Response.pdf]

We would like to thank the reviewers for their comments that have helped us to improve our manuscript. We have addressed these comments below.

In response to Reviewer One,

**Comment 1. A key issue is whether the sphingolipid profile changes observed are secondary to neuronal cell loss (and associated gliosis). One way to address this is to attempt to analyse according to Vonsattel grading (as a surrogate of cell loss estimates) in the tissues used. Another complementary approach would be to study additional markers (such as NeuN for neurons, S-100-beta for astrocytes, etc.) that might indicate the amount of neuronal cell loss, and gliosis in the tissue samples used.**

All HD subjects in this study had Vonsattel pathological gradings of IV, the most severe (described in the methods, lines 163, page 6). Therefore, we cannot relate the observed changes to different pathological severities. Despite the value provided by measuring lipids directly in the brains of human subjects with HD, the authors acknowledge that using this approach we cannot determine whether the observed lipid changes are secondary to or a precursor of neuronal and glial loss. While the measurement of NeuN and s-100B may provide additional information regarding neuronal and astrocyte number, it will not allow us to determine whether the sphingolipid changes are secondary to neuronal cells loss or gliosis, and we have no brain tissue left to allow this analysis.

We have acknowledged limitations to our use of post-mortem tissue in line 459 (page 16): *'Since our lipid extractions are from whole brain tissue, it is difficult to determine whether these changes are due specifically to defects in axons, synapses, or myelin.'*, in line 534-536 (page 18): *'In addition. it should also be noted these findings are applicable specifically to Vonsattel grade IV pathologies.'* and on page 19 (lines 573-575): *'Future studies are required to determine whether the detected changes in sphingolipids are casual or secondary to a pathology including neuronal loss or mitochondrial composition changes'.*

**Comment 2. The authors use β-actin and GAPDH as Western blot housekeeper proteins. β-actin was excluded in some regions in the results. Are the authors confident neither is affected by HD? It would be informative if they could use total protein staining as an additional control for loading.**

The authors believe GAPDH to be a reliable housekeeper. GAPDH[1–4] and β-actin[5–8] have been used as housekeepers for western blot analysis in HD, including cell, murine and post-mortem analysis. The authors are aware of literature that indicates an association of GAPDH with mHTT and that this may increase the expression of GAPDH in mitochondria[9–11]. However, the expression of GAPDH was tested in this study before being used as a housekeeper, and expression was not different for HD in both the caudate and putamen compared to controls (Supplementary Table S35). Despite β-actin being used as a housekeeper in previous published studies, we did detect a lower expression of this protein in HD putamen. Therefore, it was not used as a housekeeper, which is reported in the manuscript (lines 382-386, page 13). Because of the consistency of lower CerS1 detected in HD caudate when normalised for both GAPDH and β-actin, the authors believe the reduction in CerS1 in HD caudate to be a reliable discovery.

**Comment 3. Are there any gender differences in their results?**

The authors acknowledge that gender is an important factor to consider in neurological conditions such as Huntington's disease. However, a sample size of 13 per group (8 males, 5 females) is not large enough to detect the effect of gender in this study. The authors have included an acknowledgement of this in the discussion in line 547 (page 18-19): *'Future post-mortem studies would benefit from using larger sample sizes with the intention of examining possible gender effects on brain chemistry in Huntington's disease. Due to limited statistical power (n=8 males, n=5 females per group), the influence of gender could not be determined.'*
The data in the supplementary material (Excel) has been separated by gender for the readers information.

**Comment 4. A concern with Figures 1-4 is that presenting the data in this way means that it is impossible to judge the variance in Control samples. I would like to see control samples plotted and their variance**

**represented. Another aspect of this data representation is that individual data points cannot be visualised. Can dot plots be overlayed, as they have been in Figure 5, etc? I realise that it may not be considered appropriate for such 'violin plots' but visualising individual data points is highly informative.**

Initially, dot plots were chosen to represent the data. However, the number of species combined with the considerable differences in abundance made it challenging for readers to delineate effects between species, which we felt was very important for the paper's message. Violin plots were then chosen. These plots used in figures 2-4 allow the reader to visualise the minimum and maximum values, the median, and the first and third quartiles. Notably, the shape of the violin indicates the distribution and variance of data points. We believe these plots were the best medium for our data to be communicated. The individual subject data for all lipids will be available to the reader as a supplementary excel file.

Each figure caption explains the violin plots: e.g., for figure caption 2, *'The violin plots show the distribution of values, medians (white line) and quartiles (first and third)'* on page 30 (line 859).

**Comment 5. As the HD cases have low (close to 40) CAG repeat lengths, and therefore presumably late age of clinical HD onset, the matched controls are also fairly old (up to 78 years). Are the authors confident that the Controls didn't have any neurodegenerative pathologies that might confound the study?**

The Victorian Brain Bank confirmed the absence of any neurological pathologies or diseases in the control subjects.

**Comment 6. Do the authors have any additional data for Table 1, such as cause of death and agonal state? If the HD cases died of different terminal conditions (e.g. respiratory failure versus cardiac failure), it might contribute to post-mortem molecular findings.**

The authors do not have information concerning the agonal state of the subjects; however, the cause of death has been added to Table 1. HD patients typically died from respiratory failure (e.g., pneumonia), whilst control subjects typically died from cardiac failure (e.g., heart disease). It appears both events result in brain death via a loss of oxygen to the brain. There is no available literature examining the lipid changes in the brain following either of these types of death. Therefore, the authors can only provide the reader with the cause of death and cannot determine if it influenced the lipid differences.

**Comment 7. Perhaps the authors could extend the discussion to make detailed comparisons between their findings and transgenic mouse models of HD, where potential confounds associated with human post-mortem studies are not present. Alignment with the preclinical animal studies provides an important 'sanity check' and potential mechanistic insights regarding 'cause and effect' considerations mentioned above.**

We have included a paragraph to compare the available data from these models against the study's findings. Ganglioside data is challenging to compare as species are generally not reported.

The following paragraph has been included on page 18 (lines 537-547):

'*Transgenic mouse models are a valuable tool for examining the changes to sphingolipid metabolism at earlier disease stages and in tandem with disease progression. There is limited information on the changes to the lipids studied in this research in these models; however the findings generally align with the current data. In R6/2 transgenic mice, very long chain ceramides are increased in the striatum (Cer 24:0) and cortex (Cer 20:0, Cer 22:0, Cer 24:0, Cer 24:1)*[12]*. In the same models, the mRNA expression of CerS1 is lower in the striatum and cortex*[13]*. In R6/1 mouse models, cerebrosides (Hex-Cer) and sulfatides*[14] *are decreased in the forebrain, and sulfatides decreased in cerebellum*[15]*. Identified alterations in gangliosides in transgenic mouse models are difficult to compare against the results from this study, as individual species are often not reported*[14–16]*.'*

In response to Reviewer Two,

**Comment 1. The analysis of sphingolipid species in the brain of HD patients is timely and potentially important, as several studies have suggested that altered metabolism of sphingolipids and glycosphingolipids occurs in HD, Parkinson and other neurodegenerative disorders. In its current form, however, the study has a quite narrow descriptive scope. The more mechanistic data on CerS are quite preliminary, and the proposed implications and relevance of the findings to HD pathology remain speculative. The manuscript presents a few other shortcomings that are outlined in more details below.**

**The mechanistic link between the observed changes in sphingolipid species and CerS expression /activity is quite underdeveloped and unclear. In spite of an increase in C16 and C18 species in the caudate, the levels of CerS1 are decreased, while the levels of CerS2 are not changed in spite of a decrease in >C22 species. The authors wisely suggest that protein levels may not correlate with enzyme activity and sphingolipid levels since CerS can be regulated by post-translational modifications and dimerisation. However, none of these possibilities is explored in the paper.**

The authors acknowledge that the manuscript submitted represents one of the first investigations into sphingolipid species alterations across multiple brain regions in Huntington's disease. The inclusion of CerS was secondary to the initial mass spectrometric analysis of sphingolipids in Huntington's brain tissue and was included to examine the two most abundant species of CerS in the brain, both of which are related to the altered sphingolipid species chain lengths (C16-C20, C22-C26). There is very little data on the expression of CerS in the human brain, and reliable antibodies were challenging to acquire, optimise and validate. The use of post-mortem tissue removes the ability to examine enzyme activity.

In reference to the dimerisation of CerS. The authors are aware of some literature indicating dimerisation of CerS and the modification of their fatty acyl chain preferences in accordance with these interactions. However, our analysis of CerS1 and CerS2 did not indicate the presence of these dimers. Each antibody was optimised using reduced and non-reduced conditions. There was no difference in the western blots between reduced and non-reduced states (see **Supplementary Figure S1** for CerS2 comparison blot). As indicated in Supplementary Figure S1, there is no detection of bands between 70-100 kDa where we would expect a dimer to appear. While the authors acknowledge these dimers' possible existence, we did not detect any in our analysis and comment only on the monomers.

We have recently recruited assistance on the post-translational modification of CerS with experts on such modifications from Vanderbilt University. Whilst we hypothesise that post-translational modifications are important, we consider the structural analysis of the CerS proteins to be beyond the scope of the current manuscript. It is, however, likely to be the focus of a future manuscript once the data from our new collaboration has been collected and analysed. The focus of the current manuscript is the striking changes in sphingolipid concentrations, and the CerS have been included as complementary material for the reader's information.

**Comment 3. Furthermore, only CerS1 and CerS2 levels are measured, but no CerS5, which would be specific for the synthesis of C16 species that are increased in the HD caudate. Dimerisation with other CerS has been shown to modulate the activity of CerS1 and CerS2, so measuring the levels of other CerSs and perform analysis in native gel conditions would help to elucidate the specific CerS dysfunctions in HD brains.**

In terms of CerS5, whilst the authors acknowledge this is a key player in the metabolism of long chain sphingolipids, we could not find a reliable antibody with appropriate positive controls. The possible influence of other CerS, including CerS5, has been acknowledged in the manuscript on page 17 (line 499): *'An increased expression of other relevant CerS isoforms (CerS4, CerS5, CerS6) may have also contributed.'*

In response to dimerisation, please refer to our response to Reviewer 2 Comment 1.

**Comment 4. The quantification (densitometric analysis) of CerS1 in Fig. 2 is quite puzzling. The signal for HD tissue is extremely weak compared to control tissue, yet the densitometric analysis shows only a modest reduction in CerS1 expression in HD compared to controls. How is that possible? There is also significant variability in protein abundance among control tissues, which does not seem to be reflected by the spread of data points in the relative graphs.**

Whilst we agree that there is a stark contrast between the expression of CerS1 in HD *vs* controls in the western blots when these blots were normalised to the expression of their representative pooled sample (to standardise measurements between membranes) and by housekeepers, the contrast is considerably less stark. All full western blots, including housekeepers, have been included in the Supplementary Material. Please see **Supplementary Figures S6 to S9** for full western blot images.

**Comment 5. Have the authors performed power analysis for this study? Considering the interindividual variability in some of the measurements reported (e.g. CerS levels), one wonders whether 13 samples per group is sufficient. It would also help to see a comparative analysis between males and female to determine whether any difference observed is dependent on sex.**

Power calculations have been conducted, and a sample size of thirteen participants per group was deemed substantial enough to detect changes of at 30% or greater. Reviewing the detected changes in the data, we can see that we are able to detect at least a 30% difference between the groups, even with an alpha of 0.01. Moreover, a sample size of 13 is either on par or larger than previous lipid studies in Huntington's disease post-mortem tissue. *See* Desplats et al., *Neurobiology of Disease*, 2007 (HD n=3)[14], Di Pardo et al., *Scientific Reports*, 2017 (HD n=3)[12], Denny et al., *Journal of Neurochemistry*, 2010 (HD n=3)[15], Hunter et al., *Journal of Neurochemistry*, 2018 (HD n=4)[17]. The following sentence has been added to the methods section 'Statistics' on page 10 (line 275-280):

*'A priori sample size calculation was conducted in JMP (v15, JMP, Lane Cove, Australia), using the mean and standard deviation of previous sphingolipid analysis in control post-mortem brain (unpublished). Using an alpha of 0.05, the priori determined 13 per group sufficient to detect at least a 30% difference between groups with 80% power. Previous post-mortem studies of human Huntington's brain has used between three[15,17] and fourteen[18] subjects per group for lipid analysis.'*

In response to any gender-specific effects on lipids, please see the above response to Reviewer One (comment 3).

**Comment 6. The authors claim that the observed increase in C16 and C18 ceramide species might increase susceptibility of cells to apoptosis. Yet, higher levels of CerS1, which would be responsible for the synthesis of C18 species, correlate positively with age at death in HD patients. This is in apparent contradiction with the increase in C16 and C18 species and their suggested relevance to HD and, therefore would need to be explained.**

Increases in long chain ceramide species (particularly C16), which occurred in the caudate and putamen, are associated with neurodegenerative conditions and provocation of apoptosis. CerS1 has a specific affinity for C18 and C20 fatty acyl chain lengths, so decreases in the CerS1 protein can coincide with the C16 increases. The authors stated the evidence of changes in sphingolipid chain length composition in the promotion of apoptosis in cell models (line page) and have not explicitly tied CerS1 expression to apoptosis. The lower expression of CerS1 would coincide well with neuronal loss in Huntington's disease as it is primarily expressed in these cells. In terms of increases in C18 and C20 chain lengths, these changes could result from an impaired degradation or recycling of sphingolipids. There is an also overlap in the chain length specificities of CerS reported in the literature.

**Comment 7. It is not clear why the authors present the correlation between CerS levels and CAG repeats and age at death, when there is clearly no correlation between the shift in sphingolipid species and levels of CerS1 and CerS2. Why not investigating the correlation between sphingolipid species (changes) and CAG repeats and age at death, to more directly investigate the role of this lipid changes in HD pathogenesis?**

The authors thank the reviewer for their comment and acknowledge its significance. As previously stated, CerS expression may not always correlate directly with sphingolipid concentrations due to the overlapping specificity of CerS and the numerous recycling and degradation pathways involved in regulating sphingolipid concentrations. The relationship between CAG repeat length and lipid concentrations was investigated in all five brain regions but was not initially included in the manuscript. We believed we would see a relationship because of studies by Burke et al., 2013[19] (*BBA Biomembranes*) and Kegel et al., 2009[20] (*Journal of Neurochemistry*) reporting a relationship of mHTT

polyglutamine length to lipid interference. We found no relationships between any lipid and CAG repeat length in HD patients. We hypothesise that mHTT may indirectly affect lipids via an association with proteins involved in lipid metabolism, which is suggested by the correlation between CAG repeat length and CerS1 expression. We have now acknowledged this analysis in the manuscript as a new section in the results on page 13 (lines 372-376):

'***CAG Repeat Length and Sphingolipid Concentrations***
*In vitro models suggest an influence of polyQ (CAG repeat) length on interference with lipid bilayers*[19,21]*. Pearson's correlations were used to investigate relationships between the concentration of sphingolipid species and the CAG repeat length of HD subjects in each brain region. No relationships were identified.'*

In addition, a paragraph reflecting on this has been added to the discussion on page 19 (lines 551-562):

*'Previous studies using in vitro models have shown polyQ length-dependent effects on the interaction of the mHTT protein in lipid bilayers*[19]*. Longer polyQ are reported to cause more significant disturbance and disorganisation of these bilayers*[19]*. Therefore, the relationship between the CAG repeat length of the mHTT gene and the abundance of lipids and related proteins (CerS) was explored. As mentioned previously, CAG repeat length and Age at Death were related to CerS1 expression in the caudate of Huntington's patients. However, no relationships were identified between CAG repeat length and any lipid abundances in Huntington's patients in any brain region. Previous analysis of plasma from Huntington's patients has also found CAG repeat length not to correlate with the abundance of lipid metabolites, instead, correlating with measures of functional capacity*[22]*. The underlying reason is unclear; however, it may be that mHTT interferes with lipids in vivo through interactions with lipid enzymes and not necessarily the lipids directly.'*

**Comment 8. In human brains, sphingolipids with d20:1 sphingoid long-chain base backbone are also present in addition to d18:1. These were not reported in this study. Were they not detected?**

The authors acknowledge that d20:1 backbones have been detected in gangliosides[23] and sphingosine-1-phopshate[24] in mouse brain and in gangliosides in human brain[25]. However, there is almost no information available on the presence of these species in ceramides, sphingomyelins, hexosylceramides or lactosylceramides in human brain. Colsch et al., (2004) identified the presence of d20:1 ceramide in mouse brain but did not report the quantity or abundance compared with d18:1 and d18:0 ceramide species[23]. The same study reported that these d20:1 backbones were not detected in sulfatides[23]. Considering these backbones are reported in sphingosine-1-phopshate and gangliosides, it would be expected that they would occur in ceramides, however with the rapid metabolism of ceramide, this would be difficult to predict. Our coauthors have not detected the prescence of d20:1 backbones previously in glycosphingolipids, only in gangliosides. In the human brain, d18:1 are reported to represent 81-98% of the sphingolipid backbones[26], although there has been little reported on these relative abundances in human brain since then (1970). While the authors acknowledge these species could be present in our chosen sphingolipids, the potential detection and quantification of these species would require a different mass spectrometry workflow (dissociation) and the use of a d20:1 deutaerated internal standard.

**Comment 9. In the discussion, some of the statements relative to the relevance and significance of the findings are too speculative based on the data presented. Furthermore, references are cited that do not appear to be relevant to the interpretation of data presented in the manuscript. For example, reference 65 in support of the statement that: "These increases in the abundance of long over very long chain sphingolipids may reflect changes to the lipid profile of neuronal membranes and/or synapses" is just a study that analyses various lipids in the soma and dendrites of neurons but does not investigate the species of sphingolipids.**

Reference 65 has been removed from the manuscript.
The authors have reviewed the discussion for speculative statements and removed or re-worded several sentences.

On page 17, the sentence '*Possible modifications to CerS2 in Huntington's caudate may have diminished these protective effects and could underlie the disturbances to the synthesis of very-long-chain ceramides, affecting the downstream availability of very long chain sphingomyelin and lactosylceramide species.'* has been removed and replaced with *'These cellular studies highlight the possible implications of any modifications to CerS2 in Huntington's caudate. Possible modifications to CerS2 could interfere with the synthesis of very long chain ceramides, affecting the downstream*

*availability to lactosylceramides and sphingomyelin. Disruptions in the balance of sphingolipid species could also contribute to the apoptotic susceptibility of cells.'*

On page 17 the sentence *'The high energy demands of the striatal primary neuronal cell type, medium spiny neurons, are proposed to be the underlying reason for this region's susceptibility to mitochondrial dysfunction in Huntington's disease(Pickrell et al., 2011).'* Has been removed. The authors have deemed it unnecessary.

On page 18, the sentence *'Increased sphingomyelin content limits the insertion of HTT (35Q) into lipid bilayers in cultured cells(Chaibva et al., 2018), so this may be a protective mechanism.'* Has been replaced with *'As the synthesis of sphingomyelin typically occurs in oligodendrocytes, the increases in sphingomyelin species in the putamen may be the result of these cells(Kilkus et al., 2008; Vos et al., 1995).'*

**Comment 10. Finally, because all HD brains were stage IV, whether any lipid change observed merely a consequence of the degenerative process (with decreased neurons and increased microglia and astroglia), rather than a contributor to pathophysiology remains to be determined. It would be interesting to determine whether the shift in the sphingolipid species occurs at earlier disease stages.**

The reviewer provides a pertinent statement. The following has been included in the discussion to include this consideration on page 18 (line 533-539):

*'It should be acknowledged the results presented are attributable to end-stage Huntington's disease and cannot reflect the dynamic nature of sphingolipid metabolism at earlier stages. Post-mortem analysis of Huntington's disease brain is typically limited to end-stage, except in cases where an alternative cause of death is apparent (e.g., suicide). Transgenic mouse models are a valuable tool for examining the changes to sphingolipid metabolism at earlier disease stages and in tandem with disease progression.'*

**Comment 11. The statement that a different phospholipid composition might influence brain region vulnerability to mutant HTT is speculative at this point and has not been demonstrated yet.**

We acknowledge that brain region lipid composition influence on the susceptibility to mHTT has not yet been proven *in vivo.* The sentence on page 1 (lines 12-15) has been changed to:

'*The polyQ mutation at the N-terminus of the huntingtin protein alters its natural interactions with neural phospholipids in vitro, suggesting that the specific lipid composition of brain regions could influence their vulnerability to interference by mutant huntingtin, however this has not yet been demonstrated in vivo.'*

**Comment 12. The authors are encouraged to abbreviate huntingtin using capital letters, HTT, according to more recent nomenclature.**

When referring to the Huntingtin protein, the abbreviation has been changed in the text to HTT and mHTT.

**Comment 13. Many functions have been attributed to HTT, beside its roles in neurodevelopment. This should be acknowledged in the introduction if the authors discuss HTT functions.**

The introduction has been updated to include other functions of HTT than neurodevelopment. Please see page 3 (lines 83-84):

*"HTT appears to play an important role in neurodevelopment[27], synaptic development, neuronal survival[28] and transport[29], transcriptional regulation[29] and autophagy[29,30], however, it is still debated as to whether Huntington's disease results from a 'loss of function' of HTT or a pathological 'gain of function' of mHTT."*

**Comment 14. In the abstract and introduction, the authors should rephrase their statement that the striatum is uniquely affected, as it might generate confusion. While the striatum is the most affected, other brain regions are also affected, as well as some peripheral tissues.**

The abstract and introduction have been amended (see highlighted regions pg. 1 and 4)

'*Although the mutant huntingtin protein is ubiquitously expressed, the striatum degenerates early and consistently in the disease*.' on page 1 (lines 11-12).

The sentence on page 4 (lines 87-89) have been changed from 'Although mHTT is expressed ubiquitously in the brain, it causes region-specific atrophy of the striatum and areas of the cerebral cortex[31,32]'. to '*Although mHTT is expressed ubiquitously in the brain, the atrophy of the striatum occurs early and severely in the disease*[32,33]*. The cerebral cortex also atrophies, although not to the same extent as the striatum*[32,34]*.' on page 4 (lines 87-89).*

**Comment 15. On page 5, it is not clear what the authors mean when they state that changes in the sphingomyelin profile can significantly affect ceramide concentrations.**

The sentence has been removed from page 5.

**Comment 16. The polyQ length in R6/1 is not necessarily exactly 115, as stated, due to CAG instability and strain drifting in different labs. Therefore, the authors are advised to remove this statement.**

The sentence on page 5 (line 140) has been modified from '*Transgenic Huntington's mice (R6/1; polyQ=115)...*' to '*Transgenic R6/1 Huntington's mice...'*.

**Comment 17. On page 11, the Authors state that the total abundance of each lipid class was compared between controls and HD subjects. Differences were noted for Hex2Cer in the cerebellum of HD subjects; however, the data are not presented. The authors should refer to a table that shows these differences (or lack of for other lipid species).**

A scatter plot of control *vs* HD sphingolipid totals has been included in the supplementary material as **Supplementary Figure S3**. It has replaced the original Supplementary Figure S3 as the new graph contains both controls and HD lipid totals.

**Comment 18. On page 11, when referring to the various ceramide and sphingomyelin species, the authors should refer to the table where these data are shown.**

The relevant supplementary tables for all lipid data have been included in all results sections, including those on page 11.

**Comment 19. In many instances a p value of 0.000 is reported. This should be changed to the actual p value.**

All p values in the text have been updated to exact p values (and q values) where appropriate.

**Comment 20. In Fig. 5, the molecular weights for protein standards should be indicated. The "Pool" should be defined.**

Figure 5 has been corrected to show kDa weights of protein standards. The caption has been modified to include the pool definition. Figure 5 has been altered:

***'Figure 5 The Expression of CerS1 and CerS2 in Control and HD Striatum.*** *The mean expression of CerS1 (~40 kDa) between control and HD subjects adjusted by (B) β-actin and (D) GAPDH expression in striatal subregions (orange circles for caudate; blue triangles for putamen). The mean expression of CerS2 (~ 40 kDa) between control and HD subjects*

*adjusted by (F) β-actin and (H) GAPDH expression in the same regions. Graphs display the mean, standard error of the mean, and the individual subject values. Representative western blots of CerS1 and CerS2 in the (A, E) caudate and (C, G) putamen with β-actin and GAPDH housekeepers. β-actin was excluded as a housekeeper for putamen samples as the expression was found to be significantly different in HD subjects as compared with controls. Samples were combined to create a pooled sample which was used to standardise measurements between membranes. Data was assessed for normality using a D'Agostino Pearson Omnibus test and then analysed with an unpaired t-test with Welch's correction or Mann Whitney U test. Accepted significance was set at p<0.01. Full western blots are provided in* ***Supplementary Figures S7-S10****. Statistical test information is available in* ***Supplementary Tables S36-S37****.* ***CerS1*** *Ceramide Synthase 1,* ***CerS2*** *Ceramide Synthase 2,* ***CON*** *Control,* ***GAPDH*** *Glyceraldehyde 3-phosphate dehydrogenase,* ***HD*** *Huntington's Disease,* ***P*** *Pool'* on page 31.

**Comment 21. Concerning CerS2, multiple bands (presumably differentially glycosylated forms of the protein) are present in some of the samples (mainly HD) but not others. Whether all bands or only the bottom one was used for quantification should be specified.**

The text on page 14 has been changed to:
*'No differences in the expression of the CerS2 primary band (~40 kDa) between control and HD samples were identified in either the caudate or the putamen '*on page 14 (lines 413-414).

The caption for Figure 5 has also been amended to include the weight of the bands used in quantification (see above).

In response to the comments put forth by the editorial office,

**Comment 1. In figures 2-4, you have used t-tests to analyse the data. Please re-visit and use appropriate corrections for multiple testing.**

We acknowledge the importance of correcting for multiple comparisons where results from many t-tests are reported. For accuracy of statistical testing, we have (i) identified outliers that skew data and removed these values (lowering power), (ii) applied Welch's corrections to all t-tests, and ANOVAs (lowering power), (iii) assessed normality of every lipid species to apply the most appropriate test (t-test or Mann Whitney U), (iv) visually inspected the distribution of all significant data and (v) lowered the acceptable p value threshold to <0.01. There are approximately 380 t-tests and Mann Whitney U tests used. Using a False Discovery Rate adjustment of 1% (two-stage Benjamini, Krieger, and Yekutieli) changes the number of significant p values reported from 34 to 4. Using a p value of <0.01, we would expect that of 380 tests, 3.8 of these would be false positives. This is a very different number from the rejected 30 as determined by a FDR of 1%.

In addition to these statistical considerations, we have consulted with a statistician who supports not correcting for multiple comparisons as the patterns in the data (specifically in the caudate) are consistent across multiple lipid classes (Cer, SM, Hex-2-Cer) and between associated disease measures (e.g., CerS1 with Age at Death and CAG repeat length). This is supported by the strong correlation coefficients presented (R= -0.6975; R = 0.7251), for which CerS1 would be considered non-significant after FDR corrections. Considering Reviewer Two has already commented on the stark differences of CerS1 in controls and HD on the western blots, it seems that adjusting the data by FDR and reporting it as non-significant does not seem correct practice. Whilst multiple comparisons testing reduces the chance of type I error, it increases the chances of type II error[35]. Increasing type II error is particularly problematic for exploratory studies like ours, which seek to identify molecules of interest in disease[36,37]. We sought to investigate the changes to sphingolipids across multiple brain regions to be investigate region-specific effects. Including additional brain regions in our manuscript has supported the region-specific nature of chain length changes to sphingolipids in the caudate but lowered our ability to detect significant differences (using FDR). In reporting more information in manuscripts, authors can be 'punished' with a reduction in statistical power when using multiple comparisons[35]. We believe the non-corrected p values are the most correct values to report as this manuscript highlights molecules for consideration in the pathogenesis of Huntington's disease.

The authors urge the editorial office to consider allowing the data to remain in the manuscript without relying on the adjusted p values. For transparency, we have included the adjusted p values (q values) for all statistical tests in the supplementary material and will be supplying the raw data values for all data in an Excel file. This has been previously done in exploratory manuscripts[38,39]. By providing all statistical information, and the original and adjusted p values, we believe the readers will have sufficient information to make informed decisions about the data presented. For ANOVAs, we have included the q values in the manuscript.

We have included this information in the statistical information section of the manuscript (lines 282-289, page 10):

'*Brown Forsythe and Welch's ANOVA tests were used to compare control regions and were adjusted for multiple comparisons using a two-stage linear step-up Benjamini, Krieger and Yekutieli method. T-test and Mann Whitney U tests were tested for multiple comparisons using a Benjamini, Krieger and Yekutieli False Discovery Rate of 1%, however 30 of the original 34 significant tests did not survive these corrections. As the purpose of this study was exploratory in nature, the authors have reported the data using the unadjusted p values in the manuscript but have included the adjusted p values (q) in the supplementary material for the readers information.*'

**Comment 2. For each statistical test, please report in the figure legend, the N, test used, relevant test statistic, and exact p values (for example F or t values for ANOVAs and t-tests) (exact p values missing for Figures 2-4) (N number missing for Figures 5-7) (missing statistical test in Figure 5).**

The captions for figures 2-7 have been amended to include n, calculation used to determine values (percentage differences), accepted statistical significance level and the tests used for analysis between groups. Exact p values are available for every test conducted in the supplementary material and all relevant supplementary tables have been included in each caption. The supplementary tables have been updated to include the degrees of freedom, t-ratio and Mann Whitney U rank where required. The authors believe the inclusion of all exact p values and test statistics will complicate the figure captions. For example, the figure 2 caption would need to include the p values, n, and df for eighteen tests. The captions and figures have been adjusted for clarity (e.g., y axes are now more comparable between lipid classes).

For example, Figure 2 Caption now reads:

'***Figure 2 Percentage differences of (A) ceramide, (B) sphingomyelin, (C) Hex-Cer (GluCer, GalCer), (D) Hex2Cer (lactosylceramide), (E) SHexCer and (F) OH-SHexCer species in the caudate of control (n=12) and HD subjects (n=12).*** *Arrows indicate metabolic pathways. Percentage differences were calculated as [((HD-Control)/(Control)) x 100]. Data was assessed for normality using a D'Agostino Pearson Omnibus test and analysed using an unpaired t-test with Welch's correction or Mann Whitney U test where appropriate. The violin plots show the distribution of values, medians (white line) and quartiles (first and third). Accepted significance was set at p<0.01. **p<0.01, ***p<0.001. Statistical test details including exact p values can be found in* ***Supplementary Tables S10-S14****.* ***Cer*** *Ceramide;* ***HD*** *Huntington's disease;* ***GalCer*** *Galactosyl-ceramide;* ***GluCer*** *Glucosylceramide;* ***SM*** *Sphingomyelin.'* when previously it read"

**Comment 3. In Figures 5-7, please clarify what is measured in Y-Axis.**

The Y-axis measurements have been clarified in the captions for figures 5-7. The y-axis measurements refer to the expression of the relevant protein (CerS1, CerS2) adjusted by the expression of the relevant housekeeper.

**Comment 4. Please supply a graphical abstract. A graphical abstract is a visual representation of the central finding or methodology of the paper. It will be displayed in the table of contents. Authors should strive to make the graphical abstract informative, interesting, visually appealing, and straightforward (no caption needed, no abbreviations except gene names). Please upload as a separate high resolution image file (tiff preferred). The technical requirements for the graphical abstracts are:**

**--size of the submitted image: 1200 pixels square at 300dpi**

**--font of the text: Arial 12-16 point (smaller fonts will not be legible online).**

The authors have supplied a graphical abstract to be included with the manuscript.

**References**


1. Sapp, E. *et al.* Native Mutant Huntingtin in Human Brain. *J Biol Chem* **287**, 13487–13499 (2012).
2. Sapp, E. *et al.* Protein changes in synaptosomes of Huntington's disease knock-in mice are dependent on age and brain region. *Neurobiology of Disease* **141**, 104950 (2020).
3. Daldin, M. *et al.* Polyglutamine expansion affects huntingtin conformation in multiple Huntington's disease models. *Sci Rep* **7**, 5070 (2017).
4. Mueller, K. A. *et al.* Hippo Signaling Pathway Dysregulation in Human Huntington's Disease Brain and Neuronal Stem Cells. *Sci Rep* **8**, 11355 (2018).
5. Schut, M. H. *et al.* Effect of post-mortem delay on N-terminal huntingtin protein fragments in human control and Huntington disease brain lysates. *PLOS ONE* **12**, e0178556 (2017).
6. Hwang, S., Disatnik, M.-H. & Mochly-Rosen, D. Impaired GAPDH-induced mitophagy contributes to the pathology of Huntington's disease. *EMBO Molecular Medicine* **7**, 1307–1326 (2015).
7. Carnemolla, A., Michelazzi, S. & Agostoni, E. PIN1 Modulates Huntingtin Levels and Aggregate Accumulation: An In vitro Model. *Front. Cell. Neurosci.* **0**, (2017).
8. Di Pardo, A. *et al.* Stimulation of Sphingosine Kinase 1 (SPHK1) Is Beneficial in a Huntington's Disease Pre-clinical Model. *Front. Mol. Neurosci.* **12**, (2019).
9. Wu, J., Lin, F. & Qin, Z. Sequestration of glyceraldehyde-3-phosphate dehydrogenase to aggregates formed by mutant huntingtin. *Acta Biochim Biophys Sin (Shanghai)* **39**, 885–890 (2007).
10. Bae, B.-I. *et al.* Mutant Huntingtin: Nuclear translocation and cytotoxicity mediated by GAPDH. *PNAS* **103**, 3405–3409 (2006).
11. Senatorov, V. V., Charles, V., Reddy, P. H., Tagle, D. A. & Chuang, D.-M. Overexpression and nuclear accumulation of glyceraldehyde-3-phosphate dehydrogenase in a transgenic mouse model of Huntington's disease. *Mol Cell Neurosci* **22**, 285–297 (2003).
12. Di Pardo, A. *et al.* Defective Sphingosine-1-phosphate metabolism is a druggable target in Huntington's disease. *Scientific Reports* **7**, (2017).
13. Di Pardo, A. *et al.* De novo Synthesis of Sphingolipids Is Defective in Experimental Models of Huntington's Disease. *Frontiers in Neuroscience* **11**, (2017).
14. Desplats, P. A. *et al.* Glycolipid and Ganglioside Metabolism Imbalances In Huntington's Disease. *Neurobiol Dis* **27**, 265–277 (2007).

15. Denny, C. A., Desplats, P. A., Thomas, E. A. & Seyfried, T. N. Cerebellar lipid differences between R6/1 transgenic mice and humans with Huntington's disease: Cerebellar lipids in Huntington's disease. *Journal of Neurochemistry* **115**, 748–758 (2010).
16. Maglione, V. *et al.* Impaired Ganglioside Metabolism in Huntington's Disease and Neuroprotective Role of GM1. *Journal of Neuroscience* **30**, 4072–4080 (2010).
17. Hunter, M., Demarais, N. J., Faull, R. L. M., Grey, A. C. & Curtis, M. A. Subventricular zone lipidomic architecture loss in Huntington's disease. *Journal of Neurochemistry* **146**, 613–630 (2018).
18. Graham, S. F. *et al.* Targeted biochemical profiling of brain from Huntington's disease patients reveals novel metabolic pathways of interest. *Biochimica et Biophysica Acta (BBA) - Molecular Basis of Disease* **1864**, 2430–2437 (2018).
19. Burke, K. A., Hensal, K. M., Umbaugh, C. S., Chaibva, M. & Legleiter, J. Huntingtin disrupts lipid bilayers in a polyQ-length dependent manner. *Biochimica et Biophysica Acta (BBA) - Biomembranes* **1828**, 1953–1961 (2013).
20. Kegel, K. B. *et al.* Polyglutamine expansion in huntingtin increases its insertion into lipid bilayers. *Biochemical and Biophysical Research Communications* **387**, 472–475 (2009).
21. Kegel, K. B. *et al.* Polyglutamine expansion in huntingtin alters its interaction with phospholipids. *Journal of Neurochemistry* **110**, 1585–1597 (2009).
22. Cheng, M.-L., Chang, K.-H., Wu, Y.-R. & Chen, C.-M. Metabolic disturbances in plasma as biomarkers for Huntington's disease. *The Journal of Nutritional Biochemistry* **31**, 38–44 (2016).
23. Colsch, B. *et al.* Characterization of the ceramide moieties of sphingoglycolipids from mouse brain by ESI-MS/MS: identification of ceramides containing sphingadienine. *Journal of Lipid Research* **45**, 281–286 (2004).
24. Vutukuri, R. *et al.* S1P d20:1, an endogenous modulator of S1P d18:1/S1P2 -dependent signaling. *FASEB J* **34**, 3932–3942 (2020).
25. Mendis, L. H. S., Grey, A. C., Faull, R. L. M. & Curtis, M. A. Hippocampal lipid differences in Alzheimer's disease: a human brain study using matrix-assisted laser desorption/ionization-imaging mass spectrometry. *Brain and Behavior* **6**, e00517 (2016).
26. Karlsson, K.-A. On the chemistry and occurrence of sphingolipid long-chain bases. *Chemistry and Physics of Lipids* **5**, 6–43 (1970).
27. Cattaneo, E., Zuccato, C. & Tartari, M. Normal huntingtin function: an alternative approach to Huntington's disease. *Nature Reviews Neuroscience* **6**, 919–930 (2005).
28. Burrus, C. J. *et al.* Striatal Projection Neurons Require Huntingtin for Synaptic Connectivity and Survival. *Cell Reports* **30**, 642-657.e6 (2020).

29. Saudou, F. & Humbert, S. The Biology of Huntingtin. *Neuron* **89**, 910–926 (2016).

30. Rui, Y.-N. *et al.* Huntingtin functions as a scaffold for selective macroautophagy. *Nat Cell Biol* **17**, 262–275 (2015).

31. Fossale, E. *et al.* Differential effects of the Huntington's disease CAG mutation in striatum and cerebellum are quantitative not qualitative. *Human Molecular Genetics* **20**, 4258–4267 (2011).

32. Halliday, G. M. *et al.* Regional Specificity of Brain Atrophy in Huntington's Disease. *Experimental Neurology* **154**, 663–672 (1998).

33. Aylward, E. H. *et al.* Onset and rate of striatal atrophy in preclinical Huntington disease. *Neurology* **63**, 66–72 (2004).

34. de la Monte, S. M., Vonsattel, J. P. & Richardson, E. P. Morphometric demonstration of atrophic changes in the cerebral cortex, white matter, and neostriatum in Huntington's disease. *J Neuropathol Exp Neurol* **47**, 516–525 (1988).

35. Rothman, K. J. No Adjustments Are Needed for Multiple Comparisons. *Epidemiology* **1**, 43–46 (1990).

36. Chen, S.-Y., Feng, Z. & Yi, X. A general introduction to adjustment for multiple comparisons. *J Thorac Dis* **9**, 1725–1729 (2017).

37. Whitwell, J. L. *et al.* [18F]AV-1451 tau-PET in progressive supranuclear palsy. *Mov Disord* **32**, 124–133 (2017).

38. Abbott, S. K. *et al.* Altered ceramide acyl chain length and ceramide synthase gene expression in Parkinson's disease: Altered Ceramide in Parkinson's Disease. *Movement Disorders* **29**, 518–526 (2014).

39. Rice, M. W., Smith, K. L., Roberts, R. C., Perez-Costas, E. & Melendez-Ferro, M. Assessment of Cytochrome C Oxidase Dysfunction in the Substantia Nigra/Ventral Tegmental Area in Schizophrenia. *PLoS ONE* **9**, e100054 (2014).
