# Supplementary material for: The long and the short of Huntington’s disease: how the sphingolipid profile is shifted in the caudate of advanced clinical cases
Source: Brain Commun. 2021 Dec 23;4(1):fcab303. doi: 10.1093/braincomms/fcab303 (PMC8833324; doi:10.1093/braincomms/fcab303)
Supplement: fcab303_Supplementary_Data [file Original Submission-Decision Letter.pdf › Original Submission-Decision Letter.pdf]

Original Submission – Decision Letter

13-Jul-2021

Dear Ms. Phillips,

Thank you for your recent submission BRAINCOM-2021-206 entitled "The long and the short of Huntington's disease: how the sphingolipid profile is shifted in the caudate of advanced clinical cases" to Brain Communications.

Your manuscript has been reviewed, and while the reviewers and editorial team appreciate many aspects of the paper, they raise some concerns that you will need to address in order for the paper to be considered for publication.

If you can address these concerns, I invite you to submit a revised manuscript and a response to each of the reviewers' comments. The reviewers' comments are included at the bottom of this message.

In addition to the reviewers' comments, please respond to our editorial comments added below to comply with our requirements for presentation of scientific data at Brain Communications.

Please format your revised manuscript according to our Instructions to Authors https://academic.oup.com/braincomms/pages/General_Instructions – a summary of key points is attached to help with this.

To revise your manuscript, click the link below or log into https://mc.manuscriptcentral.com/braincom and enter your Author Centre, where you will find your manuscript title listed under "Manuscripts with Decisions." Under "Actions," click on "Create a Revision." Your manuscript number will be appended to denote a revision.

*** PLEASE NOTE: This is a two-step process. After clicking on the link, you will be directed to a webpage to confirm. ***

https://mc.manuscriptcentral.com/braincom?URL_MASK=eb6849aa8ebe4ef8ada4ef31f7a4633a

Please upload a revised manuscript with changes tracked with track changes or in coloured text.

When submitting your revised manuscript, you will be asked to respond to the reviewer(s) comments. Please be as specific as possible in your response to all of the comments.

The editorial team at Brain Communications aims to increase transparency in scientific publishing and to demonstrate the value that peer review brings to the scientific process. Please therefore consider whether you are willing to have reviewer(s)' comments on your paper and your response published as supplementary data with the paper if it is accepted. This is entirely optional and will only be done if both the authors and reviewers agree. While submitting your revision there will be an opportunity to review or update the answer you gave previously, if necessary.

IMPORTANT: Your original files are available to you when you upload your revised manuscript. Please delete any redundant files before completing the submission.

Because we are trying to facilitate timely publication of manuscripts submitted to Brain Communications, we would appreciate your revised manuscript as soon as possible. If it is not possible for you to resubmit within 3 months, the option to submit a revision will expire, and we would then consider your revised paper as a new submission. However, if you need a little more time, please get in touch and we can extend this deadline for you.

Once again, thank you for submitting your manuscript to Brain Communications and I look forward to receiving your revision.

Best wishes

Professor Tara Spires-Jones
Founding Editor, Brain Communications

Reviewer(s)' Comments to Author:

Reviewer: 1

Comments to the Author
This manuscript describes an investigation of sphingolipid profiles in various brain regions of Huntington's disease (HD) at postmortem, versus matched controls (n=13 of each). The findings are important and are related to various previous studies in preclinical models of HD. The manuscript is generally well written, the data clearly presented and appropriately discussed. I have some suggestions below as to how the manuscript could be improved.

1. A key issue is whether the sphingolipid profile changes observed are secondary to neuronal cell loss (and associated gliosis). One way to address this is to attempt to analyse according to Von Sattel grading (as a surrogate of cell loss estimates) in the tissues used. Another complementary approach would be to study additional markers (such as NeuN for neurons, S-100-beta for astrocytes, etc.) that might indicate the amount of neuronal cell loss, and gliosis in the tissue samples used.

2. The authors use β-actin and GAPDH as Western blot housekeeper proteins. β-actin was excluded in some regions in the results. Are the authors confident neither is affected by HD? It would be informative if they could use total protein staining as an additional control for loading.

3. Are there any gender differences in their results?

4. A concern with Figures 1-4 is that presenting the data in this way means that it is impossible to judge the variance in Control samples. I would like to see control samples plotted and their variance represented. Another aspect of this data representation is that individual data points cannot be visualised. Can dot plots be overlayed, as they have been in Figure 5, etc? I realise that it may not be considered appropriate for such 'violin plots', but visualising individual data points is highly informative.

5. As the HD cases have low (close to 40) CAG repeat lengths, and therefore presumably late age of clinical HD onset, the matched controls are also fairly old (up to 78 years). Are the authors confident that the Controls didn't have any neurodegenerative pathologies that might confound the study?

6. Do the authors have any additional data for Table 1, such as cause of death and agonal state? If the HD cases died of different terminal conditions (e.g. respiratory failure versus cardiac failure) it might contribute to postmortem molecular findings.

7. Perhaps the authors could extend the discussion to make detailed comparisons between their findings and transgenic mouse models of HD, where potential confounds associated with human postmortem studies are not present. Alignment with the preclinical animal studies provides an important 'sanity check', as well as potential mechanistic insights, regarding 'cause and effect' considerations mentioned above.

Reviewer: 2

Comments to the Author
In this manuscript, Phillips and collaborators analyze the levels of major sphingolipid classes in 5 brain regions from healthy controls and patients with Huntington's disease (HD). Comparing results from 13 controls and 13 HD brains (various regions), the Authors show that the relative abundance of individual sphingolipid classes is not affected in HD compared to controls, with the exception of Hex2Cer (presumably lactosylceramide), which is increased in the cerebellum of HD post-mortem brains. However, within the specific classes of sphingolipids analyzed, the authors show a change in the relative distribution of species with long or very long acyl chains. Specifically, they describe an increase in the relative abundance of C16 and C18-containing species, concomitant with a decrease in C-22 + -containing species in the caudate, but not in other brain regions analyzed (putamen, cerebellum, grey and white frontal cortex). Because of this shift in species distribution, they investigate protein levels of CerS1 and CerS2, which are specific for the synthesis of C18 and C22-24 ceramides, respectively. They show a decrease in CerS1 in HD caudate tissue, and no changes in CerS2 levels. Furthermore, they show that levels of CerS1 inversely correlate with CAG number and positively with age at death. The implications of these findings remain unclear, although the authors speculate that the observed changes in sphingolipid species might affect membrane fluidity, promote mitochondrial dysfunction and apoptosis in HD brains.

Strengths and weaknesses of the manuscript.

The analysis of sphingolipid species in the brain of HD patients is timely and potentially important, as several studies have suggested that altered metabolism of sphingolipids and glycosphingolipids occurs in HD, Parkinson and other neurodegenerative disorders. In its current form, however, the study has a quite narrow descriptive scope. The more mechanistic data on CerS are quite preliminary, and the

proposed implications and relevance of the findings to HD pathology remain speculative. The manuscript presents a few other shortcomings that are outlined in more details below.

The mechanistic link between the observed changes in sphingolipid species and CerS expression /activity is quite underdeveloped and unclear. In spite of an increase in C16 and C18 species in the caudate, the levels of CerS1 are decreased, while the levels of CerS2 are not changed in spite of a decrease in >C22 species. The authors wisely suggest that protein levels may not correlate with enzyme activity and sphingolipid levels, since CerS can be regulated by post-translational modifications and dimerization. However, none of these possibilities is explored in the paper.

Furthermore, only CerS1 and CerS2 levels are measured, but no CerS5, which would be specific for the synthesis of C16 species that are increased in the HD caudate. Dimerization with other CerS has been shown to modulate the activity of CerS1 and CerS2, so measuring the levels of other CerSs and perform analysis in native gel conditions would help to elucidate the specific CerS dysfunctions in HD brains.

The quantification (densitometric analysis) of CerS1 in Fig. 2 is quite puzzling. The signal for HD tissue is extremely weak compared to control tissue, yet the densitometric analysis shows only a modest reduction in CerS1 expression in HD compared to controls. How is that possible? There is also significant variability in protein abundance among control tissues, which does not seem to be reflected by the spread of datapoints in the relative graphs.

Have the authors performed power analysis for this study? Considering the interindividual variability in some of the measurements reported (e.g. CerS levels), one wonders whether 13 samples per group is sufficient.
It would also help to see a comparative analysis between males and female to determine whether any difference observed is dependent on sex.

The authors claim that the observed increase in C16 and C18 ceramide species might increase susceptibility of cells to apoptosis. Yet, higher levels of CerS1, which would be responsible for the synthesis of C18 species, correlate positively with age at death in HD patients. This is in apparent contradiction with the increase in C16 and C18 species and their suggested relevance to HD, and therefore would need to be explained.

It is not clear why the authors present the correlation between CerS levels and CAG repeats and age at death, when there is clearly no correlation between the shift in sphingolipid species and levels of CerS1 and CerS2. Why not investigating the correlation between sphingolipid species (changes) and CAG repeats and age at death, to more directly investigate the role of this lipid changes in HD pathogenesis?

In human brains, sphingolipids with d20:1 sphingoid long-chain base backbone are also present in addition to d18:1. These were not reported in this study. Were they not detected?

In the discussion, some of the statements relative to the relevance and significance of the findings are too speculative based on the data presented. Furthermore, references are cited that do not appear to be relevant to the interpretation of data presented in the manuscript. For example, reference 65 in support of the statement that: "These increases in the abundance of long over very long chain sphingolipids may reflect changes to the lipid profile of neuronal membranes and/or synapses" is just a study that analyses various lipids in the soma and dendrites of neurons, but does not investigate the species of sphingolipids.

Finally, because all HD brains were stage IV, whether any lipid change observed merely a consequence of the degenerative process (with decreased neurons and increased microglia and astroglia), rather than a contributor to pathophysiology remains to be determined. It would be interesting to determine whether the shift in the sphingolipid species occurs at earlier disease stages.

Other minor comments:

The statement that a different phospholipid composition might influence brain region vulnerability to mutant HTT is speculative at this point and has not been demonstrated yet.

The authors are encouraged to abbreviate huntingtin using capital letters, HTT, according to more recent nomenclature.

Many functions have been attributed to HTT, beside its roles in neurodevelopment. This should be acknowledged in the introduction if the authors discuss HTT functions.
In the abstract and introduction, the authors should rephrase their statement that the striatum is uniquely affected, as it might generate confusion. While the striatum is the most affected, other brain

regions are also affected, as well as some peripheral tissues.

On page 5, it is not clear what the Authors mean when they state that changes in the sphingomyelin profile can greatly affect ceramide concentrations.

The polyQ length in R6/1 is not necessarily exactly 115, as stated, due to CAG instability and strain drifting in different labs. Therefore the authors are advised to remove this statement.

On page 11, the Authors state that the total abundance of each lipid class was compared between controls and HD subjects. Differences were noted for Hex2Cer in the cerebellum of HD subjects, however the data are not presented. The authors should refer to a table that shows these differences (or lack of for other lipid species).

On page 11, when referring to the various ceramide and sphingomyelin species, the authors should refer to the table where these data are shown.

In many instances a p value of 0.000 is reported. This should be changed to the actual p value.

In Fig. 5, the molecular weights for protein standards should be indicated. The "Pool" should be defined.

Concerning CerS2, multiple bands (presumably differentially glycosylated forms of the protein) are present in some of the samples (mainly HD) but not others. Whether all bands or only the bottom one was used for quantification should be specified.

Editorial office comments

- In figures 2-4, you have used t-tests to analyse the data. Please re-visit and use appropriate corrections for multiple testing.

-For each statistical test, please report in the figure legend, the N, test used, relevant test statistic, and exact p values (for example F or t values for ANOVAs and t-tests) (exact p values missing for Figures 2-4) (N number missing for Figures 5-7) (missing statistical test in Figure 5)

-In Figures 5-7, please clarify what is measured in Y-Axis.

-Please supply a graphical abstract. A graphical abstract is a visual representation of the central finding or methodology of the paper. It will be displayed in the table of contents. Authors should strive to make the graphical abstract informative, interesting, visually appealing, and straightforward (no caption needed, no abbreviations except gene names). Please upload as a separate high resolution image file (tiff preferred). The technical requirements for the graphical abstracts are:
  --size of the submitted image: 1200 pixels square at 300dpi
  --font of the text: Arial 12-16 point (smaller fonts will not be legible online)
