# Supplementary material for: The long and the short of Huntington’s disease: how the sphingolipid profile is shifted in the caudate of advanced clinical cases
Source: Brain Commun. 2021 Dec 23;4(1):fcab303. doi: 10.1093/braincomms/fcab303 (PMC8833324; doi:10.1093/braincomms/fcab303)
Supplement: fcab303_Supplementary_Data [file Revision 1- Decision Letter.pdf › Revision 1- Decision Letter.pdf]

Revision 1- Decision Letter

05-Nov-2021

Dear Ms. Phillips,

It is a pleasure to accept your manuscript entitled "The long and the short of Huntington’s disease: how the sphingolipid profile is shifted in the caudate of advanced clinical cases" for publication in Brain Communications. Any comments made by the reviewer(s) are included below.

The next step is for your paper to go through “First Look”. We will contact you again shortly if any final files or formatting changes are required for publication or if we have any queries.

If no changes are needed, your submission will be transferred to our Production Department and you will hear from the Production Editor in due course.

Video abstracts - Brain Communications encourages authors to provide a video abstract to accompany their article. These are hosted on the Brain/Brain Communications YouTube channel. If you are interested in producing a 5-min video abstract to promote your article, then please contact the Editorial Office (brcoms.editorialoffice@oup.com) for further details and guidelines.

Thank you very much for your contribution. On behalf of the Editors of Brain Communications, we look forward to your future contributions to the Journal.

Best wishes

Professor Tara Spires-Jones
Founding Editor, Brain Communications

Reviewer(s)' Comments to Author:

Reviewer: 1

Comments to the Author
The authors have revised the manuscript and addressed my comments.

Reviewer: 2

Comments to the Author
The authors have reasonably addressed my concerns.
